# Late-Stage Heteroarene Alkylation via Minisci Reaction with Gaseous Alkanes Enabled by Hydrogen Atom Transfer in Flow

**DOI:** 10.1021/acscentsci.5c00468

**Published:** 2025-05-13

**Authors:** Prakash Chandra Tiwari, Antonio Pulcinella, Emil Hodžić, Timothy Noël

**Affiliations:** Flow Chemistry Group, Van ’t Hoff Institute for Molecular Sciences (HIMS), 1234 University of Amsterdam, Science Park 904, 1098 XH Amsterdam, The Netherlands

## Abstract

The late-stage functionalization of complex molecules is a pivotal strategy in drug discovery, enabling the rapid optimization of lead compounds. However, the use of gaseous alkanes as alkylating agents in these processes remains underexplored due to their inertness and handling challenges. Here we present a photocatalytic platform that facilitates the alkylation of heteroarenes using abundant gaseous C1–C4 hydrocarbons under continuous-flow conditions. Through hydrogen atom transfer (HAT) catalysis, we achieve the efficient alkylation of pharmaceutically relevant compounds without the need for prefunctionalized reagents. Our method is not only scalable and sustainable but also extends to the functionalization of marketed drugs and natural products.

## Graphical abstract

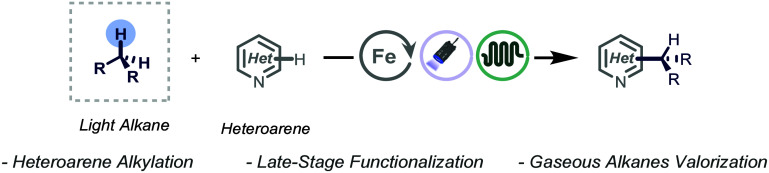

## Introduction

Nitrogen-containing heterocycles are one of the most prevalent classes of compounds found in pharmaceuticals, agrochemicals, and natural products.^1,2^ Their versatility as drug candidates stems from the ability to modulate their physicochemical properties through structural modifications.^3,4^ However, diversifying a lead compound can often be a complex process, requiring multistep synthetic sequences (Figure 1A).^5^ Traditionally, chemists have relied on *de novo* strategies, which are often step- and atom-inefficient, to create common intermediates that eventually lead to the desired analogues of a parent drug candidate.^6^ As a result, late-stage functionalization (LSF) has gained significant traction in modern synthetic chemistry, offering a more efficient alternative (Figure 1A).^1,2,5,7–10^ Over the past decade, synthetic methods that enable the generation of structural analogues without the need for prefunctionalization have greatly advanced medicinal chemistry efforts.^11,12^

**Figure 1:**
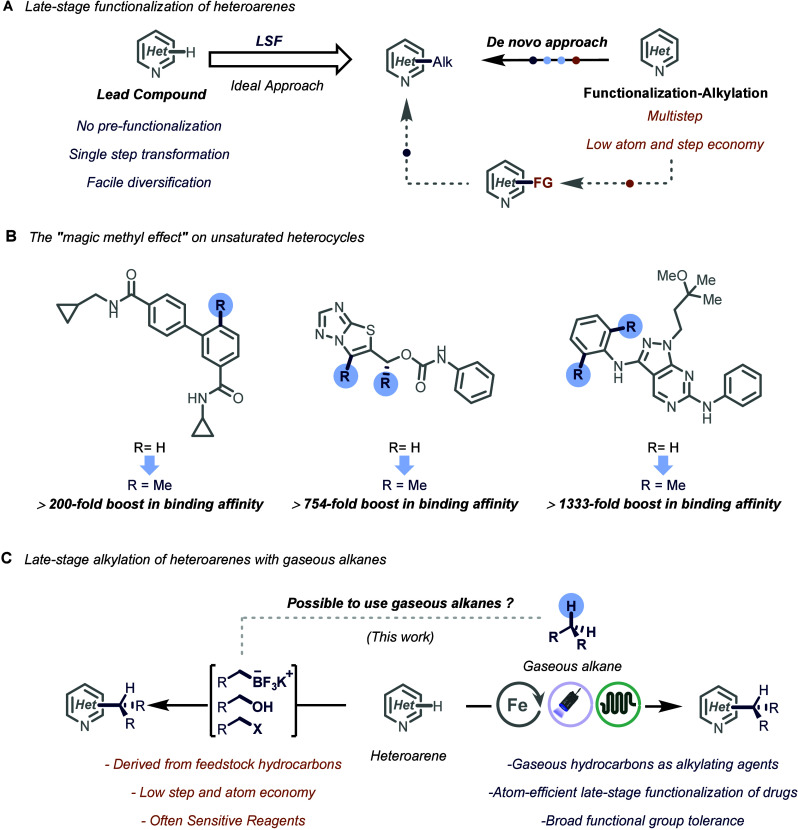
A. Late-stage functionalization of heteroarenes. B. Impact of light alkyl fragment incorporation in drugs. C. Alkylation of heteroarenes using gaseous alkanes.

In medicinal chemistry, minor structural changes to a drug molecule can significantly enhance its activity or pharmacological properties, such as logD, polar surface area, solubility, and p*K*_a_.^13–17^ To achieve these improvements, the introduction of small alkyl fragments (C1–C4) is commonly used to optimize the ADME (absorption, distribution, metabolism, and excretion) profile of lead candidates.^5,14,15,18^ A prime example of this trend is the “magic methyl effect”, where the replacement of a single hydrogen atom with a methyl group can drastically alter both the biological and physical properties of the parent molecule (Figure 1B).^5,14,15,18^ Traditionally, the incorporation of small alkyl fragments has relied on methods like transition-metal-catalyzed cross-couplings,^19–22^ direct metalation,^23^ or C­(sp^2^)–H functionalization of the corresponding arene.^24,25^ However, in recent years, Minisci-type reactions have emerged as a reliable approach for the late-stage alkylation of complex heteroarene molecules.^26–35^ While mild photocatalytic variants have demonstrated success in generating alkylated analogues of heavily decorated *N*-heterocycles, challenges remain in the efficient incorporation of small alkyl units (C1–C4).^36,37^ Specifically, these methods typically require prefunctionalized radical precursors, such as carboxylic acids,,^34,38,39^ boronic acids,^40–42^ alcohols,^26,32^ peroxides,^36^ and alkyl halides,^43–45^ to produce alkylated analogues of complex heterocycles (Figure 1C). Notably, these approaches often lack modularity and do not offer a unified strategy to incorporate a wide variety of short alkyl fragments.

On this basis, the use of gaseous alkanes as C1–C4 radical donors represents a straightforward and efficient strategy to bypass the need for traditional alkylating agents, which often have low sustainability and generate significant chemical waste in their preparation and subsequent use.^46,47^ This approach could provide a modular platform for the late-stage alkylation of medicinally relevant heteroarenes using readily available, low-cost, and atom-efficient feedstocks. However, the high bond dissociation energy (BDE), the limited solubility in common organic solvents, and the gaseous nature of these alkanes still pose challenges to their routine use as chemical reagents.^47^ To address these obstacles, our group has recently demonstrated that hydrogen atom transfer (HAT) photocatalysis under continuous-flow conditions offers an effective strategy to generate carbon-centered radicals from abundant and inert volatile alkanes.^48–54^ The use of continuous-flow reactors has notably advanced the chemistry of gaseous reagents, improved gas–liquid mass transfer, and enhanced the practicality of handling such reagents.^55^ Despite these advancements, only a limited number of reaction manifolds currently utilize gaseous hydrocarbons as alkylating agents.^48–51,56^ While there are some examples in the literature,^57–59^ no general or practical protocol has been established for the direct introduction of C1–C4 alkyl fragments via Minisci-type reactions using light alkanes. With this in mind, we propose that photoinduced iron-catalyzed ligand-to-metal charge transfer (LMCT)^60–62^ could be employed to cleave the strong C­(sp^3^–H) bonds of C1–C4 alkanes under mild conditions, enabling efficient and late-stage photocatalytic alkylation of heteroarenes (Figure 1C). Building on this concept, in this study, we present a modular and robust platform for the introduction of short alkyl fragments into a variety of pharmaceutically relevant heteroarenes by using inexpensive carbon-based gaseous feedstocks. Our method was successfully applied to the late-stage modification of marketed drugs, and we demonstrated its scalability using a commercially available continuous-flow photoreactor.

## Results and Discussion

### Reaction Optimization

To explore the feasibility of our approach, we began by investigating the photocatalytic ethylation of commercially available lepidine (**1**) using ethane as a C2 synthon under flow conditions. After optimizing key reaction parameters, we found that a 0.1 M acetonitrile solution of lepidine (**1**), containing 20 mol % FeCl_3_, 1.2 equiv of *N*-fluoro-succinimide (NFSI), and 3.5 equiv of trifluoroacetic acid (TFA), when combined with a stream of gaseous ethane (40:1 (V/V) = 23 equiv) and irradiated with 365 nm light, yielded the desired alkylated heteroarene (**2**) in 65% ^1^H NMR yield (Table 1, Entry 1). Specifically, the reaction mixture was introduced into a continuous-flow microreactor (i.d. = 0.76 mm, 2.8 mL volume) and irradiated using UV-A light (chip-on-board LED, λ = 365 nm, 144 W optical power) (see Supporting Information) for a total residence time of 60 min. A back pressure regulator at the reactor outlet ensured a constant reaction pressure (52 bar), allowing for the complete liquefaction of ethane. Prolonging the residence time to 90 min, however, resulted in a lower yield, likely due to product decomposition via HAT (Table 1, Entry 2, 60% yield). Increasing the amounts of either FeCl_3_ or TFA also led to reduced yields (Table 1, Entries 3 and 4, 60% and 42%, respectively). Adjusting the gas-to-liquid ratio (G:L) to 60:1 led to a 50% yield (Table 1, Entry 5). Interestingly, the use of PIFA significantly reduced the yield of the reaction (Table 1, Entry 6, 12% yield). Omission of any of the reaction components resulted in either complete inhibition (Table 1, Entry 7) or significantly lower yields of the desired alkylated product (Table 1, Entries 8, 9, see Supporting Information).

**Table 1:**
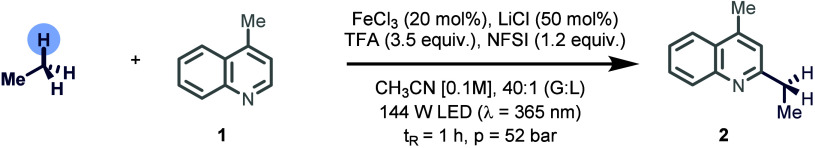
Optimization of the Alkylation of **1** with Ethane^a^

Entry	Deviation	Yield of **2** ^b^	Yield of **1** ^b^
1	none	65%	20%
2	*t*_R_ = 90 min	60%	26%
3	FeCl_3_ (40 mol %)	60%	15%
4	TFA (5.0 equiv)	42%	28%
5	60:1 (G: L)	50%	15%
6	PIFA (2.3 equiv)	12%^c^ ^,^ ^d^	76%
7	No light	n.d^c^	45%^c^
8	No NFSI	25%^c^	30%^c^
9	No FeCl_3_	43%^c^	40%^c^

a Reactions performed on a 0.2 mmol scale, 144 W of 365 nm LEDs.

b Yields determined by ^1^H NMR using trichloroethylene as an external standard.

c See Supporting Information for experimental details.

d Reaction run in trifluoroethanol (0.1 M), without FeCl_3,_ LiCl, NFSI.

### Substrate Scope

With the optimized conditions in hand, we began investigating the scope of the photochemical ethylation of heteroarenes using ethane as the alkylating agent (Figure 2). Notably, lepidine and the more electron-rich 4-methoxyquinoline afforded the desired products in moderate to good yields (**2**, **3**). Similarly, 2-phenylquinoline was efficiently alkylated under the optimized conditions (**4**). As for electron-deficient heteroarenes, we observed that decreasing light intensity (14–29 W) and lowering the residence time (30–60 min) were necessary to maximize yield and selectivity during this study.^63^ Specifically, under these modified reaction conditions, a variety of halogenated quinolines were effectively converted into their corresponding ethylated analogues in moderate to good yields (**5**–**7**). Moreover, derivatives of quinoline-4-carboxylic acid were successfully alkylated using this photocatalytic protocol (**8**–**10**). Of particular note, allylic and menthol ester analogues, which present C­(sp^3^)–H bonds with lower bond dissociation energy (BDE) compared to ethane, afforded the desired products in good yields (**9** and **10**). The method was successfully extended to other classes of pharmaceutically relevant heterocycles, such as phenanthridine, benzothiazole, 4-hydroxyquinazoline, phthalazine, and 2-chloro-3-ethylquinoxaline. In all cases, the corresponding ethylated analogues were isolated in synthetically useful yields (**11**–**15**).

**Figure 2:**
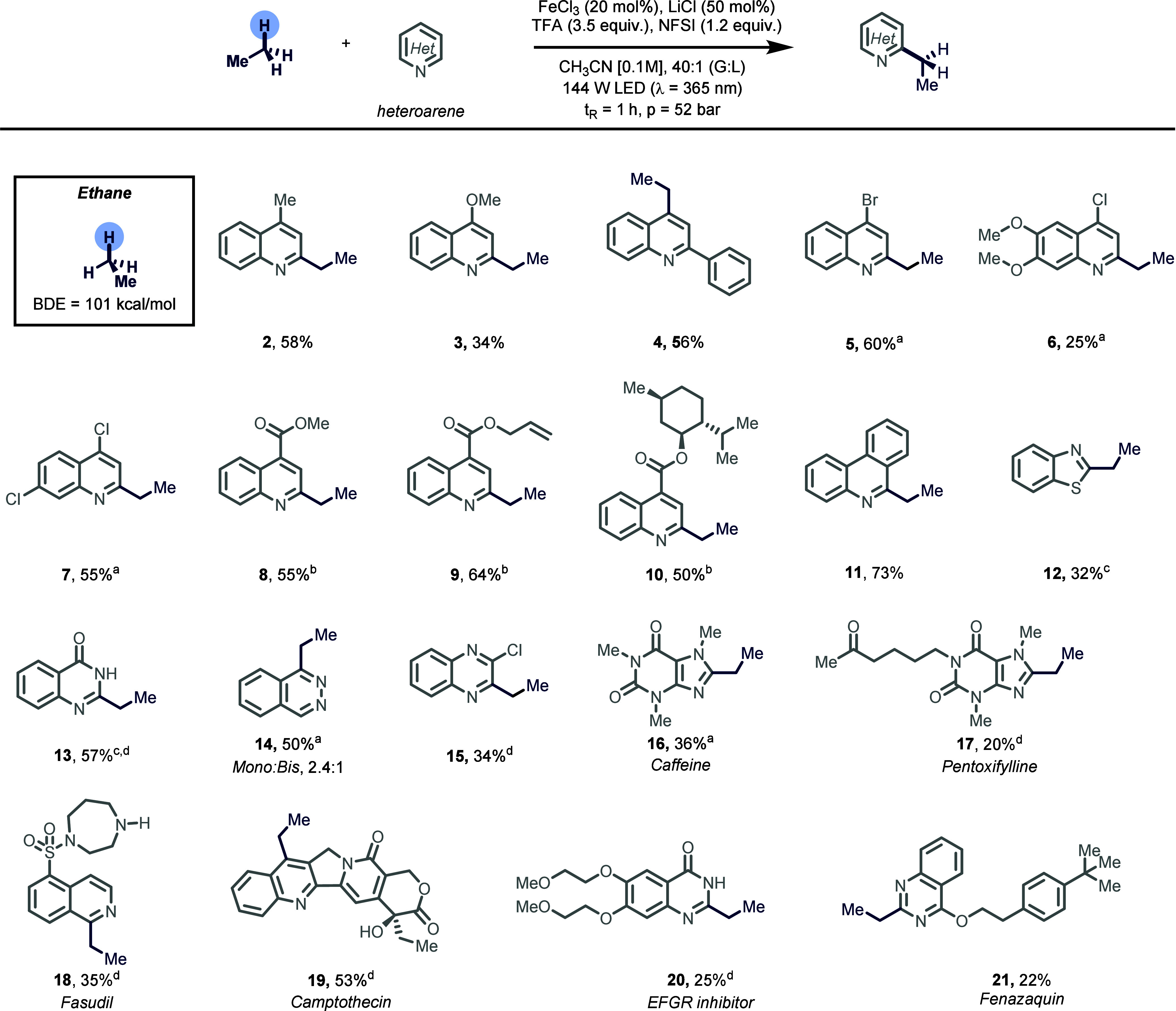
Scope of the alkylation of heteroarenes using ethane as an alkylating agents. Reaction conditions: heteroarene (0.2 mmol. 1.0 equiv), FeCl_3_ (20 mol %), NFSI (1.2 equiv), LiCl (50 mol %), and TFA (3.5 equiv) in 2 mL of CH_3_CN, G:L = 40:1, 144 W of 365 nm LEDs. All yields are those of isolated products (Supporting Information for experimental details). [a] TFA (2.5 equiv) and 29 W of 365 nm LEDs were used. [b] TFA (2.5 equiv), 14 W of 365 nm LEDs, and 30 min residence time. [c] TFA (2.5 equiv), 30 min residence time. [d] See Supporting Information for experimental details.

To further demonstrate the synthetic utility of this protocol, we targeted the late-stage incorporation of ethyl fragments into natural products and drugs. As shown in Figure 2, the xanthine cores of caffeine and pentoxifylline could be effectively alkylated (**16**, **17**). Interestingly, selective monoethylation at the C1 position of fasudil was achieved despite the presence of multiple weak aliphatic C­(sp^3^–H) bonds prone to undergo HAT (**18**, 35%). C4-selective ethylation of the anticancer natural product camptothesin, bearing sensitive functional groups, such as alcohol, lactone, and pyridone, was successfully achieved in 53% yield (**19**). Similarly, selective C2 functionalization of an EGFR tyrosinase inhibitor was accomplished, yielding the corresponding alkylated analogue (**20**, 25%). Fenzaquine, a quinazoline-based insecticide, could be readily ethylated, offering straightforward access to a previously undescribed analogue (**21**, 22%). Notably, pyridines remained unreactive under the reaction conditions, leading to quantitative recovery of the starting material (see Supporting Information, section 9).

Next, we explored whether the protocol could be extended to the incorporation of different small alkyl fragments using other abundant light alkanes. By simply switching gas bottles, both propane and butane served as effective alkylating agents under the optimized photocatalytic conditions, affording the corresponding functionalized heterocycles in moderate to good yields (**22**–**29**). For both gases, preferential functionalization occurred at the methylene site, yielding regioisomeric mixtures (i.e., secondary vs primary C–H bond) that could be readily separated via column chromatography, thus providing access to both regioisomers in a single experiment.

After establishing the generality of the developed alkylation of heteroarenes using gaseous C2–4 alkanes, we turned our attention to the introduction of a methyl unit through the activation of methane, which is one of the most abundant carbon-based feedstocks. Despite the synthetic appeal of this C1 building block, the extraordinary inertness of the C­(sp^3^)–H bonds (BDE = 105 kcal/mol) has strongly limited its use as a methylating agent in organic chemistry. Reoptimization of the reaction parameters revealed that 20 mol % of FeCl_2_, in combination with acetonitrile–TFA (3:1) as the solvent system and a residence time of 30 min, was optimal for the formation of the desired methylated phenanthridine (**30**) (see Supporting Information). Using this set of conditions, a series of electron-neutral, electron-deficient, and halogenated quinolines were methylated in medicinal-chemistry-relevant quantities (**30**–**35**).

Furthermore, the scalability of the protocol was demonstrated through the gram-scale ethylation of 4,7-dichloroquinoline, which afforded **7** in 46% isolated yield alongside 28% of unreacted starting material (Figure 3, see Supporting Information). Similarly, the methylation of acridine proceeded smoothly on a 1.8 mmol scale, affording derivative **31** in a 45% isolated yield. Taken together, these results highlight the utility of flow technology to enable the use of gaseous alkanes as alkylating agents at substantial reaction scales, previously unattainable (Figure 3).

**Figure 3:**
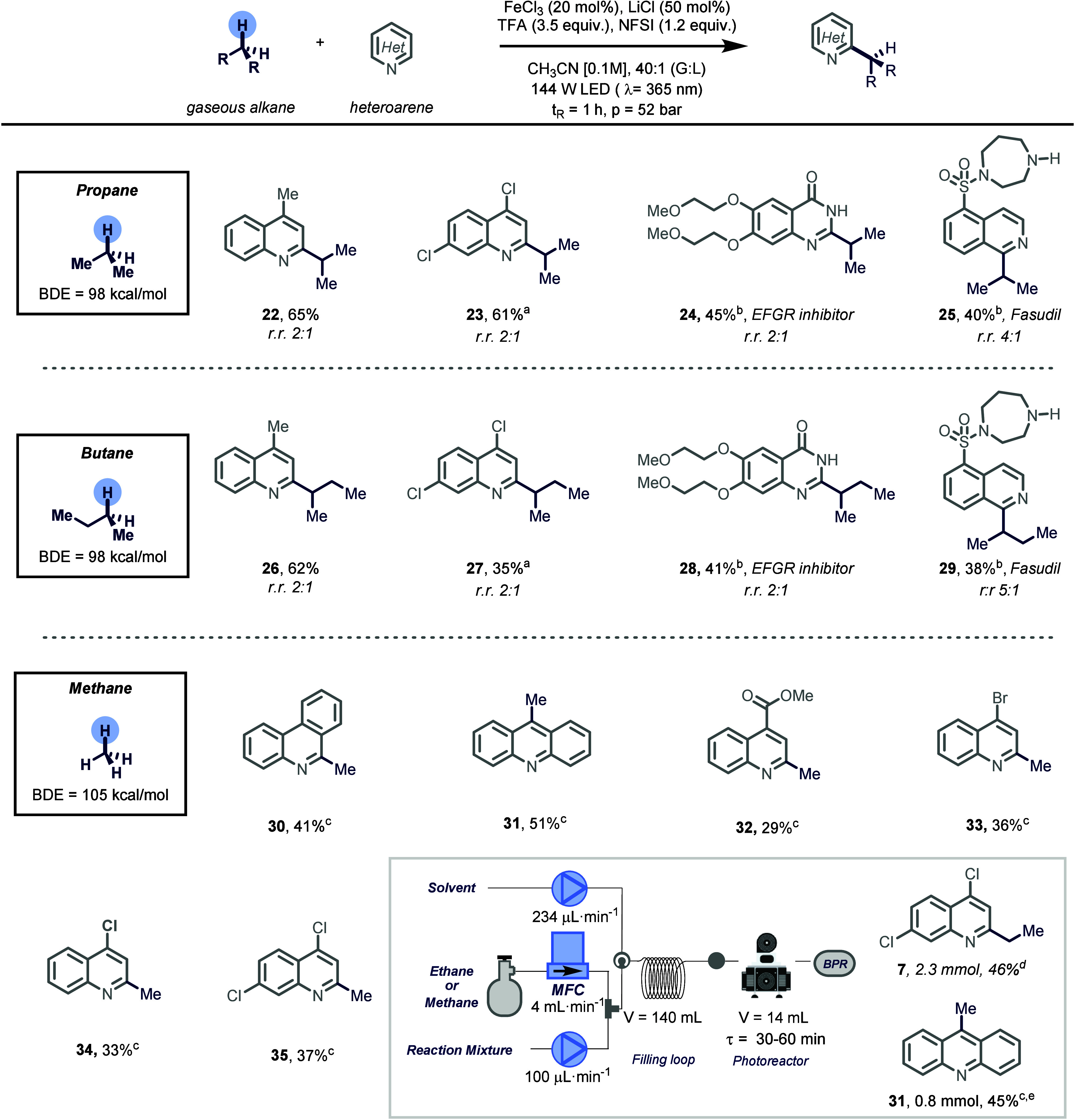
Scope of the alkylation of heteroarenes using propane, butane, and methane as alkylating agents. Reaction conditions: heteroarene (0.2 mmol. 1.0 equiv), FeCl_3_ (20 mol %), NFSI (1.2 equiv), LiCl (50 mol %), and TFA (3.5 equiv) in 2 mL of CH_3_CN, G:L = 40:1, 144 W of 365 nm LEDs. All yields and regioisomeric ratios (r.r.) are those of isolated products (see Supporting Information for experimental details). [a] TFA (2.5 equiv) and 29 W of 365 nm LEDs were used. [b] See Supporting Information for experimental details. [c] Reaction conditions: heteroarene (0.3 mmol, 1.0 equiv), FeCl_2_ (20 mol %), NFSI (1.2 equiv), and LiCl (50 mol %) in 3 mL of CH_3_CN–TFA (3:1), G:L = 40:1, 144 W of 365 nm LEDs with 30 min of residence time. All yields are those of isolated products (see Supporting Information for experimental details). [d] Scale-up conditions: heteroarene (5.0 mmol. 1.0 equiv), FeCl_3_ (20 mol %), NFSI (1.2 equiv), LiCl (50 mol %), and TFA (2.5 equiv) in 25 mL of CH_3_CN (0.2 M), G:L = 20:1, 29 W of 365 nm LEDs (Supporting Information for experimental details). [e] Scale-up performed at a 1.8 mol scale.

Additionally, we showcase the applicability of the protocol through the rapid and divergent synthesis of alkylated analogues of the fungicide quinoxyphen (Figure 4A, **36**–**41**). Using a homologous series of gaseous C1–4 hydrocarbons as alkylating agents, six analogues were rapidly obtained in synthetically useful yields, showing the practicality of this methodology. Finally, we demonstrate the possibility of achieving high regioselectivity for the branched isomer when using propane and butane as alkylating agents (Figure 4B). Building on the reported high methylene selectivity of alkoxy radicals,^64,65^ we established new conditions using 2.5 equiv of (bis­(trifluoroacetoxy)­iodo)­benzene (PIFA) in trifluoroethanol (0.1 M) under 456 nm light irradiation (Method B, Figure 4B). Pleasingly, the new method afforded the desired alkylated heteroarenes in good to excellent yields and selectivity (**22**, **23**, **26**, and **27**).

**Figure 4:**
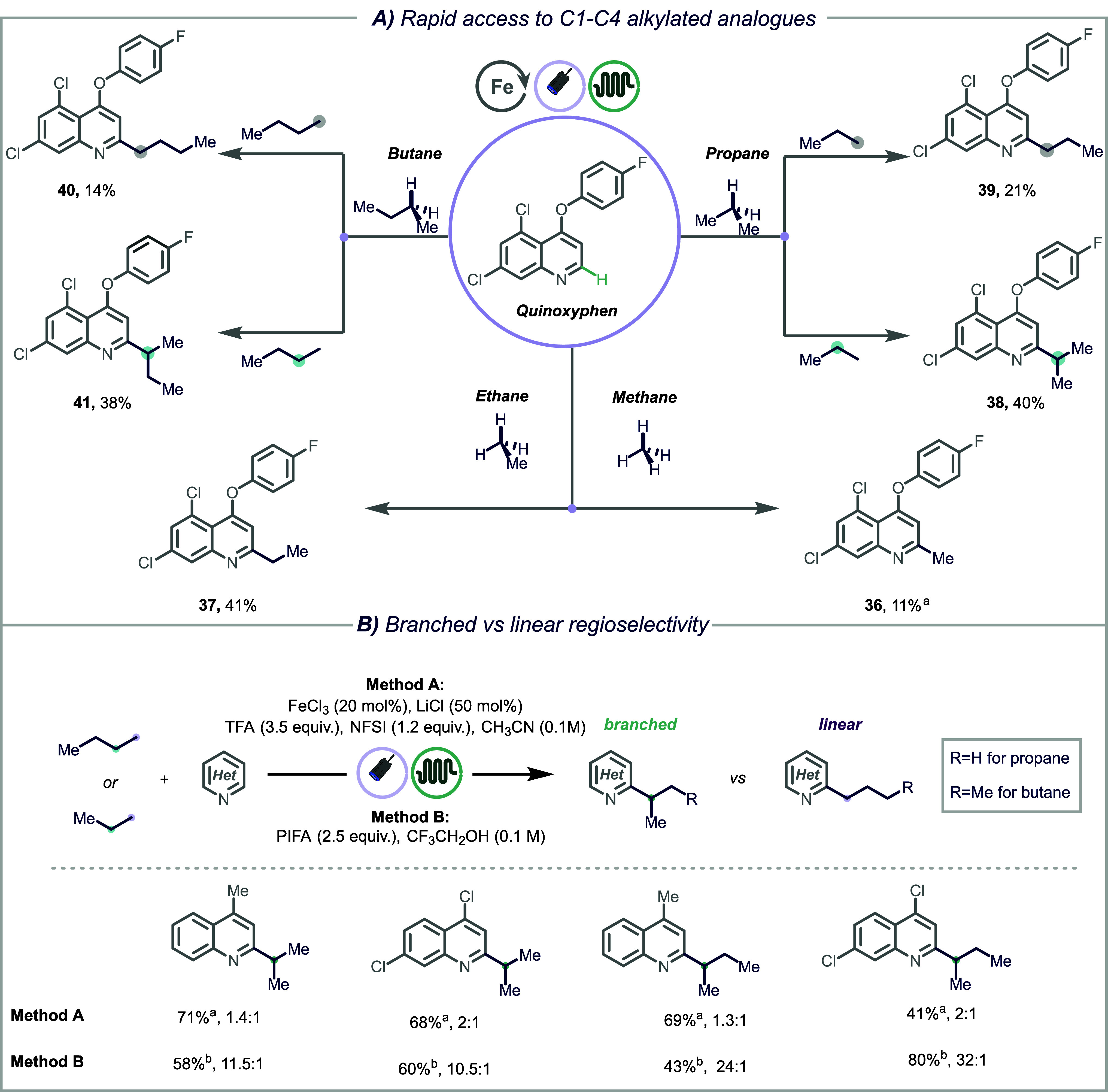
A) Rapid diversification of quinoxyphen using C1–C4 gaseous hydrocarbons as alkylating agents. Reaction conditions: quinoxyphen (0.2 mmol. 1.0 equiv), FeCl_3_ (20 mol %), NFSI (1.2 equiv), LiCl (50 mol %), and TFA (3.5 equiv) in 2 mL of CH_3_CN, G:L = 40:1, 144 W of 365 nm LEDs. All yields and regioisomeric ratio (r.r.) are those of isolated products (see Supporting Information for experimental details). [a] FeCl_2_ (20 mol %), NFSI (1.2 equiv), and LiCl (50 mol %) in 2 mL of CH_3_CN–TFA (3:1), G:L = 40:1, 144 W of 365 nm LEDs. B) Branched versus linear regioselectivity for propane and butane. Reaction conditions: heteroarene (0.2 mmol. 1.0 equiv), FeCl_3_ (20 mol %), NFSI (1.2 equiv), LiCl (50 mol %), and TFA (3.5 equiv) in 2 mL of CH_3_CN, G:L = 40:1, 144 W of 365 nm LEDs. All yields are those of isolated products, and regioisomeric ratios (r.r.) were determined by GC-MS analysis (see Supporting Information for experimental details). [a] FeCl_2_ (20 mol %), NFSI (1.2 equiv), and LiCl (50 mol %) in 2 mL of CH_3_CN–TFA (3:1), G:L = 40:1, 144 W of 365 nm LEDs. [b] Heteroarene (0.2 mmol. 1.0 equiv), PIFA (2.5 equiv) in 2 mL of CF_3_CH_2_OH, G/L = 40:1, 184 W of 456 nm LEDs. All yields are those of isolated products, and regioisomeric ratios (r.r.) were determined by GC-MS analysis (see Supporting Information for experimental details).

## Conclusions

In this study, we developed a modular and robust platform for the late-stage alkylation of heteroarenes using readily available gaseous alkanes as C1–C4 radical donors. By employing hydrogen atom transfer (HAT) photocatalysis under continuous-flow conditions, we demonstrated the efficient alkylation of pharmaceutically relevant heterocycles, including quinolines and marketed drugs. The method overcomes traditional challenges associated with gaseous alkanes, such as their high bond dissociation energy and poor solubility, offering a scalable and sustainable approach to the functionalization of complex molecules. Our results highlight the broad applicability of this strategy, particularly in lead optimization and drug discovery, where rapid and efficient diversification of bioactive compounds is crucial. The ability to directly use abundant gaseous feedstocks opens new possibilities for sustainable and practical synthetic routes in medicinal chemistry.

## Supplementary Material



## Notes

Experimental details, used materials, sample preparation, analytical data (PDF)
