# Supplementary material for: Late-Stage Heteroarene Alkylation via Minisci Reaction with Gaseous Alkanes Enabled by Hydrogen Atom Transfer in Flow
Source: ACS Cent Sci. 2025 May 13;11(6):910–7. doi: 10.1021/acscentsci.5c00468 (PMC12203263; doi:10.1021/acscentsci.5c00468)
Supplement: Supplementary file 1 [file oc5c00468_si_001.pdf]

Supplementary Information

Late-Stage Heteroarene Alkylation via Minisci Reaction with Gaseous Alkanes Enabled by Hydrogen Atom Transfer in Flow

Prakash Chandra Tiwari[1], Antonio Pulcinella[1], Emil Hodžić[1] and Timothy Noël[1,]*

[1]Flow Chemistry Group, Van 't Hoff Institute for Molecular Sciences (HIMS), University of Amsterdam, Science Park 904, 1098 XH Amsterdam, The Netherlands.

** Email: t.noel@uva.nl (T. Noel)*

Keywords: Light Alkanes • Minisci • Heteroarenes • Flow Chemistry • Photocatalysis • Late-Stage Functionalization

This PDF file includes:

Materials and Methods

Figures S1 to S6

Tables S1 to S7

References

NMR Data

Table of contents

1. General information **S3**

2. Reactor Design **S4**

2.1 Flow Equipment S4

2.2 Vapourtec Setup S5

2.3 Eagle Reactor S6

3. General Procedure for the Optimization of gas-liquid Reactions in Flow **S7**

4. Reaction Optimization **S8**

4.1 Catalysts Loading Screening S8

4.2 Residence Time Screening S9

4.3 Additive Screening S9

4.4 Oxidants Screening S10

4.5 TFA Equivalents Screening S10

4.6 Light Intensity Screening for Electron Poor Substrates S11

4.7 Optimization for Methane S13

4.8 Optimization for Scale-up S14

4.9 Optimization of the PIFA mediated HAT Minisci reaction S15

5. UV-Vis Characterization **S16**

6. General Procedures (GP) **S22**

6.1 (GP1) Ethane S22

6.2 (GP2) Propane S22

6.3 (GP3) Butane S23

6.4 (GP4) Methane S23

6.5 Scale-up (Ethane) S23

6.6 Scale-up (Methane) S24

6.7 (GP5) Propane - PIFA S24

6.8 (GP6) Butane - PIFA S24

7. Characterization data of synthesized compound **S25**

8. Proposed Mechanism **S41**

9. Limitation of the scope **S42**

10. References **S43**

11. NMR Spectra **S44**

12. GC-MS Data **S99**

1. General information

Materials. All reagents and solvents were used as received without further purification. Reagents and solvents were bought from Sigma Aldrich, TCI and Fluorochem. Technical solvents were bought from VWR International and used as received. N-butane gas with 3.5purity was purchased from Praxair, propane gas with 2.5purity was purchased from Benegas, ethane gas with 3.5purity was purchased from Gerling and Holz and Co and methane gas with 4.5purity was purchased from Nippon gases. Disposable syringes were purchased from Laboratory Glass Specialist. Syringe pumps were purchased from Chemix Inc. model Fusion 200 Touch. All capillary tubing, microfluidic fittings and Back Pressure Regulator (BPR) were purchased from IDEX Health & Science. Product isolation was performed automatically, by a Biotage® Isolation Four, with Biotage® SNAP KP-Sil 4 or 10 g flash chromatography cartridges, or manually, using silica (P60, SILICYCLE). TLC analysis was performed using Silica on aluminum foils TLC plates (F254, SILICYCLE) with visualization under ultraviolet light (254 nm and 365 nm) or appropriate TLC staining (potassium permanganate). Organic solutions were concentrated under reduced pressure on a Büchi rotary evaporator (in vacuo at 40 ºC, ~5 mbar). The synthesis of starting materials (2R,5S)-2-isopropyl-5-methylcyclohexyl quinoline-4-carboxylate[1] and methyl quinoline-4-carboxylate[2] were prepared following literature procedures.

NMR spectroscopy. ^{1}H (400 and 300 MHz), ^{13}C (101 and 128 MHz), ^{19}F (282 and 376 MHz) spectra were recorded at ambient temperature using Bruker AV 300-I, AV 400 and AV 500-NEO. ^{1}H NMR spectra are reported in parts per million (ppm) downfield relative to $CDCl_3$ (7.26 ppm) and all 13C NMR spectra are reported in ppm relative to $CDCl_3$ (77.16 ppm) unless stated otherwise. The multiplicities of signals are designated by the following abbreviations: s (singlet), d (doublet), t (triplet), q (quartet), p (pentet), sext (sextet), m (multiplet), dd (doublet of doublets), dt (doublet of triplets), td (triplet of doublets), ddd (doublet of doublet of doublets). Coupling constants (J) are reported in hertz (Hz). NMR data was processed using the MestReNova 14 software package. Known products were characterized by comparing to the corresponding ^{1}NMR, ^{13}C NMR, ^{19}F NMR with those available in the literature.

Mass spectrometry. High resolution mass spectra (HRMS) were collected on an AccuTOF GC v 4g, JMS-T100GCV Mass spectrometer (JEOL, Japan).

Determination of Regioisomeric Ratio. All the reported regioisomeric ratios referred to the isolated products after column chromatography.

2. Reactor Design

2.1 Flow Equipment

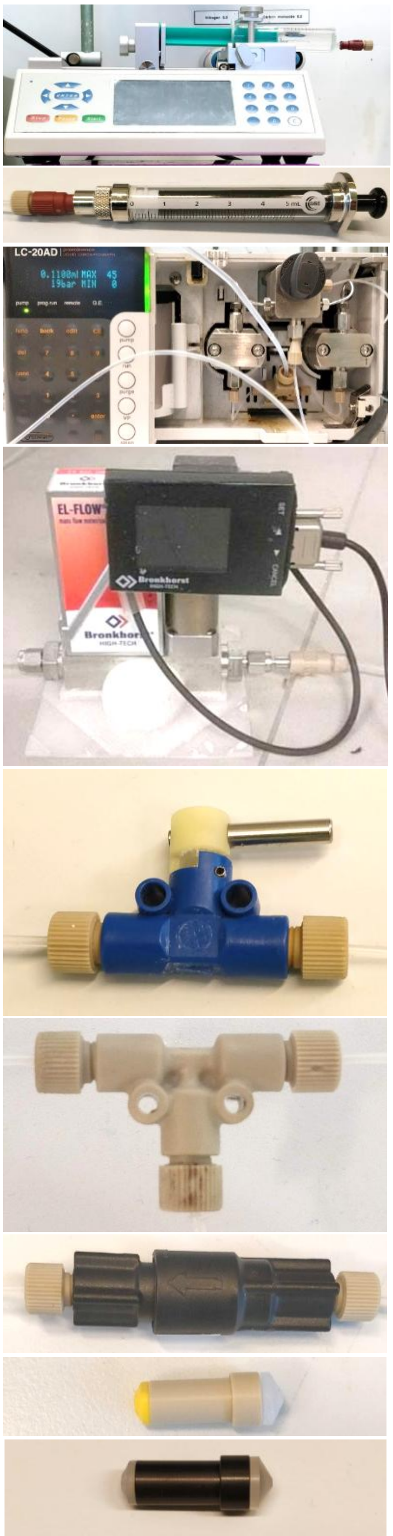

Syringe pump
(Chemyx Fusion 200)

Gastight syringe (SGE
Luer Lock 5)

HPLC pump
(Shimadzu LC-20AD)

Mass Flow Controller
(Bronkhorst EL-)

FLOW - Shut-Off

Valve (IDEX P-783) T-
mixer (IDEX P-712)

BPR holder (IDEX P-
789)

BPR cartridge

(IDEX P-789)

Figure S1: Flow equipment used for the photocatalytic reactions.

2.2 Vapourtec Setup

Preliminary experiments were carried out with a Vapourtec UV-150 photochemical reactor setup, equipped with a a 60 W 365 nm LED lamp (Figure S2).

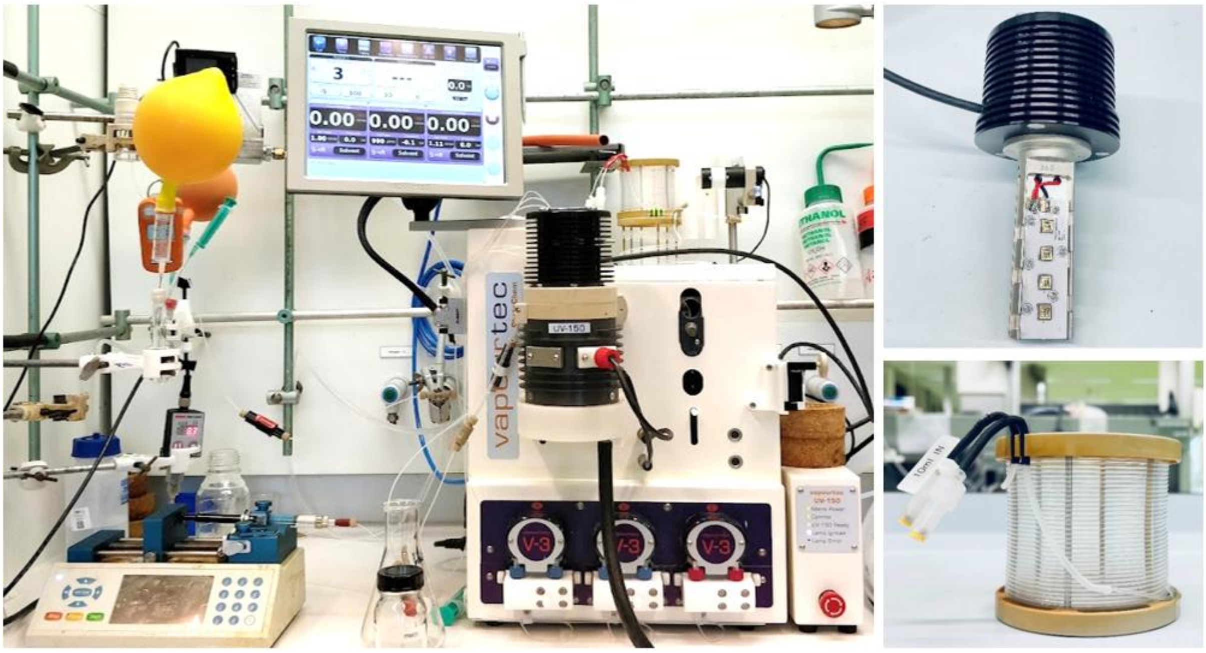

Figure S2: Overview of the Vapourtec Setup and details of the LEDs and PFA reactor coil (1.3 mm ID, 1.6 mm OD, 10 mL).

2.3 Eagle Reactor

A Signify photochemical reactor is used, consisting of a base assembly with six 365 nm UV-A chip-on-board light modules.[5] Each of these light source modules contain a fan and a heat sink to efficiently dissipate heat generated through the high power LEDs. Also the head cap assembly contains blowers to cool the interior of the reactor system, to reduce undesired thermal side-reactions. The LED modules and chamber cooling blowers are connected to a driver box, allowing to set the current of each of the LED modules individually, as well as the rotation speed of the cooling blowers. The six LED modules (365 nm, max. 144 W combined optical output power) are positioned in an hexagonal form around an aluminum cylinder support (80 mm height, 75 mm diameter), which has the reactor coil wrapped around (FEP capillary tubing: 1.6 mm OD, 0.8 mm ID, 11 mL volume or 0.5 mm ID, 2.8 mL volume).

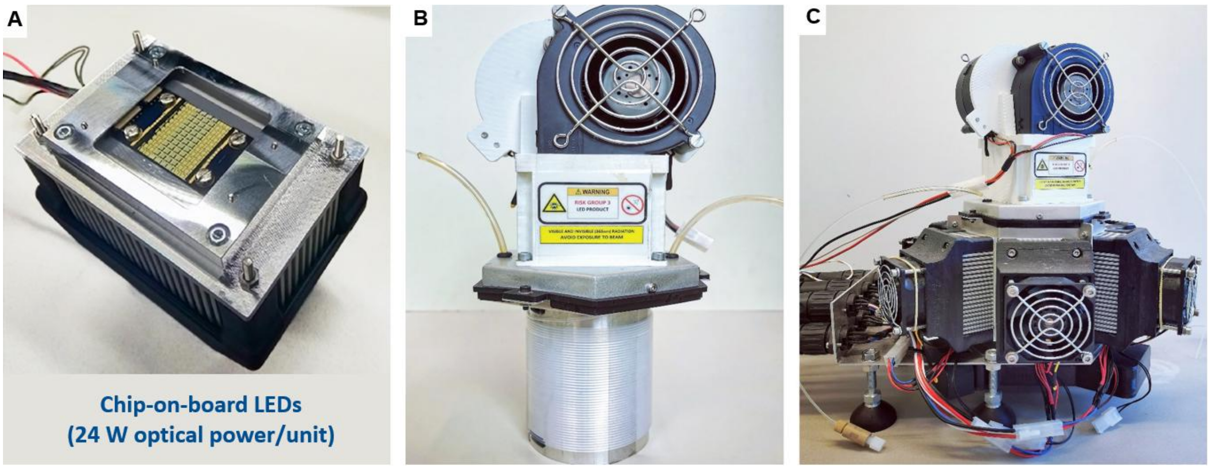

Figure S3 Signify Eagle Reactor with (A) six chip-on-board LED modules, (B) head assembly with reactor coil, and (C) complete assembly with fans, heat sinks and LED modules.

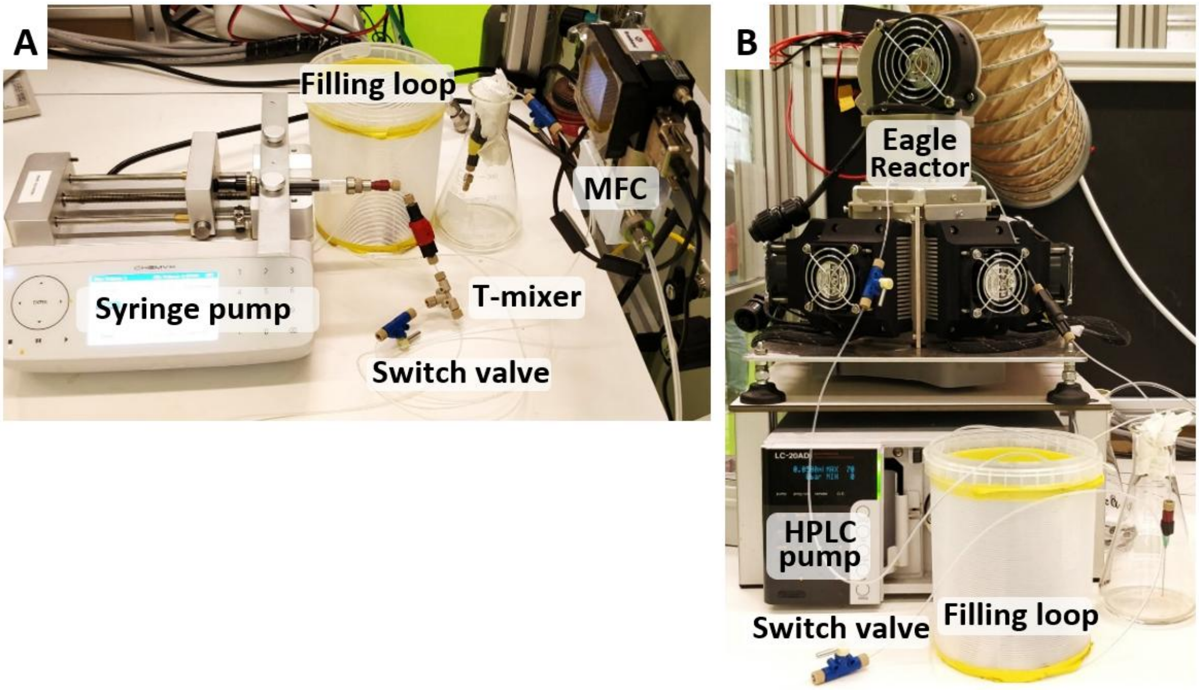

Figure S4: Overview of Setup for (A) loop filling of gas- and liquid, (B) photochemical reaction with Signify Eagle Reactor.

3. General Procedure for the Optimization of gas-liquid Reactions in Flow

An elaborate description of the procedure for optimizing gas-liquid and gas-gas-liquid reactions in flow is described in the supporting information of our previous work on photocatalytic carbonylation of light and heavy hydrocarbons. [6] The relevant descriptions and calculations for this work are repeated here. For reactions above the maximum pressure of the liquid stream (syringe pump) or above the maximum pressure of the gas stream (pressure of the gas cylinder or reducer), a loop filling method is applied (Figure S5 A). With this method, the gas and liquid stream are first combined in a filling loop, then pressurized with a HPLC pump and finally injected into the reactor coil under the desired flow rate with the HPLC pump. (Figure S5 B)

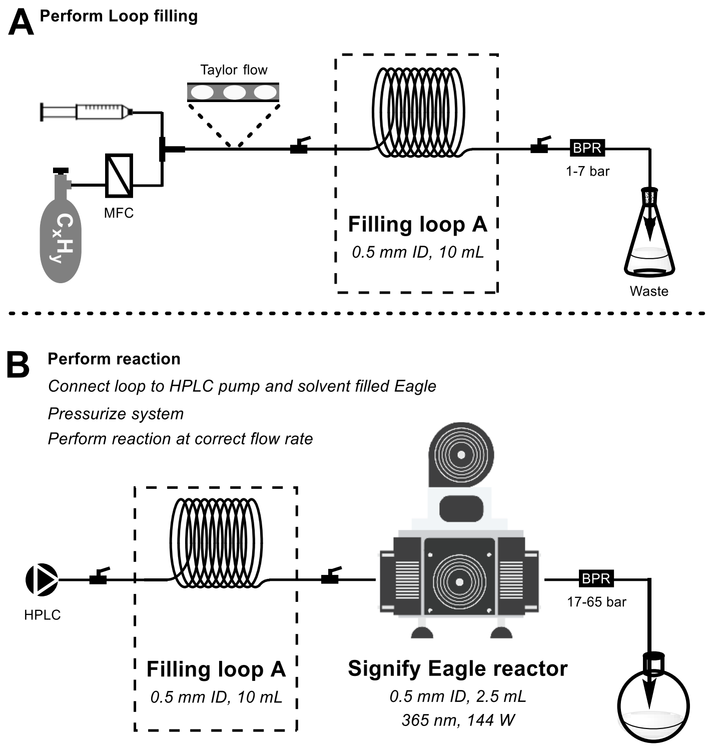

Figure S5: Schematic representation of gas-liquid reactions performed with a loop filling method. (A) Loop filling of gas-liquid mixture, (B) Pressurizing the system and run the reaction using the correct flow rate. BPR: Back-Pressure Regulator.

The flow over the BPR only starts when the system has reached the design pressure of the BPR. The reaction is then performed through irradiation of the solution inside the reactor and the reaction mixture is collected at the outlet

4. Reaction Optimization

The alkylation of Lepidine (**1**) using ethane was chosen as model reaction and an initial screening of reaction parameters was performed in the Signify Eagle Reactor (365 nm, 144 W output power) as described above.

4.1 Catalysts Loading Screening

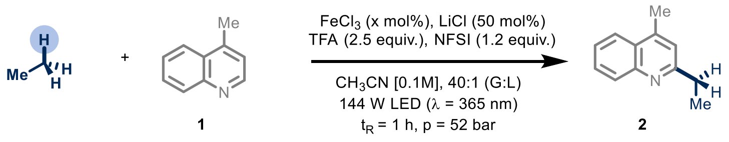

Table S1

Catalysts Loading	Yield of **2**	Yield of **1**
$FeCl_3$ (10 mol%)	48%	14%
**$FeCl_3$ (20 mol%)**	**60%**	**30%**
$FeCl_3$ (40 mol%)	60%	15%
$FeCl_3$ (0 mol%)	43%	40%

4.2 Residence Time Screening

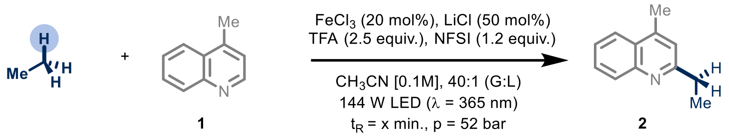

Table S2

Residence Time [min]	Yield of **2**	Yield of **1**
45 min	47%	45%
**60 min**	**60%**	**30%**
90 min	60%	26%

4.3 Additive Screening

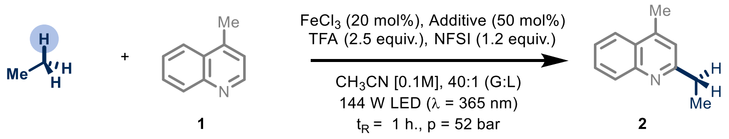

Table S3

Additive	Yield of **2**	Yield of **1**
**LiCl**	**60%**	**30%**
TBACl	51%	25%
TEACl	49%	14%

4.4 Oxidants Screening

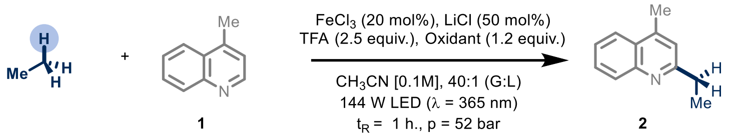

Table S4

Oxidant	Yield of **2**	Yield of **1**
**NFSI**	**60%**	**30%**
$K_2S_2O_8$	-[a]	-[a]
$Na_2S_2O_8$	-[a]	-[a]
Selectfluor	nd	nd

[a] Not amenable to flow. Insoluble oxidant.

4.5 TFA Equivalents Screening

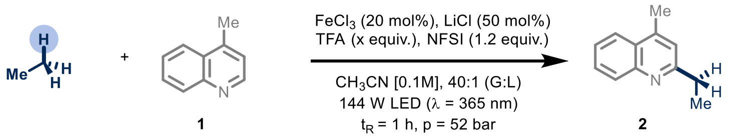

Table S5

TFA equiv.	Yield of **2**	Yield of **1**
2	47%	9%
2.5	60%	30%
**3.5**	**65%**	**20%**
5	42%	28%

4.6 Light Intensity Screening for Electron Poor Substrates

During our optimization for substrates bearing electron poor substituents, we observed that light intensity plays a key role in the reaction outcome.

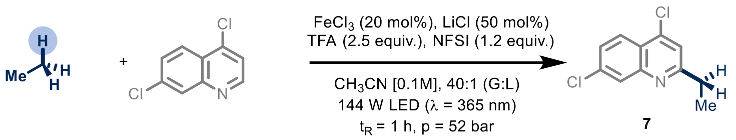

Table S6

Light Intensity	Yield of **2**	Yield of **1**
144 W	Messy crude	n.d.
72 W	42%	5%
**28.8 W**	**63%**	**8%**

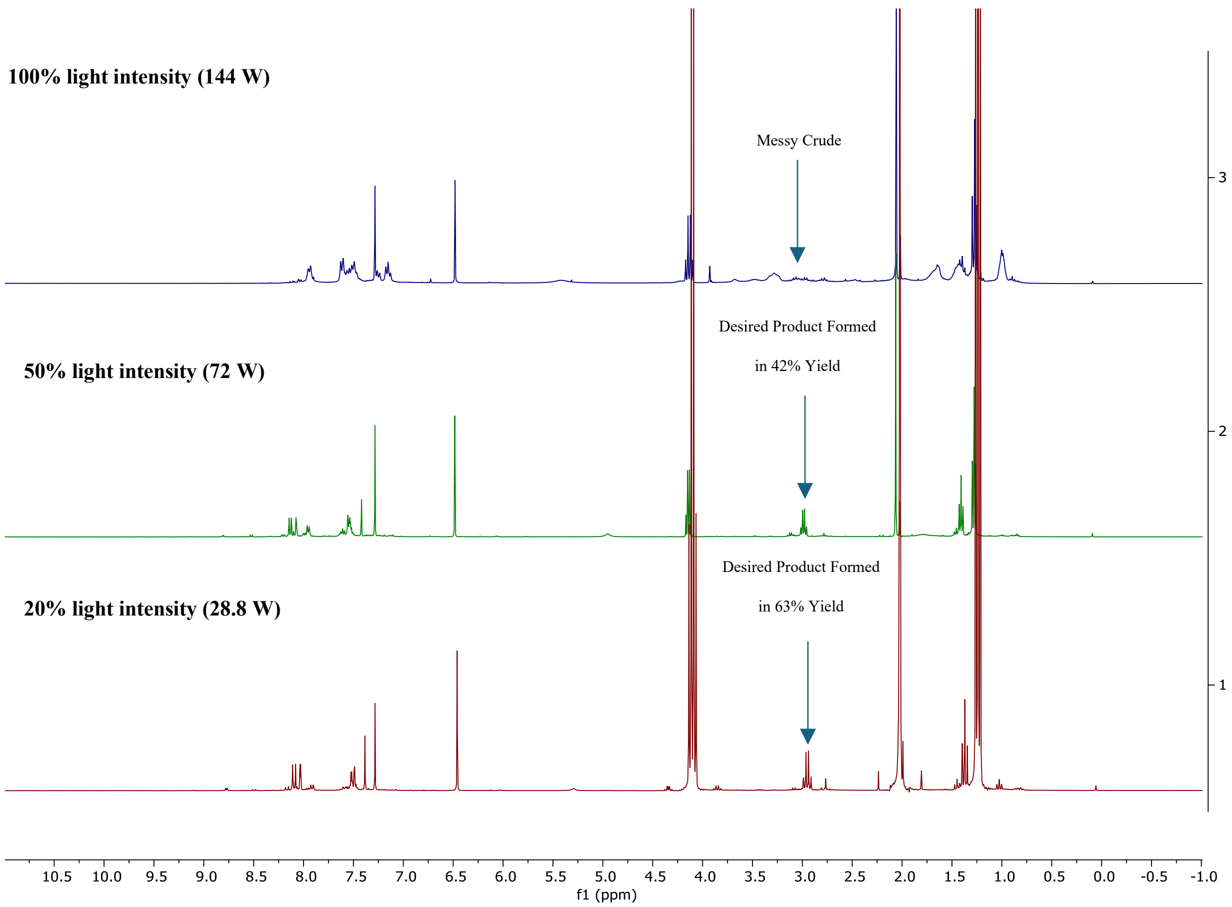

Figure S6: ^{1}H NMR of the reaction mixture with different light intensity.

4.7 Optimization for Methane

The methylation of phenanthridine using methane was chosen as model reaction and an initial screening of reaction parameters was performed in the Signify Eagle Reactor (365 nm, 144 W output power) as described above.

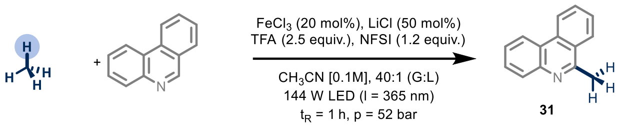

Table S7

Deviation from standard Conditions	Yield of **31**	Yield of **30**
none	33%	16%
40 mol% $FeCl_3$	25%	11%
Gas: Liquid (60:1)	19%	10%
CD_3CN insted of CH_3CN	28%	18%
1.5 h residense time	28%	8%
CH_3CN: TFA (3:1) with 0.5 h residense time	35%	22%
**$FeCl_2$ (20 mol%) as a photocatalysts with CH_3CN: TFA (3:1) and 0.5 h residense time**	**43%**	**8%**

4.8 Optimization for Scale-up

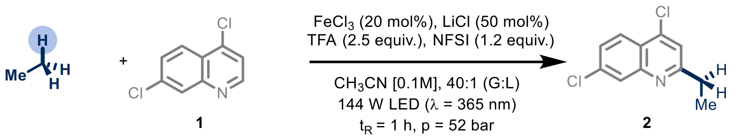

Table S8

Deviation from standard Conditions	Yield of **2**	Yield of **1**
none	63%	8%
CH_3CN: Water (9:1) as solvent	48%	20%
CH_3CN: Water (3:1) as solvent	30%	n.d.
**0.2 M solution with 20: 1 (G: L)**	**52%**	**18%**

4.9 Optimization of the PIFA mediated HAT Minisci reaction

The alkylation of Lepidine (**1**) using propane was chosen as model reaction and a screening of reaction parameters was performed in the Signify Eagle Reactor (456 nm, 184 W output power). The aim of the screening was to obtain new reaction conditions that would afford the desired alkylated heteroarene with high branched selectivity.

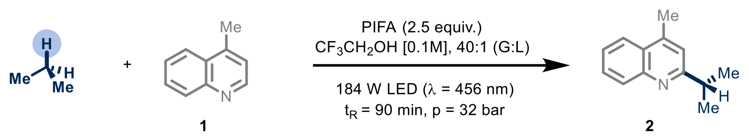

Table S9

Deviation from standard Conditions	Yield of **2**	Yield of **1**
**none**	**58%**	**30%**
120 min residence time	48%	20%
60 min residence time	30%	n.d.
3.5 equiv. of PIFA	60%	28%
TFA (2.5 equiv.) as an additive	59%	32%

5. UV-Vis Characterization

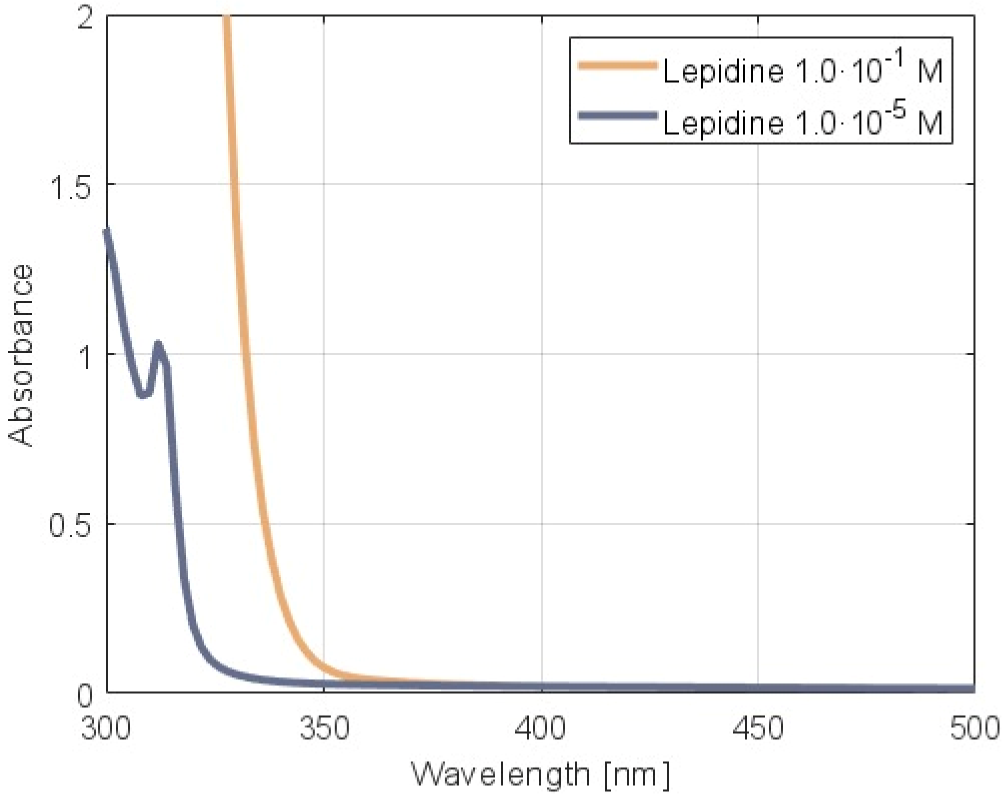

Figure S7: Absorption spectra of prepared Lepidine. The lepidine solution was independently prepared in acetonitrile (0.1 M). The spectra were recorded in acetonitrile in quartz cuvettes (optical path: 1 cm) with a bandwidth of 5 nm and a data pitch of 1 nm. Scan rate: medium.

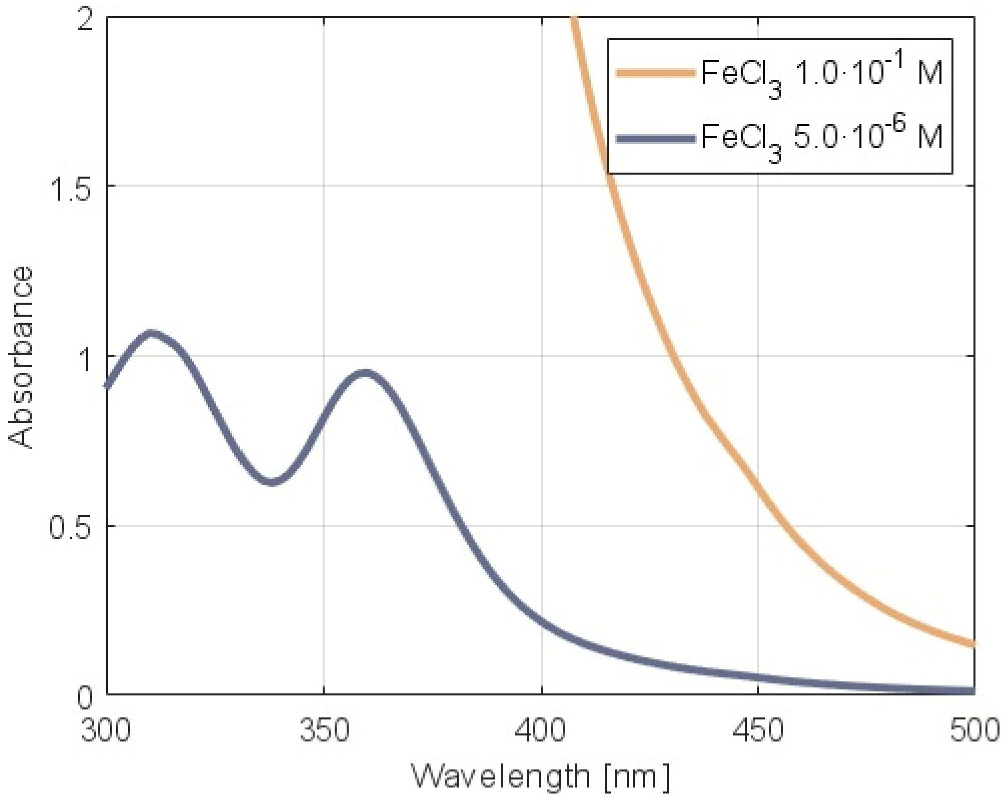

Figure S8: Absorption spectra of prepared $FeCl_3$ solution. The $FeCl_3$ solution was independently prepared in acetonitrile (0.1 M). The spectra were recorded in acetonitrile in quartz cuvettes (optical path: 1 cm) with a bandwidth of 5 nm and a data pitch of 1 nm. Scan rate: medium.

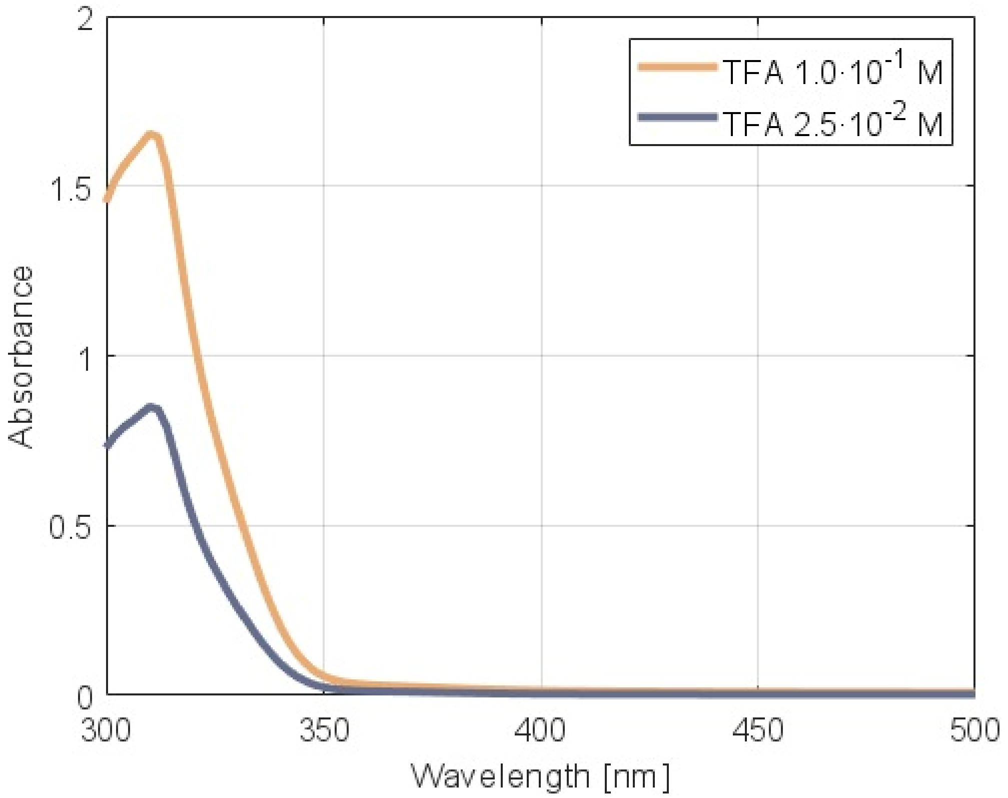

Figure S9: Absorption spectra of prepared TFA solution. The TFA solution was independently prepared in acetonitrile (0.1 M). The spectra were recorded in acetonitrile in quartz cuvettes (optical path: 1 cm) with a bandwidth of 5 nm and a data pitch of 1 nm. Scan rate: medium.

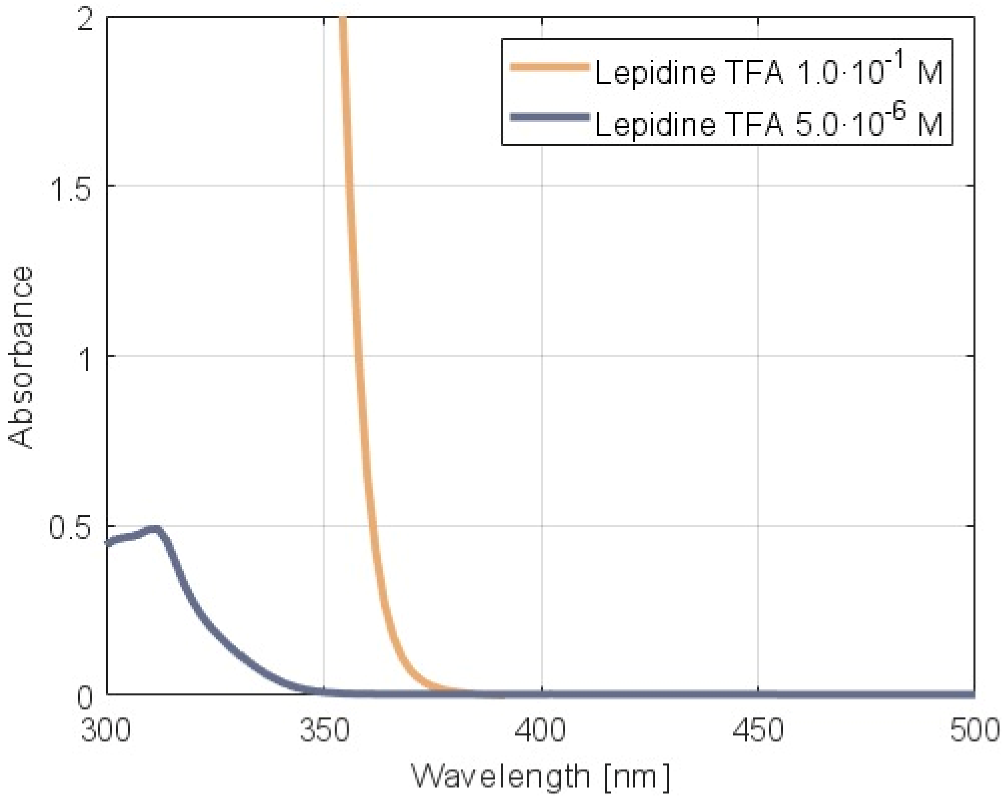

Figure S10: Absorption spectra of prepared lepidine TFA intermediate. The Lepidine TFA intermediate was independently prepared by reacting them in acetonitrile (0.1 M). The spectra were recorded in acetonitrile in quartz cuvettes (optical path: 1 cm) with a bandwidth of 5 nm and a data pitch of 1 nm. Scan rate: medium.

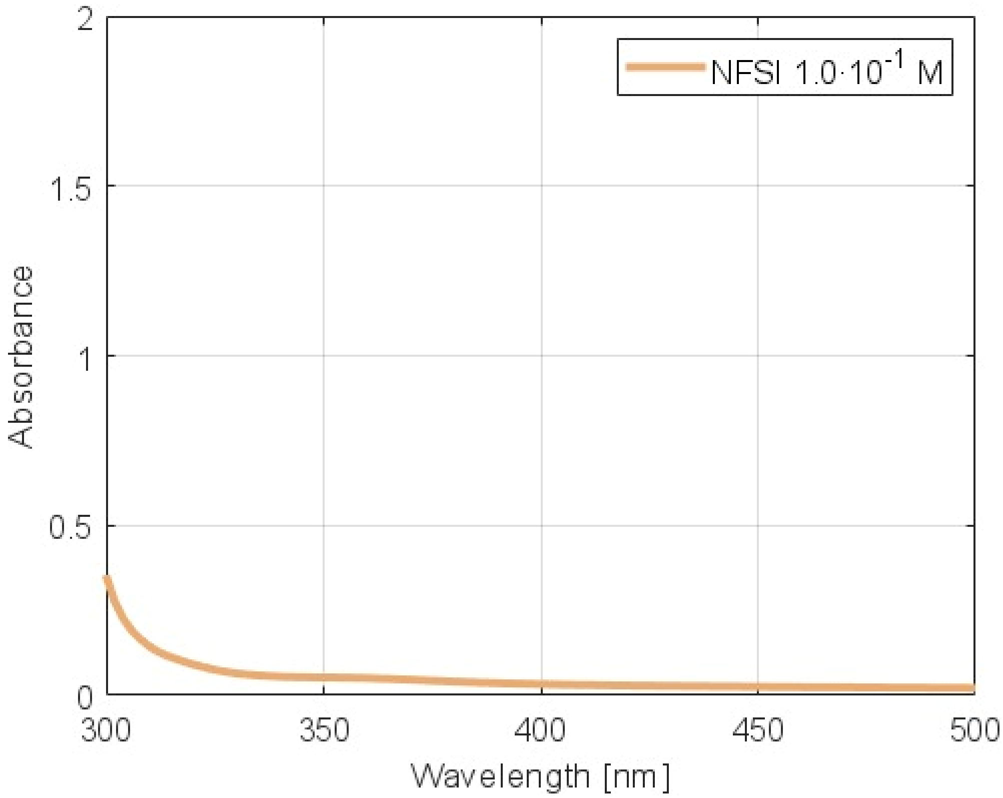

Figure S11: Absorption spectra of prepared NFSI solution. The NFSI solution was independently prepared in acetonitrile (0.1 M). The spectra were recorded in acetonitrile in quartz cuvettes (optical path: 1 cm) with a bandwidth of 5 nm and a data pitch of 1 nm. Scan rate: medium.

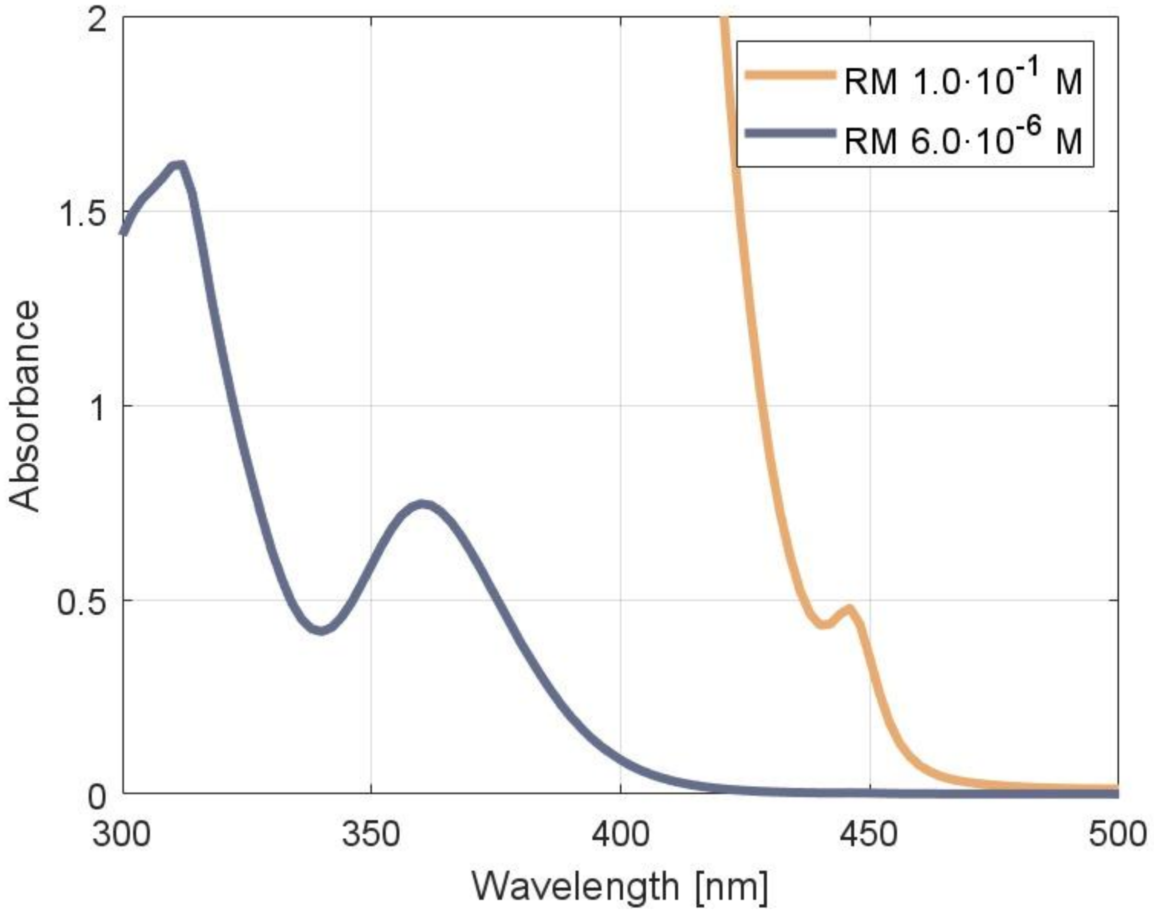

Figure S12: Absorption spectra of prepared Reaction. The reaction mixture was prepared Prepared according to GP1 from lepidine (0.1 mmol, 1 equiv.). The spectra were recorded in acetonitrile in quartz cuvettes (optical path: 1 cm) with a bandwidth of 5 nm and a data pitch of 1 nm. Scan rate: medium.

6. General Procedures (GP)

6.1 (GP1) Ethane

To a nitrogen-purged, screw-capped vial, fitted with a rubber septum and charged with $FeCl_3$ (7 mg, 40 µmol, 20 mol%), LiCl (5 mg, 100 µmol, 50 mol%), NFSI (76 mg, 0.24 mmol, 1.2 equiv.), TFA (27 µL, 0.7 mmol, 3.5 equiv.), the heteroaryl (0.2 mmol, 1 equiv.) was solubilized in 2 mL CH_3CN (0.1 M).
The stock solution is charged in a gastight syringe, positioned in a syringe pump and combined with a stream of ethane gas (80 mL, 4.6 mmol, 23 equiv.) through a T-mixer into filling loop A, with a liquid flow rate of 0.1 mL·min^{-1} and a ethane gas flow rate of 4 mL·min^{-1} (40 : 1 = gas : liquid).
A BPR of 2.8 bar is used during the loop filling.
Next, the filling loop is connected to the reactor, the system is pressurized to 52 bar using an HPLC pump and the reaction mixture is pumped over the Signify Eagle reactor (365 nm, 144 W output power, FEP capillary: 0.5 mm ID, 2.5 mL) at a flow rate of 0.042 mL·min^{-1}, resulting in a residence time of 1 h. The obtained reaction mixture is collected into vial.Then, the reaction mixture is transferred to a separatory funnel, diluted with water (20 mL), neutralized with $NaHCO_3$ and extracted with EtOAc (3x20 mL). The combined organic layers are dried over Na_2SO_4 and evaporated in vacuo. The residue is purified by column chromatography (*n*-pentane:AcOEt).

Modifications of the general procedure were used for certain substrate classes:

Modification 1: 2.5 equiv. of TFA were used, 29 W (20% light intensity) of 365-nm LEDs were used.

Modification 2: 2.5 equiv. of TFA were used, 30 min. residence time and 14 W (10% light intensity) of 365-nm LEDs were used.

Modification 3: 2.5 equiv. of TFA were used, 30 min. residence time.

6.2 (GP2) Propane

To a nitrogen-purged, screw-capped vial, fitted with a rubber septum and charged with $FeCl_3$ (7 mg, 40 µmol, 20 mol%), LiCl (5 mg, 100 µmol, 50 mol%), NFSI (76 mg, 0.24 mmol, 1.2 equiv.), TFA (27 µL, 0.7 mmol, 3.5 equiv.), the heteroaryl (0.2 mmol, 1 equiv.) was solubilized in 2 mL CH_3CN (0.1 M).
The stock solution is charged in a gastight syringe, positioned in a syringe pump and combined with a stream of propane gas (80 ml_n, 3.2 mmol, 16 equiv.) through a T-mixer into filling loop A, with a liquid flow rate of 0.1 mL·min^{-1} and a propane gas flow rate of 4 mL·min^{-1} (40 : 1 = gas : liquid).
A BPR of 2.8 bar is used during the loop filling.
Next, the filling loop is connected to the reactor, the system is pressurized to 52 bar using an HPLC pump and the reaction mixture is pumped over the Signify Eagle reactor (365 nm, 144 W output power, FEP capillary: 0.5 mm ID, 2.5 mL) at a flow rate of 0.042 mL·min-1, resulting in a residence time of 1 h. The obtained reaction mixture is collected into vial. Then, the reaction mixture is transferred to a separatory funnel, diluted with water (20 mL), neutralized with $NaHCO_3$ and extracted with EtOAc (3x20 mL). The combined organic layers are dried over Na_2SO_4 and evaporated in vacuo. The residue is purified by column chromatography (*n*-pentane:AcOEt).

Modifications of the general procedure were used for certain substrate classes:

Modification 1: 2.5 equiv. of TFA were used, 29 W (20% light intensity) of 365-nm LEDs were used.

6.3 (GP3) Butane

To a nitrogen-purged, screw-capped vial, fitted with a rubber septum and charged with $FeCl_3$ (7 mg, 40 µmol, 20 mol%), LiCl (5 mg, 100 µmol, 50 mol%), NFSI (76 mg, 0.24 mmol, 1.2 equiv.), TFA (27 µL, 0.7 mmol, 3.5 equiv.), the heteroaryl (0.2 mmol, 1 equiv.) was solubilized in 2 mL CH_3CN (0.1 M).
The stock solution is charged in a gastight syringe, positioned in a syringe pump and combined with a stream of butane gas (80 mL, 2.4 mmol, 12 equiv.) through a T-mixer into filling loop A, with a liquid flow rate of 0.1 $mL·min^{-1}$ and a butane gas flow rate of 4 $mL·min^{-1}$ (40 : 1 = gas : liquid).
N.B. The BPR was not used during the loop filling.
Next, the filling loop is connected to the reactor, the system is pressurized to 52 bar using an HPLC pump and the reaction mixture is pumped over the Signify Eagle reactor (365 nm, 144 W output power, FEP capillary: 0.5 mm ID, 2.5 mL) at a flow rate of 0.042 mL·min-1, resulting in a residence time of 1 h. The obtained reaction mixture is collected into vial. Then, the reaction mixture is transferred to a separatory funnel, diluted with water (20 mL), neutralized with $NaHCO_3$ and extracted with EtOAc (3x20 mL). The combined organic layers are dried over Na_2SO_4 and evaporated in vacuo. The residue is purified by column chromatography (*n*-pentane:AcOEt).

Modifications of the general procedure were used for certain substrate classes:

Modification 1: 2.5 equiv. of TFA were used, 29 W (20% light intensity) of 365-nm LEDs were used.

6.4 (GP4) Methane

To a nitrogen-purged, screw-capped vial, fitted with a rubber septum and charged with $FeCl_2$ (8 mg, 60 µmol, 20 mol%), LiCl (6 mg, 150 µmol, 50 mol%), NFSI (114 mg, 0.36 mmol, 1.2 equiv.), the heteroaryl (0.3 mmol, 1 equiv.) was solubilized in 3 mL of CH_3CN: TFA (3:1).
The stock solution is charged in a gastight syringe, positioned in a syringe pump and combined with a stream of methane gas (120 mL, 7.0 mmol, 35 equiv.) through a T-mixer into filling loop A, with a liquid flow rate of 0.1 $mL·min^{-1}$ and a methane gas flow rate of 4 $mL·min^{-1}$ (40 : 1 = gas : liquid).
A BPR of 2.8 bar is used during the loop filling.
Next, the filling loop is connected to the reactor, the system is pressurized to 52 bar using an HPLC pump and the reaction mixture is pumped over the Signify Eagle reactor (365 nm, 144 W output power, FEP capillary: 0.5 mm ID, 2.5 mL) at a flow rate of 0.084 mL·min-1, resulting in a residence time of 30 min. The obtained reaction mixture is collected into vial. Then, the reaction mixture is transferred to a separatory funnel, diluted with water (20 mL), neutralized with $NaHCO_3$ and extracted with EtOAc (3x20 mL). The combined organic layers are dried over Na_2SO_4 and evaporated in vacuo. The residue is purified by column chromatography (*n*-pentane:AcOEt).

6.5 Scale-up (Ethane)

To a nitrogen-purged round bottom flaks, fitted with a rubber septum and charged with $FeCl_3$ (160 mg, 1.0 mmol, 20 mol%), LiCl (110 mg, 2.6 mmol, 50 mol%), NFSI (1.9 g, 6.1 mmol, 1.2 equiv.), TFA (980 µL, 12.8 mmol, 2.5 equiv.), 4,7-dichloroquinoline (5.1 mmol, 1 equiv.) was solubilized in 25.5 mL CH_3CN (0.2 M).
The stock solution is charged in a gastight syringe, positioned in a syringe pump and combined with a stream of ethane gas (1000 mL, 28.75 mmol, 11.5 equiv.) through a T-mixer into filling loop A, with a liquid flow rate of 0.1 $mL·min^{-1}$ and a ethane gas flow rate of 4 $mL·min^{-1}$ (20 : 1 = gas : liquid).
A BPR of 2.8 bar is used during the loop filling.
Next, the filling loop is connected to the reactor, the system is pressurized to 32 bar using an HPLC pump and the reaction mixture is pumped over the Signify Eagle reactor (365 nm, 144 W output power, FEP capillary: 0.5 mm ID, 14 mL) at a flow rate of 0.23 $mL·min^{-1}$, resulting in a residence time of 1 h. The obtained reaction mixture is collected into vial.Then, the reaction mixture is transferred to a separatory funnel, diluted with water (60 mL), neutralized with $NaHCO_3$ and extracted with EtOAc (3x60 mL). The combined organic layers are dried over Na_2SO_4 and evaporated in vacuo. The residue is purified by column chromatography (from Pentane to Pentane:Ethyl acetate 20:1) to afford the product as a white solid to afford **7** as a yellowish liquid (588 mg, 46% yield).

6.6 Scale-up (Methane)

To a nitrogen-purged round bottom flaks, fitted with a rubber septum and charged with $FeCl_2$ (48 mg, 0.36 mmol, 20 mol%), LiCl (36 mg, 0.9 mmol, 50 mol%), NFSI (684 mg, 2.16 mmol, 1.2 equiv.), acridine (1.8 mmol, 1 equiv.) was solubilized in 18 mL CH_3CN: TFA (3:1) (0.1 M).
The stock solution is charged in a gastight syringe, positioned in a syringe pump and combined with a stream of methane gas (720 mL, 42 mmol, 35 equiv.) through a T-mixer into filling loop A, with a liquid flow rate of 0.1 $mL \cdot min^{-1}$ and a methane gas flow rate of 4 $mL \cdot min^{-1}$ (40: 1 = gas : liquid).
A BPR of 2.8 bar is used during the loop filling.
Next, the filling loop is connected to the reactor, the system is pressurized to 52 bar using an HPLC pump and the reaction mixture is pumped over the Signify Eagle reactor (365 nm, 144 W output power, FEP capillary: 0.5 mm ID, 2.8 mL) at a flow rate of 0.094 $mL \cdot min^{-1}$, resulting in a residence time of 30 min. The obtained reaction mixture is collected into vial. Then, the reaction mixture is transferred to a separatory funnel, diluted with water (60 mL), neutralized with $NaHCO_3$ and extracted with EtOAc (3x60 mL). The combined organic layers are dried over Na_2SO_4 and evaporated in vacuo. The residue is purified by column chromatography (from Pentane to Pentane:Ethyl acetate 20:1) to afford the product **31** as a brown solid (157 mg, 45% yield).

6.7 (GP5) Propane - PIFA

To a nitrogen-purged, screw-capped vial, fitted with a rubber septum and charged with PIFA (210 mg, 0.5 mmol, 2.5 equiv.), the heteroaryl (0.2 mmol, 1 equiv.) was solubilized in 2 mL CH_3CH_2OH (0.1 M).
The stock solution is charged in a gastight syringe, positioned in a syringe pump and combined with a stream of propane gas (80 ml_n, 3.2 mmol, 16 equiv.) through a T-mixer into filling loop A, with a liquid flow rate of 0.1 $mL \cdot min^{-1}$ and a propane gas flow rate of 4 $mL \cdot min^{-1}$ (40 : 1 = gas : liquid).
A BPR of 2.8 bar is used during the loop filling.
Next, the filling loop is connected to the reactor, the system is pressurized to 32 bar using an HPLC pump and the reaction mixture is pumped over the Signify Eagle reactor (456 nm, 184 W output power, FEP capillary: 0.5 mm ID, 2.5 mL) at a flow rate of 0.028 mL·min-1, resulting in a residence time of 1.5 h. The obtained reaction mixture is collected into vial. Then, the reaction mixture is transferred to a separatory funnel, diluted with water (20 mL), neutralized with $NaHCO_3$ and extracted with DCM (3x20 mL). The combined organic layers are dried over Na_2SO_4 and evaporated in vacuo. The residue was is subjected to GC-MS analysis to get the selectivity of crude product and purified by column chromatography (*n*-pentane:AcOEt).

Modifications of the general procedure were used for certain substrate classes:

Modification 1: 30 min of residence time.

6.8 (GP6) Butane - PIFA

To a nitrogen-purged, screw-capped vial, fitted with a rubber septum and charged with PIFA (210 mg, 0.5 mmol, 2.5 equiv.), TFA (38 µL 0.5 mmol, 2.5 equiv.), the heteroaryl (0.2 mmol, 1 equiv.) was solubilized in 2 mL CH_3CH_2OH (0.1 M).
The stock solution is charged in a gastight syringe, positioned in a syringe pump and combined with a stream of propane gas (80 ml_n, 3.2 mmol, 16 equiv.) through a T-mixer into filling loop A, with a liquid flow rate of 0.1 $mL \cdot min^{-1}$ and a propane gas flow rate of 4 $mL \cdot min^{-1}$ (40 : 1 = gas : liquid).
N.B. The BPR was not used during the loop filling.
Next, the filling loop is connected to the reactor, the system is pressurized to 32 bar using an HPLC pump and the reaction mixture is pumped over the Signify Eagle reactor (456 nm, 184 W output power, FEP capillary: 0.5 mm ID, 2.5 mL) at a flow rate of 0.028 mL·min-1, resulting in a residence time of 1.5 h. The obtained reaction mixture is collected into vial. Then, the reaction mixture is transferred to a separatory funnel, diluted with water (20 mL), neutralized with $NaHCO_3$ and extracted with DCM (3x20 mL). The combined organic layers are dried over Na_2SO_4 and evaporated in vacuo. The residue was is subjected to GC-MS analysis to get the selectivity of crude product and purified by column chromatography (*n*-pentane:AcOEt).

Modifications of the general procedure were used for certain substrate classes:
Modification 1: 30 min of residence time without TFA.

7. Characterization data of synthesized compound

Ethane:

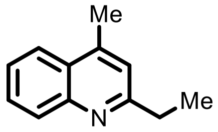

2-ethyl-4-methylquinoline **(2).** Prepared according to GP1 from lepidine (28.6 mg, 0.2 mmol, 1.0 equiv.). Purified via flash column chromatography on silica gel (from Pentane to Pentane:Ethyl acetate 50:1) to afford the product as a yellow oil (20 mg, 58% yield).

Characterization data are in accordance with literature.[3]

^{1}H NMR (400 MHz, $CDCl_3$) δ 7.97 (d, J = 8.4 Hz, 1H), 7.87 (d, J = 8.4 Hz, 1H), 7.65 – 7.54 (m, 1H), 7.51 – 7.37 (m, 1H), 7.08 (s, 1H), 2.89 (q, J = 7.5 Hz, 2H), 2.61 (s, 3H), 1.31 (t, J = 7.6, 1.0 Hz, 3H).
^{13}C NMR (101 MHz, $CDCl_3$) δ 163.8, 147.8, 144.5, 129.4, 129.2, 126.9, 125.6, 123.7, 121.7, 32.3, 18.8, 14.2.
HRMS (ESI) m/z calcd for $C_{12}H_{13}N$ +: [M]+ 171.1048; found: 171.1051.

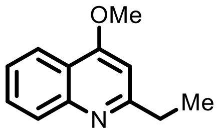

2-ethyl-4-methoxyquinoline **(3).** Prepared according to GP1 from 4-methoxyquinoline (31.8 mg, 0.2 mmol, 1.0 equiv.). Purified via flash column chromatography on silica gel (from Pentane to Pentane:Ethyl acetate 20:1) to afford the product as a white solid (13 mg, 34% yield).

^{1}H NMR (400 MHz, $CDCl_3$) δ 8.14 (d, J = 8.3 Hz, 1H), 8.00 (d, J = 8.5 Hz, 1H), 7.66 (t, J = 7.8 Hz, 1H), 7.54 – 7.38 (t, J = 7.8 Hz 1H), 6.65 (s, 1H), 4.05 (s, 3H), 2.97 (q, J = 7.8 Hz, 2H), 1.40 (t, J = 7.7 Hz, 3H).
^{13}C NMR (101 MHz, $CDCl_3$) δ 165.4, 162.8, 148.6, 130.0, 128.1, 125.0, 121.7, 120.1, 99.4, 55.7, 32.9, 14.3.
HRMS (ESI) m/z calcd for $C_{12}H_{13}NO$ + : [M]+ 187.0997; found: 187.0994.

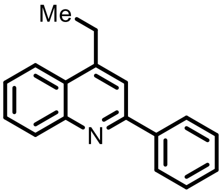

4-ethyl-2-phenylquinoline **(4).** Prepared according to GP1 from 2-phenylquinoline (41 mg, 0.2 mmol, 1.0 equiv.). Purified via flash column chromatography on silica gel (from Pentane to Pentane:Ethyl acetate 100:1) to afford the product as a white solid (26 mg, 56% yield).

Characterization data are in accordance with literature.[4]

^{1}H NMR (400 MHz, $CDCl_3$) δ 8.23 (d, J = 8.4 Hz, 1H), 8.16 (d, J = 7.4 Hz, 2H), 8.05 (d, J = 8.4 Hz, 1H), 7.75 – 7.67 (m, 2H), 7.54 (m, 3H), 7.50 – 7.44 (m, 1H), 3.19 (q, J = 7.5 Hz, 2H), 1.46 (t, J = 7.5 Hz, 3H).
^{13}C NMR (101 MHz, $CDCl_3$) δ 157.4, 150.9, 148.3, 139.9, 130.5, 129.4, 129.4, 128.9, 127.8, 126.5, 126.2, 123.4, 117.0, 25.6, 14.4.
HRMS (ESI) m/z calcd for $C_{17}H_{15}N$ + : [M]+ 233.1204; found: 233.1201.

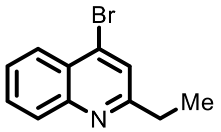

***4-bromo-2-ethylquinoline* (5).** Prepared according to GP1, Modification 1 from 4-bromoquinoline (41.6 mg, 0.2 mmol, 1.0 equiv.). Purified via flash column chromatography on silica gel (from Pentane to Pentane:Ethyl acetate 100:1) to afford the product as a brown solid (28 mg, 60% yield).

Characterization data are in accordance with literature.[5]

^{1}H NMR (400 MHz, $CDCl_3$) δ 8.14 (d, *J* = 8.4 Hz, 1H), 8.03 (d, *J* = 8.5 Hz, 1H), 7.72 (t, *J* = 7.7 Hz, 1H), 7.62 (s, 1H), 7.57 (t, *J* = 7.6 Hz, 1H), 2.98 (q, *J* = 7.5 Hz, 2H), 1.40 (t, *J* = 7.5 Hz, 3H).
^{13}C NMR (101 MHz, $CDCl_3$) δ 164.0, 148.7, 134.3, 130.4, 129.4, 127.0, 126.7, 126.4, 124.9, 32.1, 13.9.
HRMS (ESI) m/z calcd for $C_{11}H_{10}BrN$ + : [M]+ 234.9997; found: 234.9991.

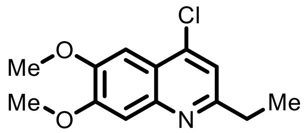

***4-chloro-2-ethyl-6,7-dimethoxyquinoline* (6).** Prepared according to GP1, Modification 1 from 4-chloro-6,7-dimethoxyquinoline (44.6 mg, 0.2 mmol, 1.0 equiv.). Purified via flash column chromatography on silica gel (from Pentane to Pentane:Ethyl acetate 50:1) to afford the product as a white solid (12.6 mg, 25% yield).

^{1}H NMR (400 MHz, $CDCl_3$) δ 7.41 (s, 1H), 7.36 (s, 1H), 7.27 (s, 1H), 4.04 (s, 3H), 4.02 (s, 3H), 2.93(q, *J* = 7.6 Hz, 2H), 1.37 (t, *J* = 7.6 Hz, 3H).
^{13}C NMR (101 MHz, $CDCl_3$) δ 161.7, 153.2, 150.2, 145.5, 141.0, 120.3, 119.1, 107.8, 101.8, 56.4, 56.3, 31.8, 14.1.
HRMS (ESI) m/z calcd for $C_{13}H_{14}ClNO_2$ + : [M]+ 251.0713; found: 251.0719.

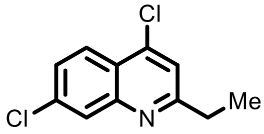

***4,7-dichloro-2-ethylquinoline* (7).** Prepared according to GP1, Modification 1 from 4,7-dichloroquinoline (39.6 mg, 0.2 mmol, 1.0 equiv.). Purified via flash column chromatography on silica gel (from Pentane to Pentane:Ethyl acetate 50:1) to afford the product as a yellow oil (25 mg, 55% yield).

^{1}H NMR (400 MHz, $CDCl_3$) δ 8.11 (d, *J* = 9.0 Hz, 1H), 8.05 (d, *J* = 2.1 Hz, 1H), 7.52 (dd, *J* = 8.9, 2.1 Hz, 1H), 7.40 (s, 1H), 2.97 (q, *J* = 7.6 Hz, 2H), 1.39 (t, *J* = 7.6 Hz, 3H).
^{13}C NMR (101 MHz, $CDCl_3$) δ 165.4, 149.3, 142.7, 136.5, 128.4, 127.8, 125.5, 123.6, 121.3, 32.2, 13.7.
HRMS (ESI) m/z calcd for $C_{11}H_9Cl_2N$ + : [M]+ 225.0112; found: 225.0117.

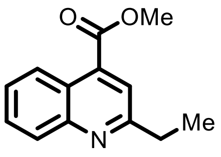

methyl 2-ethylquinoline-4-carboxylate **(8).** Prepared according to GP1, Modification 2 from methyl quinoline-4-carboxylate (37.4 mg, 0.2 mmol, 1.0 equiv.). Purified via flash column chromatography on silica gel (from Pentane to Pentane:Ethyl acetate 50:1) to afford the product as a yellow oil (24 mg, 55% yield).

^{1}H NMR (400 MHz, $CDCl_3$) δ 8.68 (dd, *J* = 8.6, 1.5 Hz, 1H), 8.09 (dd, *J* = 8.5, 1.4 Hz, 1H), 7.81 (s, 1H), 7.76 – 7.68 (m, 1H), 7.61 – 7.53 (m, 1H), 4.03 (s, 3H), 3.04 (q, *J* = 7.6 Hz, 2H), 1.41 (t, *J* = 7.6 Hz, 3H).
^{13}C NMR (101 MHz, $CDCl_3$) δ 167.0, 163.5, 149.0, 135.3, 129.7, 129.5, 127.3, 125.5, 123.6, 122.3, 52.7, 32.3, 13.9.
HRMS (ESI) m/z calcd for $C_{13}H_{13}NO_2$ + : [M]+ 215.0946; found: 215.0951.

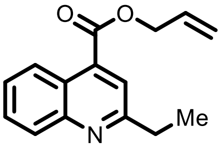

allyl 2-ethylquinoline-4-carboxylate **(9).** Prepared according to GP1, Modification 2 from allyl quinoline-4-carboxylate (42.6 mg, 0.2 mmol, 1.0 equiv.). Purified via flash column chromatography on silica gel (from Pentane to Pentane:Ethyl acetate 50:1) to afford the product as a yellow oil (31 mg, 64% yield).

^{1}H NMR (400 MHz, $CDCl_3$) δ 8.69 (d, *J* = 8.2 Hz, 1H), 8.10 (d, *J* = 8.1 Hz, 1H), 7.84 (s, 1H), 7.73 (t, *J* = 7.5 Hz, 1H), 7.61 – 7.57 (m, 1H), 6.12 (tdd, *J* = 16.9, 6.9, 4.0 Hz, 1H), 5.48 (d, *J* = 17.2 Hz, 1H), 5.37 (d, *J* = 10.0 Hz, 1H), 4.95 (d, *J* = 6.1 Hz, 2H), 3.06 (q, *J* = 8.3 Hz, 2H), 1.43 (t, *J* = 8.0 Hz, 3H).
^{13}C NMR (101 MHz, $CDCl_3$) δ 166.3, 163.6, 149.0, 135.4, 131.8, 129.8, 129.6, 127.3, 125.5, 123.7, 122.3, 119.4, 66.5, 32.3, 13.9.
HRMS (ESI) m/z calcd for $C_{15}H_{15}NO_2$ + : [M]+ 241.1103; found: 241.1109.

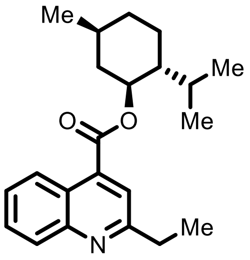

(1S,2R,5S)-2-isopropyl-5-methylcyclohexyl 2-ethylquinoline-4-carboxylate **(10).** Prepared according to GP1, Modification 2 from (1S,2R,5S)-2-isopropyl-5-methylcyclohexyl quinoline-4-carboxylate (62.2 mg, 0.2 mmol, 1.0 equiv.). Purified via flash column chromatography on silica gel (from Pentane to Pentane:Ethyl acetate 50:1) to afford the product as a orange oil (34 mg, 50% yield).

^{1}H NMR (400 MHz, $CDCl_3$) δ 8.65 (d, *J* = 8.5 Hz, 1H), 8.10 (d, *J* = 8.5 Hz, 1H), 7.74 (m, 2H), 7.58 (t, *J* = 8.6, 1H), 5.10 (td, *J* = 10.9, 4.4 Hz, 1H), 3.06 (q, *J* = 7.6 Hz, 2H), 2.23 (d, *J* = 11.5 Hz, 1H), 1.99 (m, 1H), 1.77 (d, *J* = 10.1 Hz, 2H), 1.61 (t, *J* = 17.6, 2H), 1.43 (t, *J* = 7.6 Hz, 3H), 1.33 – 1.09 (m, 3H), 0.98 (d, *J* = 6.6 Hz, 3H), 0.94 (d, *J* = 7.0 Hz, 3H), 0.86 (d, *J* = 6.9 Hz, 3H).
^{13}C NMR (101 MHz, $CDCl_3$) δ 166.3, 163.5, 148.9, 136.4, 129.7, 129.5, 127.2, 125.5, 123.8, 121.9, 76.1, 47.3, 41.1, 34.4, 32.3, 31.7, 26.6, 23.6, 22.2, 21.0, 16.5, 13.9.
HRMS (ESI) m/z calcd for $C_{22}H_{29}NO_2$ + : [M]+ 339.2198; found: 339.2196.

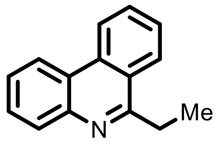

***6-ethylphenanthridine* (11).** Prepared according to GP1 from phenanthridine (35.8 mg, 0.2 mmol, 1.0 equiv.). Purified via flash column chromatography on silica gel (from Pentane to Pentane:Ethyl acetate 20:1) to afford the product as a orange solid (30.3 mg, 73% yield).

Characterization data are in accordance with literature.[4]

^{1}H NMR (300 MHz, $CDCl_3$) δ 8.63 (dd, *J* = 8.3, 1.2 Hz, 1H), 8.53 (dd, *J* = 8.1, 1.4 Hz, 1H), 8.30 – 8.19 (m, 1H), 8.14 (dd, *J* = 8.1, 1.4 Hz, 1H), 7.81 (ddd, *J* = 8.3, 7.0, 1.3 Hz, 1H), 7.69 (ddd, *J* = 9.7, 8.2, 7.0, 1.4 Hz, 2H), 7.61 (ddd, *J* = 8.3, 7.0, 1.4 Hz, 1H), 3.41 (q, *J* = 7.6 Hz, 2H), 1.52 (t, *J* = 7.6 Hz, 3H).
^{13}C NMR (101 MHz, $CDCl_3$) δ 163.3, 143.9, 133.0, 130.4, 129.7, 128.7, 127.3, 126.4, 126.3, 125.1, 123.8, 122.6, 122.0, 29.5, 13.7.
HRMS (ESI) m/z calcd for $C_{15}H_{13}N$ + : [M]+207.1048; found:207.1051.

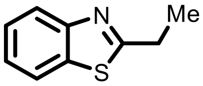

***2-ethylbenzo[d]thiazole* (12) .** Prepared according to GP1 Modification 3 from benzo[d]thiazole (27 mg, 0.2 mmol, 1.0 equiv.). Purified via flash column chromatography on silica gel (from Pentane to Pentane:Ethyl acetate 20:1) to afford the product as a yellow oil (10.6 mg, 32% yield).

Characterization data are in accordance with literature.[4]

^{1}H NMR (400 MHz, $CDCl_3$) δ 7.97 (d, *J* = 8.2 Hz, 1H), 7.84 (d, *J* = 7.9 Hz, 1H), 7.49 – 7.38 (m, 1H), 7.37 – 7.27 (m, 1H), 3.16 (q, *J* = 7.6 Hz, 2H), 1.48 (t, *J* = 7.6 Hz, 3H).
^{13}C NMR (101 MHz, $CDCl_3$) δ 173.7, 153.4, 135.2, 126.0, 124.7, 122.7, 121.6, 27.9, 13.9.
HRMS (ESI) m/z calcd for C_9H_9NS + : [M]+163.0456; found:163.0457.

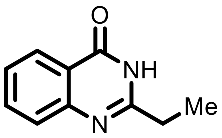

***2-ethylquinazolin-4(3H)-one* (13) .** Prepared according to GP1, Modification 3 and 0.2 ml of H_2O as a co-solvent from quinazolin-4(3H)-one (29.2 mg, 0.2 mmol, 1.0 equiv.). Purified via flash column chromatography on silica gel (Pentane: Ethyl acetate 1:1) to afford the product as a white solid (20 mg, 57% yield).

Characterization data are in accordance with literature.[6]

^{1}H NMR (400 MHz, $CDCl_3$) δ 11.36 (s, 1H), 8.29 (dd, *J* = 8.0, 1.6 Hz, 1H), 7.77 (ddd, *J* = 8.4, 6.9, 1.6 Hz, 1H), 7.71 (dd, *J* = 8.2, 1.3 Hz, 1H), 7.47 (ddd, *J* = 8.2, 7.0, 1.3 Hz, 1H), 2.83 (q, *J* = 7.6 Hz, 2H), 1.45 (t, *J* = 7.6 Hz, 3H).
^{13}C NMR (101 MHz, $CDCl_3$) δ 164.1, 157.5, 149.6, 134.9, 127.4, 126.5, 126.4, 120.7, 29.3, 11.6.
HRMS (ESI) m/z calcd for $C_{10}H_{10}N_20$ + : [M]+173.0793; found:173.0788.

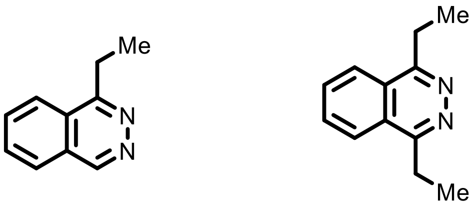

Mono Bis

***1-ethylphthalazine* (14, mono) .** Prepared according to GP1, Modification 1 and 0.2 ml of H_2O as a co-solvent from phthalazine (26.0 mg, 0.2 mmol, 1.0 equiv.). Purified via flash column chromatography on silica gel (Pentane: Ethyl acetate 5:1) to afford the mono alkylated product as a yellow liquid (11 mg, 35% yield) and bis alkylated product as a brown gel (5.6 mg, 15% yield).

Characterization data are in accordance with literature.[4]

^{1}H NMR (400 MHz, $CDCl_3$) ^{1}H NMR (400 MHz, Chloroform-*d*) δ 9.40 (s, 1H), 8.13 (d, *J* = 8.0 Hz, 1H), 8.01 – 7.73 (m, 3H), 3.40 (q, *J* = 7.5 Hz, 2H), 1.51 (t, *J* = 7.5 Hz, 3H).
^{13}C NMR (101 MHz, $CDCl_3$) δ 161.5, 150.6, 132.5, 132.0, 127.2, 126.7, 125.6, 124.1, 26.7, 13.5.
HRMS (ESI) m/z calcd for $C_{10}H_{10}N_2$+ : [M]+158.0844; found:158.0848.

***1,4-diethylphthalazine* (14, bis)**
^{1}H NMR (400 MHz, $CDCl_3$δ 8.12 (dd, *J* = 6.3, 3.3 Hz, 2H), 7.86 (dd, *J* = 6.2, 3.2 Hz, 2H), 3.35 (q, *J* = 7.5 Hz, 4H), 1.49 (t, *J* = 7.5 Hz, 6H).
^{13}C NMR (101 MHz, $CDCl_3$) δ 160.2, 131.7, 125.6, 124.7, 26.5, 13.3.
HRMS (ESI) m/z calcd for $C_{12}H_{14}N_2$+ : [M]+186.1157; found:186.1161.

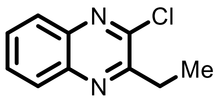

***2-chloro-3-ethylquinoxalin* (15) .** Prepared according to GP1 Modification 3 and 0.2 ml of H_2O as a co-solvent, from 2-chloroquinoxalin (32.9 mg, 0.2 mmol, 1.0 equiv.). Purified via flash column chromatography on silica gel (Pentane: Ethyl acetate 5:1) to afford the product as a black solid (13 mg, 34% yield).

^{1}H NMR (400 MHz, $CDCl_3$) δ 8.05 (d, *J* = 7.7 Hz, 1H), 7.98 (d, *J* = 8.7 Hz, 1H), 7.73 (m, 2H), 3.17 (q, *J* = 6.8 Hz, 2H), 1.44 (t, *J* = 7.4 Hz, 3H).
^{13}C NMR (101 MHz, $CDCl_3$) δ 156.8, 147.7, 141.2, 141.0, 130.1, 130.0, 128.8, 128.2, 29.3, 11.8.
HRMS (ESI) m/z calcd for $C_{10}H_9ClN_2$+ : [M]+192.0454; found:192.0460.

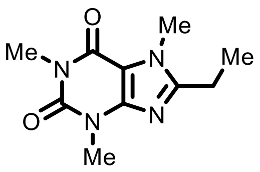

***8-ethyl-1,3,7-trimethyl-3,7-dihydro-1H-purine-2,6-dione* (16).** Prepared according to GP1, Modification 1 from 1,3,7-trimethyl-3,7-dihydro-1H-purine-2,6-dione (38.8 mg, 0.2 mmol, 1.0 equiv.). Purified via flash column chromatography on silica gel (Pentane: Ethyl acetate 5:1) to afford the product as a white solid (16 mg, 36% yield).

^{1}H NMR (400 MHz, $CDCl_3$) δ 3.91 (s, 3H), 3.57 (s, 3H), 3.40 (s, 3H), 2.75 (q, *J* = 6.8 Hz, 2H), 1.34 (t, *J* = 7.4 Hz, 3H).
^{13}C NMR (101 MHz, $CDCl_3$) δ 155.5, 155.4, 151.9, 148.1, 107.5, 31.7, 29.8, 28.0, 20.4, 11.8.
HRMS (ESI) m/z calcd for $C_{10}H_{14}N_4O_2$+ : [M]+222.1117; found: 222.1120.

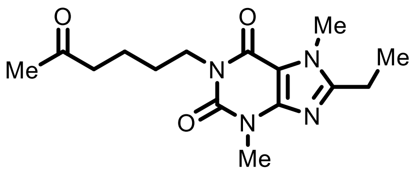

***8-ethyl-3,7-dimethyl-1-(5-oxohexyl)-3,7-dihydro-1H-purine-2,6-dione* (17).** Prepared according to GP1, Modification 1 and 0.2 ml of H_2O is used as a co-solvent from 3,7-dimethyl-1-(5-oxohexyl)-3,7-dihydro-1H-purine-2,6-dione (55.8 mg, 0.2 mmol, 1.0 equiv.). Purified via flash column chromatography on silica gel (Pentane: Ethyl acetate 5:1) to afford the product as a white solid (12.3 mg, 20% yield).

^{1}H NMR (400 MHz, $CDCl_3$) δ 4.00 (t, *J* = 6.8 Hz, 2H), 3.90 (s, 3H), 3.55 (s, 3H), 2.76 (q, *J* = 6.8 Hz, 2H), 2.49 (t, *J* = 6.8 Hz, 2H), 2.14 (s, 3H), 1.65 (m, 4H), 1.43 – 1.28 (t, *J* = 7.4 Hz, 3H).
^{13}C NMR (101 MHz, $CDCl_3$) δ 208.8, 155.3, 155.2, 151.5, 148.1, 107.4, 43.2, 40.7, 31.6, 29.9, 29.6, 27.5, 21.0, 20.3, 11.7.
HRMS (ESI) m/z calcd for $C_{15}H_{22}N_4O_3$+ : [M]+306.1692; found: 306.1698.

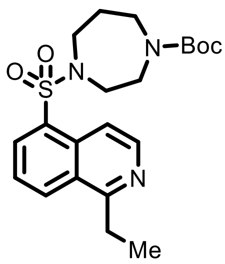

***tert-butyl 4-((1-ethylisoquinolin-5-yl)sulfonyl)-1,4-diazepane-1-carboxylate* (18) .** Prepared according to GP1 with the utilization of modification 1 and 0.4 ml of H_2O is used as a co-solvent from 5-((1,4-diazepan-1-yl)sulfonyl)isoquinoline hydrogen chloride (65.4 mg, 0.2 mmol, 1.0 equiv.). Then, the solvent was removed under reduced pressure.

N.B. For the ease of purification, the crude rection mixture was subsequently dissolved in 5 mL of CH_2Cl_2 and Boc-anhydride (66 mg, 1.5 equiv.) was added portion-wise. Then, triethylamine (39 µL, 3 equiv.) was added, and the mixture was stirred for 2h at rt. Next, the solvent was removed under reduced pressure and the crude was purified via flash column chromatography on silica gel (Pentane: Ethyl acetate 1:1) to afford the product as a yellow gel (29.4 mg, 35% yield).

Characterization data are in accordance with literature.[7]

^{1}H NMR (400 MHz, $CDCl_3$) δ 8.57 (d, *J* = 6.1 Hz, 1H), 8.41 (d, *J* = 8.5 Hz, 1H), 8.35 – 8.22 (m, 2H), 7.64 (t, *J* = 8.0 Hz, 1H), 3.51 (dt, *J* = 12.8, 5.6 Hz, 4H), 3.43 – 3.30 (m, 6H), 1.94 (p, *J* = 6.3 Hz, 2H), 1.43 (m, 12H).
^{13}C NMR (101 MHz, $CDCl_3$) δ 164.2, 155.1 & 154.9 (rotameric signals), 144.1, 135.1 & 135.0 (rotameric signals) 132.7 & 132.4 (rotameric signals), 132.2, 131.0, 127.5, 125.4, 115.8, 80.1 & 80.0 (rotameric signals), 50.2 & 49.9 (rotameric signals), 49.5 & 49.4 (rotameric signals), 47.7 & 47.5 (rotameric signals), 46.1 & 45.5 (rotameric signals), 29.1, 28.7 & 28.3 (rotameric signals), 28.5, 13.7.

HRMS (ESI) m/z calcd for $C_{21}H_{29}N_3O_4S$ + : [M]+419.1879; found: 419.1885

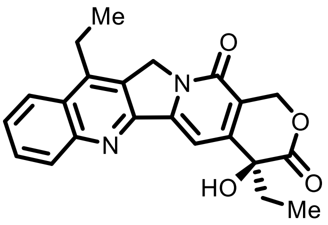

(S)-4,11-diethyl-4-hydroxy-1,12-dihydro-14H-pyrano[3',4':6,7]indolizino[1,2-b]quinoline-3,14(4H)-dione **(19)** **.** Prepared according to GP1 but CH_3CN:TFA (3:1) was used as a solvent from (S)-4-ethyl-4-hydroxy-1,12-dihydro-14H-pyrano[3',4':6,7]indolizino[1,2-b]quinoline-3,14(4H)-dione (69.6 mg, 0.2 mmol, 1.0 equiv.). Purified via flash column chromatography on silica gel (Pentane: Ethyl acetate 1:1) to afford the product as a yellow solid (40 mg, 53% yield).

^{1}H NMR (400 MHz, $CDCl_3$) δ 8.23 (dd, *J* = 8.5, 1.4 Hz, 1H), 8.12 (dd, *J* = 8.5, 1.4 Hz, 1H), 7.80 (ddd, *J* = 8.4, 6.8, 1.4 Hz, 1H), 7.70 – 7.62 (m, 2H), 5.75 (d, *J* = 16.3 Hz, 1H), 5.30 (d, *J* = 16.4 Hz, 1H), 5.25 (s, 2H), 3.86 (s, 1H), 3.21 (q, *J* = 7.7 Hz, 2H), 1.94-1.86 (m, 2H), 1.41 (t, *J* = 7.7 Hz, 3H), 1.04 (t, *J* = 7.4 Hz, 3H).
^{13}C NMR (101 MHz, $CDCl_3$) δ 174.1, 157.8, 152.1, 150.3, 149.6, 147.2, 145.8, 130.9, 130.2, 127.8, 127.1, 126.9, 123.6, 118.6, 98.1, 72.9, 66.5, 49.6, 31.8, 23.2, 14.2, 8.0.
HRMS (ESI) m/z calcd for $C_{22}H_{20}N_2O_4$ + : [M]+376.1423; found: 376.1418.

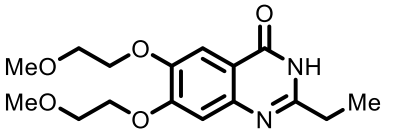

2-ethyl-6,7-bis(2-methoxyethoxy)quinazolin-4(3H)-one **(20) .** Prepared according to GP4, ethane as gaseous alkane and 60 min. residence time, from 6,7-bis(2-methoxyethoxy)quinazolin-4(3H)-one (58.8 mg, 0.2 mmol, 1.0 equiv.). Purified via flash column chromatography on silica gel (Pentane: Ethyl acetate 1:1 to ethyl acetate) to afford the product as a white solid (16 mg, 25% yield).

Characterization data are in accordance with literature.[8]

^{1}H NMR (400 MHz, $CDCl_3$) δ 11.69 (s, 1H), 7.58 (s, 1H), 7.09 (s, 1H), 4.40 – 4.08 (m, 4H), 3.86-3.80 (m, 4H), 3.47 (s, 3H), 3.47 (s, 3H), 2.79 (q, *J* = 7.6 Hz, 2H), 1.41 (t, *J* = 7.6 Hz, 3H).
^{13}C NMR (101 MHz, $CDCl_3$) δ 162.8, 155.4, 148.7, 131.2, 128.3, 126.9, 107.2, 70.8, 70.6, 68.8, 68.7, 59.4, 29.8, 11.8.
One carbon peak not visible
HRMS (ESI) m/z calcd for $C_{16}H_{22}N_2O_5$ + : [M]+322.1529; found: 322.1535.

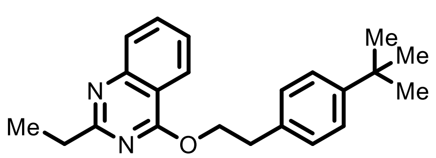

4-(4-(tert-butyl)phenethoxy)-2-ethylquinazoline **(21).** Prepared according to GP1 from 4-(4-(tert-butyl)phenethoxy)-quinazoline (61.2 mg, 0.2 mmol, 1.0 equiv.). Purified via flash column chromatography on silica gel (from Pentane to Pentane:Ethyl acetate 10:1) to afford the product as a yellow solid (14.7 mg, 22% yield).

^{1}H NMR (400 MHz, $CDCl_3$) δ 8.34 – 7.94 (m, 1H), 7.90 – 7.80 (m, 1H), 7.77 (ddd, *J* = 8.4, 6.9, 1.5 Hz, 1H), 7.48 (ddd, *J* = 8.1, 6.9, 1.2 Hz, 1H), 7.40 – 7.33 (m, 2H), 7.28 (d, *J* = 8.3 Hz, 2H), 4.78 (t, *J* = 7.1 Hz, 2H), 3.18 (t, *J* = 7.1 Hz, 2H), 2.96 (q, *J* = 7.6 Hz, 2H), 1.41 (t, *J* = 7.6 Hz, 3H), 1.32 (s, 9H).
^{13}C NMR (101 MHz, $CDCl_3$) δ 168.2, 166.7, 151.5, 149.6, 135.2, 133.4, 128.9, 127.2, 126.1, 125.6, 123.6, 115.0, 67.5, 34.9, 34.6, 33.2, 31.5, 12.9.
HRMS (ESI) m/z calcd for $C_{22}H_{26}N_2O$ +: [M]+ 334.2045; found:334.2051.

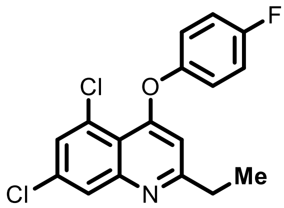

***5,7-dichloro-2-ethyl-4-(4-fluorophenoxy) quinoline* (37).** Prepared according to GP1 from 5,7-dichloro-4-(4-fluorophenoxy) quinoline (61.6 mg, 0.2 mmol, 1.0 equiv.). Purified via flash column chromatography on silica gel (from Pentane to Pentane: Ethyl acetate 10:1) to afford the product as a white solid (27.5 mg, 41% yield).

Characterization data are in accordance with literature.[4]

^{1}H NMR (400 MHz, $CDCl_3$) δ 7.94 (s, 1H), 7.51 (s, 1H), 7.21 – 6.98 (m, 4H), 6.50 (s, 1H), 2.80 (q, *J* = 7.6 Hz, 2H), 1.26 (t, *J* = 7.6 Hz, 3H).
^{13}C NMR (101 MHz, $CDCl_3$) δ 166.8, 162.6, 161.1 (d, *J* = 244.4 Hz), 151.6, 150.3 (d, *J* = 2.8 Hz), 135.0, 130.1, 128.8, 127.5, 122.3 (d, *J* = 8.4 Hz), 117.3, 117.1, 106.6, 32.3, 13.6.
^{19}F NMR (282 MHz, $CDCl_3$) δ -117.16.
HRMS (ESI) m/z calcd for $C_{17}H_{12}Cl_2FNO^+$: [M]+ 335.0280; found:335.0287.

Propane:

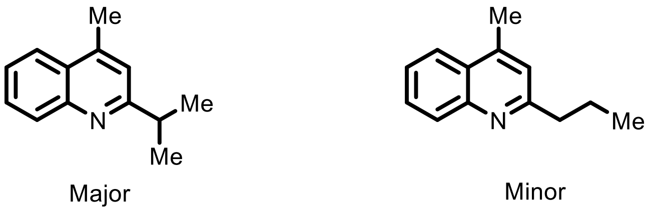

2-isopropyl-4-methylquinoline **(22 major).** Prepared according to GP2 from lepidine (28.6 mg, 0.2 mmol, 1.0 equiv.). Purified via flash column chromatography on silica gel (from Pentane to Pentane:Ethyl acetate 50:1) to afford the major product as a colorless oil (16 mg, 44% yield) and the minor as a colorless oil (8 mg, 21% yield).

Characterization data are in accordance with literature.[9]

^{1}H NMR (400 MHz, $CDCl_3$) δ 8.11 – 8.01 (m, 1H), 7.95 (dd, *J* = 8.3, 1.5 Hz, 1H), 7.67 (ddd, *J* = 8.4, 6.9, 1.4 Hz, 1H), 7.50 (ddd, *J* = 8.2, 6.8, 1.3 Hz, 1H), 7.18 (s, 1H), 3.21 (hept, *J* = 6.9 Hz, 1H), 2.69 (s, 3H), 1.39 (d, *J* = 6.9 Hz, 6H).
^{13}C NMR (101 MHz, $CDCl_3$) δ 167.5, 147.7, 144.5, 129.7, 129.1, 127.2, 125.5, 123.7, 119.9, 37.4, 22.7, 19.0.
HRMS (ESI) m/z calcd for $C_{13}H_{15}N$ +: [M]+ 185.1204; found: 185.1225.

Characterization data are in accordance with literature.[10]

2-propyl-4-methylquinoline **(22 minor).**
^{1}H NMR (400 MHz, $CDCl_3$) δ 8.04 (dd, *J* = 8.5, 1.5 Hz, 1H), 7.95 (dd, *J* = 8.3, 1.6 Hz, 1H), 7.67 (ddd, *J* = 8.4, 6.8, 1.5 Hz, 1H), 7.50 (ddd, *J* = 8.3, 6.8, 1.4 Hz, 1H), 7.14 (s, 1H), 2.92- 2.88 (d, 7.6 Hz, 2H), 2.68 (s, 3H), 1.91 – 1.75 (m, 2H), 1.02 (t, *J* = 7.4 Hz, 3H).
^{13}C NMR (101 MHz, $CDCl_3$) δ 162.7, 147.9, 144.3, 129.5, 129.1, 126.9, 125.5, 123.7, 122.2, 41.4, 23.4, 18.8, 14.2.
HRMS (ESI) m/z calcd for $C_{13}H_{15}N$ +: [M]+ 185.1204; found: 185.1215.

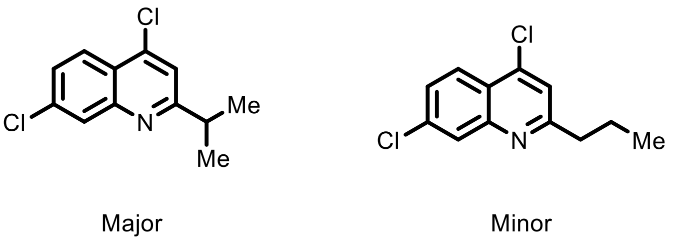

4,7-dichloro-2-isopropylquinoline **(23 major).** Prepared according to GP2, Modification 1 from 4,7-dichloroquinoline (39.6 mg, 0.2 mmol, 1.0 equiv.). Purified via flash column chromatography on silica gel (from Pentane to Pentane:Ethyl acetate 50:1) to afford the major product as a yellow oil (19 mg, 40% yield) and minor product as a yellow oil (11 mg, 21% yield).

^{1}H NMR (400 MHz, $CDCl_3$) δ 8.19 – 8.04 (m, 2H), 7.51 (dd, *J* = 8.9, 2.0 Hz, 1H), 7.41 (s, 1H), 3.21 (hept, *J* = 6.9 Hz, 1H), 1.38 (d, *J* = 6.9 Hz, 6H).
^{13}C NMR δ 169.1, 149.1, 142.8, 136.4, 128.4, 127.8, 125.4, 123.7, 119.9, 37.2, 22.4.
HRMS (ESI) m/z calcd for $C_{12}H_{11}Cl_2N$ + : [M]+ 239.0269; found: 239.0287.

4,7-dichloro-2-propylquinoline **(23 minor).**
^{1}H NMR (400 MHz, $CDCl_3$) δ 8.11 (d, *J* = 8.9 Hz, 1H), 8.06 (d, *J* = 2.1 Hz, 1H), 7.52 (dd, *J* = 8.9, 2.1 Hz, 1H), 7.38 (s, 1H), 2.93 – 2.88 (t, *J*= 7.4 Hz, 2H), 1.95 – 1.74 (m, 2H), 1.02 (t, *J* = 7.4 Hz, 3H).
^{13}C NMR δ 164.3, 149.2, 142.6, 136.5, 128.3, 127.8, 125.5, 123.6, 121.7, 41.1, 23.0, 14.0.
HRMS (ESI) m/z calcd for $C_{12}H_{11}Cl_2N$ + : [M]+ 239.0269; found: 239.0277.

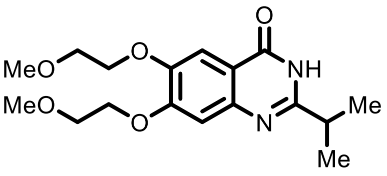

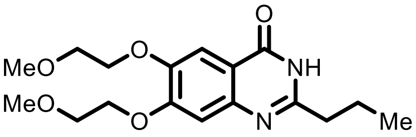

2-isopropyl-6,7-bis(2-methoxyethoxy) quinazolin-4(3H)-one **(24 major)** Prepared according to GP4, propane as gaseous alkane and 60 min. residence time, from 6,7-bis(2-methoxyethoxy)quinazolin-4(3H)-one (58.8 mg, 0.2 mmol, 1.0 equiv.). Purified via flash column chromatography on silica gel (Pentane: Ethyl acetate 1:1 to ethyl acetate) to afford the major product as a yellow solid (20.2 mg, 30% yield) and minor product as a yellow gel (10 mg, 15% yield)

Characterization data are in accordance with literature.[4]

^{1}H NMR (300 MHz, $CDCl_3$) δ 11.12 (s, 1H), 7.60 (s, 1H), 7.10 (s, 1H), 4.27 (ddd, *J* = 5.3, 4.1, 2.0 Hz, 4H), 3.84 (ddd, *J* = 6.4, 4.0, 1.9 Hz, 4H), 3.48 (d, *J* = 1.9 Hz, 6H), 2.99 (hept, *J* = 7.0 Hz, 1H), 1.41 (d, *J* = 6.9 Hz, 6H).
^{13}C NMR δ 163.3, 159.7, 155.1, 148.3, 146.1, 114.1, 109.1, 107.3, 70.9, 70.7, 68.8, 68.5, 59.5, 59.4, 34.8, 20.7.
HRMS (ESI) m/z calcd for $C_{17}H_{24}N_2O_5$ + : [M]+ 336.1685; found: 336.1698.

6,7-bis(2-methoxyethoxy)-2-propylquinazolin-4(3H)-one **(24 minor).**
^{1}H NMR (300 MHz, $CDCl_3$) δ 11.24 (s, 1H), 7.60 (s, 1H), 7.09 (s, 1H), 4.51 – 4.09 (m, 4H), 3.84 (ddd, *J* = 6.0, 4.1, 1.3 Hz, 4H), 3.48 (d, *J* = 2.2 Hz, 6H), 2.78 – 2.67 (t, *J*= 7.4 Hz, 2H), 1.88 (h, *J* = 7.4 Hz, 2H), 1.06 (t, *J* = 7.4 Hz, 3H).
^{13}C NMR δ 163.2, 155.5, 155.2, 148.4, 146.0, 113.9, 109.0, 107.3, 70.9, 70.7, 68.9, 68.6, 59.5, 59.5, 37.8, 21.2, 13.9.
HRMS (ESI) m/z calcd for $C_{17}H_{24}N_2O_5$ + : [M]+ 336.1685; found: 336.1701.

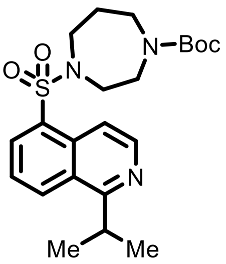

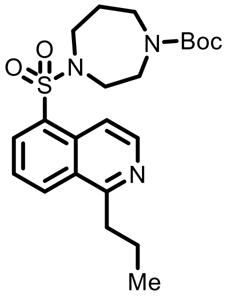

tert-butyl 4-((1-isopropylisoquinolin-5-yl) sulfonyl)-1,4-diazepane-1-carboxylate **(25 major) .** Prepared according to GP2 with the utilization of modification 1 and 0.4 ml of H_2O is used as a co-solvent from 5-((1,4-diazepan-1-yl) sulfonyl)isoquinoline hydrogen chloride (65.4 mg, 0.2 mmol, 1.0 equiv.). Then, the solvent was removed under reduced pressure.

N.B. For the ease of purification, the crude rection mixture was subsequently dissolved in 5 mL of CH_2Cl_2 and Boc-anhydride (66 mg, 1.5 equiv.) was added portion-wise. Then, triethylamine (39 μL, 3 equiv.) was added, and the mixture was stirred for 2h at rt. Next, the solvent was removed under reduced pressure and the crude was purified via flash column chromatography on silica gel (Pentane: Ethyl acetate 1:1) to afford the major product as a yellow gel (28 mg, 32% yield) and minor product as a yellow gel (7 mg, 8% yield).

^{1}H NMR (300 MHz, $CDCl_3$) δ 8.63 (d, *J* = 6.1 Hz, 1H), 8.49 (d, *J* = 8.5 Hz, 1H), 8.29 (dd, *J* = 7.2, 4.1 Hz, 2H), 7.66 (t, *J* = 8.0 Hz, 1H), 3.96 (h, *J* = 6.7 Hz, 1H), 3.51 (dt, *J* = 12.7, 5.8 Hz, 4H), 3.38 (dd, *J* = 11.6, 6.0 Hz, 4H), 1.95 (p, *J* = 6.3 Hz, 2H), 1.45 (d, *J* = 6.7 Hz, 6H), 1.41 (s, 9H).
^{13}C NMR (101 MHz, $CDCl_3$) δ 167.3, 155.2 & 154.9 (rotameric signals), 143.4, 135.3 & 135.2 (rotameric signals), 132.8 & 132.5 (rotameric signals), 132.5, 130.6, 127.0, 125.6, 115.9, 80.1 & 80.0 (rotameric signals), 50.1 & 49.9 (rotameric

signals), 49.4 & 49.3 (rotameric signals), 47.8 & 47.5 (rotameric signals), 46.1 & 45.6 (rotameric signals), 31.6, 28.7 & 28.3 (rotameric signals), 28.5, 22.4.
HRMS (ESI) m/z calcd for $C_{22}H_{31}N_3O_4S$ + : [M]+ 433.2035; found: 433.2065.

tert-butyl 4-((1-propylisoquinolin-5-yl) sulfonyl)-1,4-diazepane-1-carboxylate **(25 minor) .**
^{1}H NMR (300 MHz, $CDCl_3$) δ 8.57 (d, *J* = 6.0 Hz, 1H), 8.41 (d, *J* = 8.5 Hz, 1H), 8.29 (t, *J* = 8.7 Hz, 2H), 7.65 (t, *J* = 7.9 Hz, 1H), 3.51 (dt, *J* = 12.8, 5.8 Hz, 4H), 3.45 – 3.26 (m, 6H), 1.93 (m, 4H), 1.41 (s, 9H), 1.06 (t, *J* = 7.3 Hz, 3H).
^{13}C NMR (101 MHz, $CDCl_3$) δ 163.2, 155.3 & 154.9 (rotameric signals), 143.5, 135.2 & 135.1 (rotameric signals), 132.8 & 132.6 (rotameric signals), 132.3, 131.2, 127.7, 125.6, 116.0, 80.1 & 80.0 (rotameric signals), 50.2 & 49.9 (rotameric signals), 49.5 & 49.3 (rotameric signals), 47.8 & 47.6 (rotameric signals), 46.1 & 45.6 (rotameric signals), 37.8, 28.7 & 28.3 (rotameric signals), 28.5, 23.3, 14.4.
HRMS (ESI) m/z calcd for $C_{22}H_{31}N_3O_4S$ + : [M]+ 433.2035; found: 433.2044.

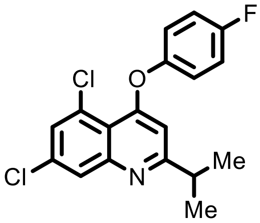

Major (**38**)

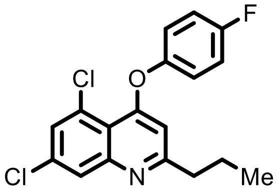

Minor (**39**)

5,7-dichloro-4-(4-fluorophenoxy)-2-isopropylquinoline **(38).** Prepared according to GP1 from 5,7-dichloro-4-(4-fluorophenoxy) quinoline (61.6 mg, 0.2 mmol, 1.0 equiv.). Purified via flash column chromatography on silica gel (from Pentane to Pentane: Ethyl acetate 10:1) to afford the major product (**38**) as a white solid (28 mg, 40% yield) and minor (**39**) product as a white solid (14.7 mg, 21% yield).

Characterization data are in accordance with literature.[4]

^{1}H NMR (300 MHz, $CDCl_3$) δ 7.96 (d, *J* = 2.1 Hz, 1H), 7.51 (d, *J* = 2.1 Hz, 1H), 7.22 – 7.00 (m, 4H), 6.53 (s, 1H), 3.03 (hept, *J* = 6.9 Hz, 1H), 1.25 (d, *J* = 6.9 Hz, 6H).
^{13}C NMR (101 MHz, Chloroform-*d*) δ 170.4, 162.4, 160.0 (d, *J* = 244.0 Hz), 151.4, 150.3 (d, *J* = 2.8 Hz), 134.8, 129.9, 128.7, 127.6, 121.9 (d, *J* = 8.4 Hz), 117.3, 117.0, 105.4, 37.1, 22.1.
^{19}F NMR (282 MHz, $CDCl_3$) δ -117.37.
HRMS (ESI) m/z calcd for $C_{18}H_{14}Cl_2FNO$ +: [M]+ 349.0436; found: 349.0475.

5,7-dichloro-4-(4-fluorophenoxy)-2-propylquinoline **(39).**
^{1}H NMR (300 MHz, $CDCl_3$) δ 8.03 (d, *J* = 2.1 Hz, 1H), 7.55 (d, *J* = 1.8 Hz, 1H), 7.23 – 7.02 (m, 4H), 6.50 (s, 1H), 2.84 – 2.73 (m, 2H), 1.83 – 1.59 (m, 2H), 0.95 (t, *J* = 7.3 Hz, 3H).
^{13}C NMR (101 MHz, $CDCl_3$) δ 165.6, 163.2, 161.2 (d, *J* = 242.4 Hz), 150.0, 150, 135.8, 130.4, 129.3, 126.6, 122.3 (d, *J* = 8.5 Hz), 117.3 117.2, 106.9, 40.5, 22.9, 14.0.
^{19}F NMR (282 MHz, $CDCl_3$) δ -117.17.
HRMS (ESI) m/z calcd for $C_{18}H_{14}Cl_2FNO$ +: [M]+ 349.0436; found: 349.0463.

Butane:

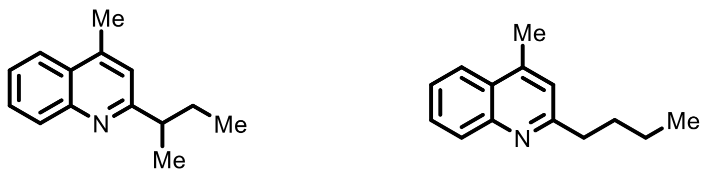

Major

Minor

2-(sec-butyl)-4-methylquinoline (**26 major*****).*** Prepared according to GP3 from lepidine (28.6 mg, 0.2 mmol, 1.0 equiv.). Purified via flash column chromatography on silica gel (from Pentane to Pentane:Ethyl acetate 50:1) to afford the major product as a colorless oil (16.7 mg, 42% yield) and minor product as a colorless oil (8.3 mg, 20% yield).

Characterization data are in accordance with literature.[11]

^{1}H NMR (400 MHz, $CDCl_3$) δ 8.05 (d, *J* = 8.4 Hz, 1H), 7.95 (d, *J* = 8.3 Hz, 1H), 7.72 – 7.62 (m, 1H), 7.50 (dd, *J* = 8.5, 6.7 Hz, 1H), 7.13 (s, 1H), 2.95 (h, *J* = 7.1 Hz, 1H), 2.69 (s, 3H), 1.84 (dq, *J* = 14.9, 7.4 Hz, 1H), 1.71 (tq, *J* = 14.3, 7.3 Hz, 1H), 1.35 (d, *J* = 6.9 Hz, 3H), 0.89 (t, *J* = 7.4 Hz, 3H).
^{13}C NMR (101 MHz, $CDCl_3$) δ 166.9, 147.8, 144.3, 129.7, 129.0, 127.2, 125.5, 123.7, 120.3, 44.7, 30.1, 20.5, 19.0, 12.4.
HRMS (ESI) m/z calcd for $C_{14}H_{17}N$+: [M]+ 199.1361; found: 199.1395.

Characterization data are in accordance with literature.[12]

2-butyl-4-methylquinoline **(26 minor).**
^{1}H NMR (400 MHz, $CDCl_3$) δ 8.04 (d, *J* = 8.5 Hz, 1H), 7.98 – 7.89 (m, 1H), 7.67 (ddd, *J* = 8.5, 6.8, 1.6 Hz, 1H), 7.56 – 7.46 (m, 1H), 7.15 (s, 1H), 2.92 (t, *J* = 8.0 Hz, 2H), 2.68 (s, 3H), 1.79 (ddd, *J* = 7.8, 5.3, 1.9 Hz, 2H), 1.44 (q, *J* = 7.5 Hz, 2H), 0.96 (t, *J* = 7.3 Hz, 3H).
^{13}C NMR (101 MHz, $CDCl_3$)) δ 163.0, 147.9, 144.3, 129.5, 129.1, 126.9, 125.5, 123.7, 122.2, 39.2, 32.4, 22.9, 18.8, 14.2.
HRMS (ESI) m/z calcd for $C_{14}H_{17}N$+: [M]+ 199.1361; found: 199.1384.

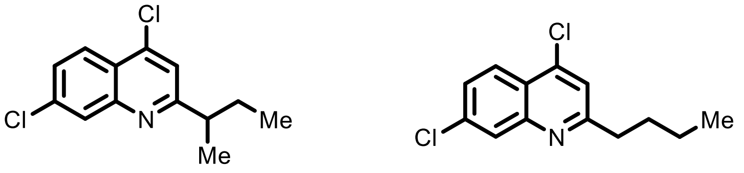

Major

Minor

2-(sec-butyl)-4,7-dichloroquinoline **(27 major).** Prepared according to GP3, Modification 1, from 4,7-dichloroquinoline (39.6 mg, 0.2 mmol, 1.0 equiv.). Purified via flash column chromatography on silica gel (from Pentane to Pentane:Ethyl acetate 50:1) to afford the major product as a yellow liquid (12 mg, 24% yield) and minor product as a yellow liquid (5.5 mg, 11% yield).

^{1}H NMR (400 MHz, $CDCl_3$) δ 8.17 – 7.91 (m, 2H), 7.52 (dd, *J* = 8.9, 2.1 Hz, 1H), 7.37 (s, 1H), 2.96 (h, *J* = 7.0 Hz, 1H), 1.98 – 1.60 (m, 2H), 1.35 (d, *J* = 6.9 Hz, 3H), 0.89 (t, *J* = 7.4 Hz, 3H).
^{13}C NMR (101 MHz, $CDCl_3$) δ 168.5, 149.1, 142.7, 136.4, 128.5, 127.7, 125.5, 123.8, 120.3, 44.5, 29.9, 20.2, 12.3.
HRMS (ESI) m/z calcd for $C_{13}H_{13}Cl_2N$+ : [M]+ 253.0425; found: 253.0430.

2-butyl-4,7-dichloroquinoline **(27 minor).**
^{1}H NMR (400 MHz, $CDCl_3$) δ 8.24 – 7.96 (m, 2H), 7.52 (dd, *J* = 8.9, 2.0 Hz, 1H), 7.38 (s, 1H), 3.12 – 2.76 (m, 2H), 1.97 – 1.65 (m, 2H), 1.43 (q, *J* = 7.4 Hz, 2H), 0.96 (t, *J* = 7.3 Hz, 3H).
^{13}C NMR δ 164.5, 149.2, 142.7, 136.5, 128.3, 127.8, 125.5, 123.6, 121.7, 38.9, 31.9, 22.7, 14.1.

HRMS (ESI) m/z calcd for $C_{13}H_{13}Cl_2N$+ : [M]+ 253.0425; found: 253.0441.

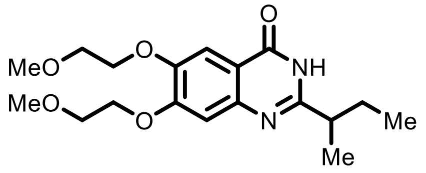

Major

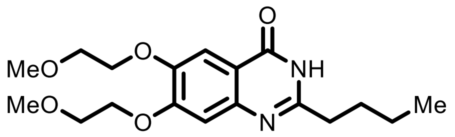

Minor

2-(sec-butyl)-6,7-bis(2-methoxyethoxy) quinazolin-4(3H)-one **(28 major)** Prepared according to GP4, butane as gaseous alkane and 60 min. residence time, from 6,7-bis(2-methoxyethoxy) quinazolin-4(3H)-one (58.8 mg, 0.2 mmol, 1.0 equiv.). Purified via flash column chromatography on silica gel (Pentane: Ethyl acetate 1:1 to ethyl acetate) to afford the major product as a white solid (19 mg, 26% yield) and minor product as a white solid (10 mg, 15% yield).

^{1}H NMR (300 MHz, $CDCl_3$) δ 11.06 (s, 1H), 7.60 (s, 1H), 7.10 (s, 1H), 4.41 – 4.17 (m, 4H), 3.84 (m, 4H), 3.47 (d, *J* = 2.2 Hz, 6H), 2.74 (h, *J* = 7.0 Hz, 1H), 1.99 – 1.79 (m, 1H), 1.71 (dt, *J* = 13.9, 7.0 Hz, 1H), 1.38 (d, *J* = 7.0 Hz, 3H), 0.96 (t, *J* = 7.4 Hz, 3H).
^{13}C NMR (101 MHz, $CDCl_3$) δ 163.2, 159.2, 155.2, 148.3, 146.0, 114.1, 109.1, 70.9, 70.6, 68.9, 68.5, 59.4 (2C), 42.1, 28.4, 18.5, 12.1.
HRMS (ESI) m/z calcd for $C_{18}H_{26}N_2O_5$+ : [M]+ 350.1842; found: 350.1877.

6,7-bis(2-methoxyethoxy)-2-propylquinazolin-4(3H)-one **(28 minor).**
^{1}H NMR (300 MHz, $CDCl_3$) δ 11.29 (s, 1H), 7.59 (s, 1H), 7.09 (s, 1H), 4.36 – 4.11 (m, 4H), 3.84 (m, 4H), 3.48 (d, *J* = 1.9 Hz, 6H), 2.90 – 2.56 (m, 2H), 1.92 – 1.78 (m, 2H), 1.55 – 1.39 (m, 2H), 0.98 (t, *J* = 7.3 Hz, 3H).
^{13}C NMR δ (101 MHz, $CDCl_3$) δ 163.3, 155.7, 155.2, 148.4, 146.0, 113.8, 108.9, 107.2, 70.9, 70.6, 68.8, 68.6, 59.5, 59.4, 35.7, 29.8, 22.5, 13.9.
HRMS (ESI) m/z calcd for $C_{18}H_{26}N_2O_5$+ : [M]+ 350.1842; found: 350.1869.

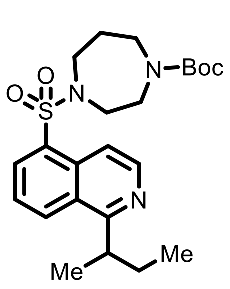

Major

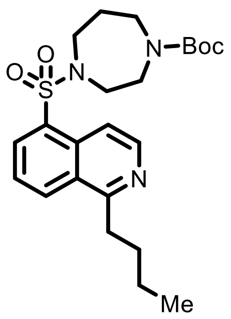

Minor

tert-butyl 4-((1-(sec-butyl) isoquinolin-5-yl) sulfonyl)-1,4-diazepane-1-carboxylate **(29 major) .** Prepared according to GP3 with the utilization of modification 1 and 0.4 ml of H_2O is used as a co-solvent from 5-((1,4-diazepan-1-yl)sulfonyl)isoquinoline hydrogen chloride (65.4 mg, 0.2 mmol, 1.0 equiv.). Then, the solvent was removed under reduced pressure.

N.B. For the ease of purification, the crude rection mixture was subsequently dissolved in 5 mL of CH_2Cl_2 and Boc-anhydride (66 mg, 1.5 equiv.) was added portion-wise. Then, triethylamine (39 µL, 3 equiv.) was added, and the mixture was stirred for 2h at rt. Next, the solvent was removed under reduced pressure and the crude was purified via flash column chromatography on silica gel (Pentane: Ethyl acetate 1:1) to afford the major product as a yellow gel (28.3mg, 32% yield) and minor product as a yellow gel (6 mg, 6% yield).

^{1}H NMR (300 MHz, $CDCl_3$) δ 8.64 (d, *J* = 6.1 Hz, 1H), 8.48 (d, *J* = 8.6 Hz, 1H), 8.34 – 8.21 (m, 2H), 7.64 (t, *J* = 8.0 Hz, 1H), 3.70 (p, *J* = 6.8 Hz, 1H), 3.52 (dt, *J* = 12.7, 5.9 Hz, 4H), 3.45 – 3.30 (m, 4H), 2.13 – 1.91 (m, 3H), 1.77 (dp, *J* = 14.3, 7.3 Hz, 1H), 1.41 (d, *J* = 7.3 Hz, 12H), 0.89 (t, *J* = 7.4 Hz, 3H).

^{13}C NMR (101 MHz, $CDCl_3$) δ 167.0, 155.3 & 154.9 (rotameric signals), 143.8, 135.2 & 135.1 (rotameric signals), 132.6, 132.4, 130.6, 127.6, 125.4, 115.5, 80.1 & 80.0 (rotameric signals), 50.2 & 50.0 (rotameric signals), 49.5 & 49.4 (rotameric signals), 47.8 & 47.6 (rotameric signals), 46.1 & 45.6 (rotameric signals), 38.4, 29.8, 28.7 & 28.3 (rotameric signals), 28.5, 20.5, 12.4.
HRMS (ESI) m/z calcd for $C_{23}H_{33}N_3O_4S$ + : [M]+ 447.2192; found: 447.2199.

tert-butyl 4-((1-propylisoquinolin-5-yl)sulfonyl)-1,4-diazepane-1-carboxylate **(29 minor) .**
^{1}H NMR (300 MHz, $CDCl_3$) δ 8.58 (d, *J* = 6.2 Hz, 1H), 8.42 (d, *J* = 8.5 Hz, 1H), 8.35 – 8.22 (m, 2H), 7.65 (t, *J* = 7.9 Hz, 1H), 3.51 (t, *J* = 6.4 Hz, 4H), 3.43 – 3.28 (m, 6H), 1.96 (p, *J* = 6.3 Hz, 2H), 1.85 (p, *J* = 7.6 Hz, 2H), 1.55 – 1.46 (m, 2H), 1.43 (s, 9H), 0.99 (t, *J* = 7.3 Hz, 3H).
^{13}C NMR (101 MHz, $CDCl_3$) δ 166.9, 155.2, 144.0, 135.0, 132.4, 132.2, 130.5, 127.5, 125.1, 115.3, 79.9, 50.1 & 49.9 (rotameric signals), 49.3, 47.7, 46.0 & 45.5 (rotameric signals), 38.3, 29.7, 28.6, 28.2 & 28.4 (rotameric signals), 20.3, 12.3.
HRMS (ESI) m/z calcd for $C_{23}H_{33}N_3O_4S$ + : [M]+ 447.2192; found: 447.2170.

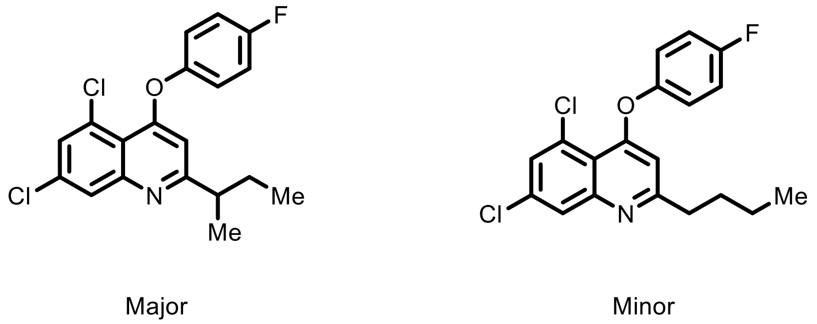

2-(sec-butyl)-5,7-dichloro-4-(4-fluorophenoxy) quinoline **(41 major).** Prepared according to GP1 from 5,7-dichloro-4-(4-fluorophenoxy) quinoline (61.6 mg, 0.2 mmol, 1.0 equiv.). Purified via flash column chromatography on silica gel (from Pentane to Pentane: Ethyl acetate 10:1) to afford the major product as a white solid (27.5 mg, 38% yield) and minor product as a white solid (10.2 mg, 14% yield).

Characterization data are in accordance with literature.[13]

^{1}H NMR (300 MHz, $CDCl_3$) δ 7.97 (d, *J* = 2.2 Hz, 1H), 7.51 (d, *J* = 2.2 Hz, 1H), 7.18 – 7.07 (m, 4H), 6.50 (s, 1H), 2.78 (h, *J* = 7.0 Hz, 1H), 1.74 – 1.51 (m, 2H), 1.22 (d, *J* = 6.9 Hz, 3H), 0.82 (t, *J* = 7.4 Hz, 3H).
^{13}C NMR (101 MHz, $CDCl_3$) δ 169.9, 162.4, 160.0 (d, *J* = 244.3 Hz), 151.5, 150.5 (d, *J* = 2.8 Hz), 134.9, 130.0, 128.8, 127.7, 122.0 (d, *J* = 8.4 Hz), 117.3, 117.0, 106.0, 44.5, 29.7, 20.0, 12.2.
^{19}F NMR (282 MHz, $CDCl_3$) δ -117.37.
HRMS (ESI) m/z calcd for $C_{19}H_{16}Cl_2FNO$ +: [M]+ 363.0593; found: 363.0625.

2-butyl-5,7-dichloro-4-(4-fluorophenoxy) quinoline **(40 minor).**
^{1}H NMR (300 MHz, $CDCl_3$) δ 7.95 (d, *J* = 2.1 Hz, 1H), 7.52 (d, *J* = 2.1 Hz, 1H), 7.20 – 7.05 (m, 4H), 6.48 (s, 1H), 2.81 – 2.70 (t, *J* = 7.4 Hz, 2H), 1.75 – 1.56 (m, 2H), 1.37 (dt, *J* = 15.0, 7.4 Hz, 2H), 0.91 (t, *J* = 7.3 Hz, 3H).
^{13}C NMR (101 MHz, $CDCl_3$) δ 169.9, 162.4, 160.0 (d, *J* = 244.0 Hz), 151.6, 150.5 (d, *J* = 2.6 Hz), 134.9, 130.0, 128.8, 127.7, 122.0 (d, *J* = 8.4 Hz), 117.3, 117.0, 106.0, 44.5, 29.6, 20.0, 12.2.
^{19}F NMR (282 MHz, $CDCl_3$) δ -117.42.
HRMS (ESI) m/z calcd for $C_{19}H_{16}Cl_2FNO$ +: [M]+ 363.0593; found: 363.0635.

Methane:

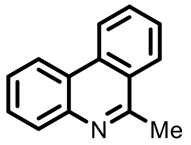

***6-methylphenanthridine* (30).** Prepared according to GP4 from Phenanthridine (54 mg, 0.3 mmol, 1.0 equiv.). Purified via flash column chromatography on silica gel (from Pentane to Pentane:Ethyl acetate 50:1) to afford the product as a yellow solid (24 mg, 41% yield).

Characterization data are in accordance with literature.[14]

^{1}H NMR (400 MHz, $CDCl_3$) δ 8.63 (d, *J* = 8.2 Hz, 1H), 8.58 – 8.52 (m, 1H), 8.29 – 8.20 (m, 1H), 8.10 (dd, *J* = 8.2, 1.4 Hz, 1H), 7.85 (ddd, *J* = 8.2, 7.0, 1.3 Hz, 1H), 7.76 – 7.66 (m, 2H), 7.62 (ddd, *J* = 8.3, 7.0, 1.4 Hz, 1H), 3.05 (s, 3H).
^{13}C NMR (101 MHz, $CDCl_3$) δ 158.9, 143.7, 132.6, 130.5, 129.4, 128.7, 127.3, 126.6, 126.3, 125.9, 123.8, 122.3, 122.0, 23.4.
HRMS (ESI) m/z calcd for $C_{14}H_{11}N^+$: [M]+ 193.0891; found: 193.0877.

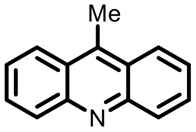

***9-methylacridine* (31).** Prepared according to GP4 from acridine (54 mg, 0.3 mmol, 1.0 equiv.). Purified via flash column chromatography on silica gel (from Pentane to Pentane:Ethyl acetate 50:1) to afford the product as a brown solid (30 mg, 51% yield).

^{1}H NMR (400 MHz, $CDCl_3$) δ 8.23 (m, 4H), 7.76 (ddd, *J* = 8.9, 6.6, 1.3 Hz, 2H), 7.54 (ddd, *J* = 8.8, 6.5, 1.2 Hz, 2H), 3.11 (s, 3H).
^{13}C NMR (101 MHz, $CDCl_3$) δ 148.4, 142.6, 130.2, 130.0, 125.6, 125.5, 124.7, 13.8.
HRMS (ESI) m/z calcd for $C_{14}H_{11}N^+$: [M]+ 193.0891; found: 193.0878.

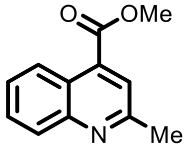

***methyl 2-methylquinoline-4-carboxylate* (32).** Prepared according to GP4 from methyl quinoline-4-carboxylate (56.1 mg, 0.3 mmol, 1.0 equiv.). Purified via flash column chromatography on silica gel (from Pentane to Pentane:Ethyl acetate 50:1) to afford the product as a white solid (18 mg, 29% yield).

^{1}H NMR (400 MHz, $CDCl_3$) δ 8.68 (ddd, *J* = 8.5, 1.5, 0.6 Hz, 1H), 8.18 – 8.04 (m, 1H), 7.80 (s, 1H), 7.73 (ddd, *J* = 8.4, 6.9, 1.5 Hz, 1H), 7.58 (ddd, *J* = 8.4, 6.9, 1.4 Hz, 1H), 4.03 (s, 3H), 2.79 (s, 3H).
^{13}C NMR (101 MHz, $CDCl_3$) δ 166.9, 158.6, 148.9, 135.2, 129.9, 129.3, 127.4, 125.5, 123.4, 123.5, 52.8, 25.4.
HRMS (ESI) m/z calcd for $C_{12}H_{11}NO_2^+$: [M]+201.0790; found: 201.0750.

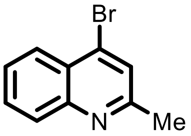

***4-bromo-2-methylquinoline* (33).** Prepared according to GP4 from 4-bromoquinoline (62.4 mg, 0.2 mmol, 1.0 equiv.). Purified via flash column chromatography on silica gel (from Pentane to Pentane:Ethyl acetate 50:1) to afford the product as a yellow oil (24 mg, 36% yield).

Characterization data are in accordance with literature.[15]

^{1}H NMR (400 MHz, $CDCl_3$) δ 8.18 – 8.10 (m, 1H), 8.07 – 7.97 (m, 1H), 7.72 (ddd, *J* = 8.4, 6.9, 1.5 Hz, 1H), 7.66 – 7.52 (m, 2H), 2.72 (s, 3H).
^{13}C NMR (101 MHz, $CDCl_3$) δ 159.0, 148.6, 134.3, 130.5, 129.2, 127.1, 126.7, 126.2, 126.0, 25.1.
HRMS (ESI) m/z calcd for $C_{10}H_8BrN$ + : [M]+220.9840; found: 220.9885.

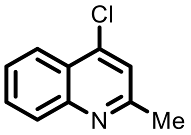

***4-chloro-2-methylquinoline* (34).** Prepared according to GP4 from 4-chloroquinoline (49 mg, 0.3 mmol, 1.0 equiv.). Purified via flash column chromatography on silica gel (from Pentane to Pentane:Ethyl acetate 50:1) to afford the product as a yellow oil (18 mg, 33% yield).

^{1}H NMR (400 MHz, $CDCl_3$) δ 8.19 (dd, *J* = 8.5, 1.5 Hz, 1H), 8.09 (d, *J* = 8.4 Hz, 1H), 7.75 (ddd, *J* = 8.5, 6.9, 1.5 Hz, 1H), 7.60 (ddd, *J* = 8.2, 6.9, 1.1 Hz, 1H), 7.42 (s, 1H), 2.75 (s, 3H).
^{13}C NMR (101 MHz, $CDCl_3$) 158.8, 131.1, 129.7, 128.4, 127.2, 125.0, 124.2, 122.2, 115.5, 24.8.
HRMS (ESI) m/z calcd for $C_{10}H_8ClN$ + : [M]+ 177.0345; found: 177.0370.

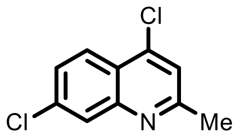

***4,7-dichloro-2-methylquinoline* (35).** Prepared according to GP4 from 4,7-dichloroquinoline (49 mg, 0.3 mmol, 1.0 equiv.). Purified via flash column chromatography on silica gel (from Pentane to Pentane:Ethyl acetate 50:1) to afford the product as a white solid (22 mg, 37% yield).

^{1}H NMR (400 MHz, $CDCl_3$) δ 8.10 (d, *J* = 8.9 Hz, 1H), 8.02 (d, *J* = 2.1 Hz, 1H), 7.51 (dd, *J* = 8.9, 2.1 Hz, 1H), 7.37 (s, 1H), 2.70 (s, 3H).
^{13}C NMR (101 MHz, $CDCl_3$) δ 160.4, 149.2, 142.6, 136.6, 128.1, 127.8, 125.5, 123.4, 122.3, 25.3.
HRMS (ESI) m/z calcd for $C_{10}H_7Cl_2N$ + : [M]+ 210.9956; found: 210.9990.

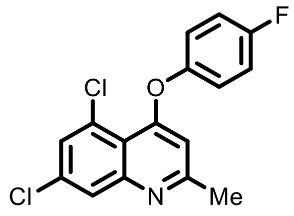

***5,7-dichloro-4-(4-fluorophenoxy)-2-methylquinoline* (36).** Prepared according to GP4 from 5,7-dichloro-4-(4-fluorophenoxy) quinoline (61.6 mg, 0.2 mmol, 1.0 equiv.). Purified via flash column chromatography on silica gel (from Pentane to Pentane:Ethyl acetate 50:1) to afford the product as a white solid (7.1 mg, 11% yield).

Characterization data are in accordance with

^{1}H NMR (400 MHz, $CDCl_3$) δ 7.92 (d, *J* = 2.1 Hz, 1H), 7.52 (d, *J* = 2.1 Hz, 1H), 7.21 – 6.95 (m, 4H), 6.47 (s, 1H), 2.55 (s, 3H).
^{13}C NMR (101 MHz, $CDCl_3$) δ 162.5, 161.9, 160.2 (d, *J* = 244.9 Hz), 151.5, 150.1, 135.1, 130.2, 128.9, 127.3, 122.4 (d, *J* = 8.3 Hz), 117.3, 117.11, 107.5, 25.5.
^{19}F NMR (282 MHz, $CDCl_3$) δ -116.96.
HRMS (ESI) m/z calcd for $C_{16}H_{10}Cl_2FNO+$: [M]+321.0123; found: 321.0169.

8. Proposed Mechanism

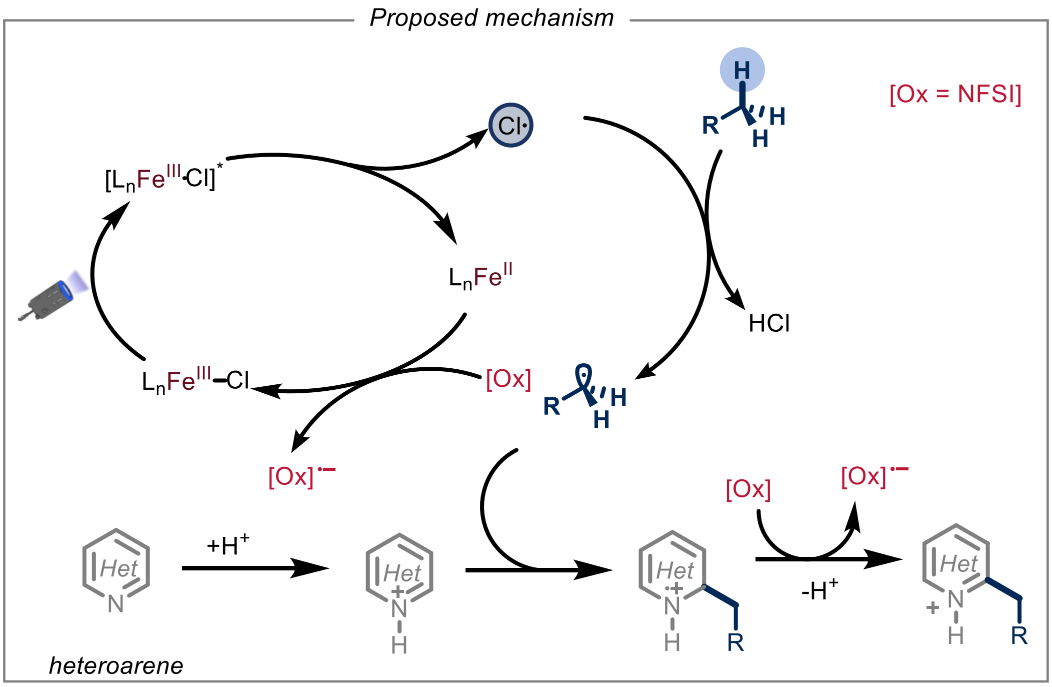

Figure S13: Proposed reaction mechanism

9. Limitation of the scope

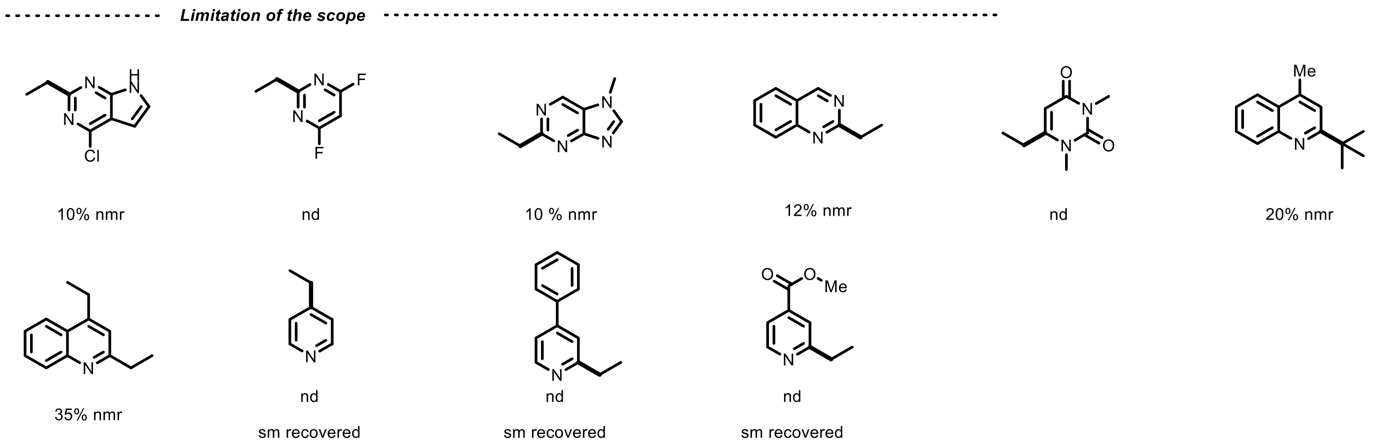

Figure S14: Limitations of the protocol.

10. References

[1] C. Jiang, Y. Liao, H. Li, S. Zhang, P. Liu, P. Sun, *Adv. Synth. Catal.* **2023**, *365*, 1205-1210.
[2] J. M. dos Santos Filho, M. V. B. de Souza Castro, *J. Organomet. Chem.* **2022**, *979*, 122488.
[3] J. Dong, F. Yue, H. Song, Y. Liu, Q. Wang, *Chem. Commun.* **2020**, *56*, 12652-12655.
[4] D.-S. Li, T. Liu, Y. Hong, C.-L. Cao, J. Wu, H.-P. Deng, *ACS Cat.* **2022**, *12*, 4473-4480.
[5] X. Gu, T. Wang, K. Yan, *Org. Lett.* **2023**, *25*, 7287-7292.
[6] Z. Li, J. Dong, X. Chen, Q. Li, Y. Zhou, S.-F. Yin, *J. Org. Chem.* **2015**, *80*, 9392-9400.
[7] G.-X. Li, C. A. Morales-Rivera, Y. Wang, F. Gao, G. He, P. Liu, G. Chen, *Chem. Sci.* **2016**, *7*, 6407-6412.
[8] C. Wang, H. Shi, G.-J. Deng, H. Huang, *Org. Biomol. Chem.* **2021**, *19*, 9177-9181.
[9] J. Dong, Z. Wang, X. Wang, H. Song, Y. Liu, Q. Wang, *J. Org. Chem.* **2019**, *84*, 7532-7540.
[10] Z. Wang, Q. Liu, X. Ji, G.-J. Deng, H. Huang, *ACS Cat.* **2020**, *10*, 154-159.
[11] Z. Wang, X. Ji, J. Zhao, H. Huang, *Green Chem.* **2019**, *21*, 5512-5516.
[12] L. Zhang, Z.-Q. Liu, *Org. Lett.* **2017**, *19*, 6594-6597.
[13] Y. Wang, L. Yang, S. Liu, L. Huang, Z.-Q. Liu, *Adv. Synth. Catal.* **2019**, *361*, 4568-4574.
[14] W. Liu, X. Yang, Z.-Z. Zhou, C.-J. Li, *Chem* **2017**, *2*, 688-702.
[15] P. Natarajan, A. Basak, O. Metin, *Green Chem.* **2023**, *25*, 3187-3197.

11. NMR Spectra

^{1}H NMR (400 MHz, $CDCl_3$) of **2**

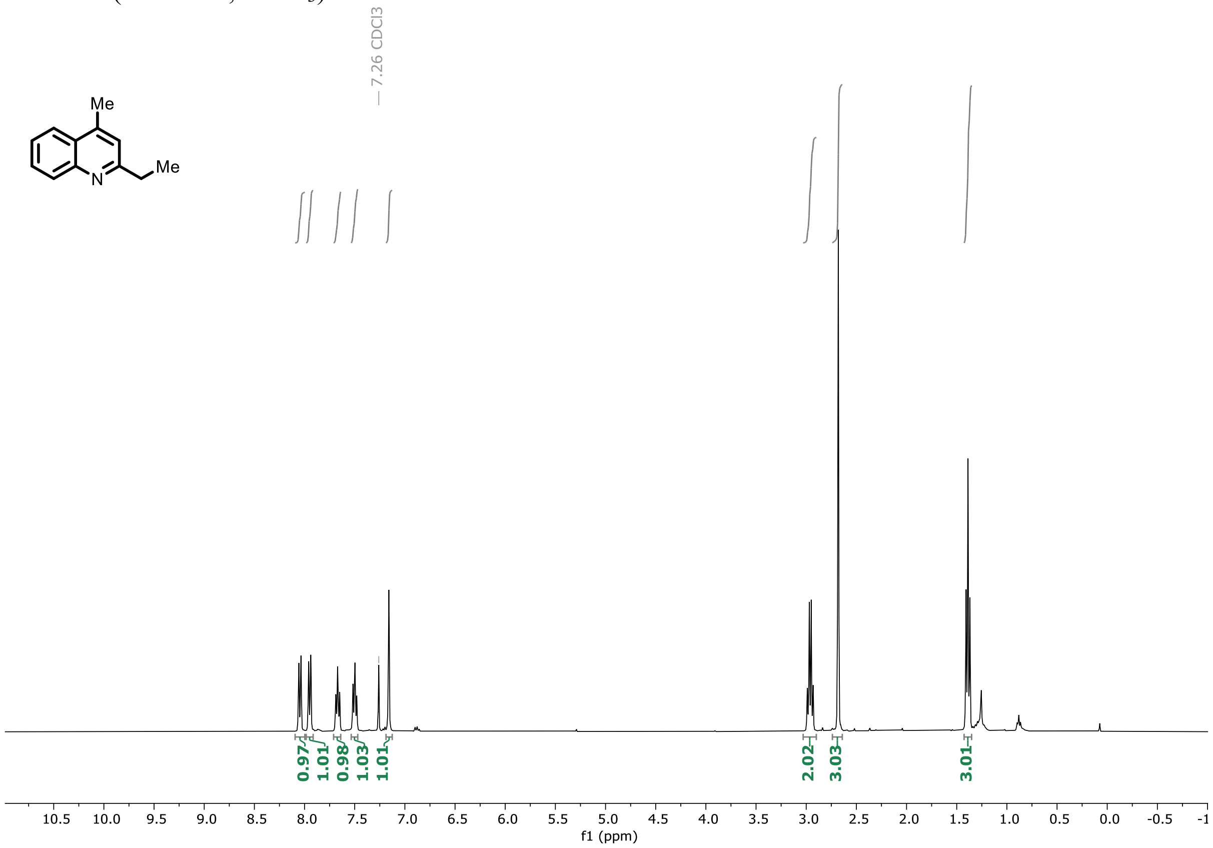

^{13}C NMR (101 MHz, $CDCl_3$) of **2**

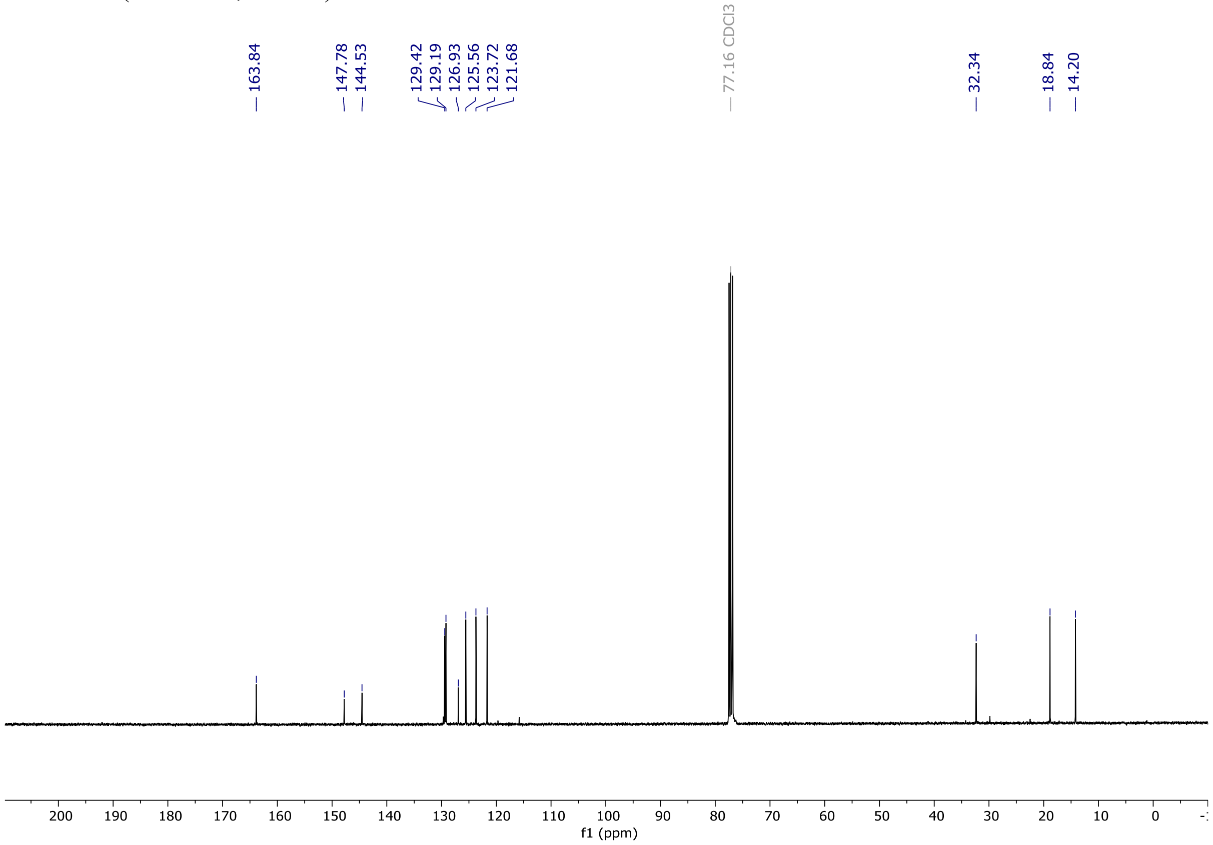

^{1}H NMR (400 MHz, $CDCl_3$) of **3**

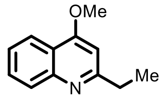

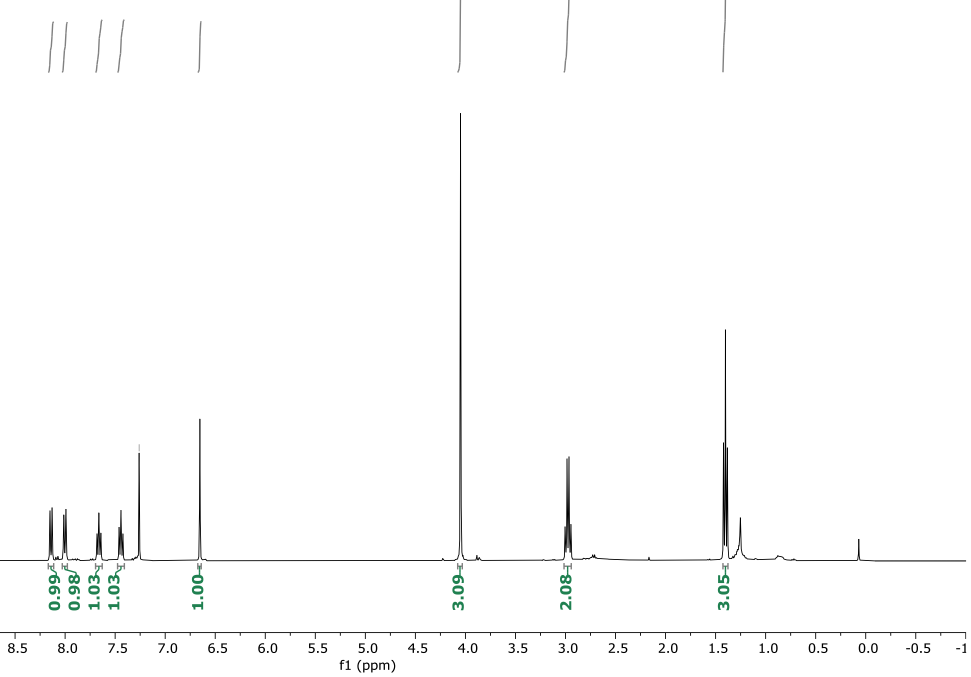

^{13}C NMR (101 MHz, $CDCl_3$) of **3**

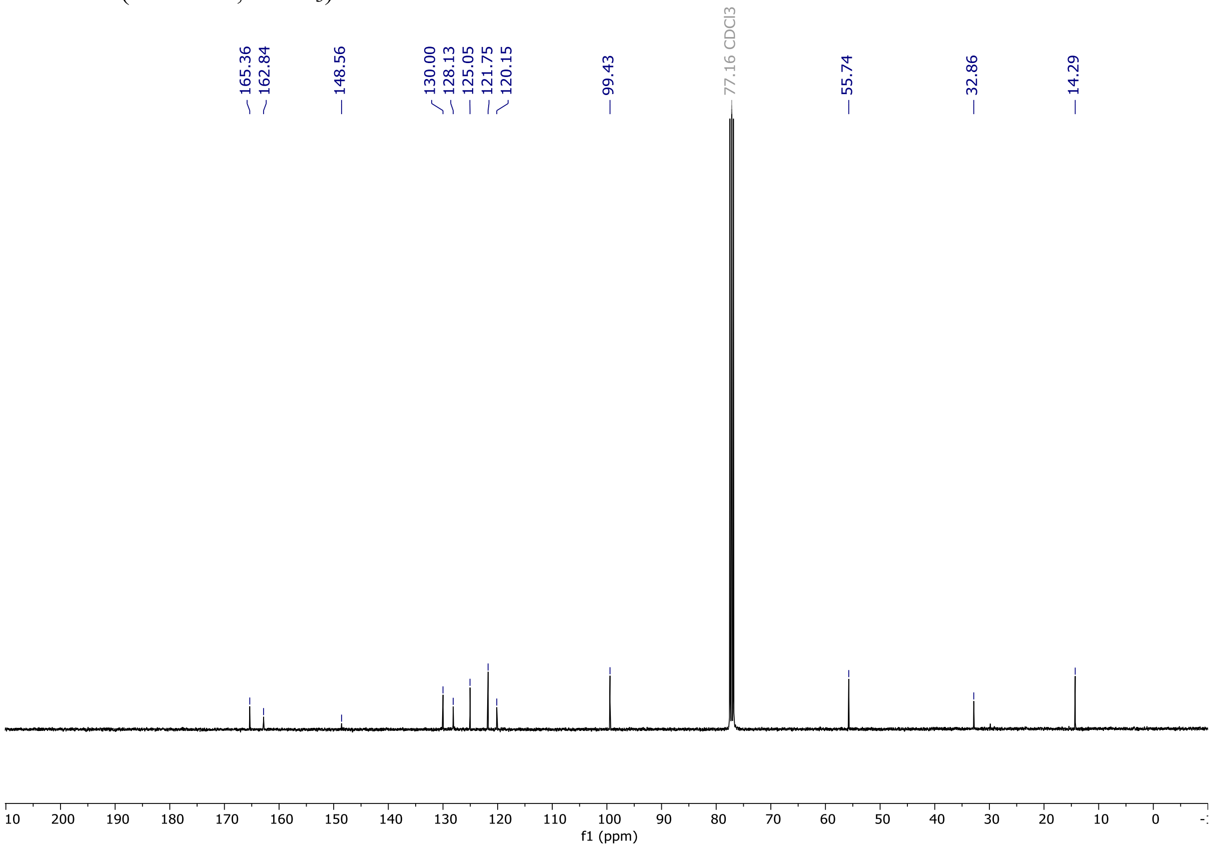

^{1}H NMR (400 MHz, $CDCl_3$) of **4**

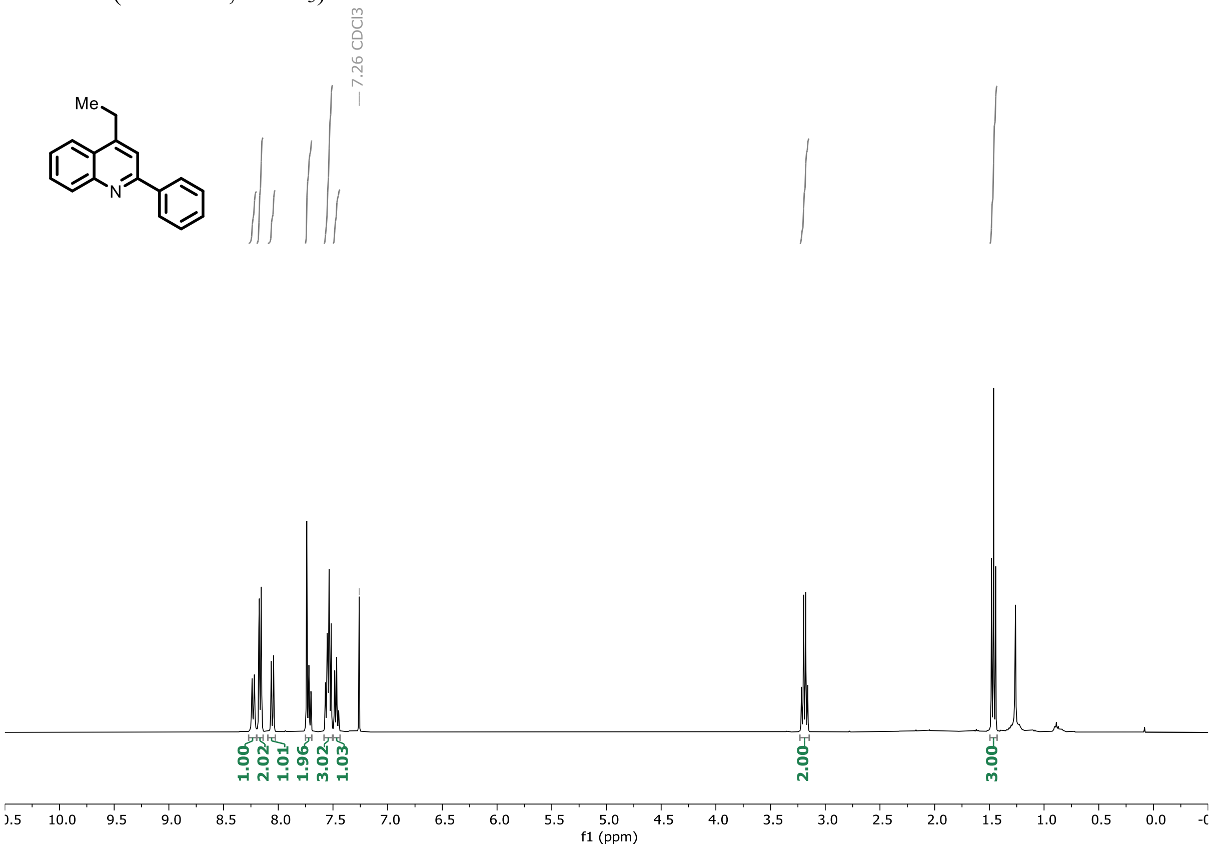

^{13}C NMR (101 MHz, $CDCl_3$) of **4**

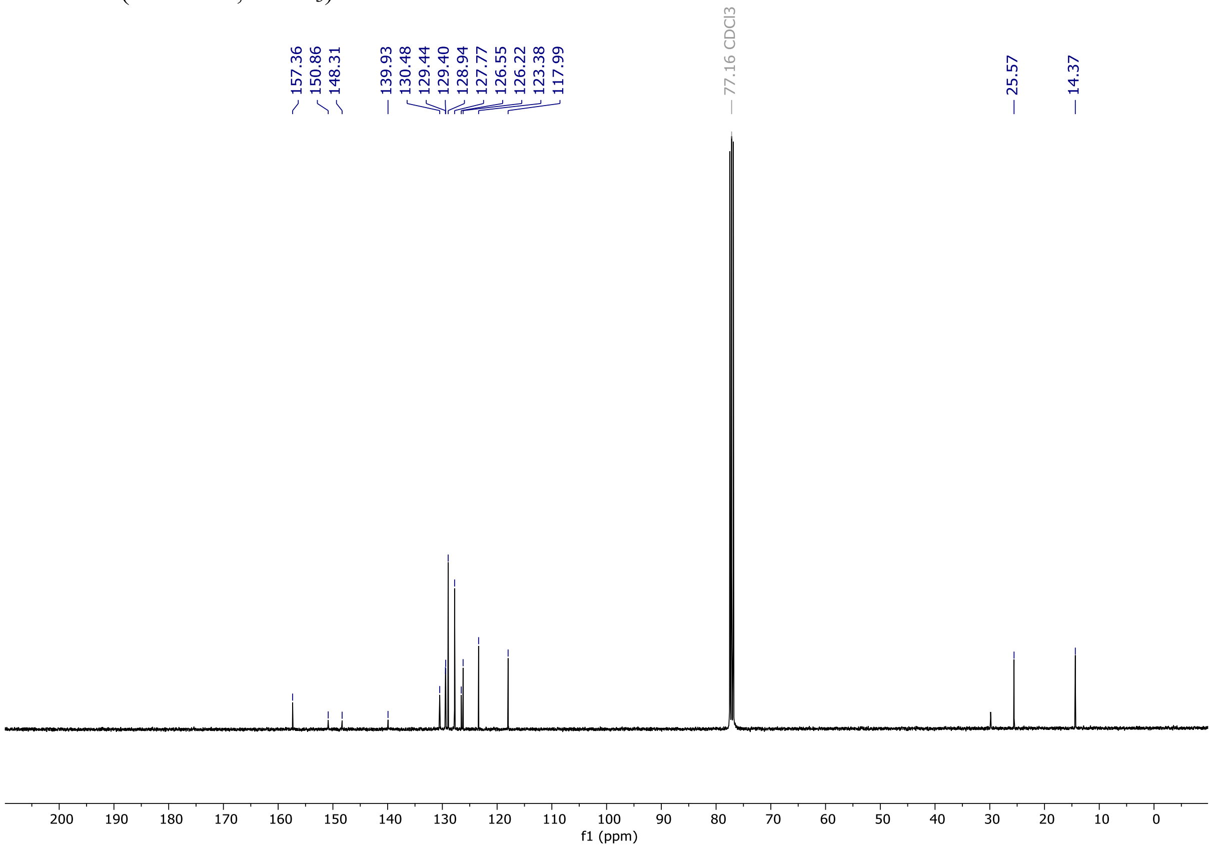

^{1}H NMR (400 MHz, $CDCl_3$) of **5**

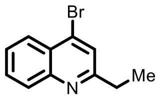

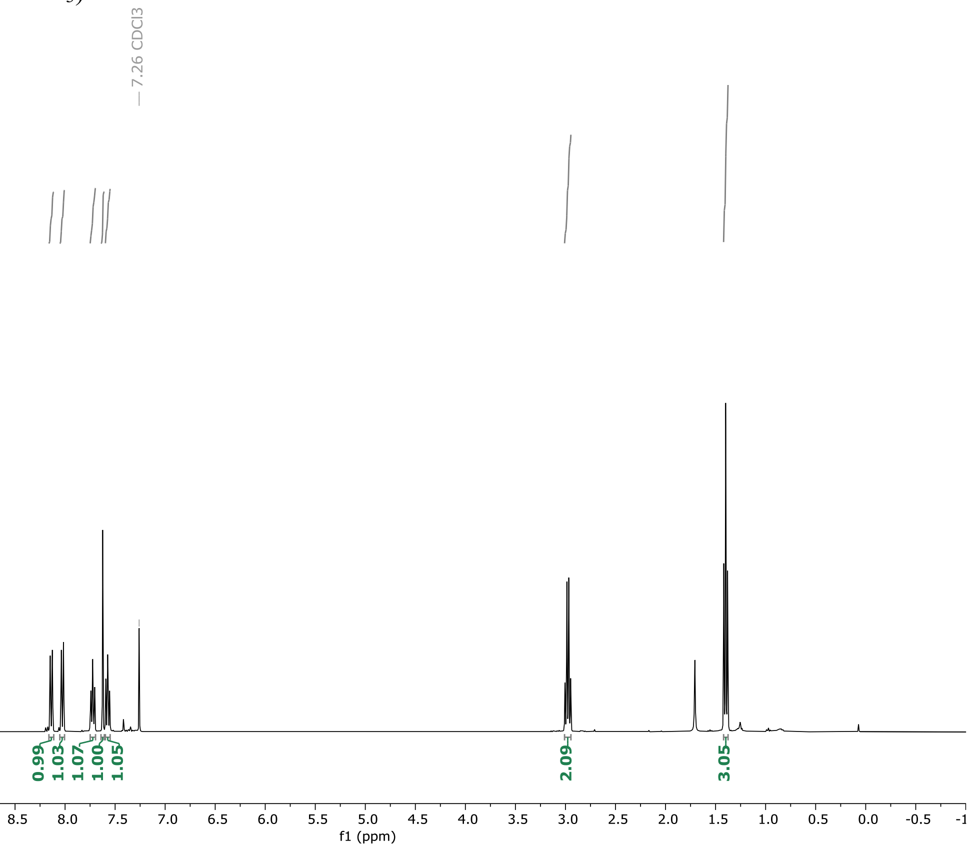

^{13}C NMR (101 MHz, $CDCl_3$) of **5**

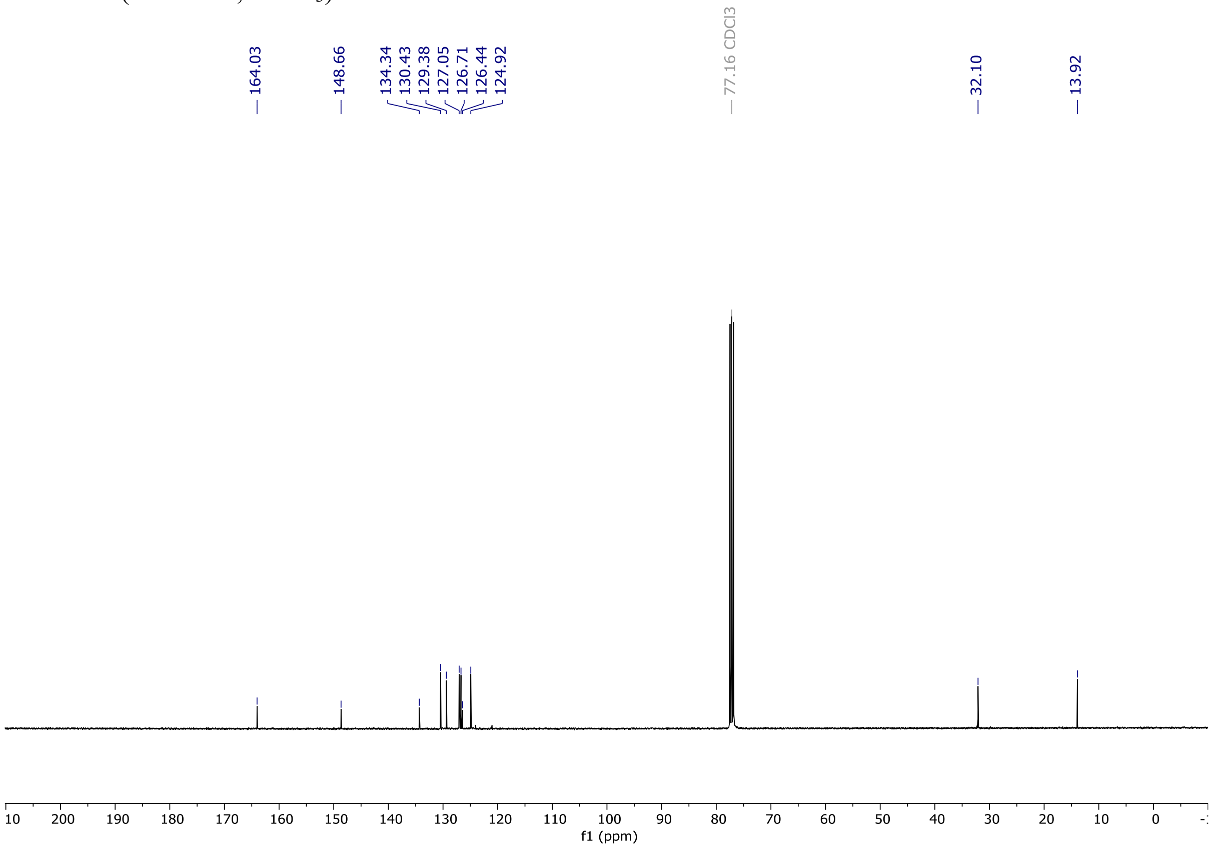

^{1}H NMR (400 MHz, $CDCl_3$) of **6**

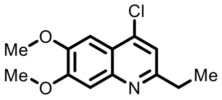

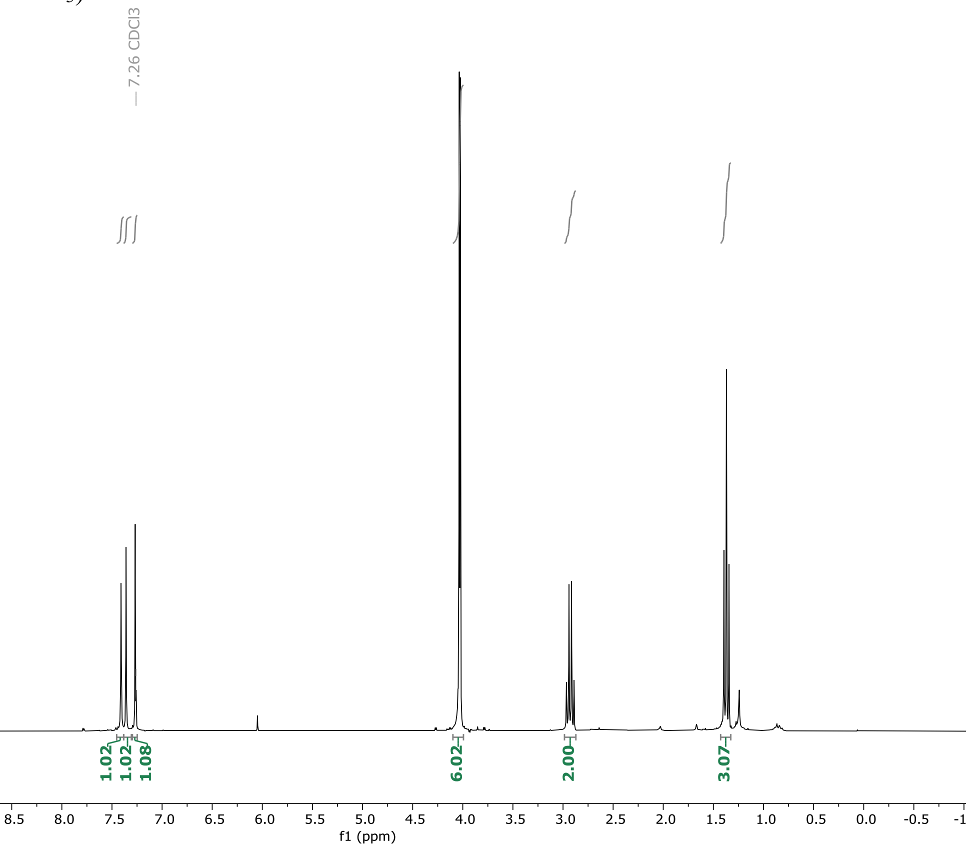

^{13}C NMR (101 MHz, $CDCl_3$) of **6**

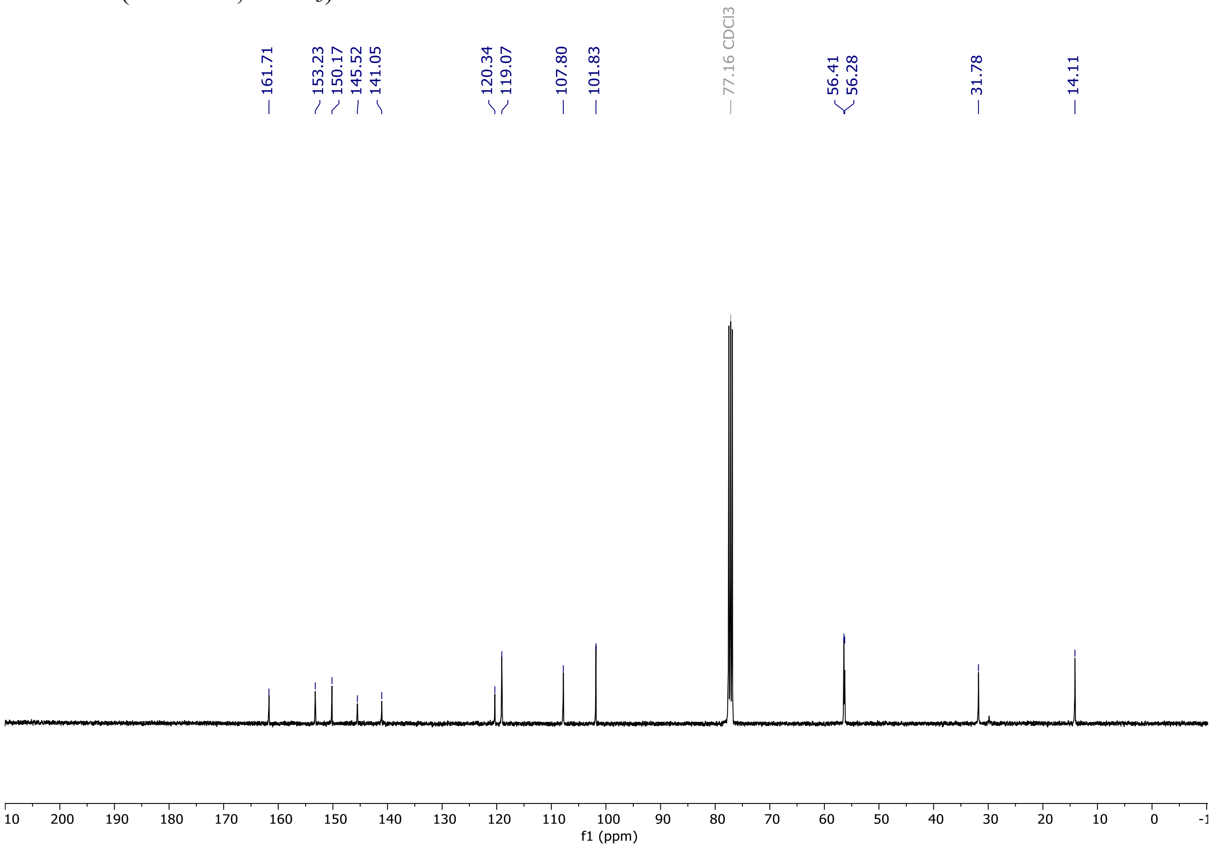

^{1}H NMR (400 MHz, $CDCl_3$) of **7**

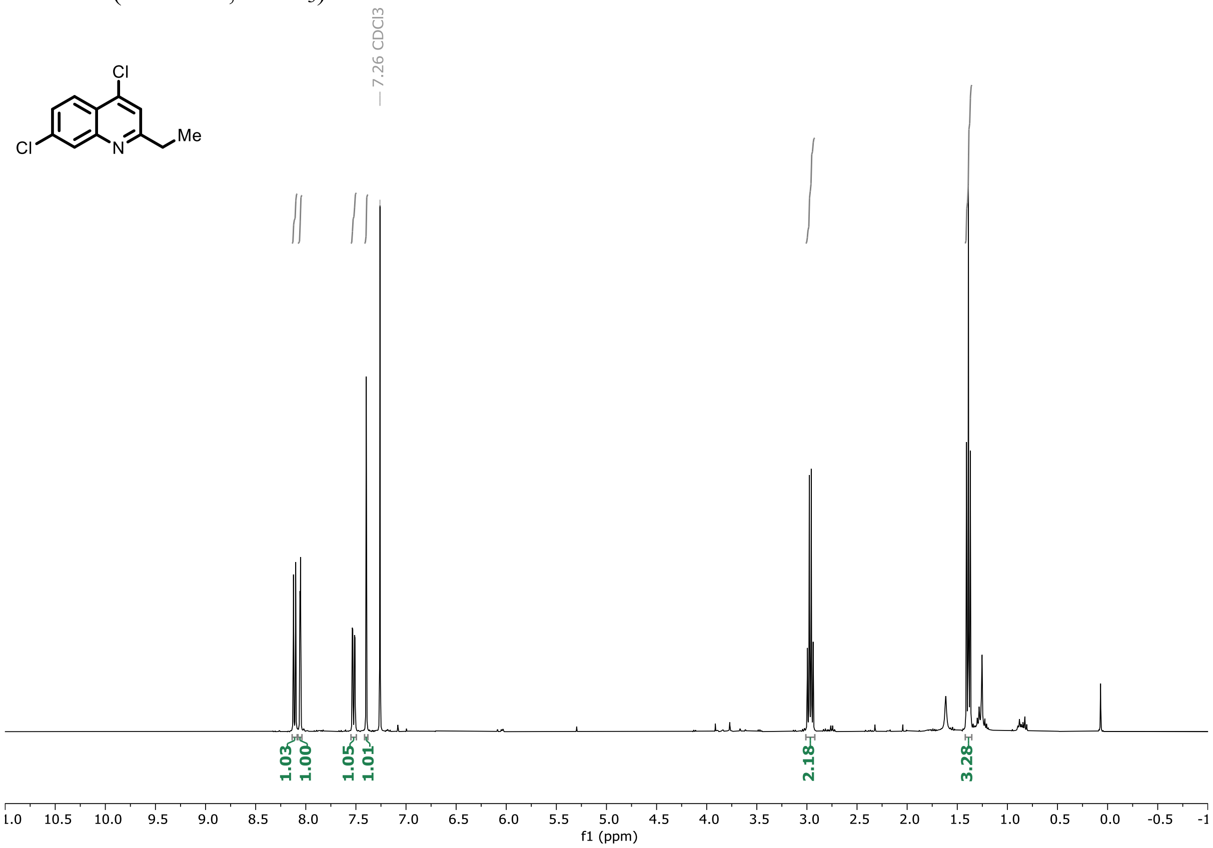

^{13}C NMR (101 MHz, $CDCl_3$) of **7**

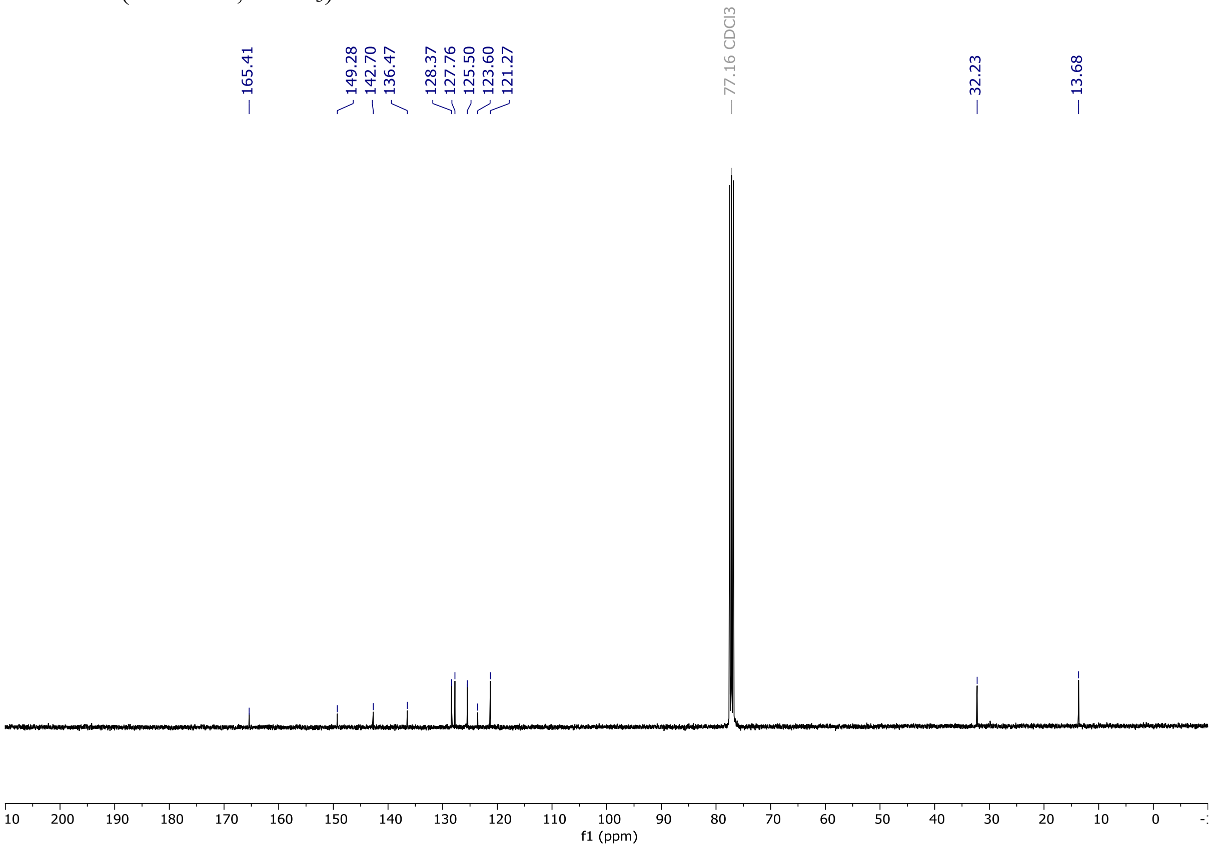

^{1}H NMR (400 MHz, $CDCl_3$) of **8**

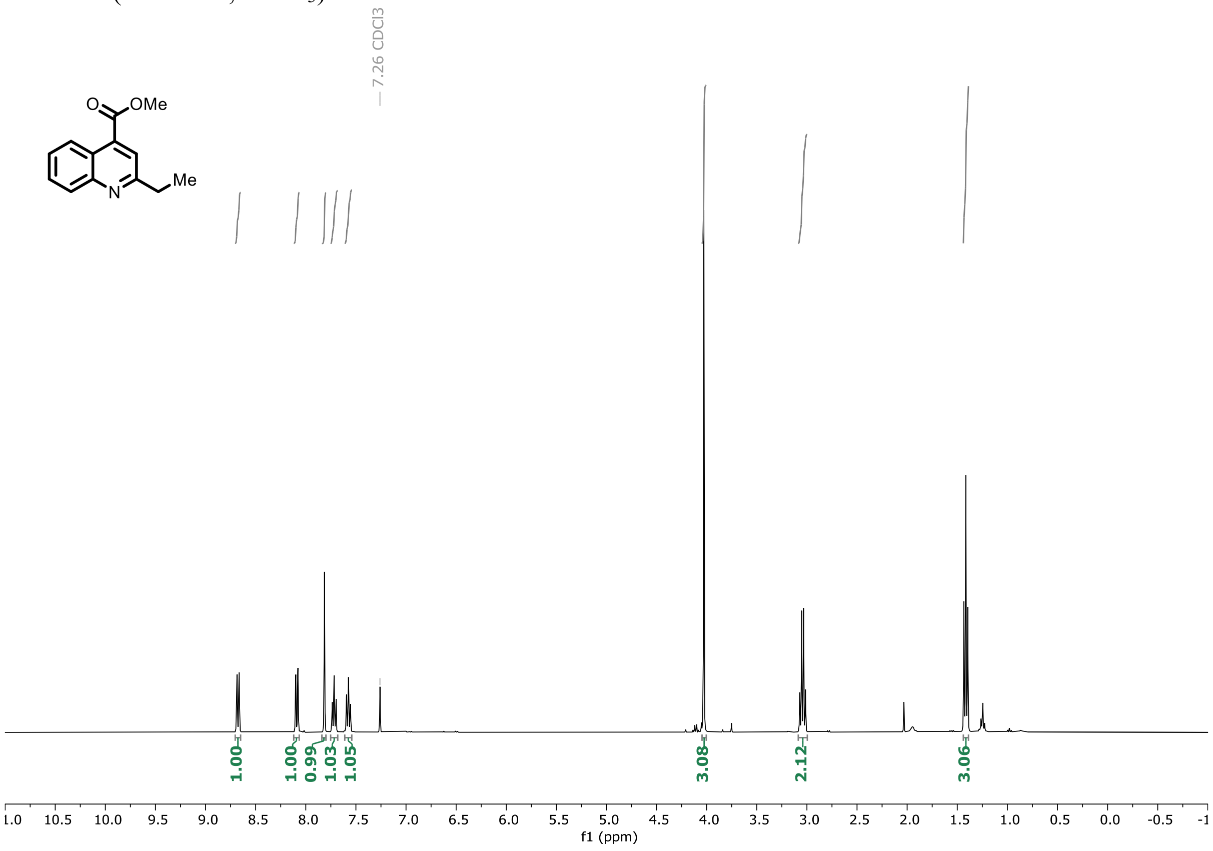

^{13}C NMR (101 MHz, $CDCl_3$) of **8**

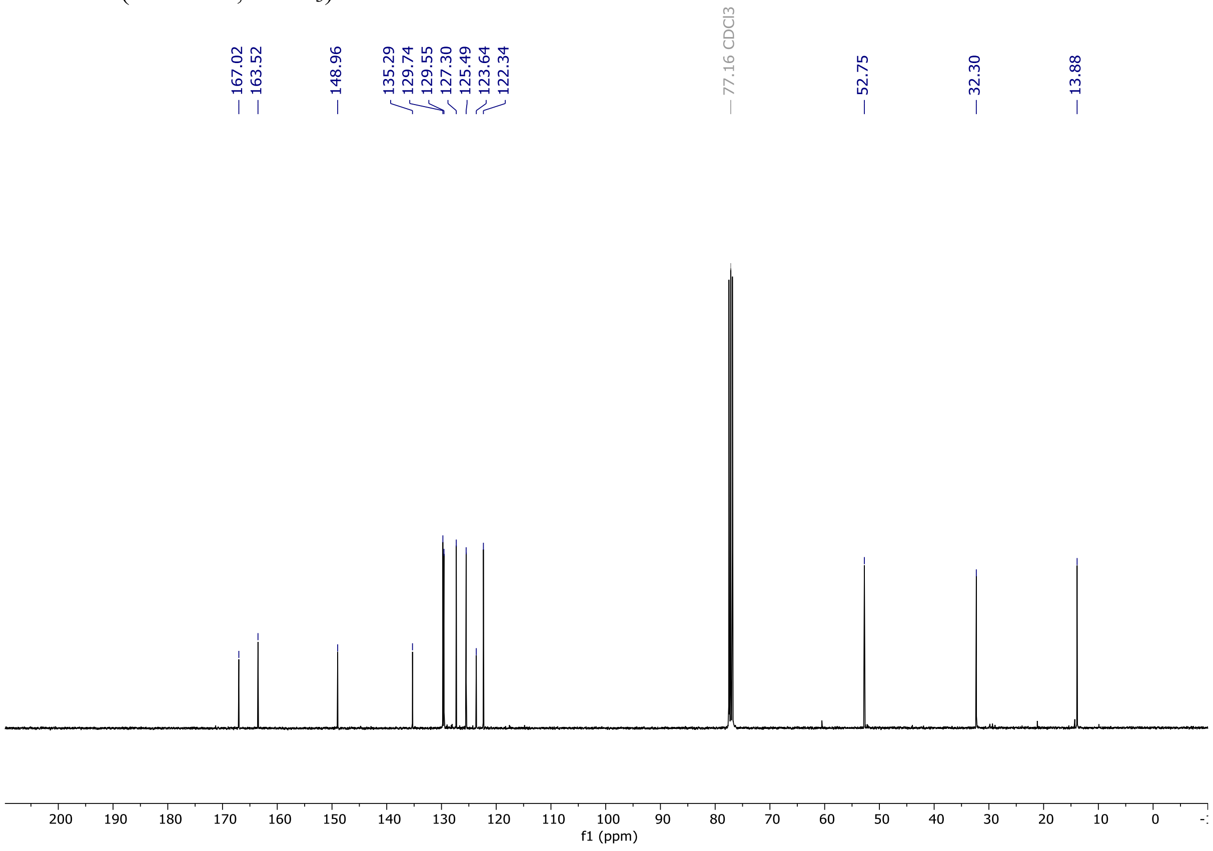

^{1}H NMR (400 MHz, $CDCl_3$) of **9**

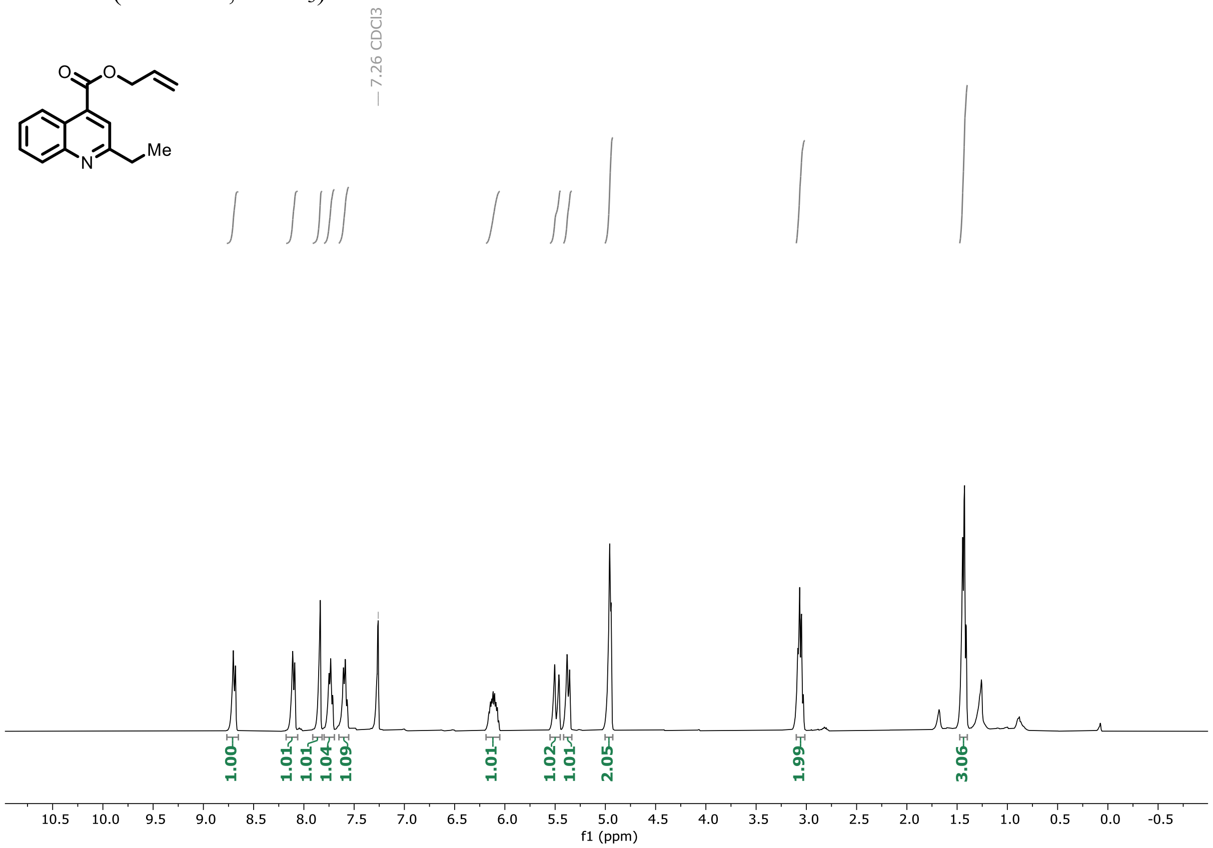

^{13}C NMR (101 MHz, $CDCl_3$) of **9**

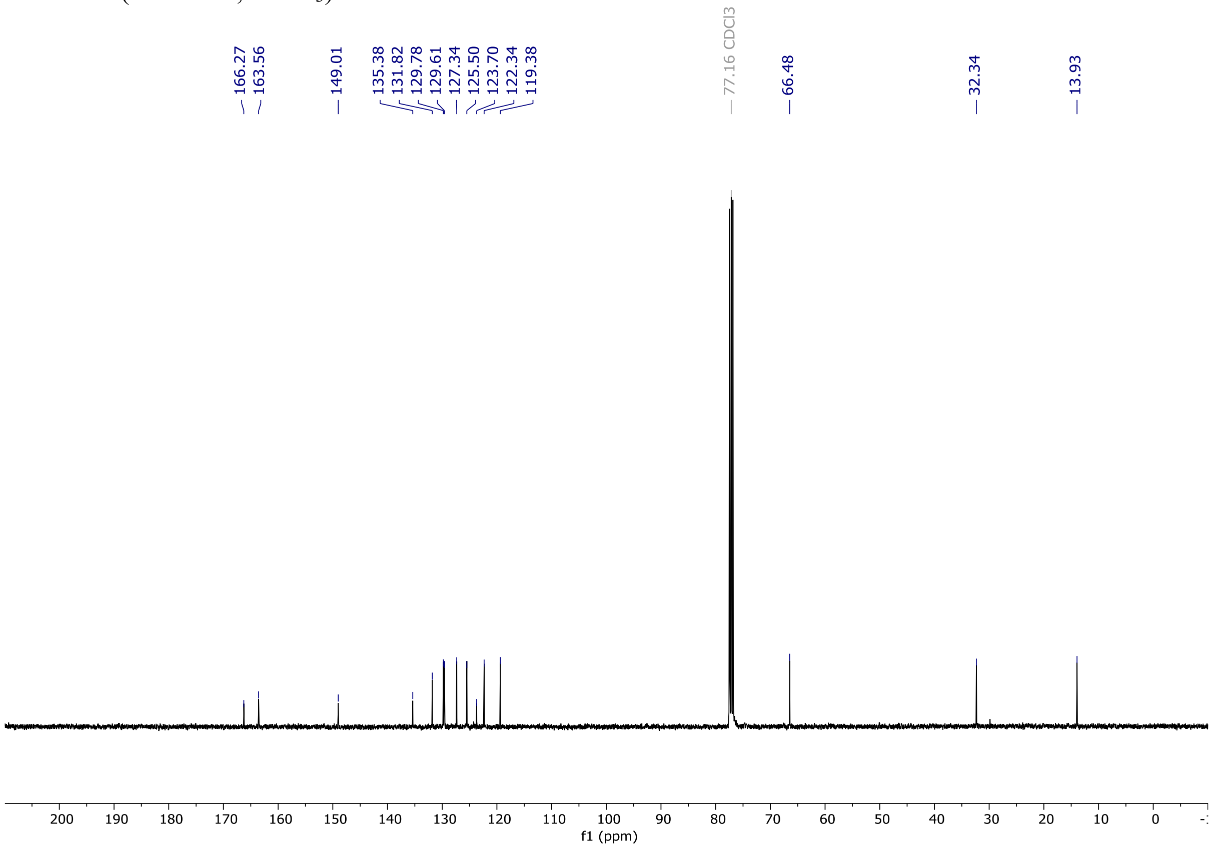

^{1}H NMR (400 MHz, $CDCl_3$) of **10**

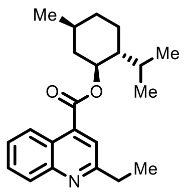

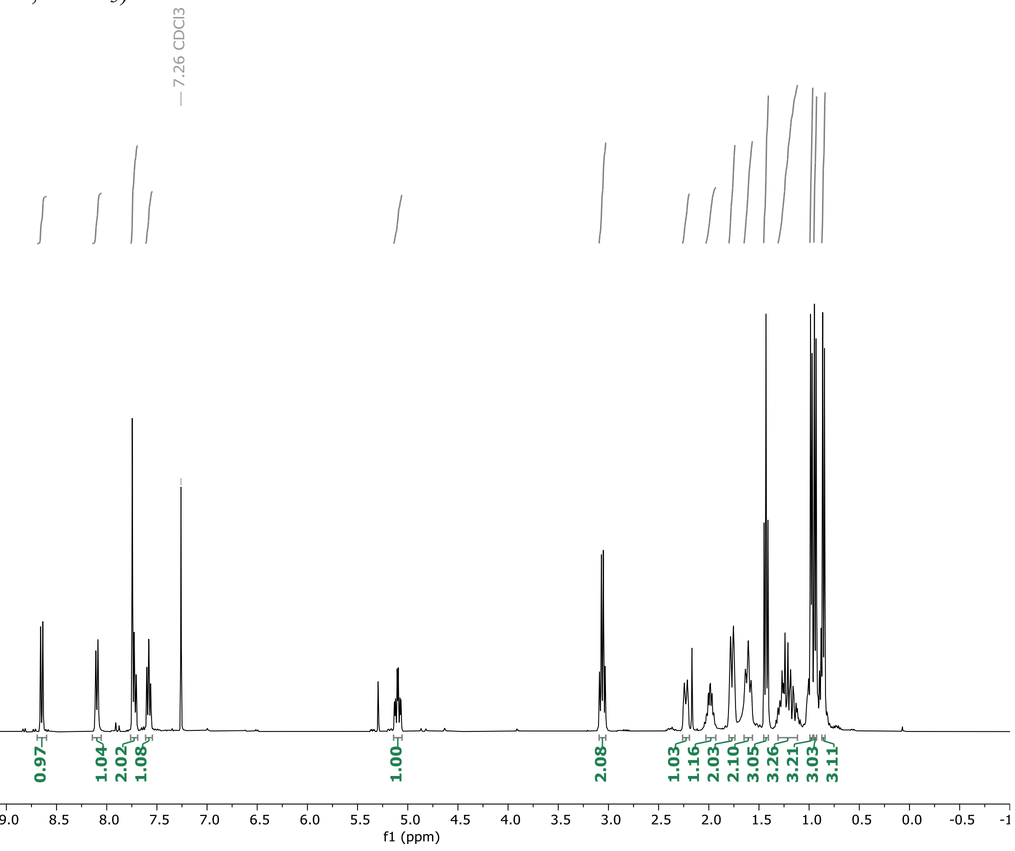

^{13}C NMR (101 MHz, $CDCl_3$) of **10**

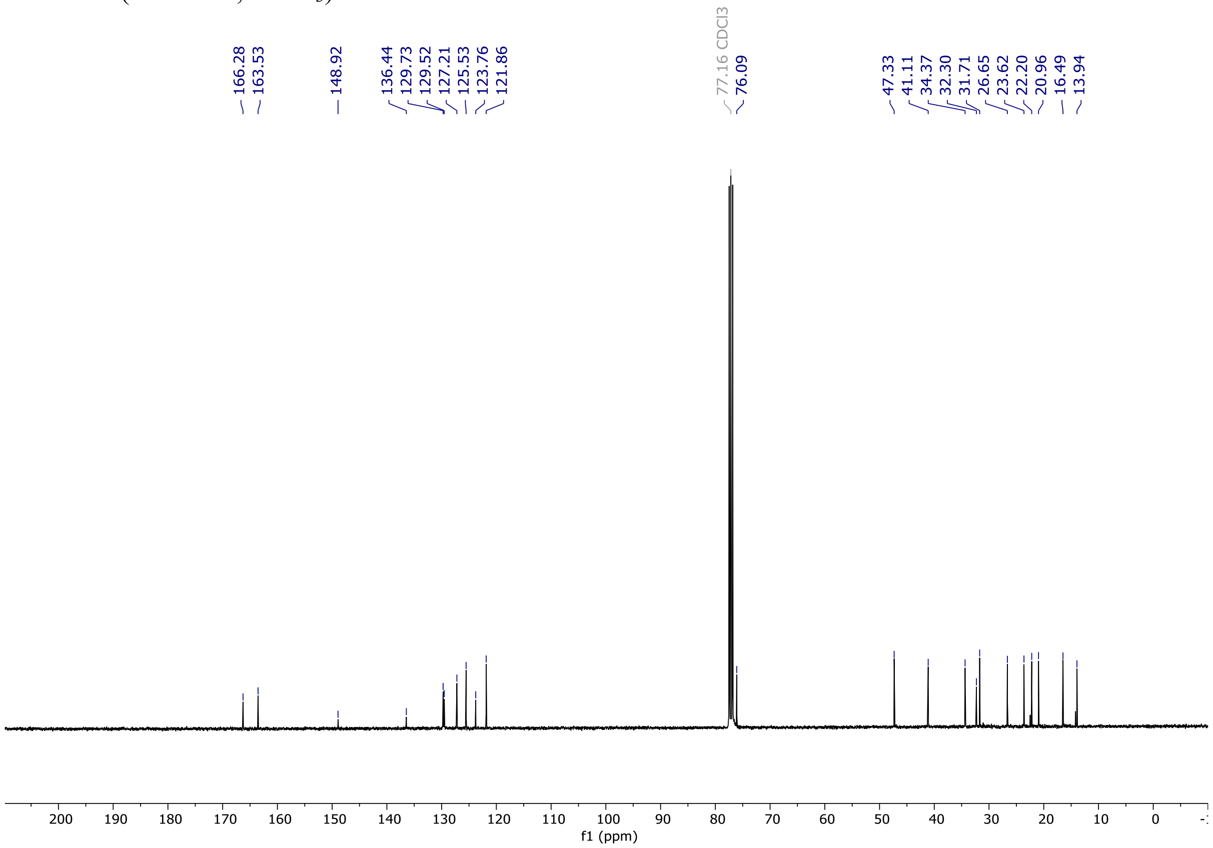

^{1}H NMR (400 MHz, $CDCl_3$) of **11**

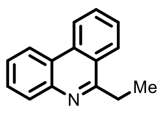

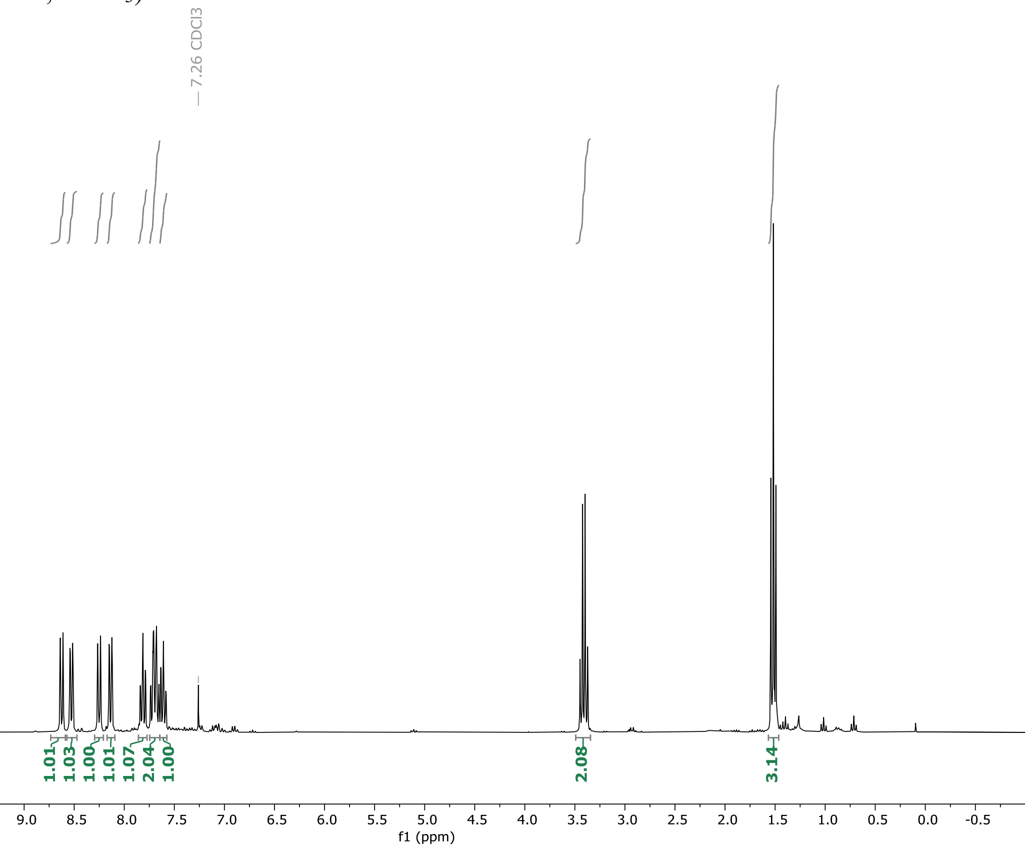

^{13}C NMR (101 MHz, $CDCl_3$) of **11**

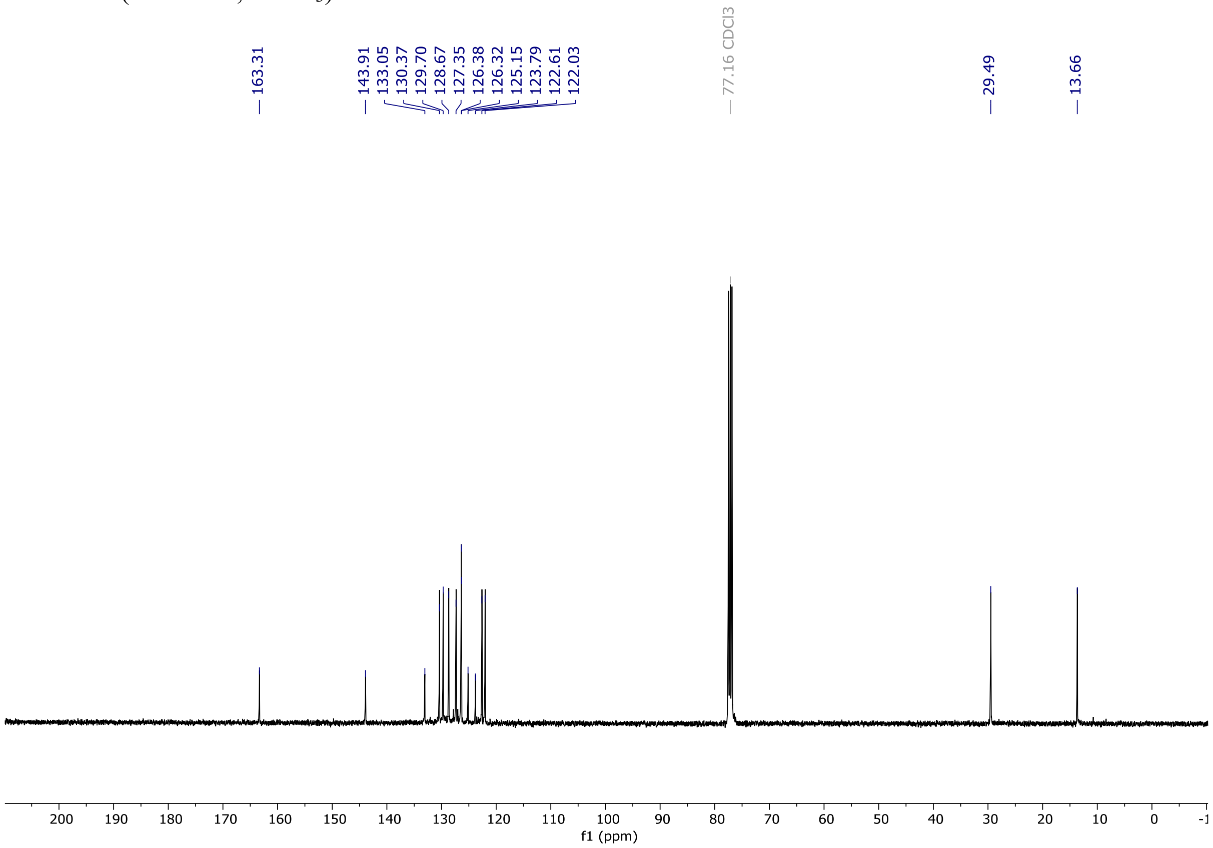

^{1}H NMR (400 MHz, $CDCl_3$) of **12**

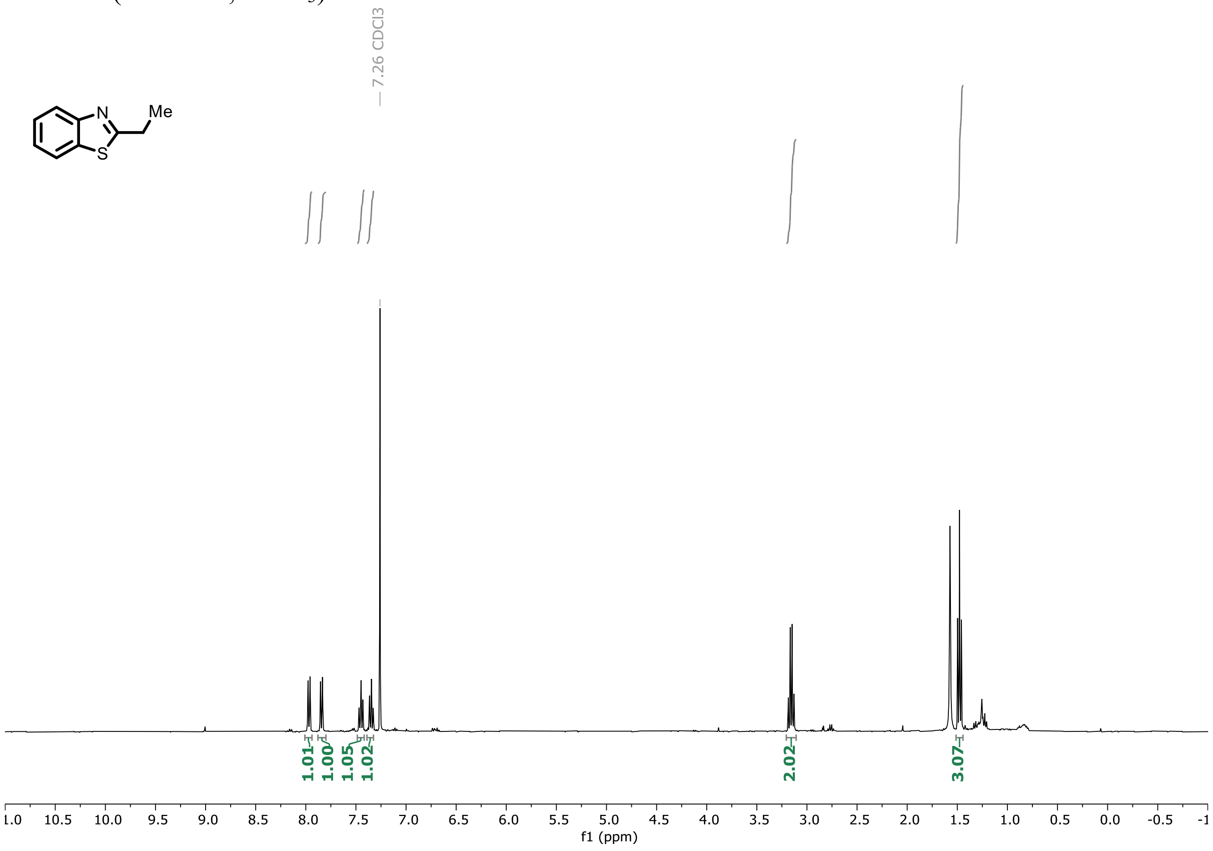

^{13}C NMR (101 MHz, $CDCl_3$) of 1**2**

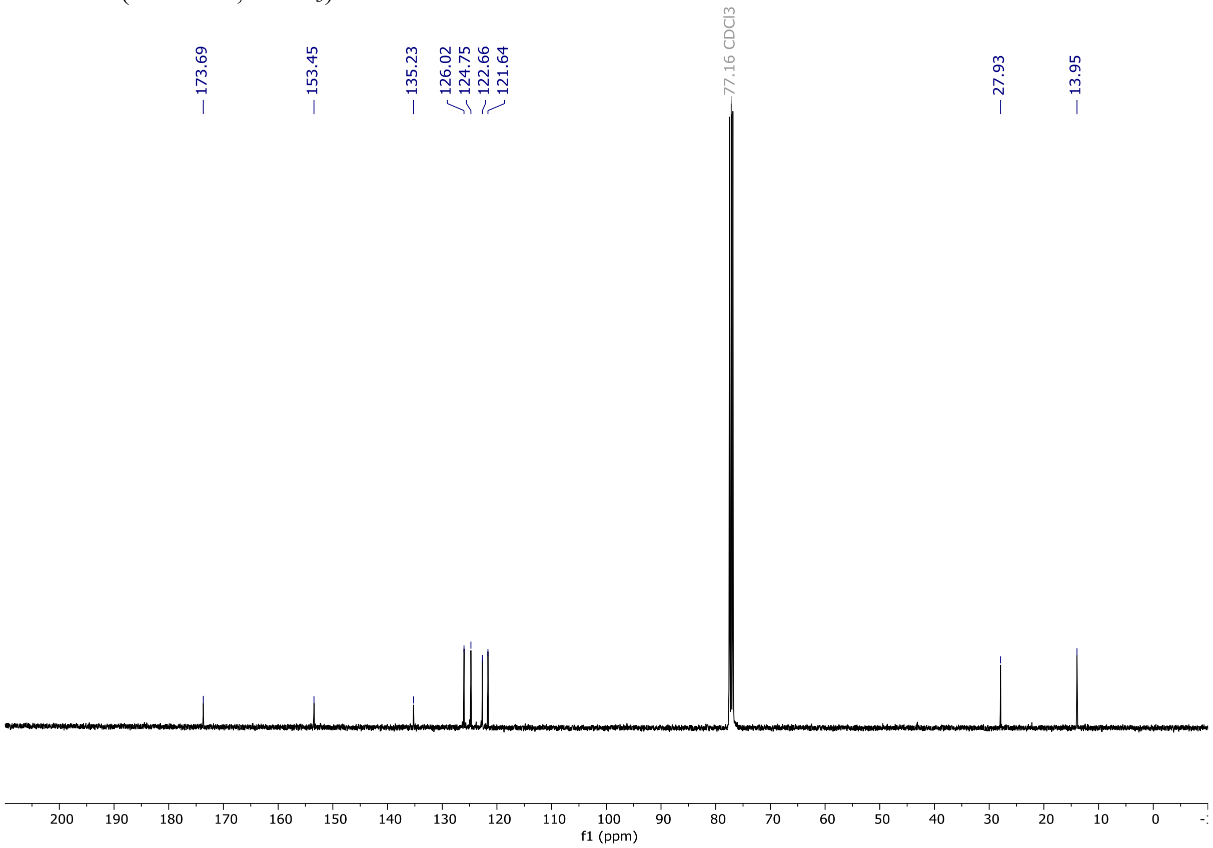

^{1}H NMR (400 MHz, $CDCl_3$) of **13**

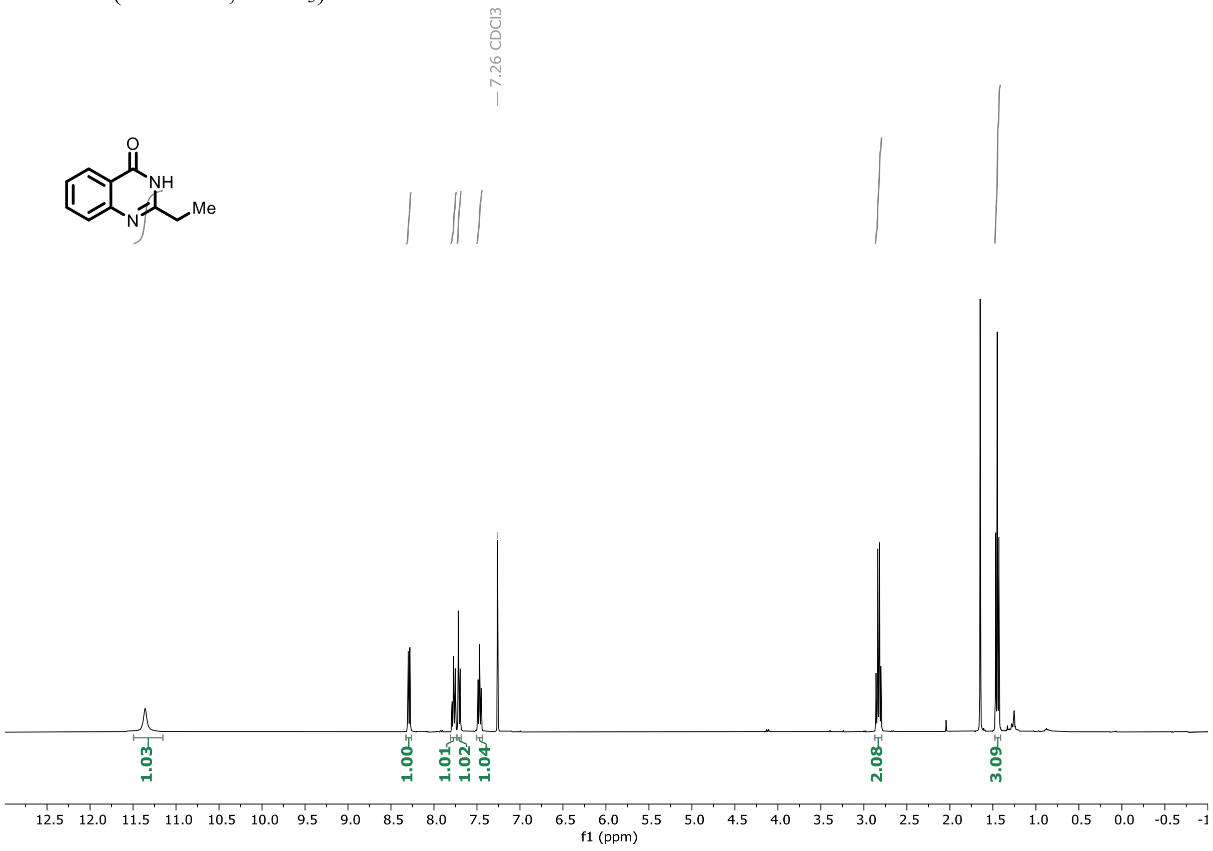

^{13}C NMR (101 MHz, $CDCl_3$) of **13**

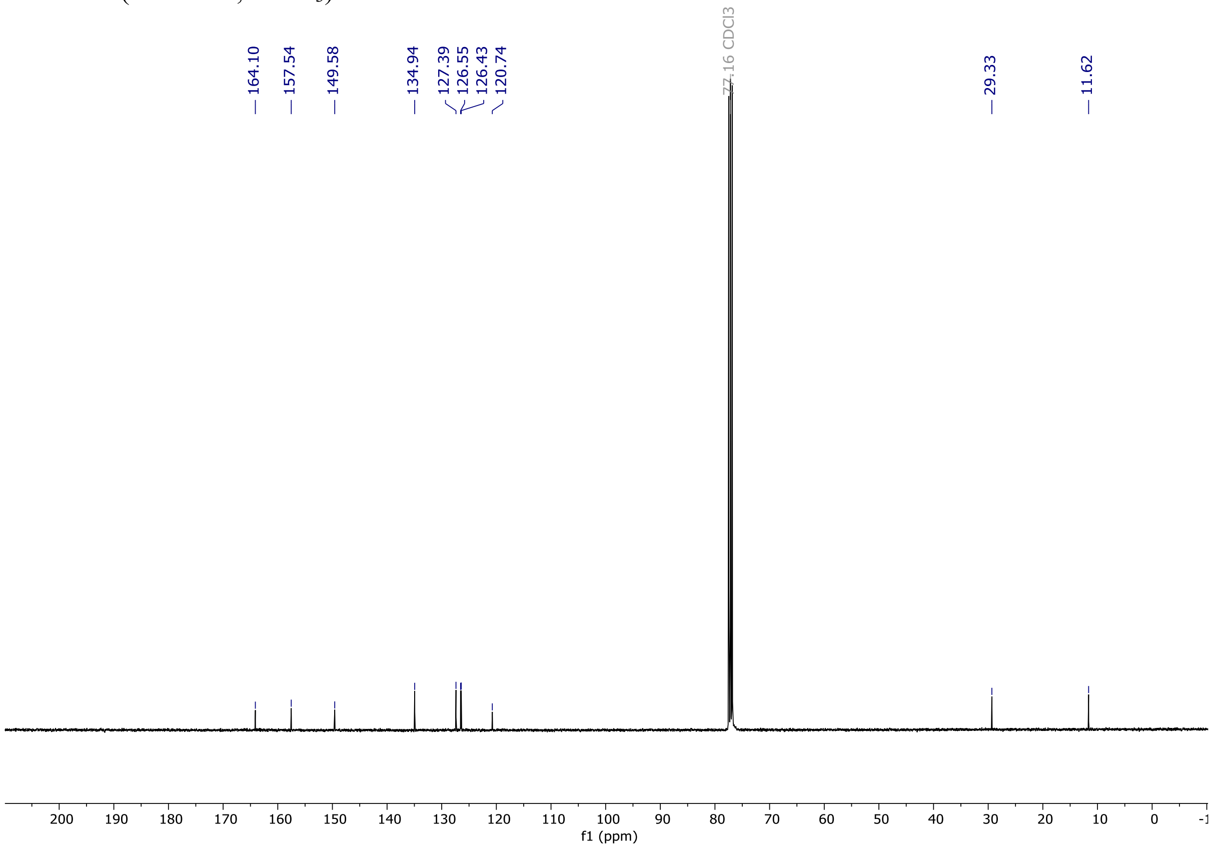

^{1}H NMR (400 MHz, $CDCl_3$) of **14 mono**

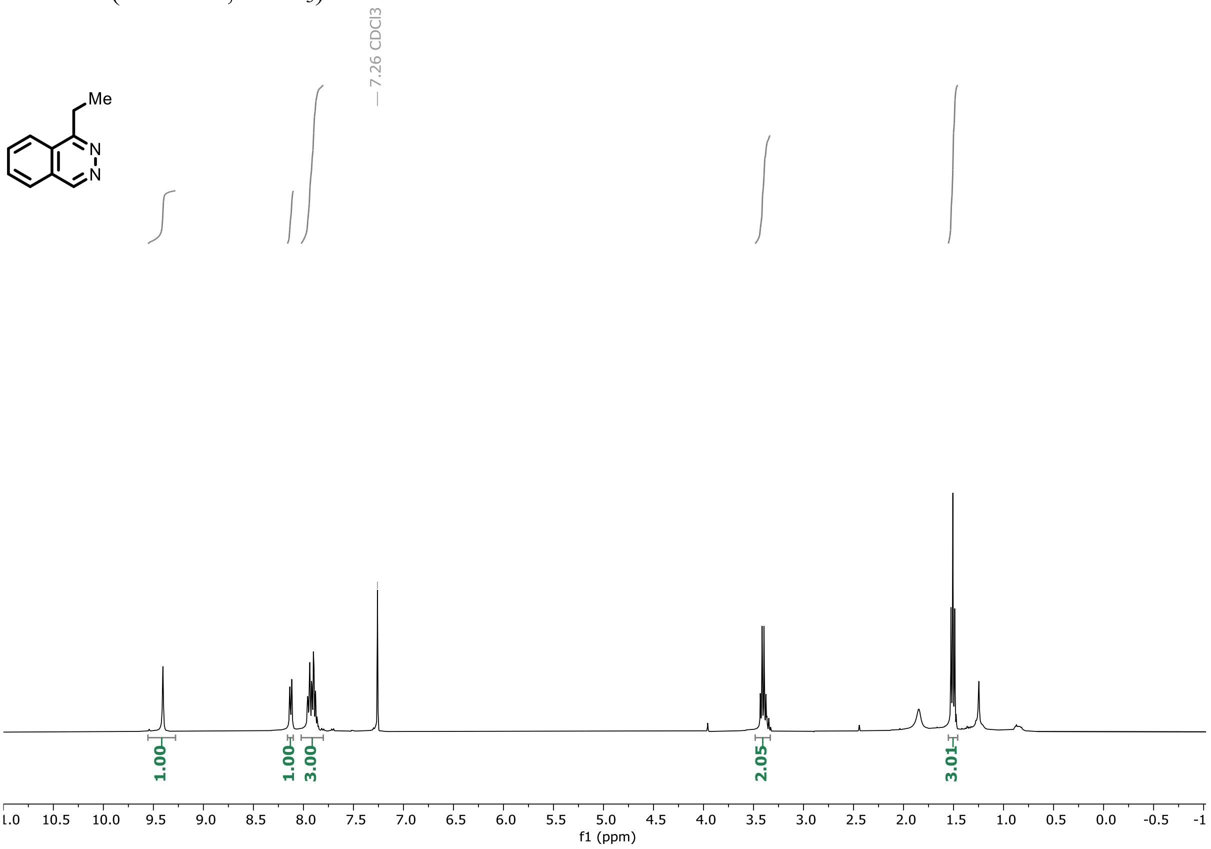

^{13}C NMR (101 MHz, $CDCl_3$) of **14 mono**

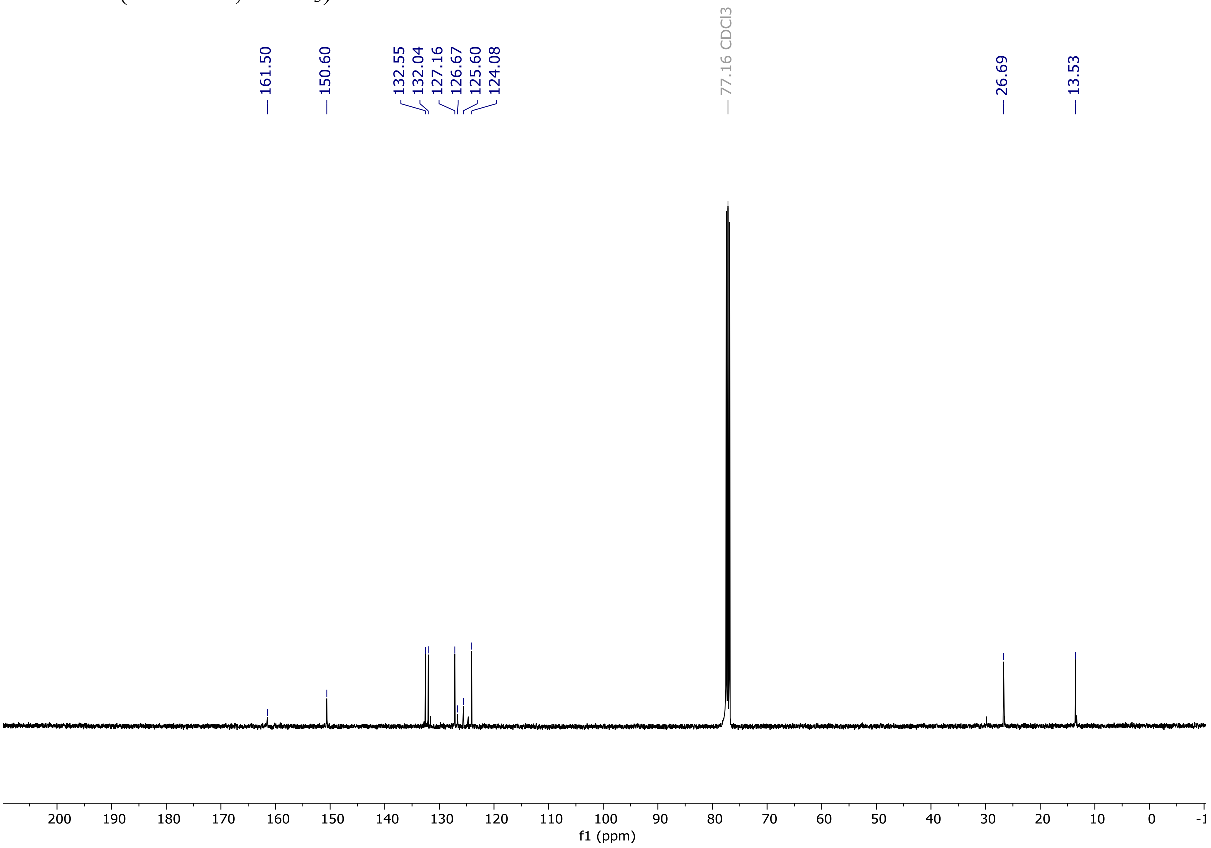

^{1}H NMR (400 MHz, $CDCl_3$) of **14 bis**

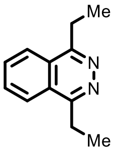

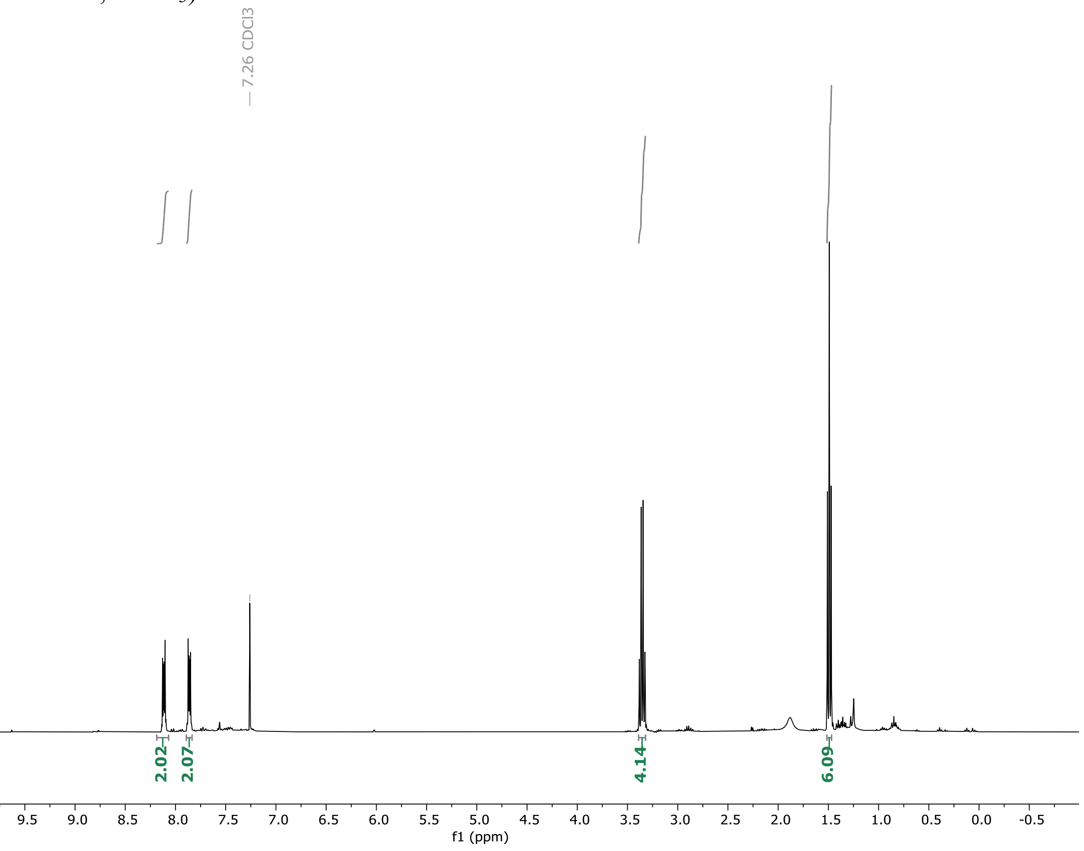

^{13}C NMR (101 MHz, $CDCl_3$) of **14 bis**

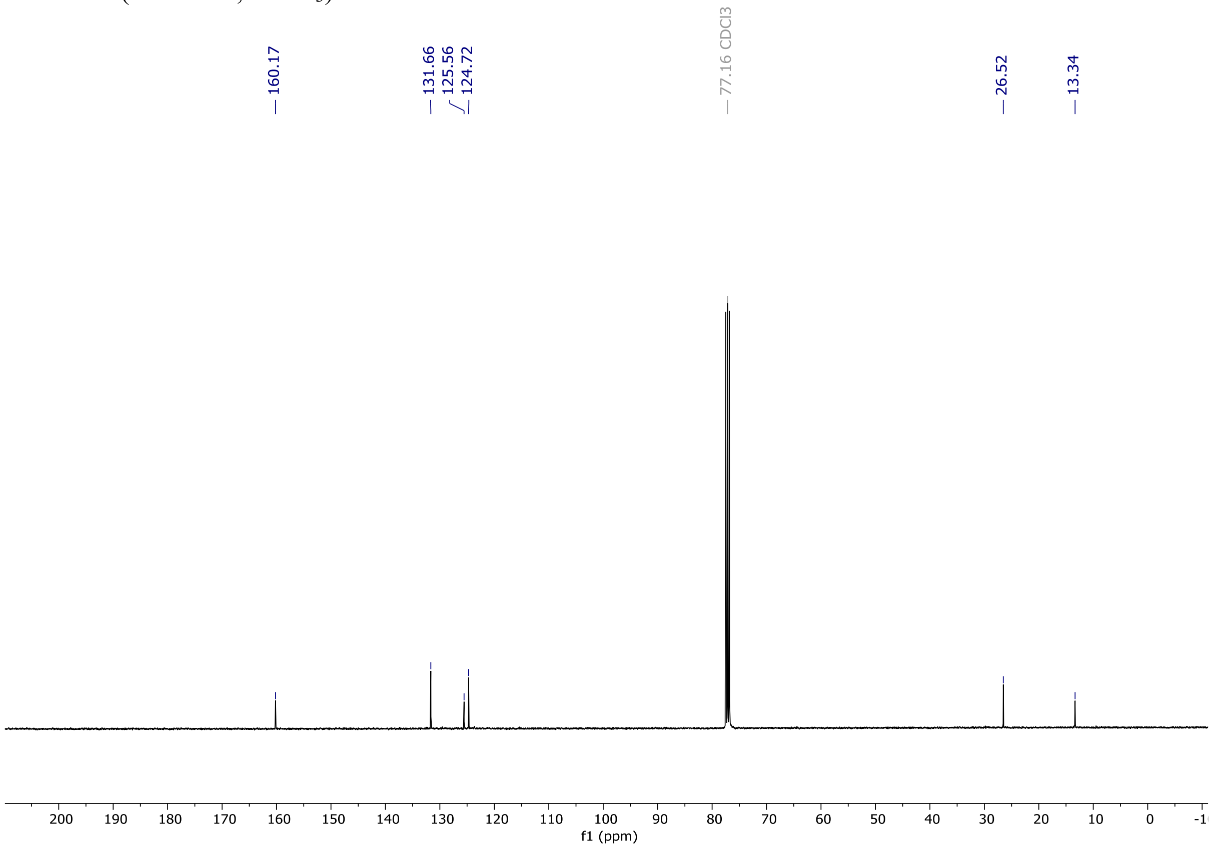

^{1}H NMR (400 MHz, $CDCl_3$) of **15**

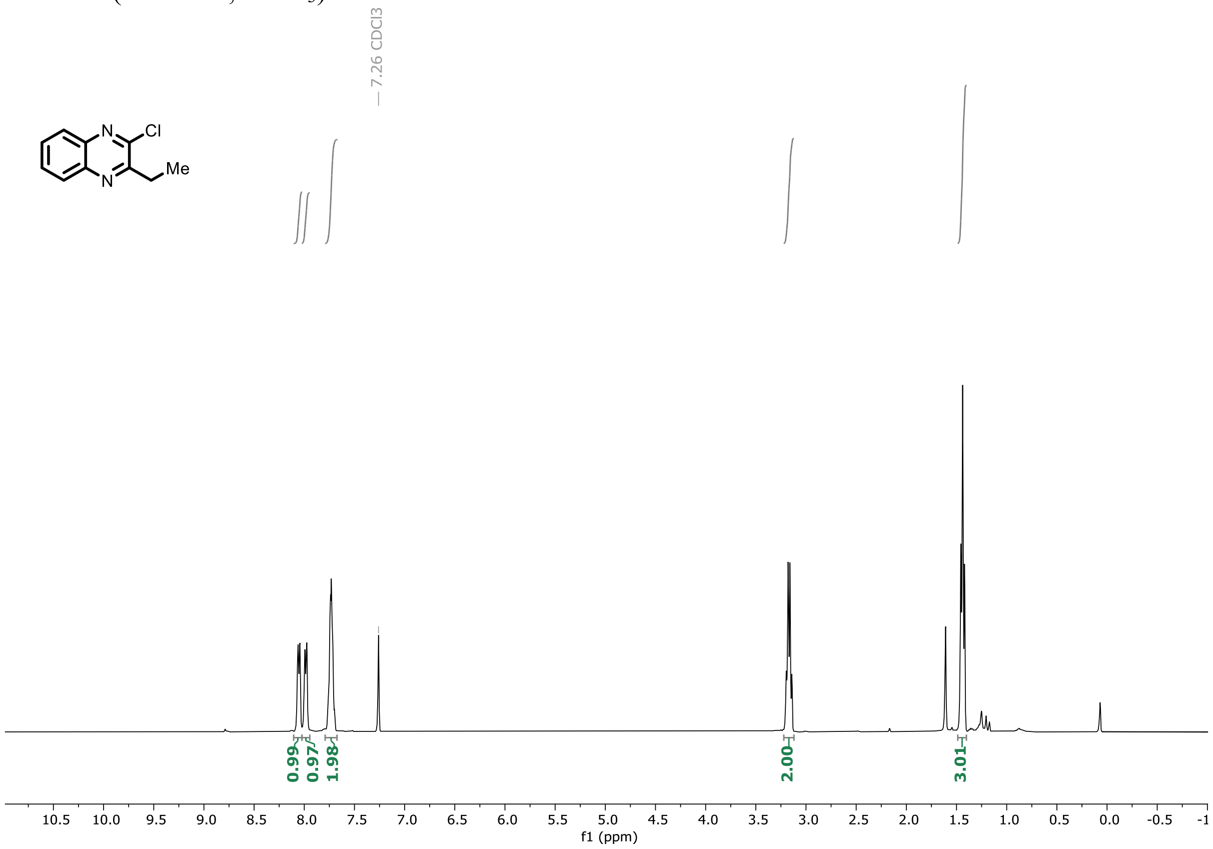

^{13}C NMR (101 MHz, $CDCl_3$) of **15**

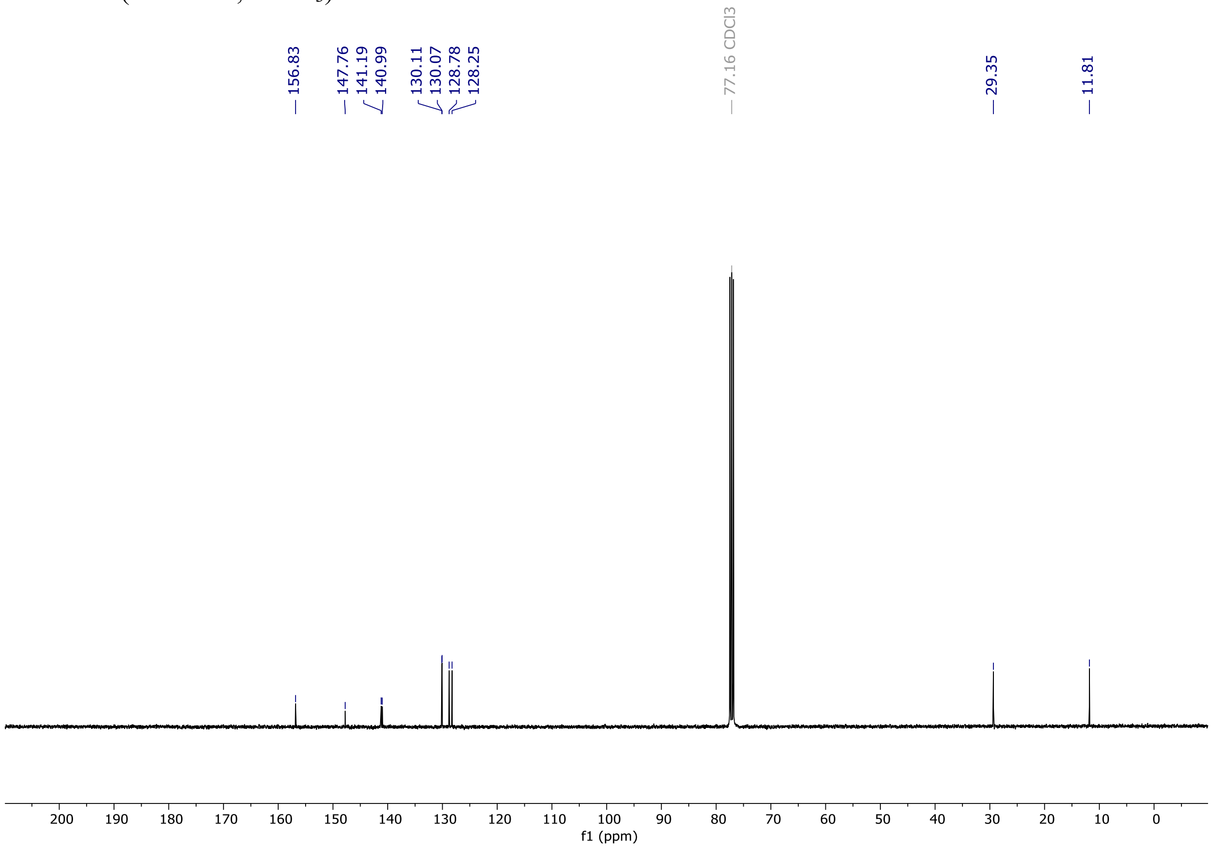

^{1}H NMR (400 MHz, $CDCl_3$) of **16**

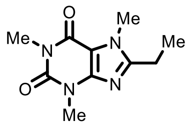

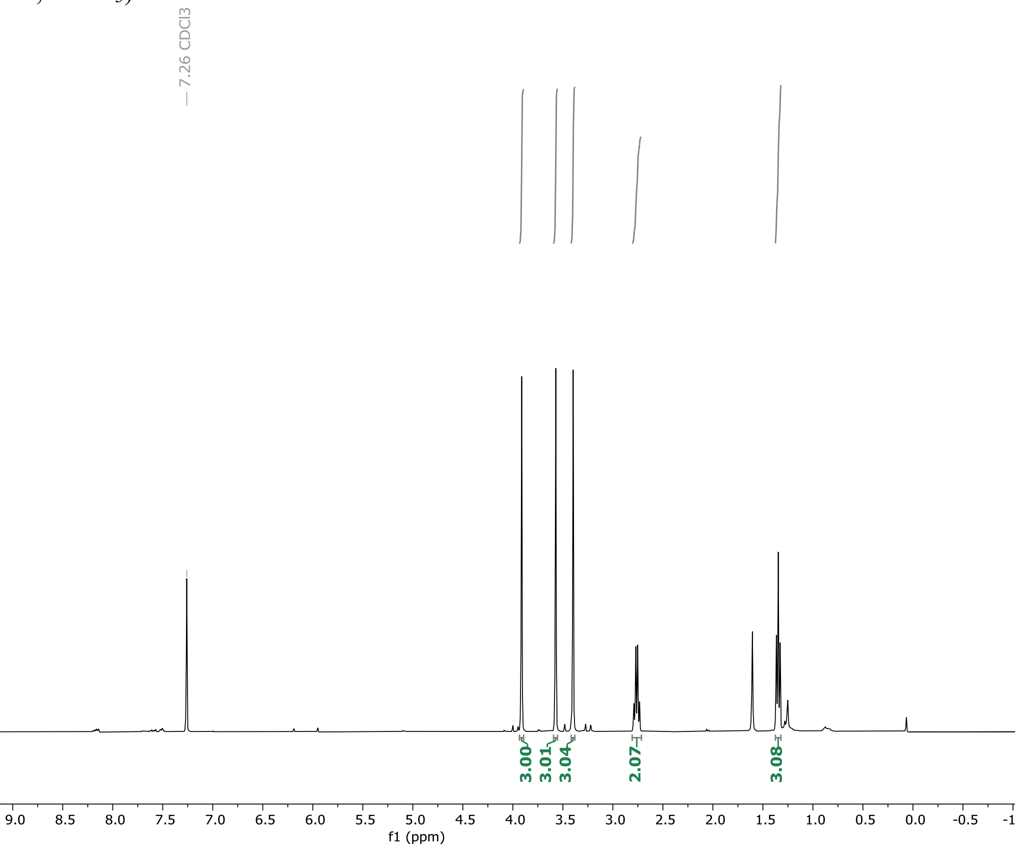

^{13}C NMR (101 MHz, $CDCl_3$) of **16**

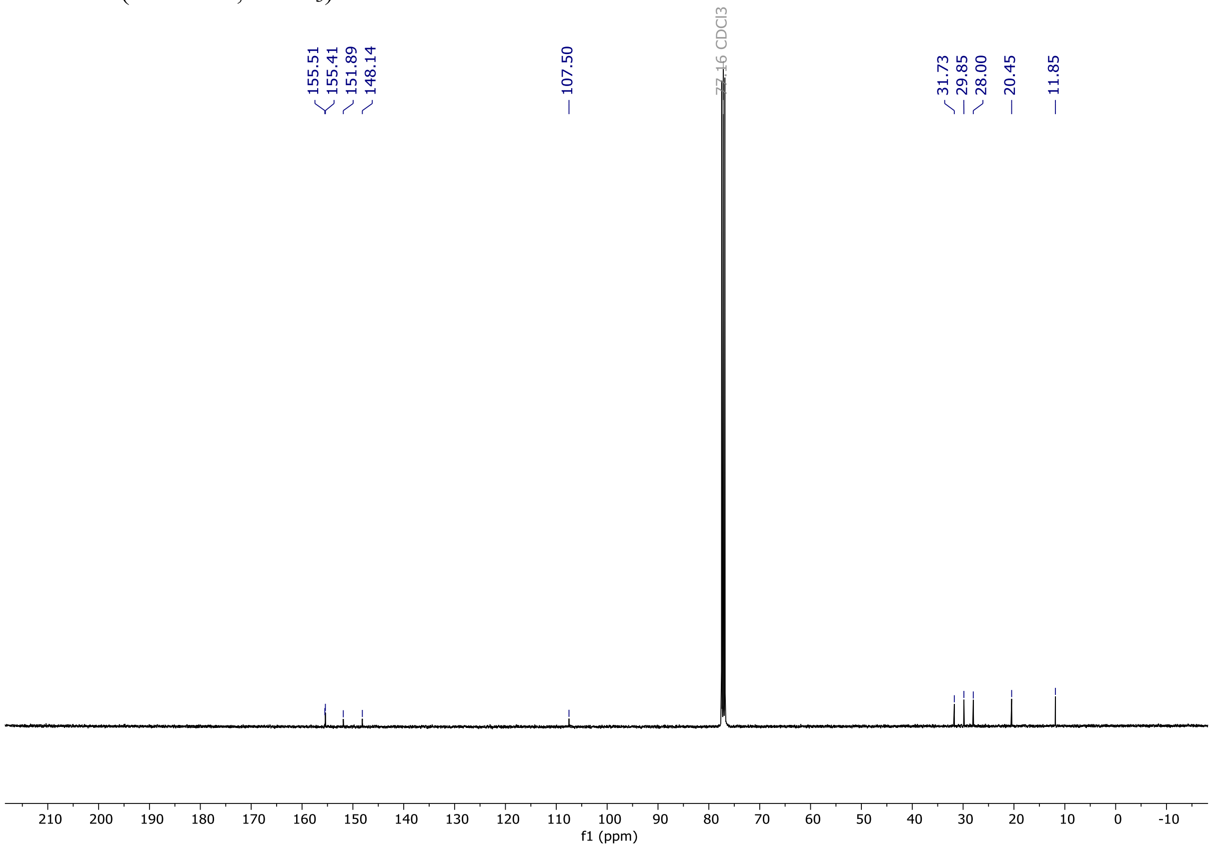

^{1}H NMR (400 MHz, $CDCl_3$) of **17**

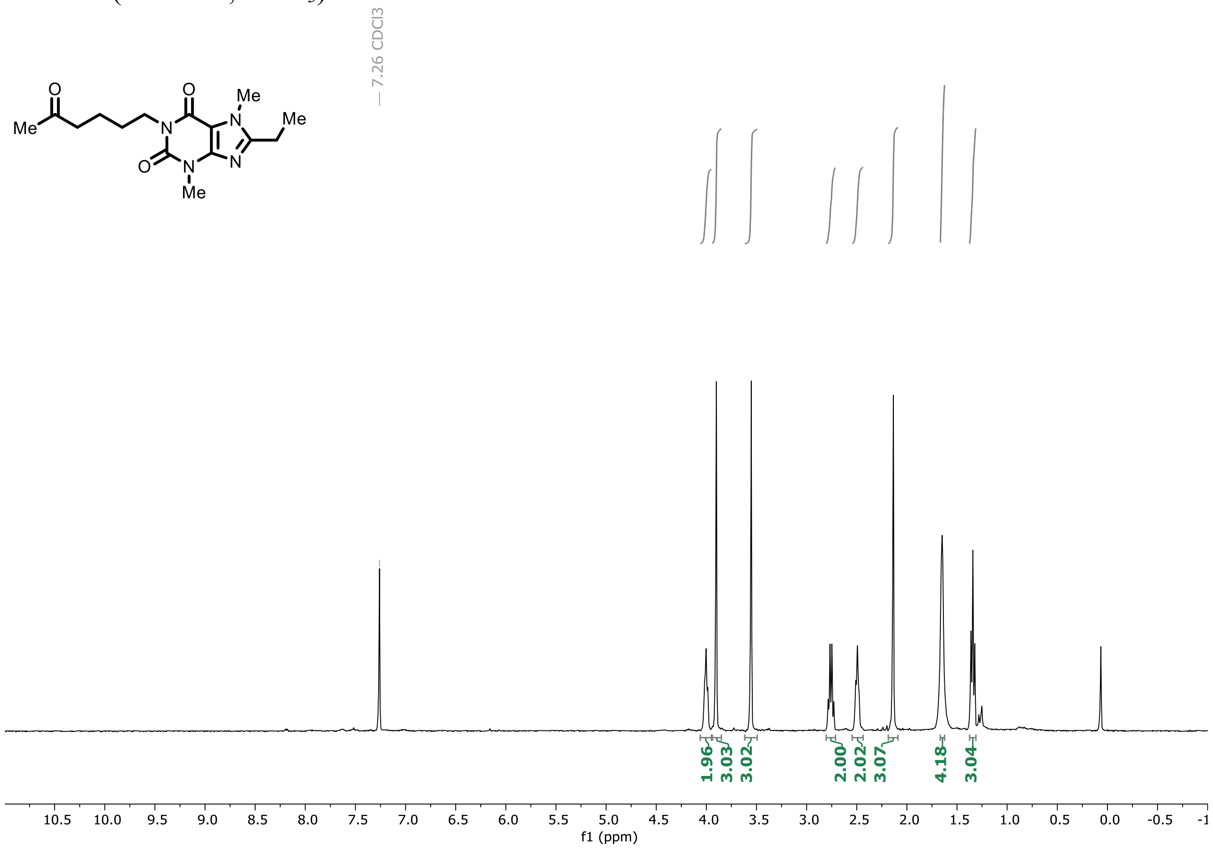

^{13}C NMR (101 MHz, $CDCl_3$) of **17**

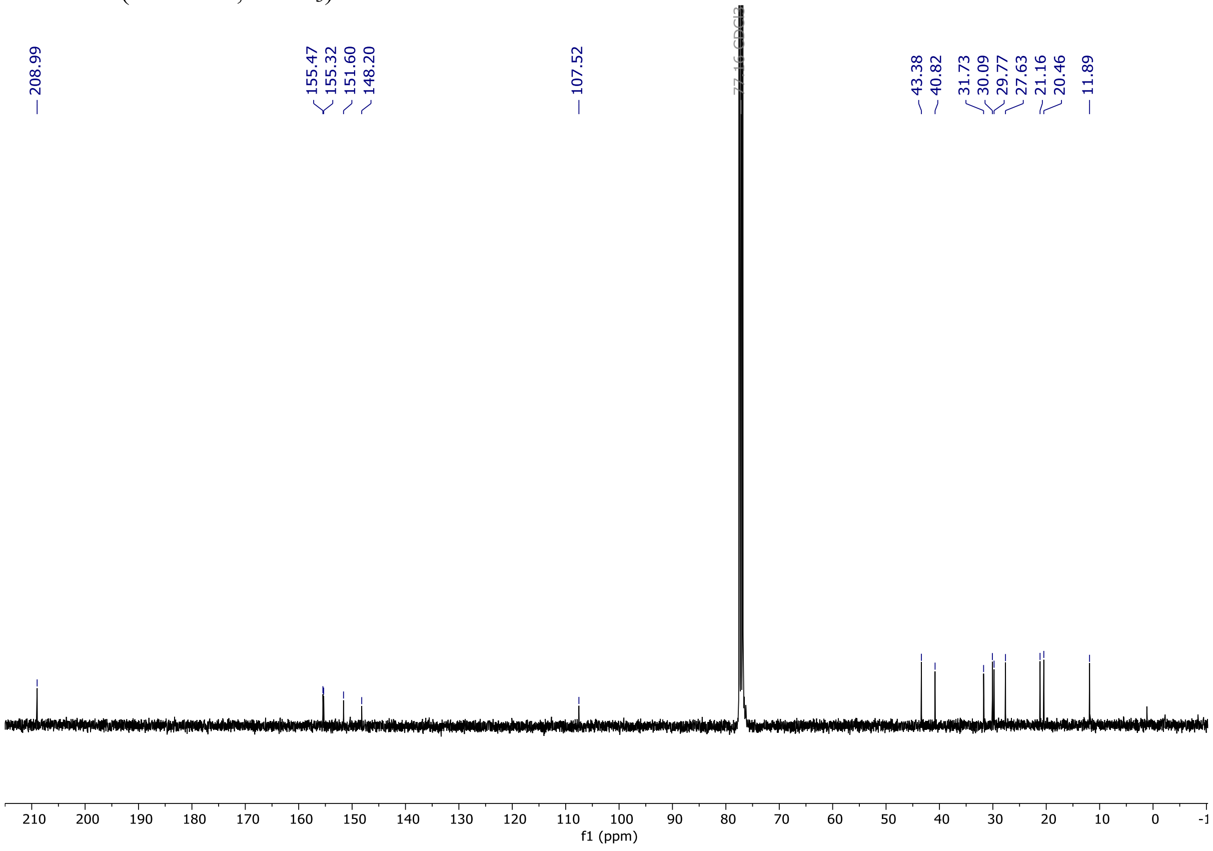

^{1}H NMR (400 MHz, $CDCl_3$) of **18**

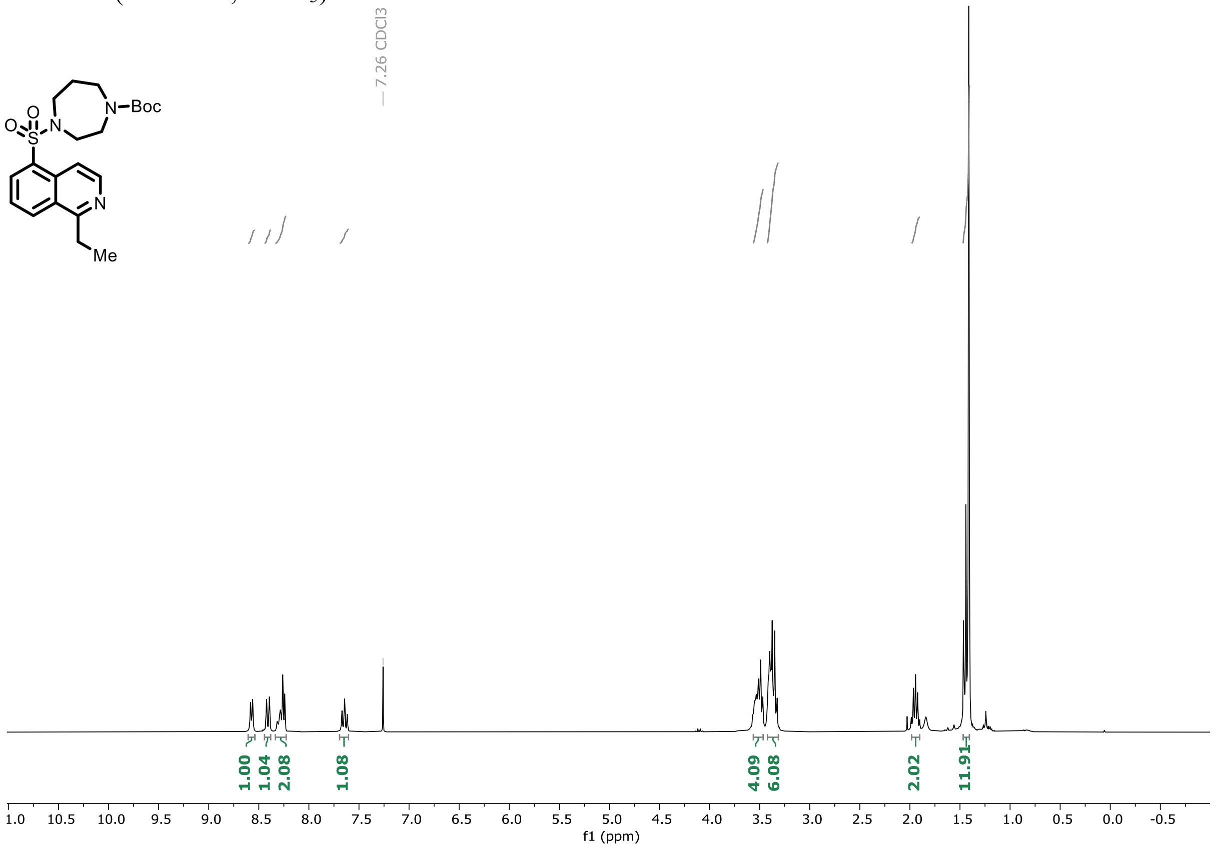

^{13}C NMR (101 MHz, $CDCl_3$) of **18**

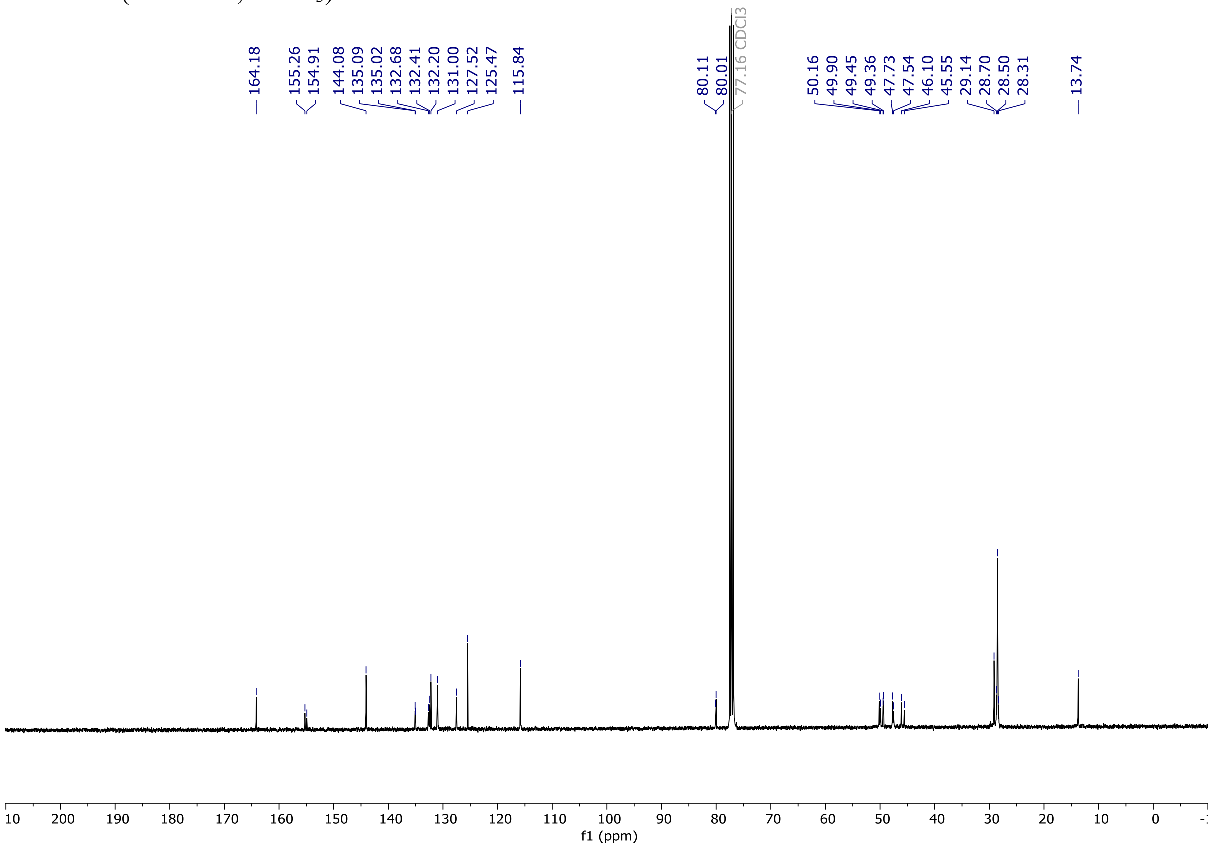

^{1}H NMR (400 MHz, $CDCl_3$) of **19**

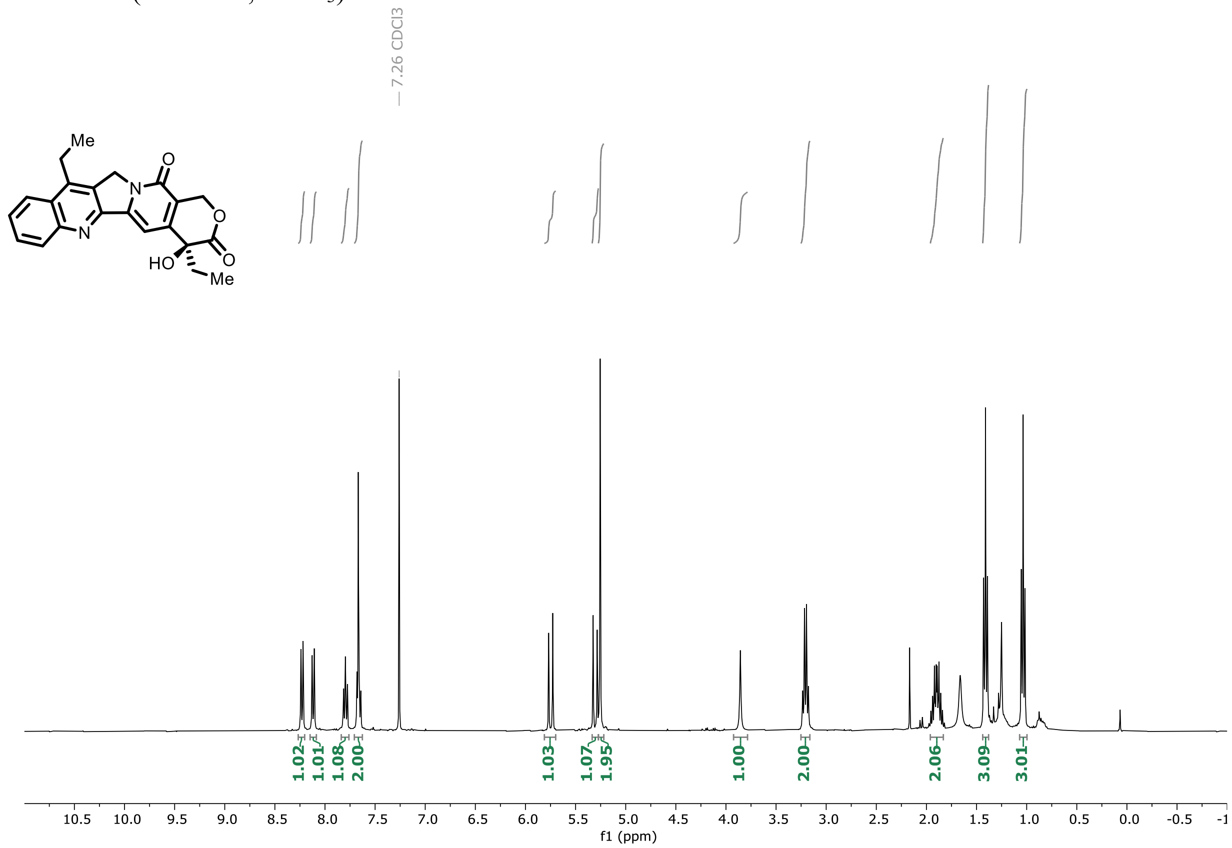

^{13}C NMR (101 MHz, $CDCl_3$) of **19**

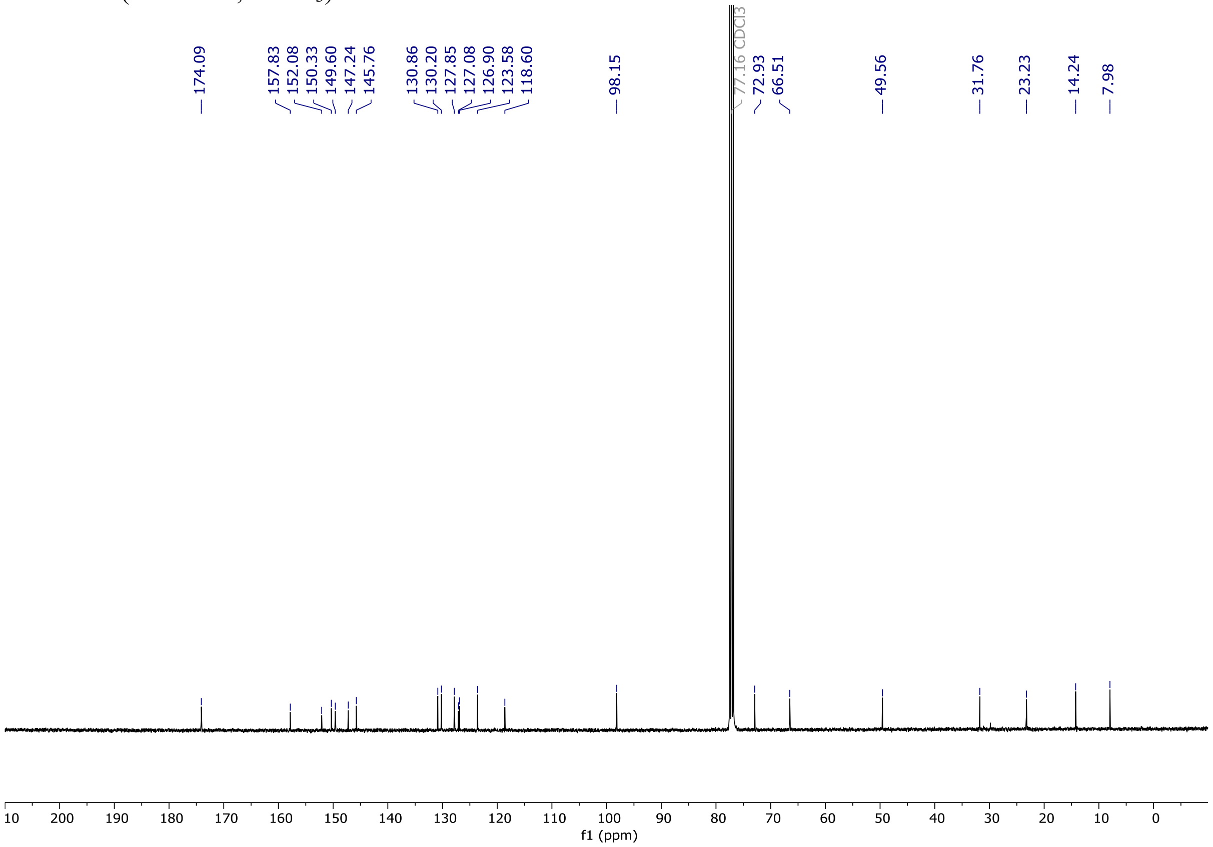

^{1}H NMR (400 MHz, $CDCl_3$) of **20**

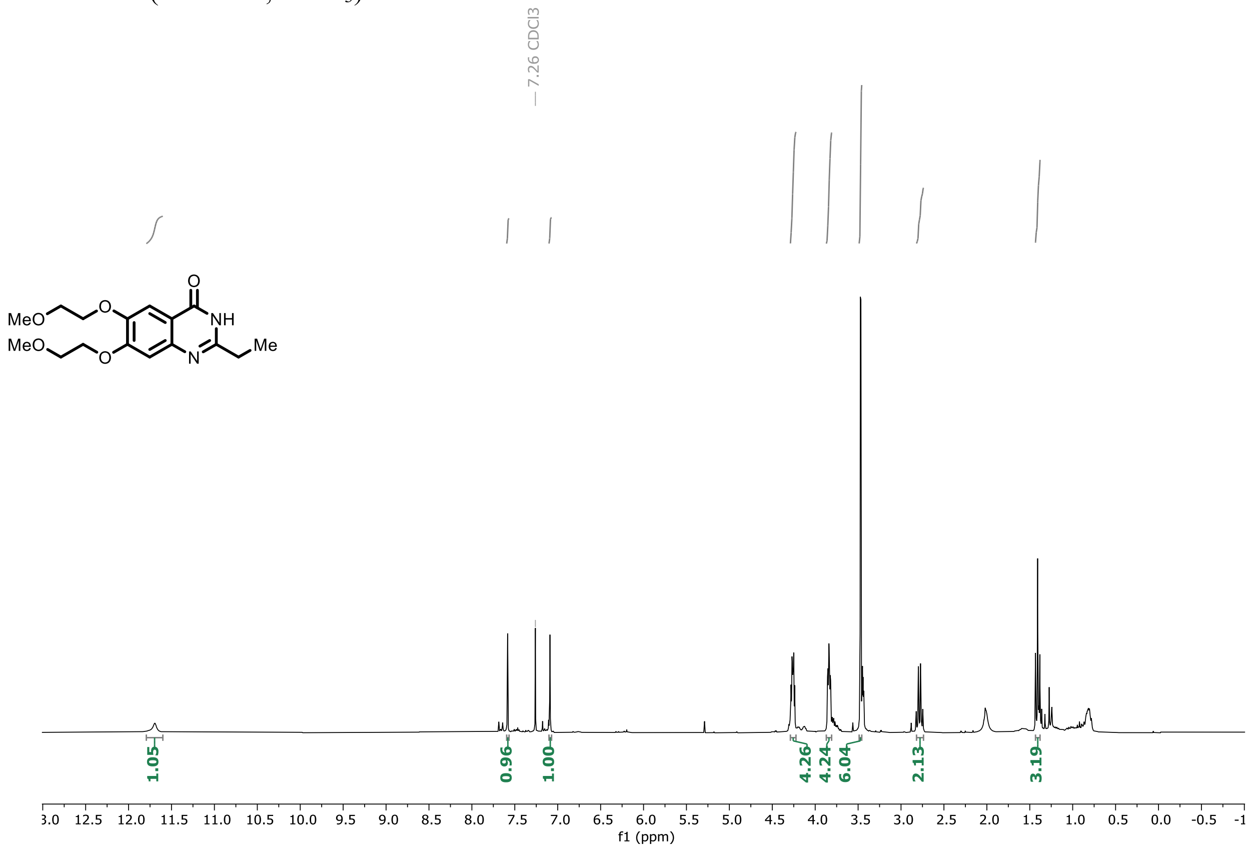

^{13}C NMR (101 MHz, $CDCl_3$) of **20**

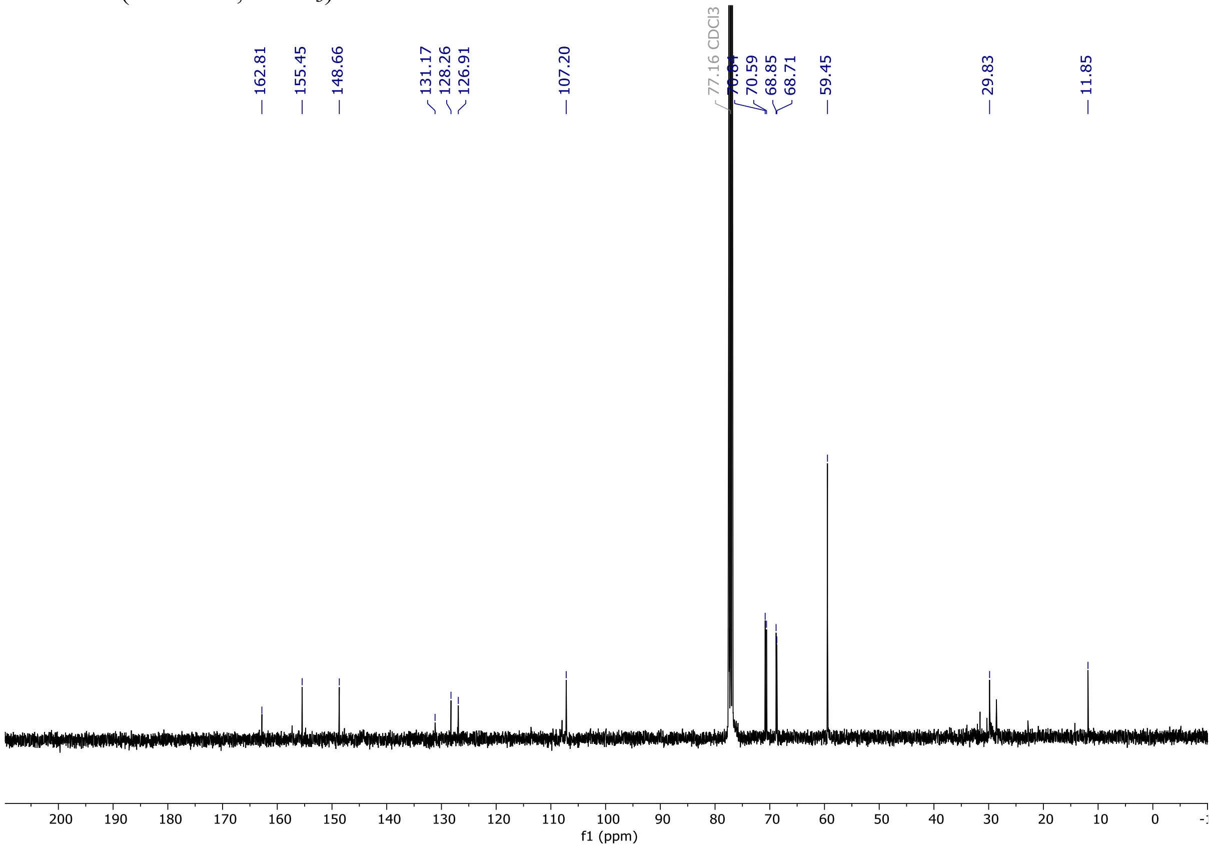

^{1}H NMR (400 MHz, $CDCl_3$) of **21**

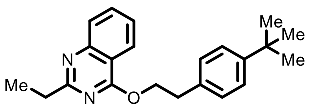

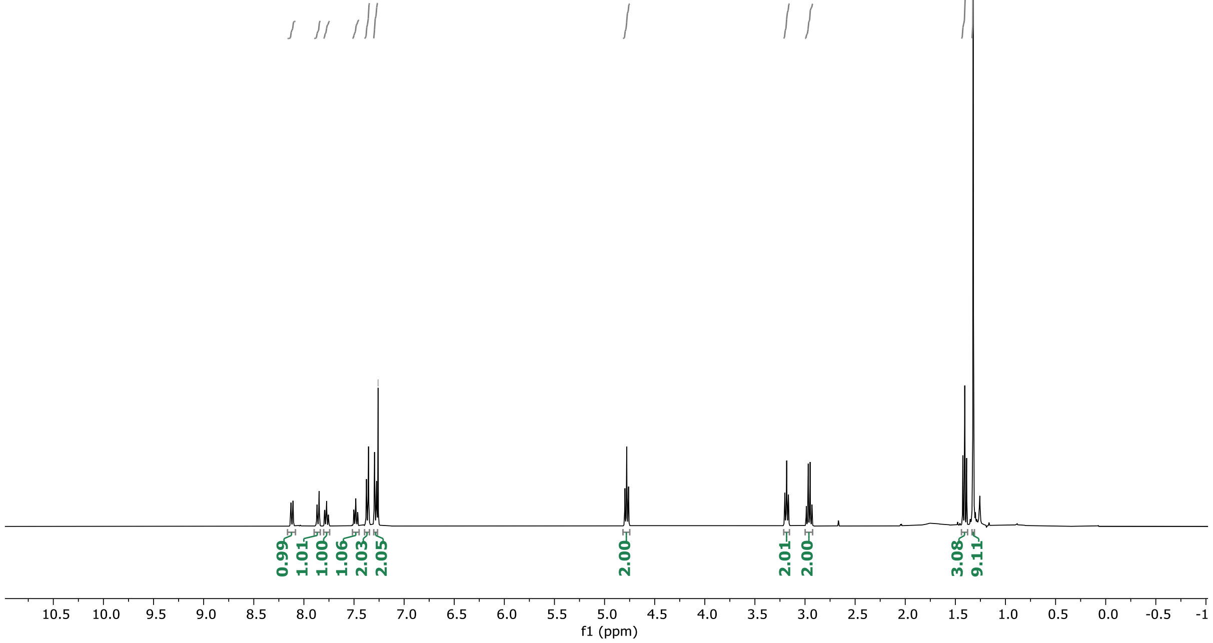

^{13}C NMR (101 MHz, $CDCl_3$) of **21**

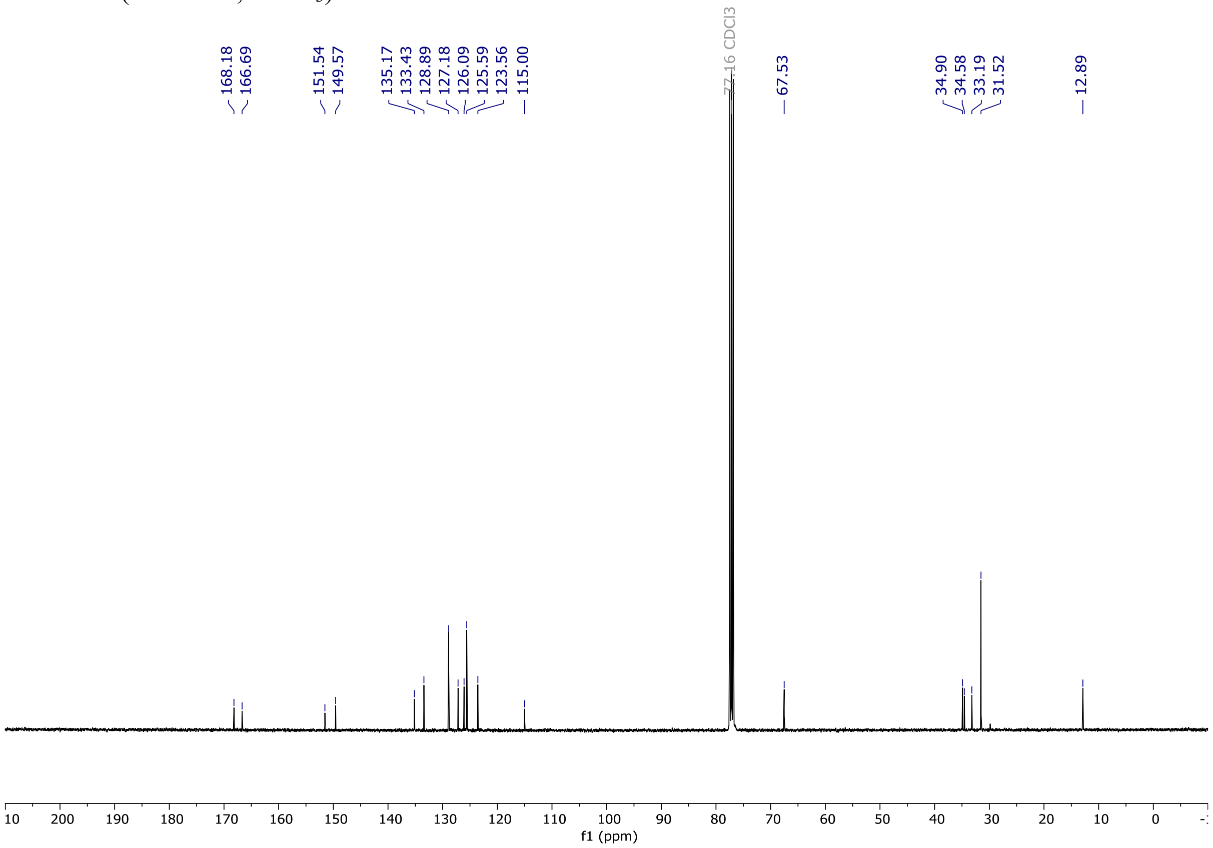

^{1}H NMR (400 MHz, $CDCl_3$) of **22 major**

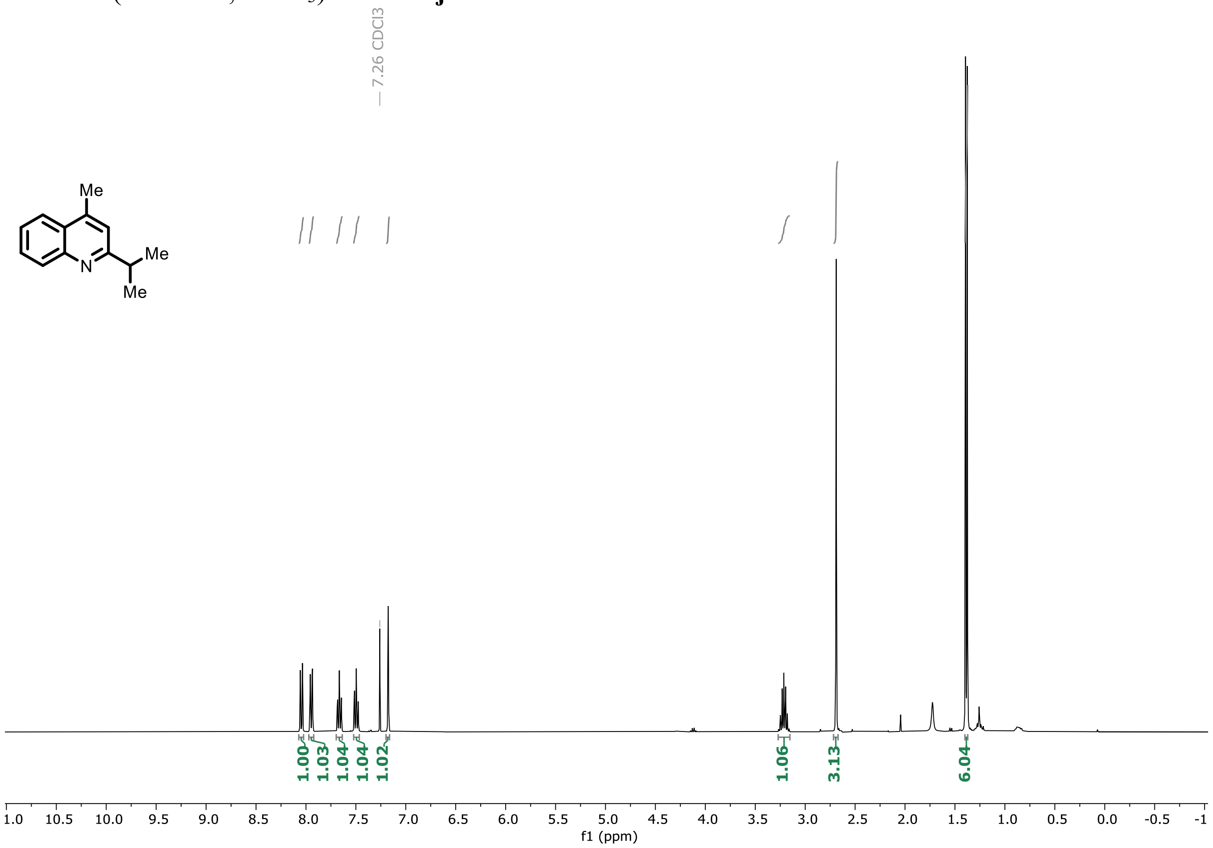

^{13}C NMR (101 MHz, $CDCl_3$) of **22 major**

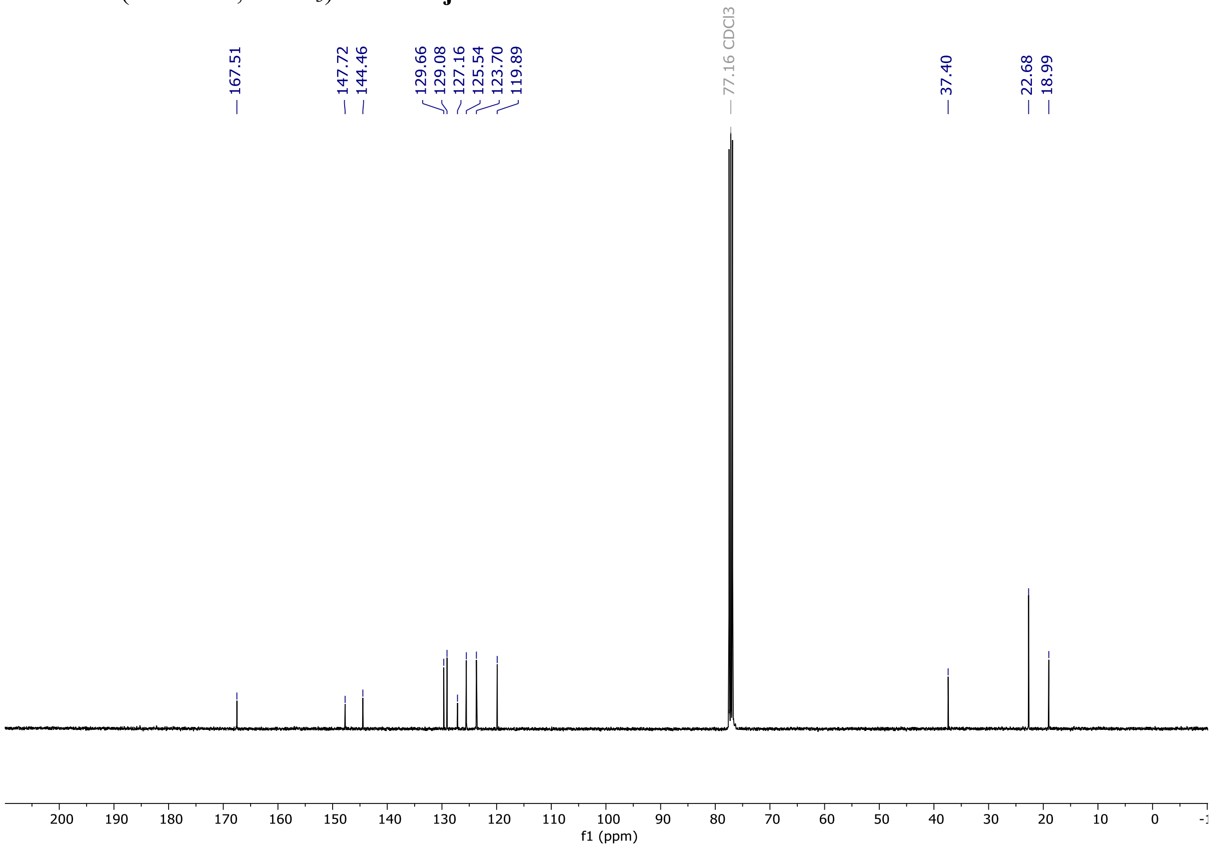

^{1}H NMR (400 MHz, $CDCl_3$) of **22 minor**

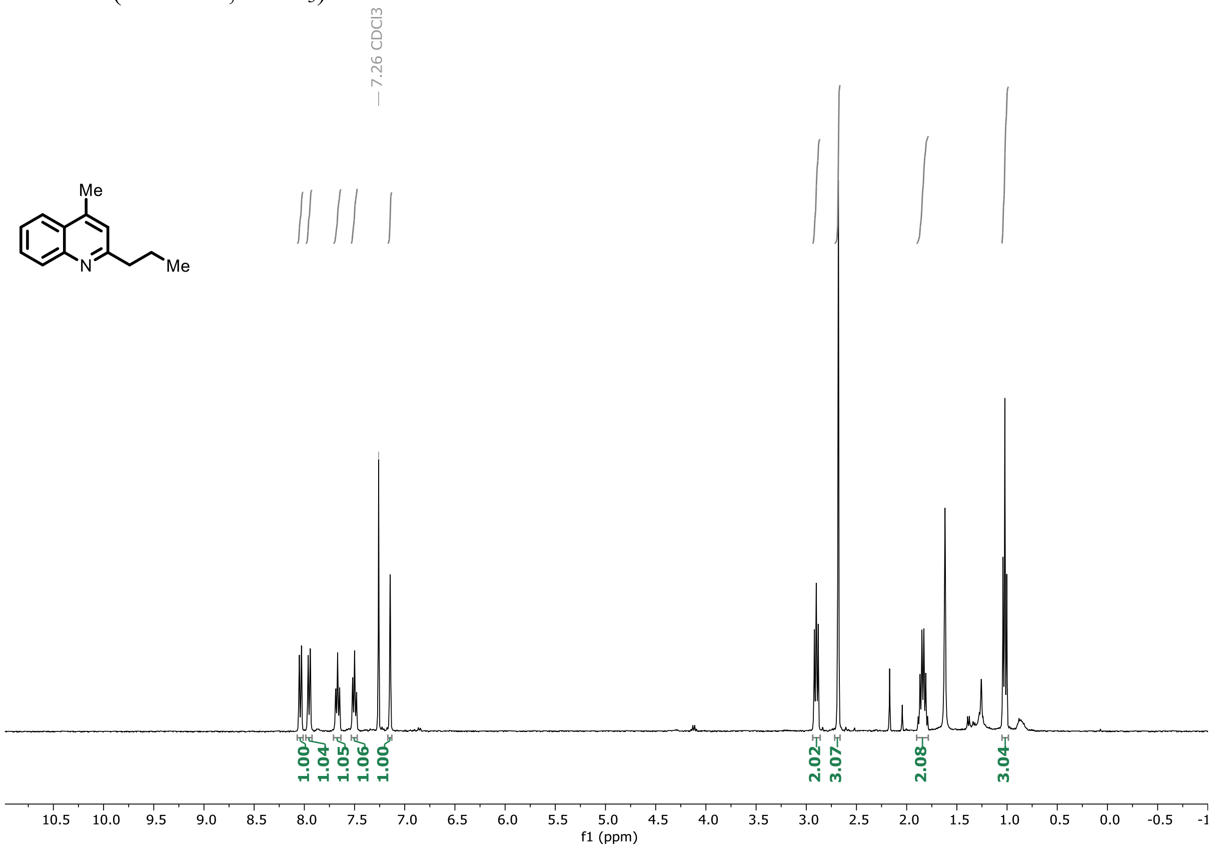

^{13}C NMR (101 MHz, $CDCl_3$) of **22 minor**

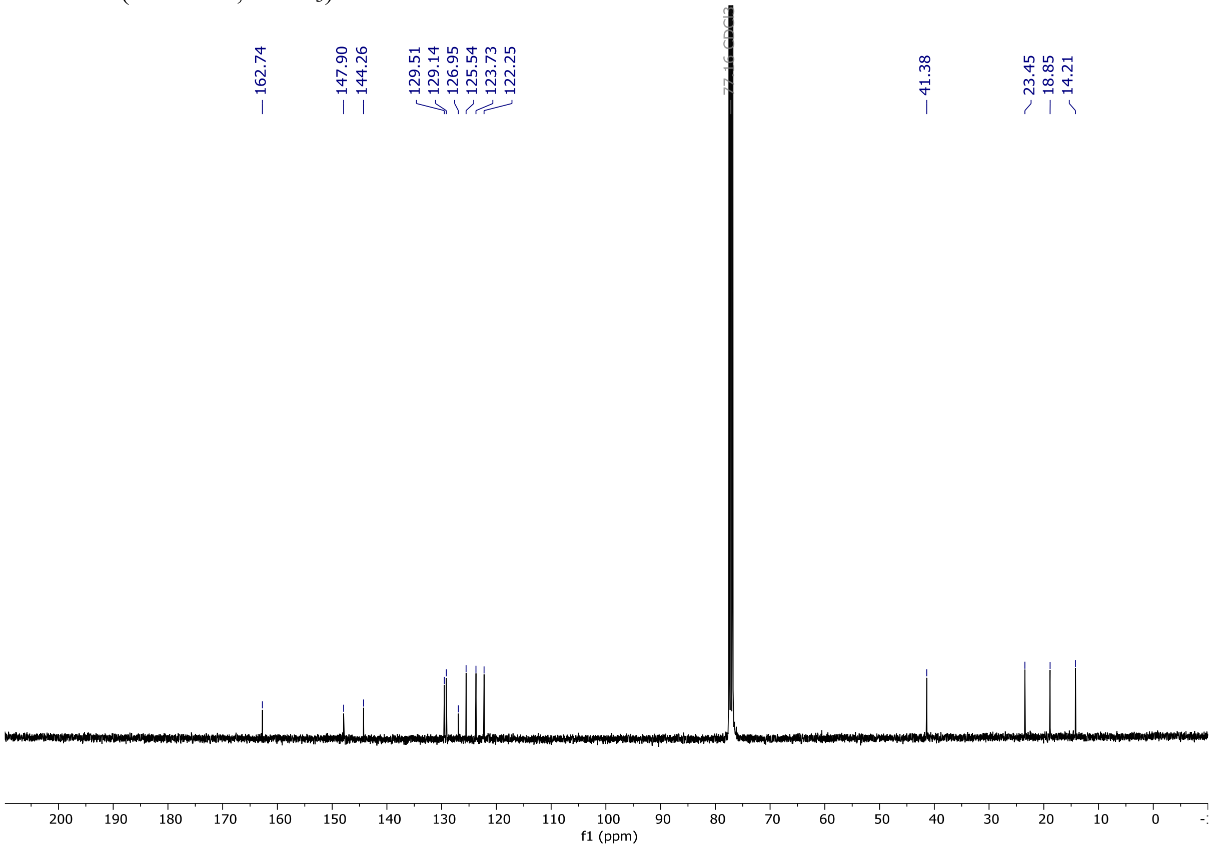

^{1}H NMR (400 MHz, $CDCl_3$) of **23 major**

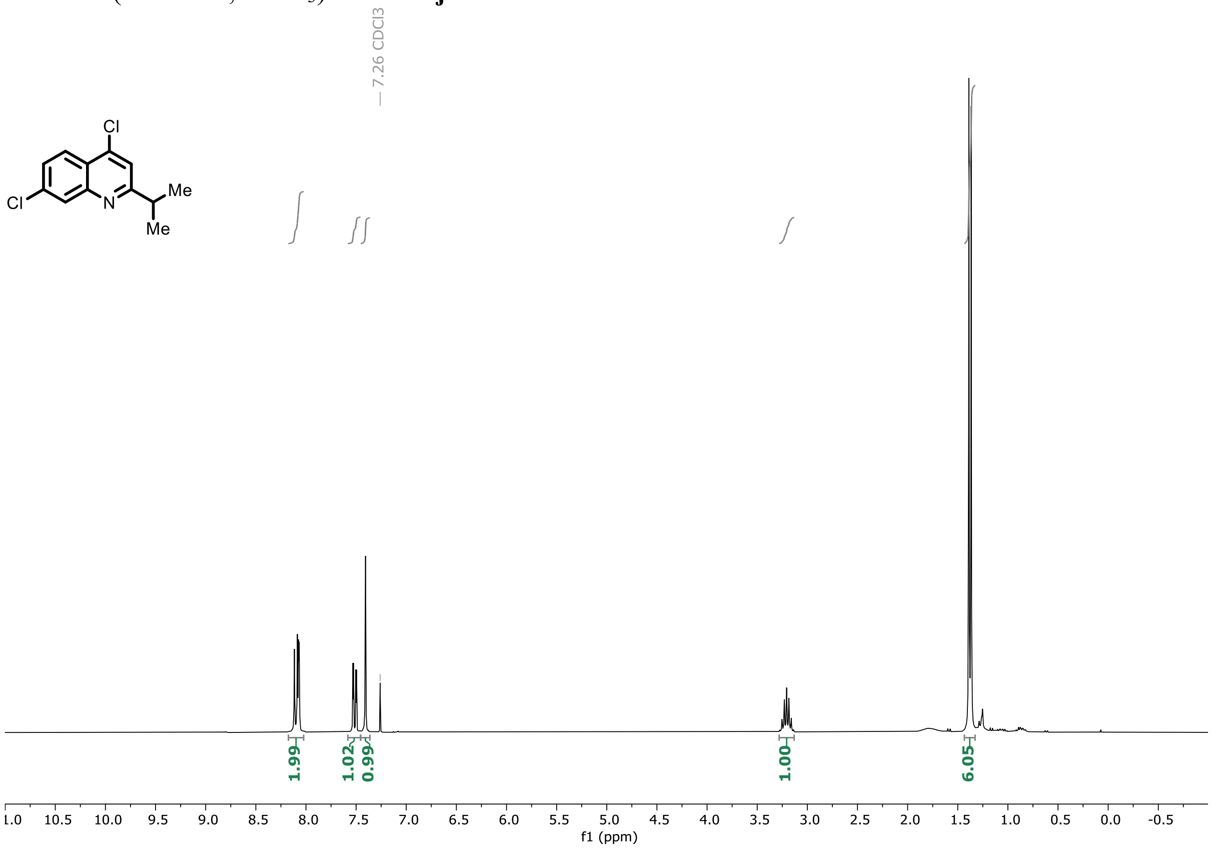

^{13}C NMR (101 MHz, $CDCl_3$) of **23 major**

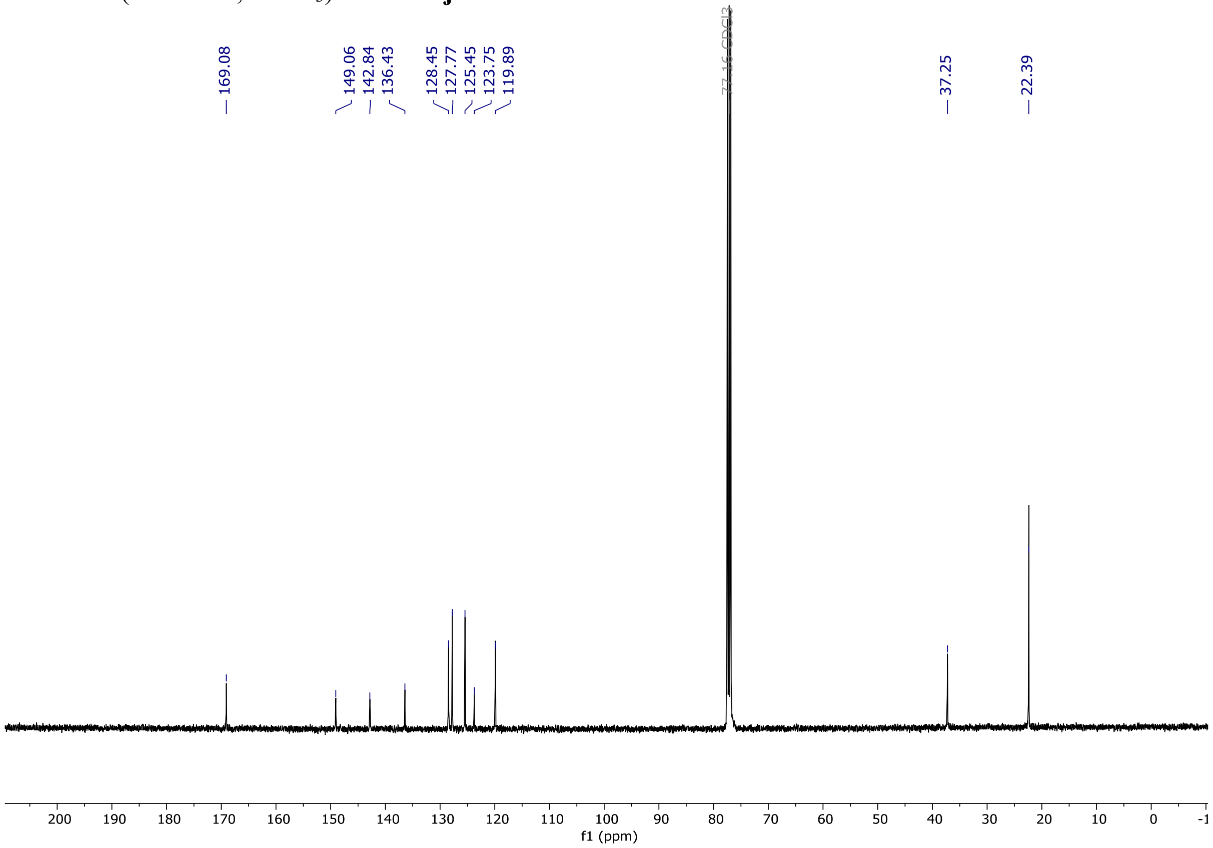

^{1}H NMR (400 MHz, $CDCl_3$) of **23 minor**

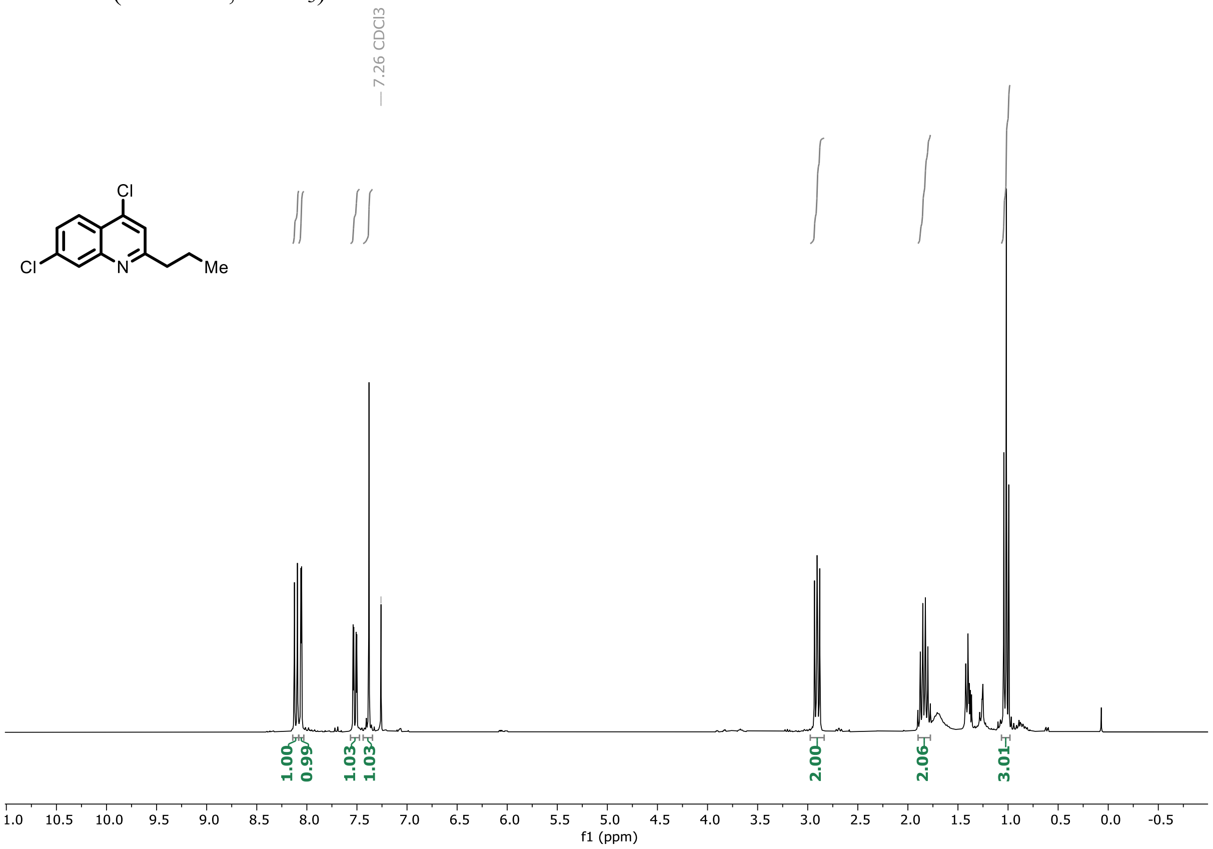

^{13}C NMR (101 MHz, $CDCl_3$) of **23 minor**

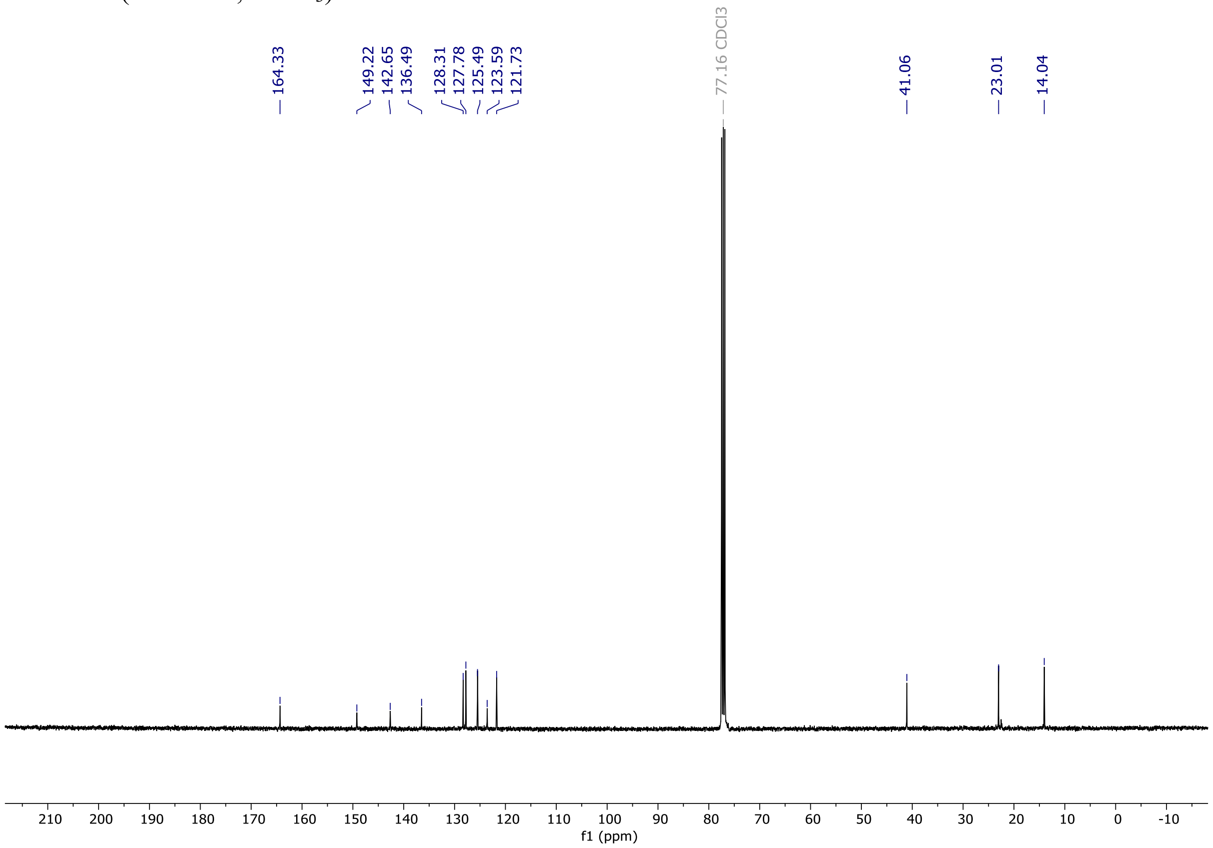

^{1}H NMR (400 MHz, $CDCl_3$) of **24 major**

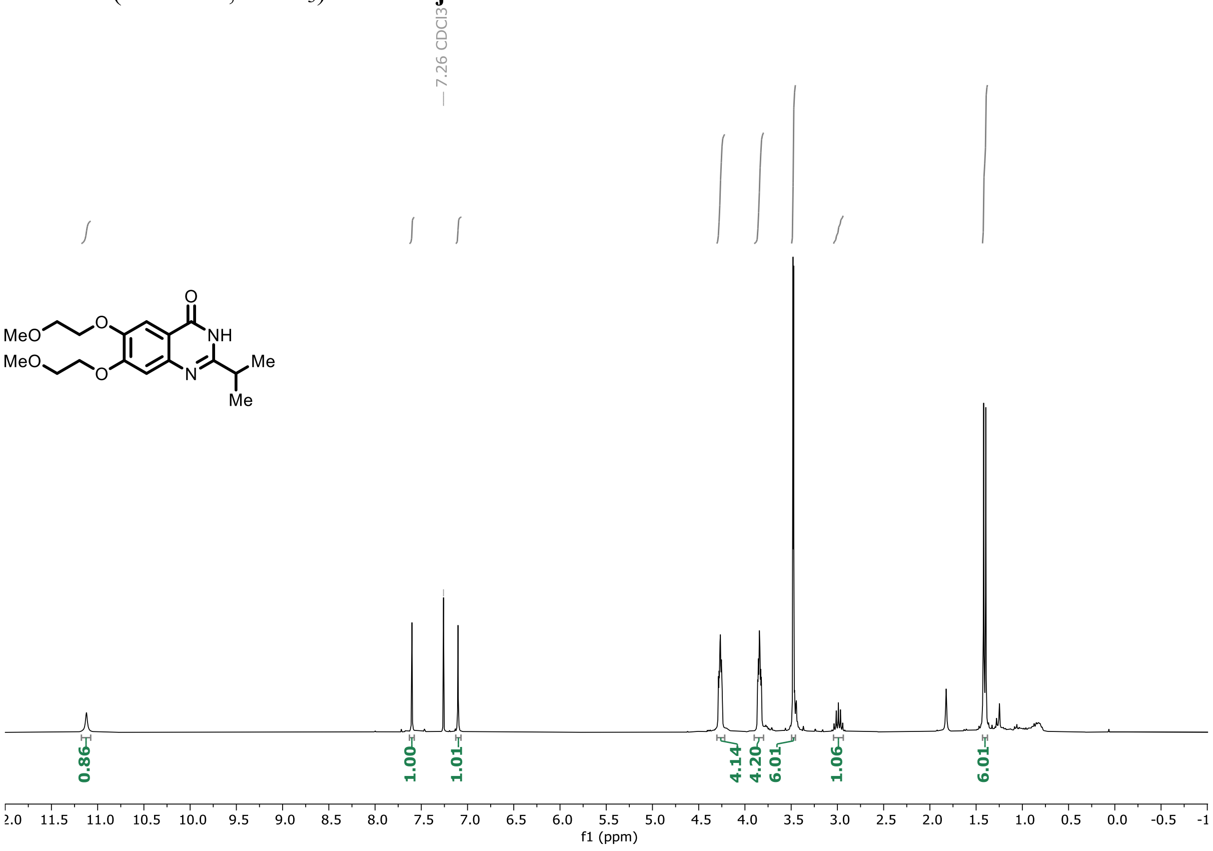

^{13}C NMR (101 MHz, $CDCl_3$) of **24 major**

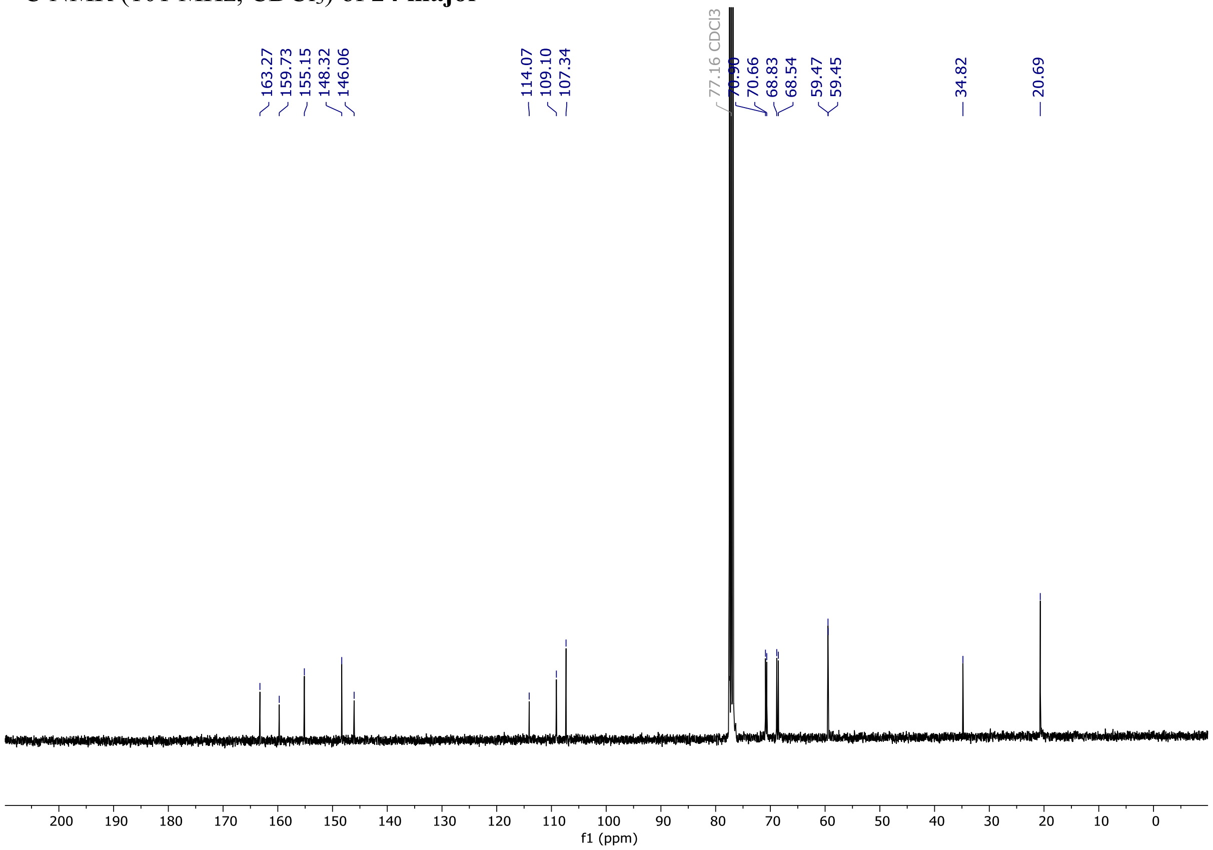

^{1}H NMR (400 MHz, $CDCl_3$) of **24 minor**

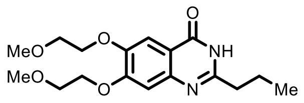

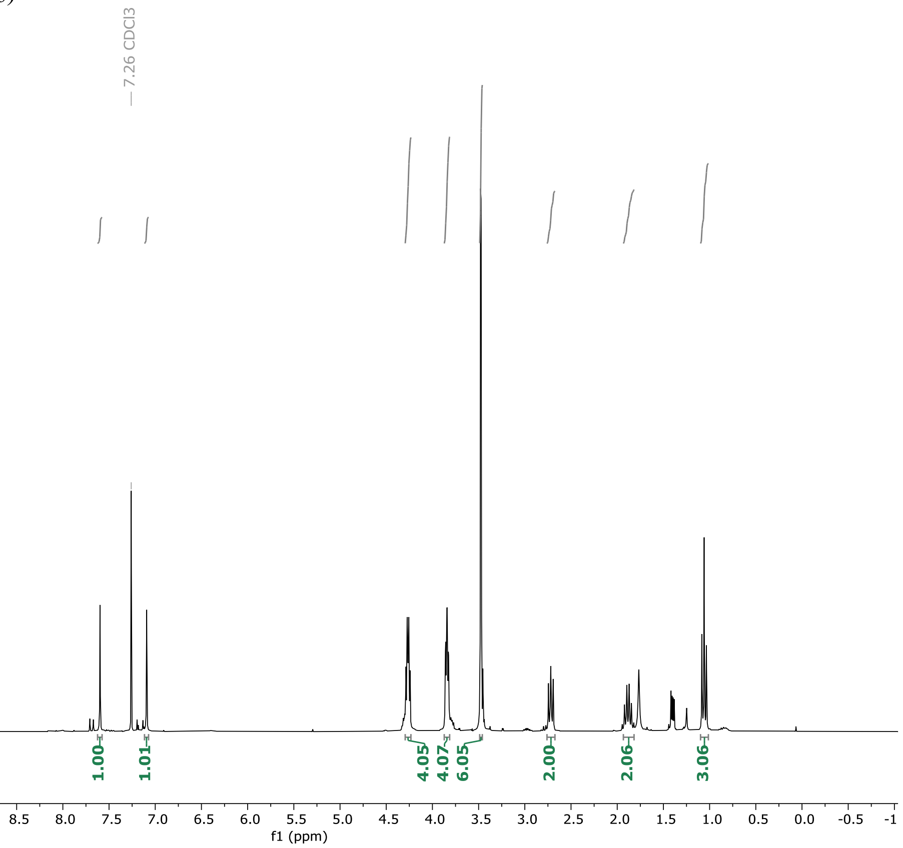

^{13}C NMR (101 MHz, $CDCl_3$) of **24 minor**

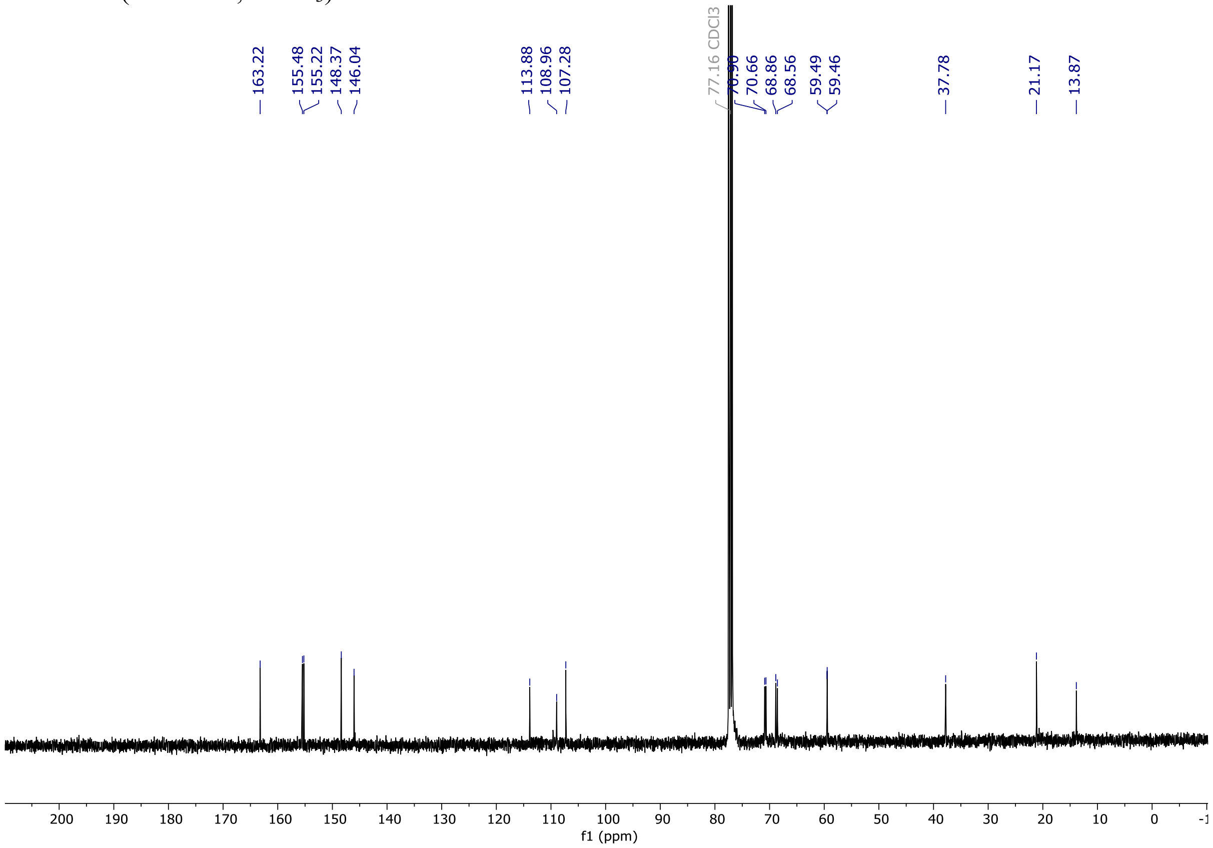

^{1}H NMR (400 MHz, $CDCl_3$) of **25 major**

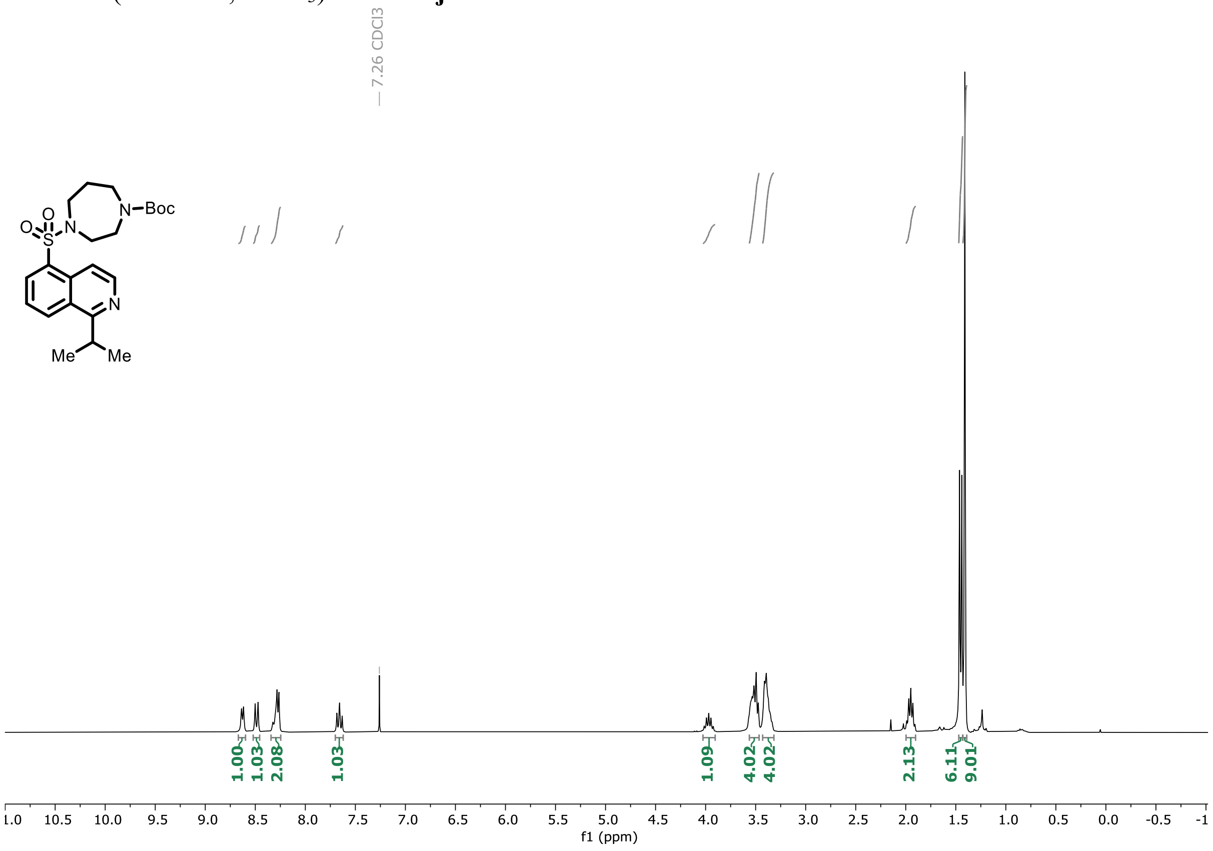

^{13}C NMR (101 MHz, $CDCl_3$) of **25 major**

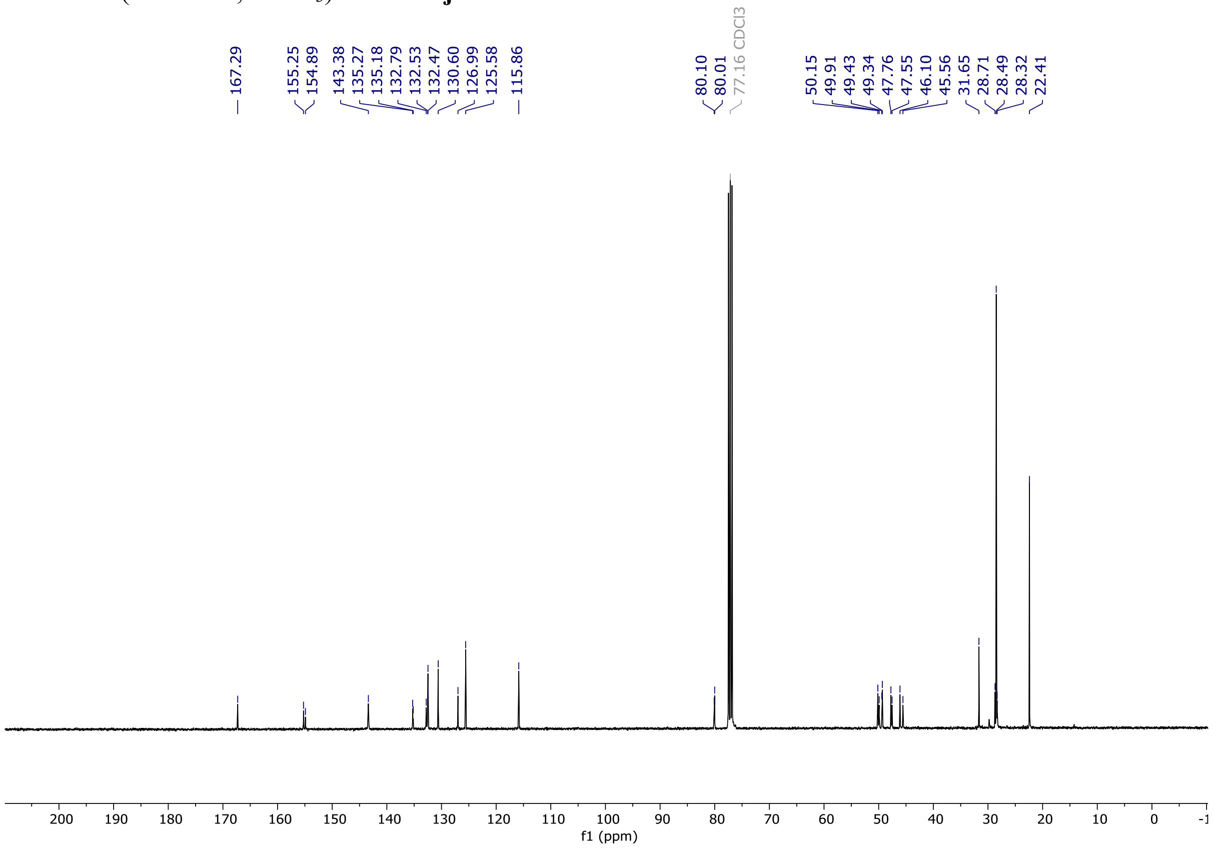

^{1}H NMR (400 MHz, $CDCl_3$) of **25 minor**

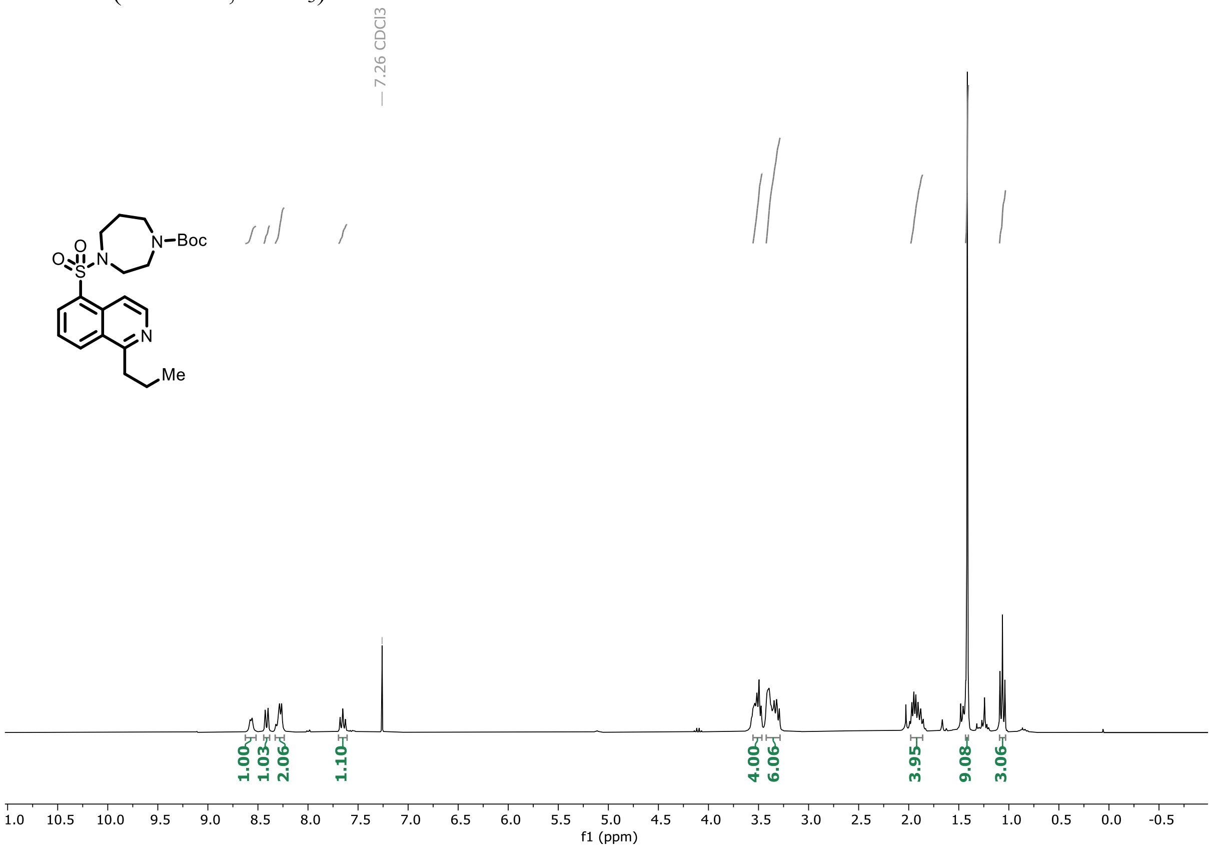

^{13}C NMR (101 MHz, $CDCl_3$) of **25 minor**

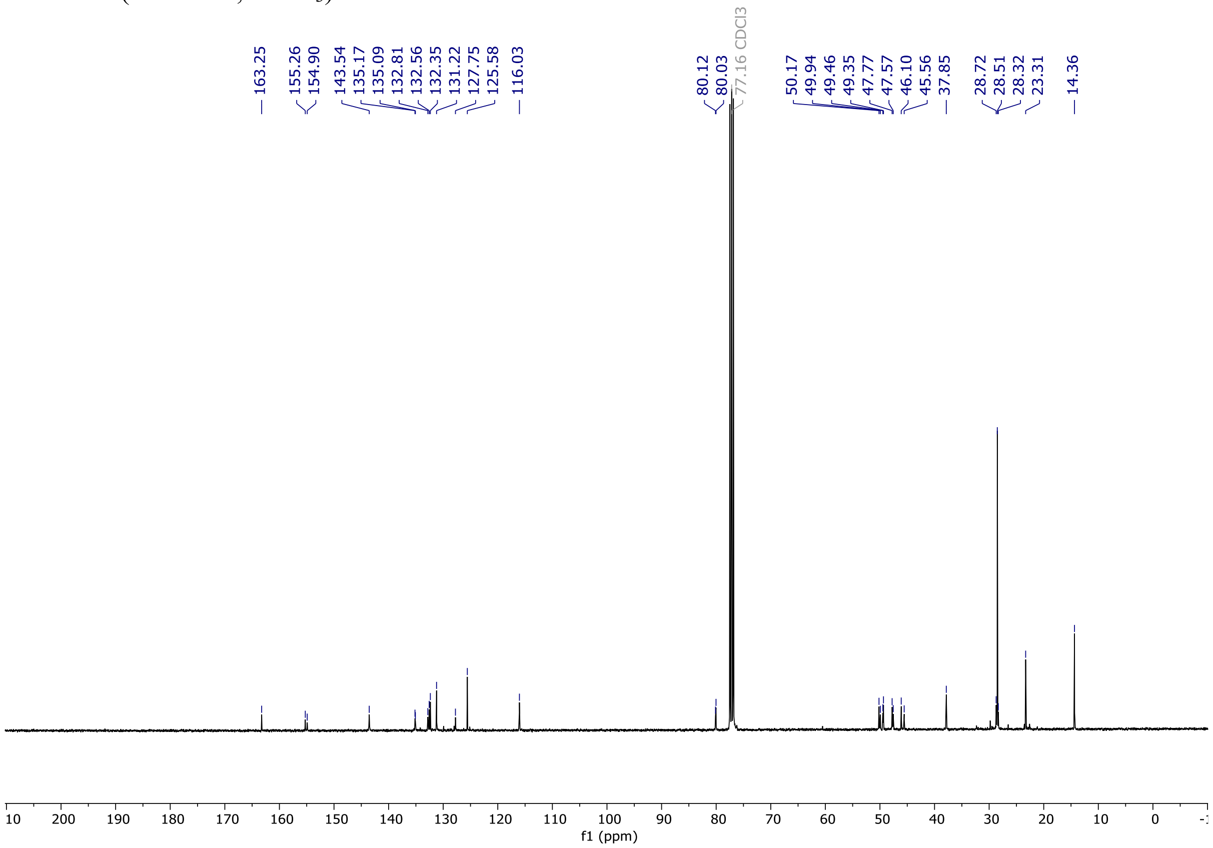

^{1}H NMR (400 MHz, $CDCl_3$) of **26 major**

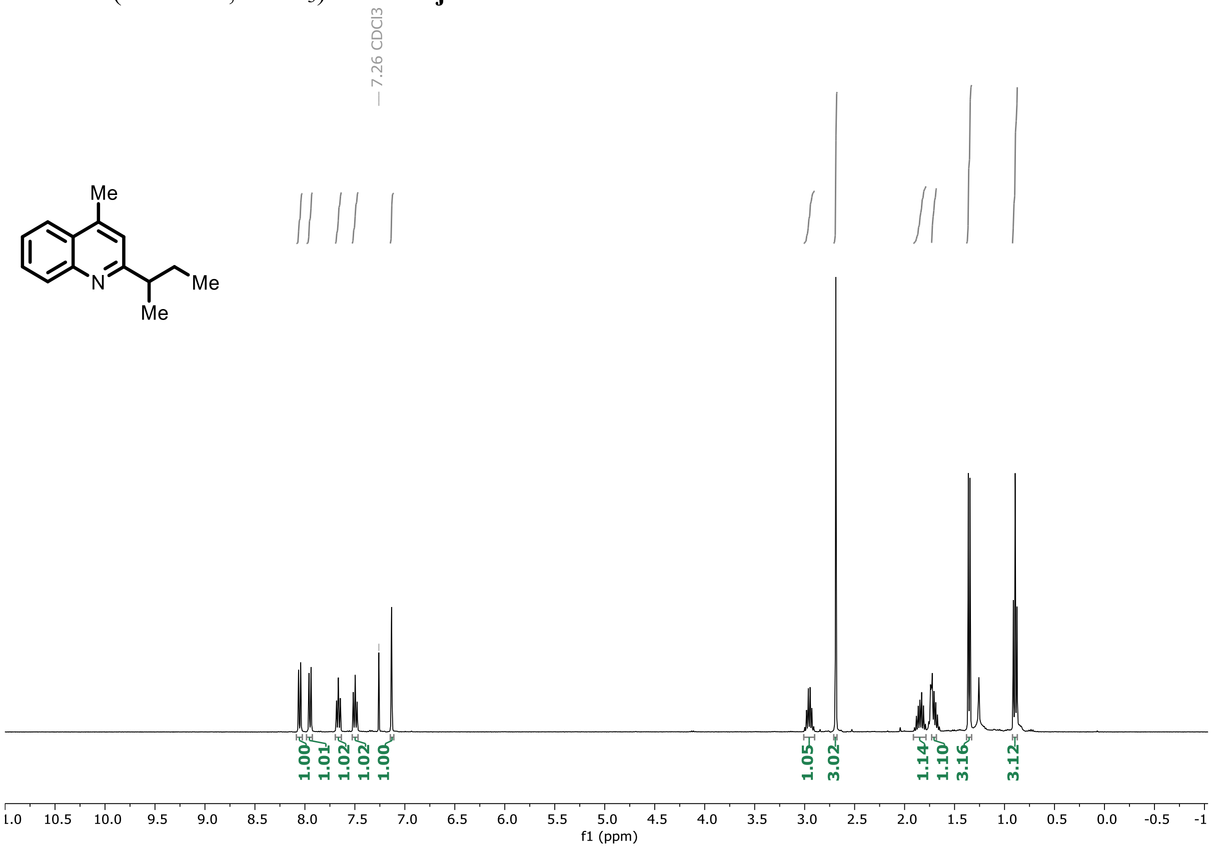

^{13}C NMR (101 MHz, $CDCl_3$) of **26 major**

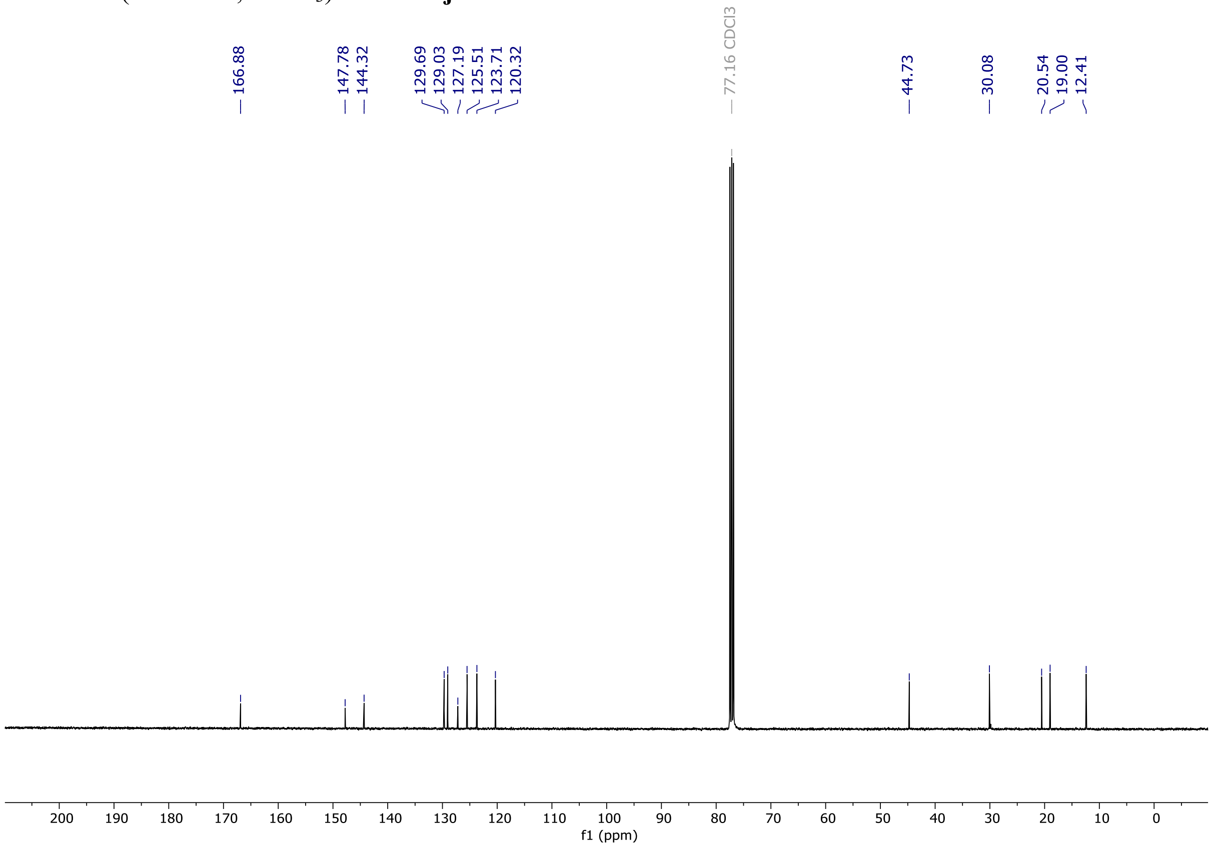

^{1}H NMR (400 MHz, $CDCl_3$) of **26 minor**

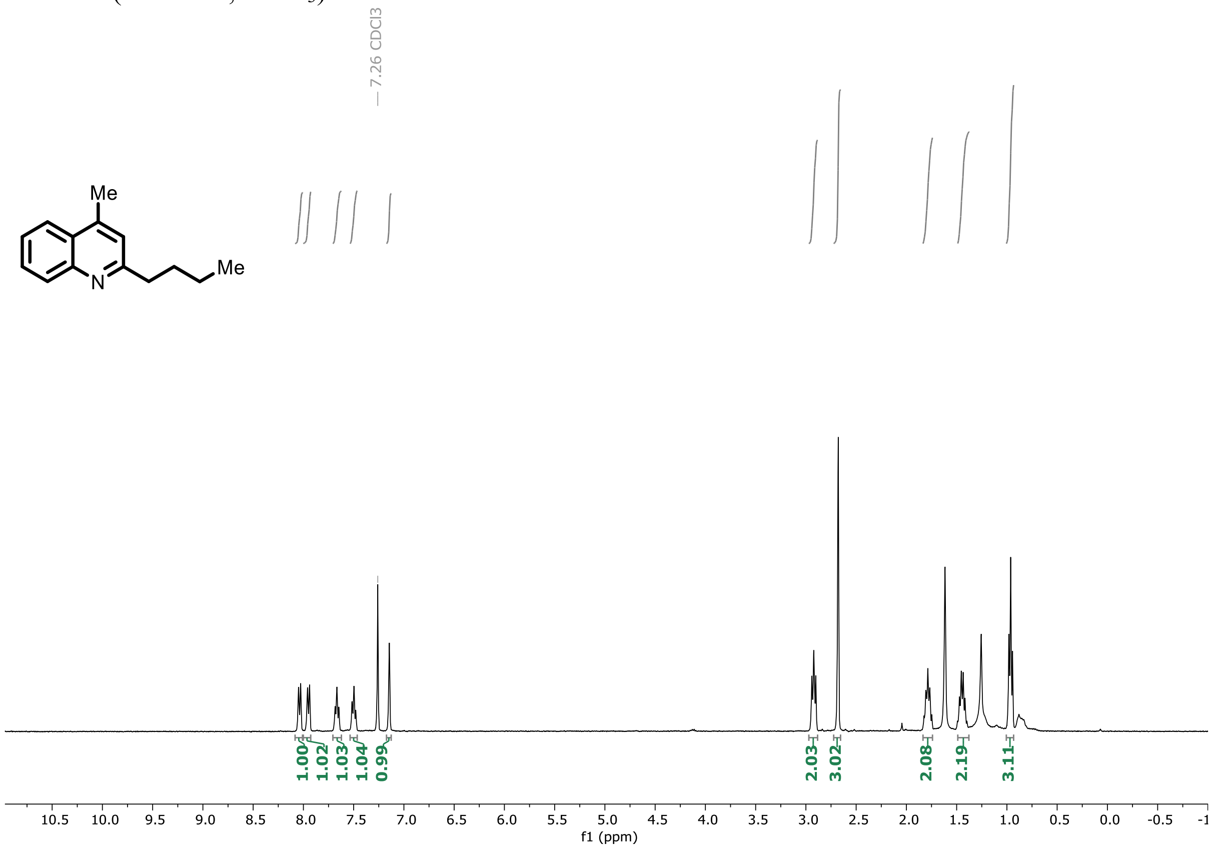

^{13}C NMR (101 MHz, $CDCl_3$) of **26 minor**

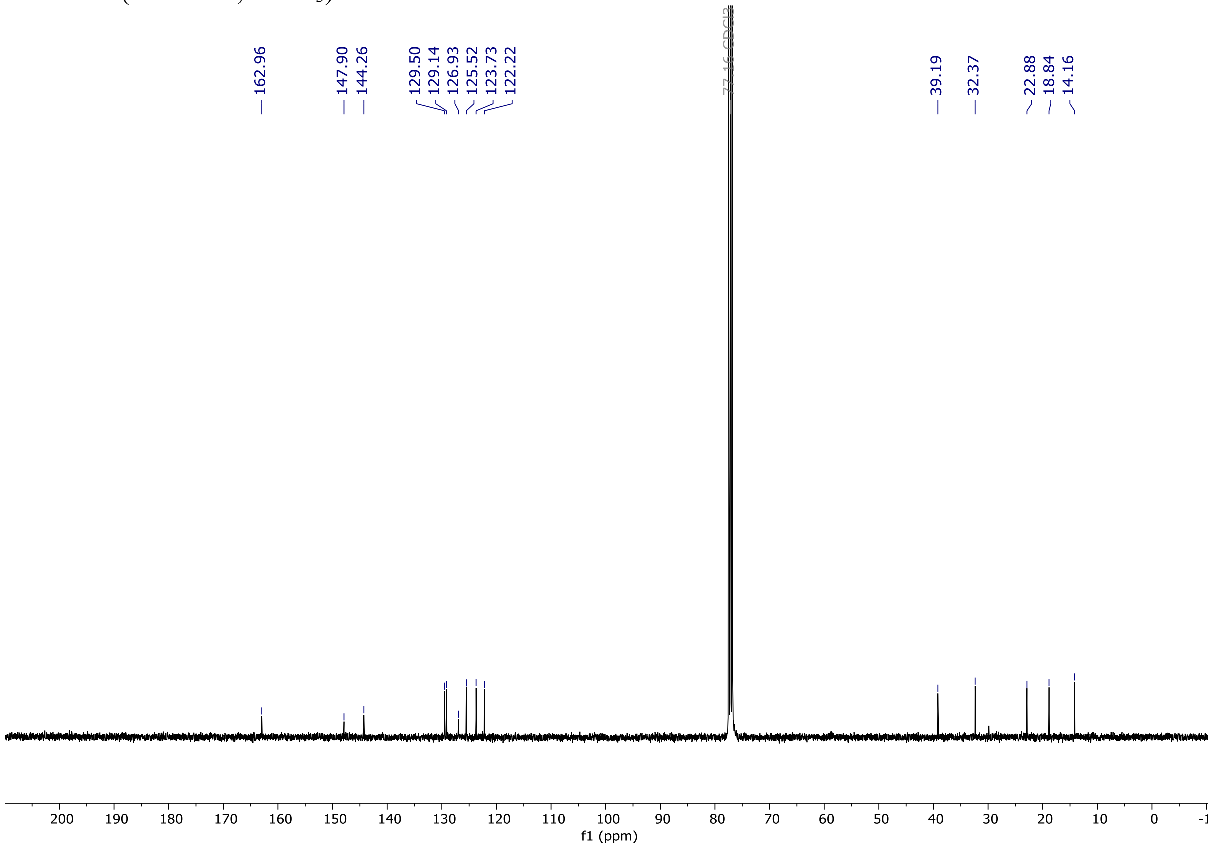

^{1}H NMR (400 MHz, $CDCl_3$) of **27 major**

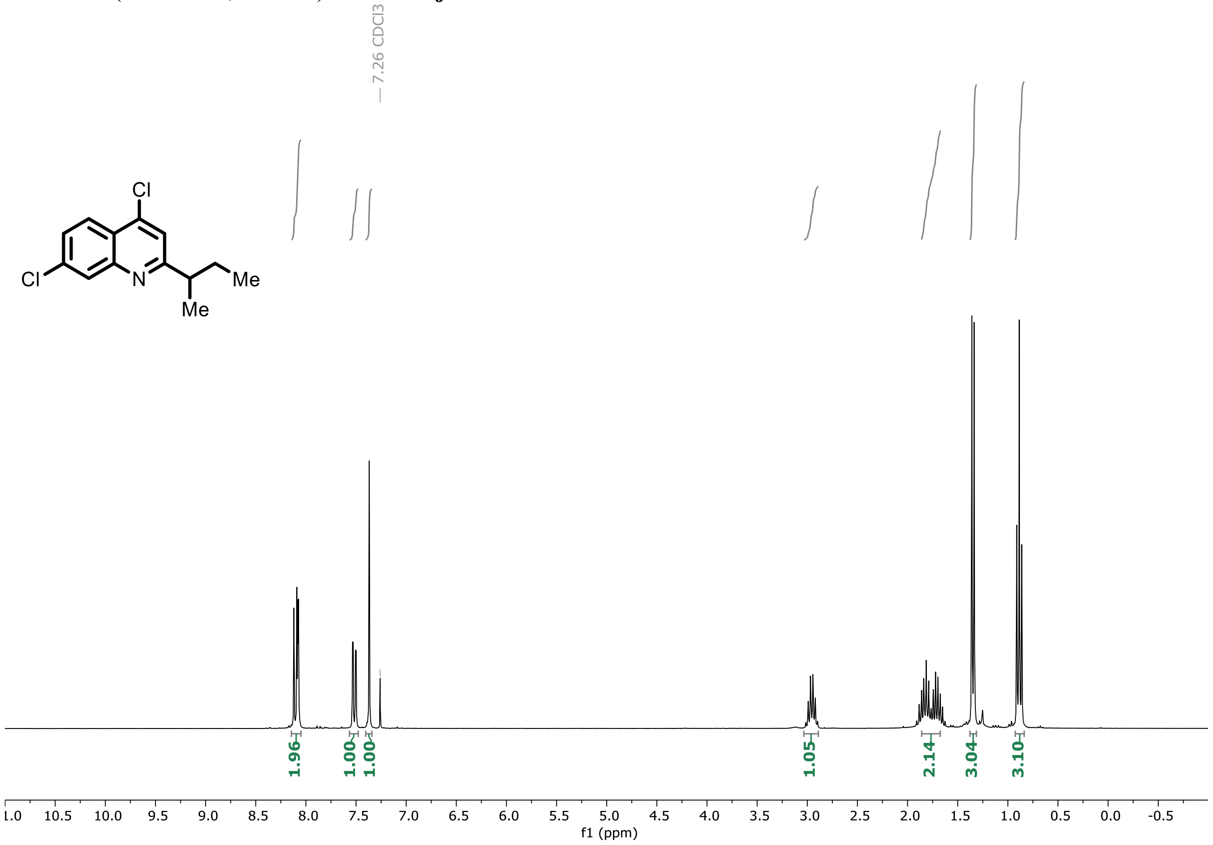

^{13}C NMR (101 MHz, $CDCl_3$) of **27 major**

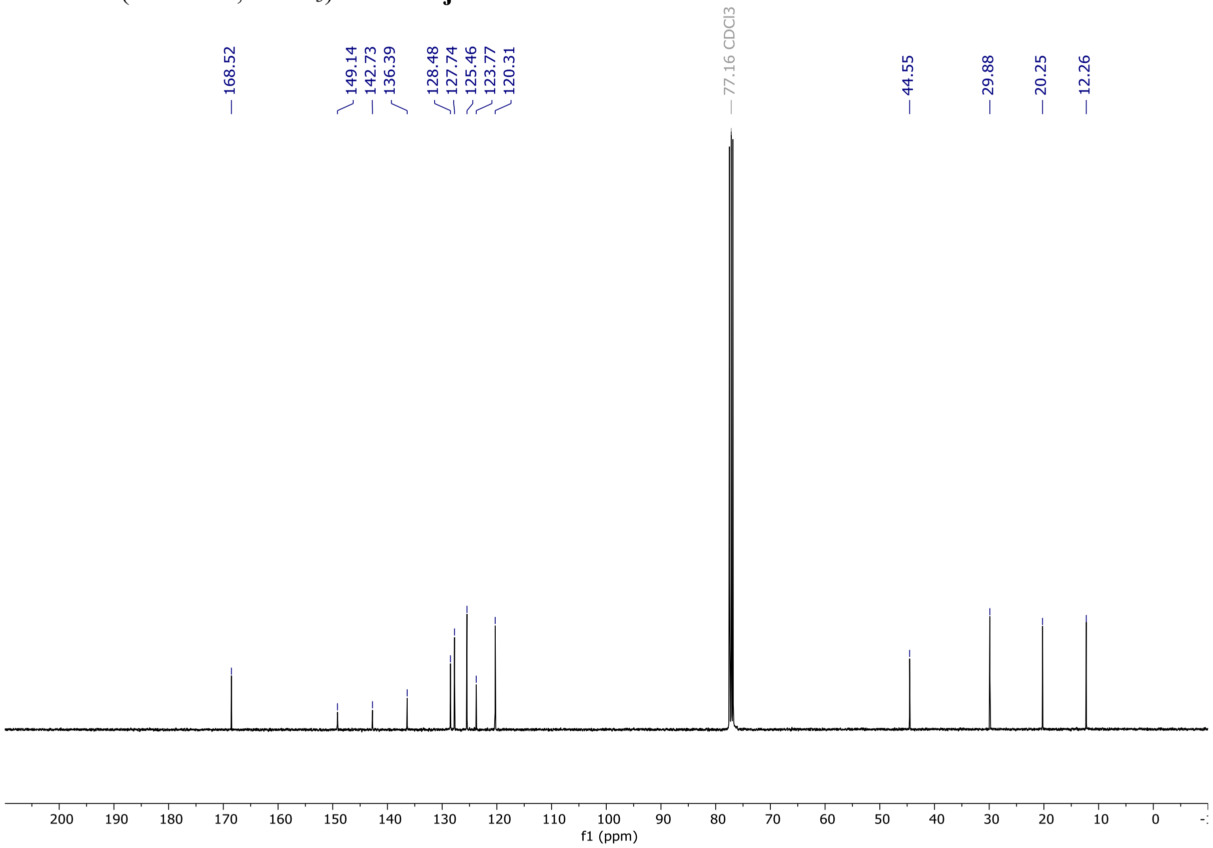

^{1}H NMR (400 MHz, $CDCl_3$) of **27 minor**

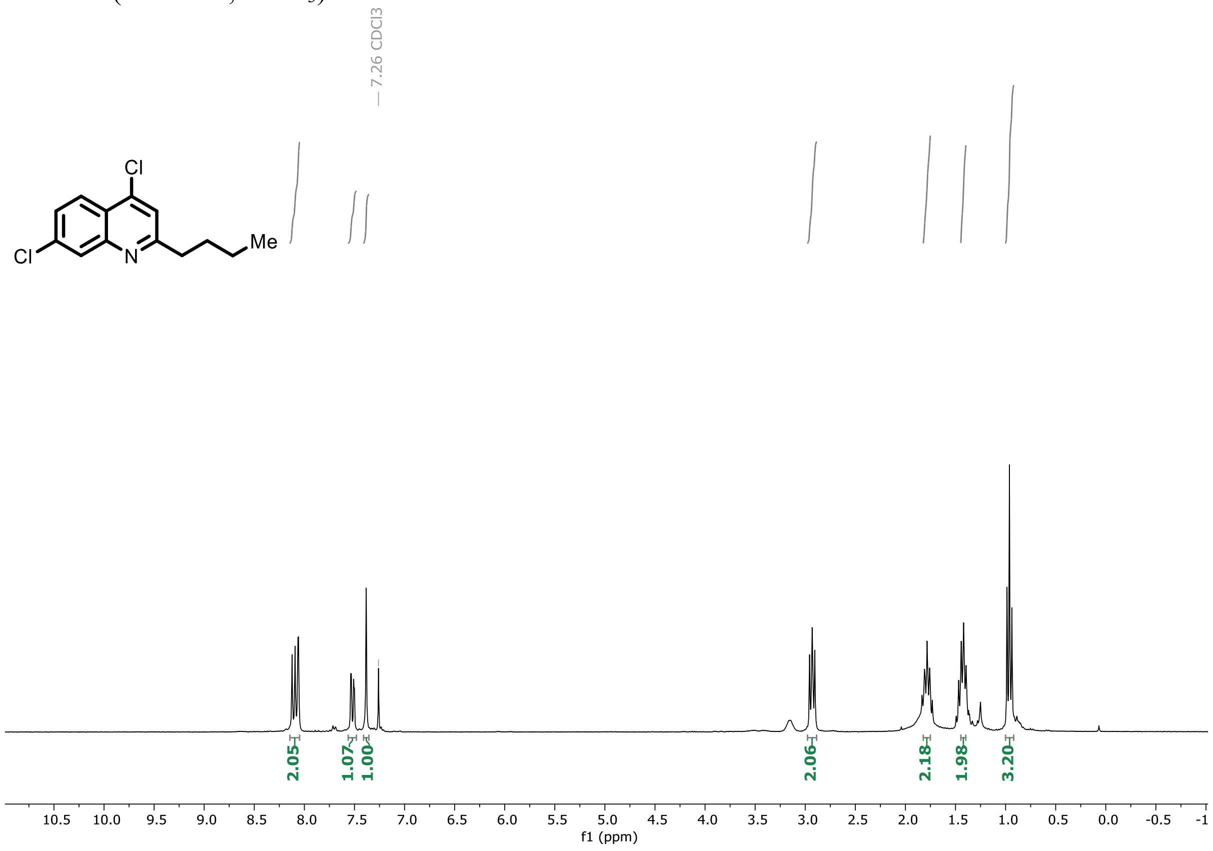

^{13}C NMR (101 MHz, $CDCl_3$) of **27 minor**

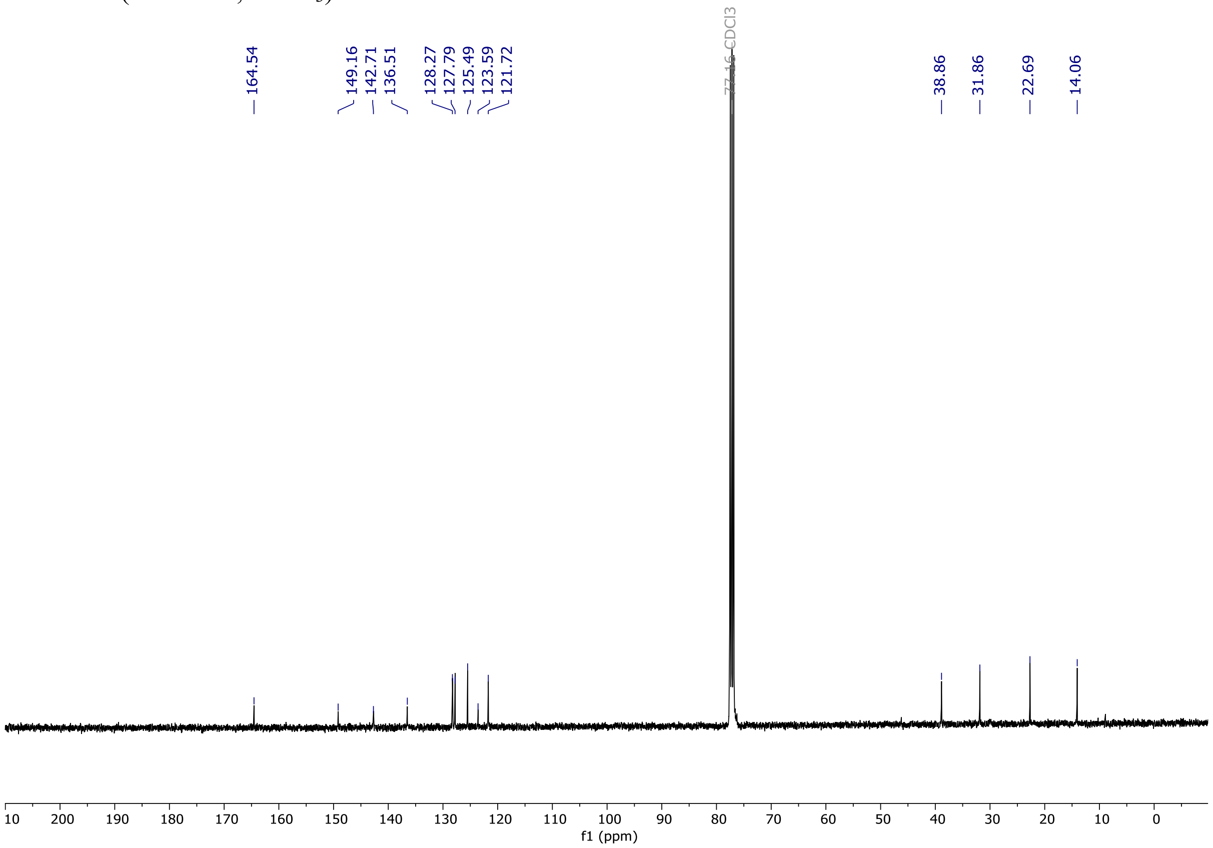

^{1}H NMR (400 MHz, $CDCl_3$) of **28 major**

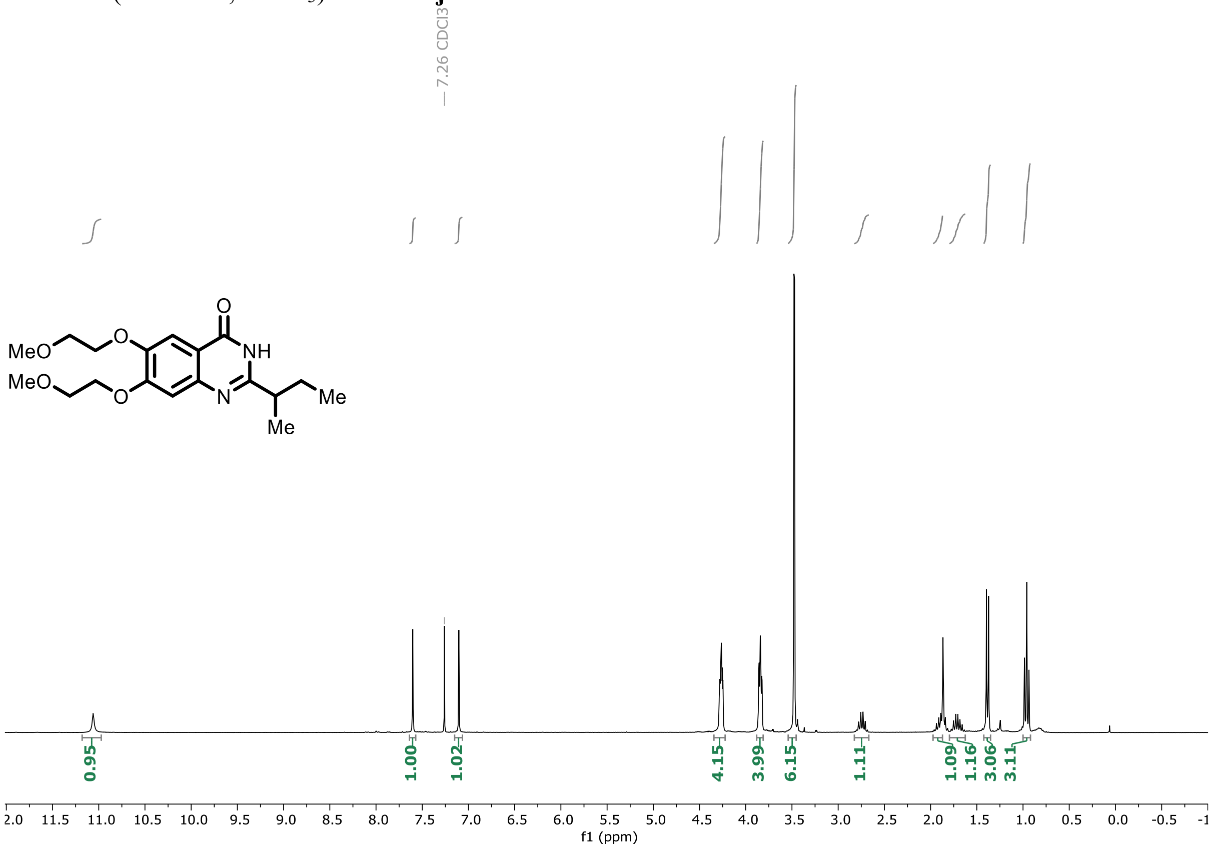

^{13}C NMR (101 MHz, $CDCl_3$) of **28 major**

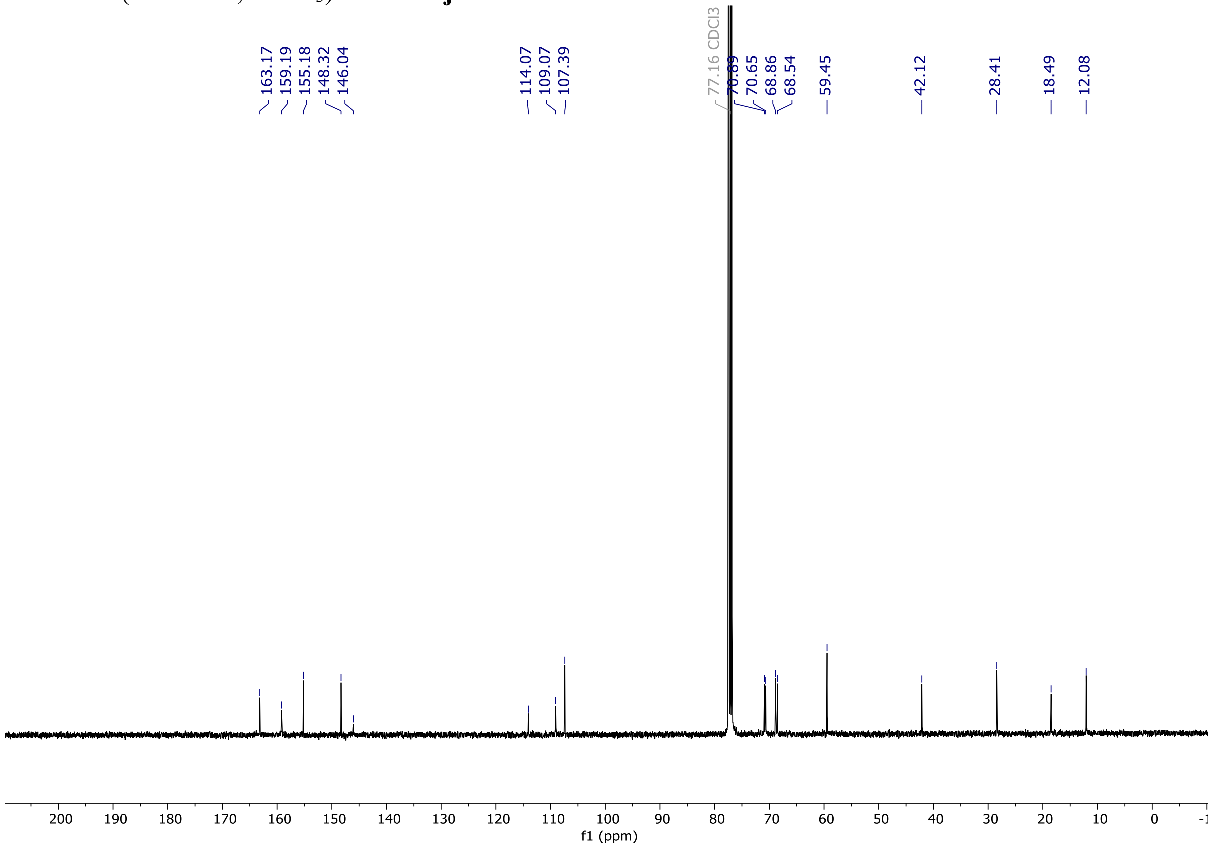

^{1}H NMR (400 MHz, $CDCl_3$) of **28 minor**

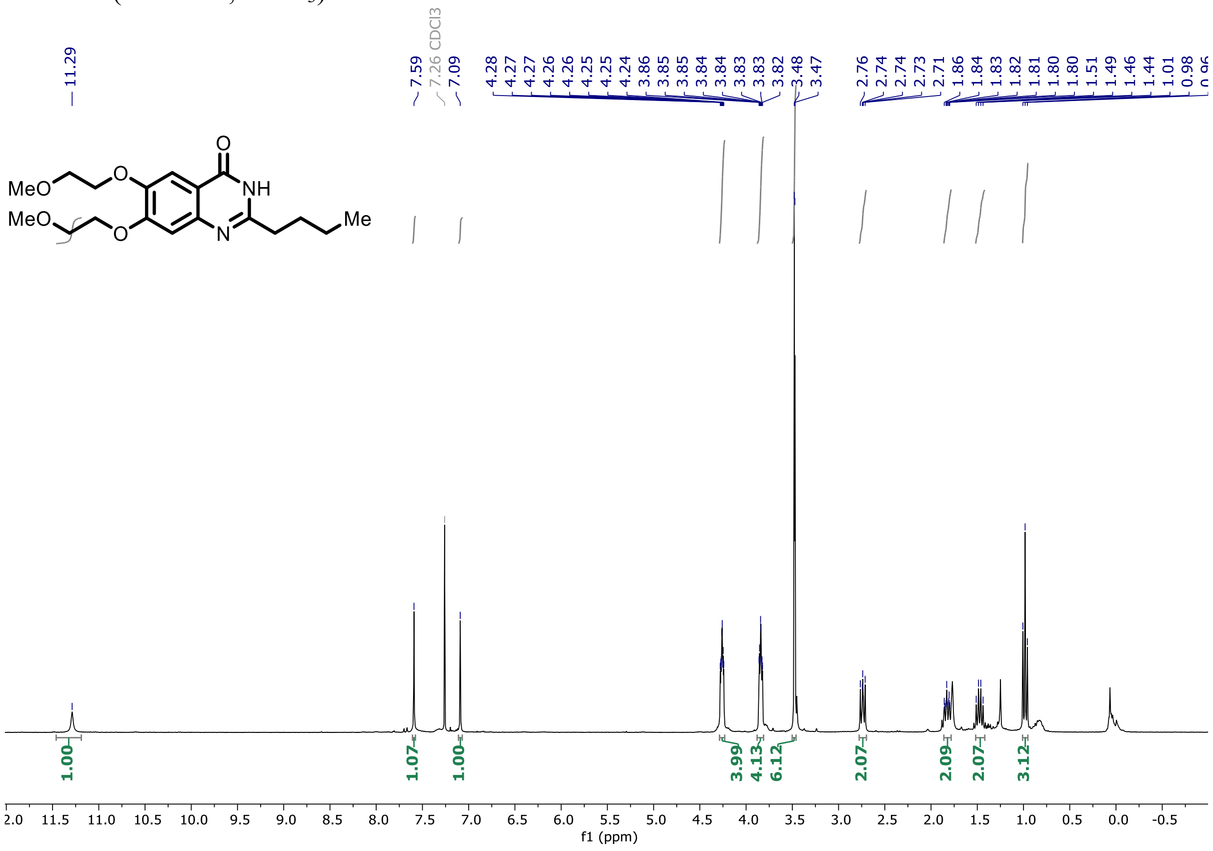

^{13}C NMR (101 MHz, $CDCl_3$) of **28 minor**

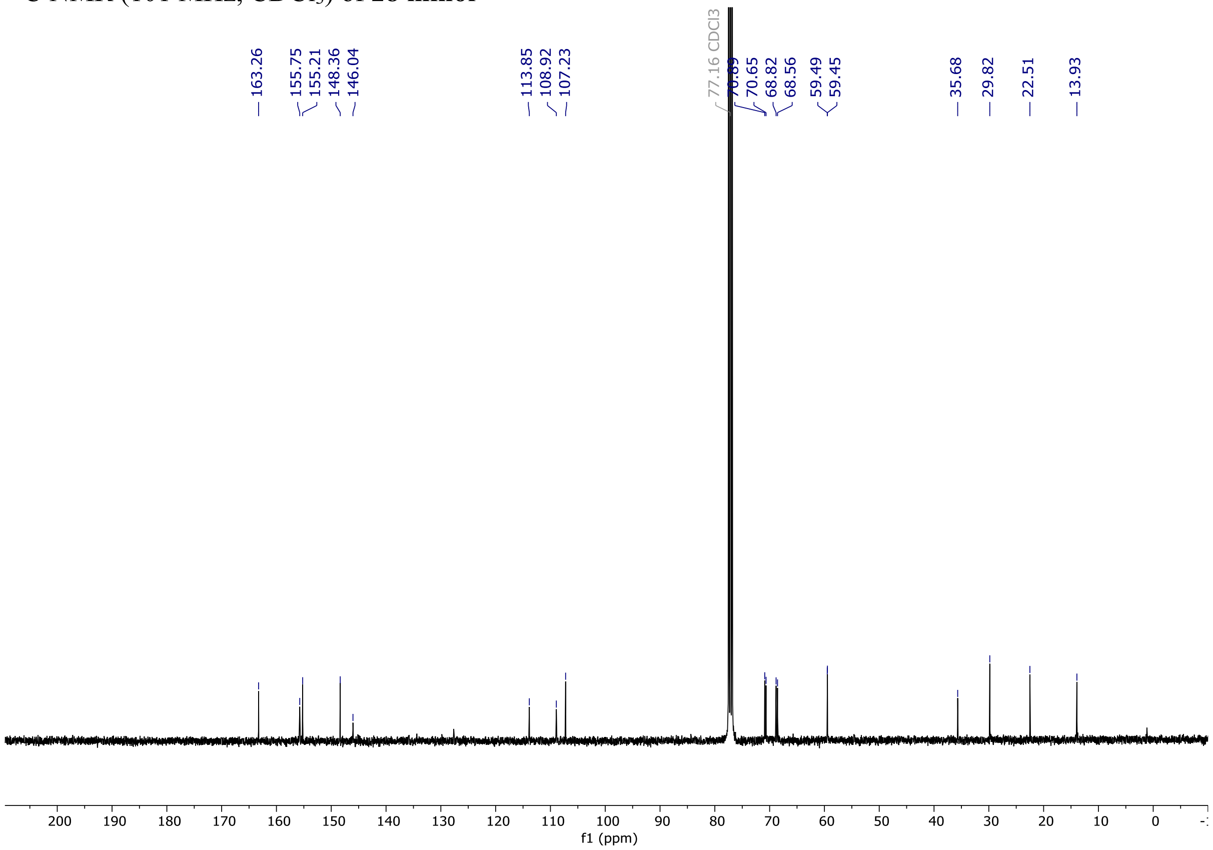

^{1}H NMR (400 MHz, $CDCl_3$) of **29 major**

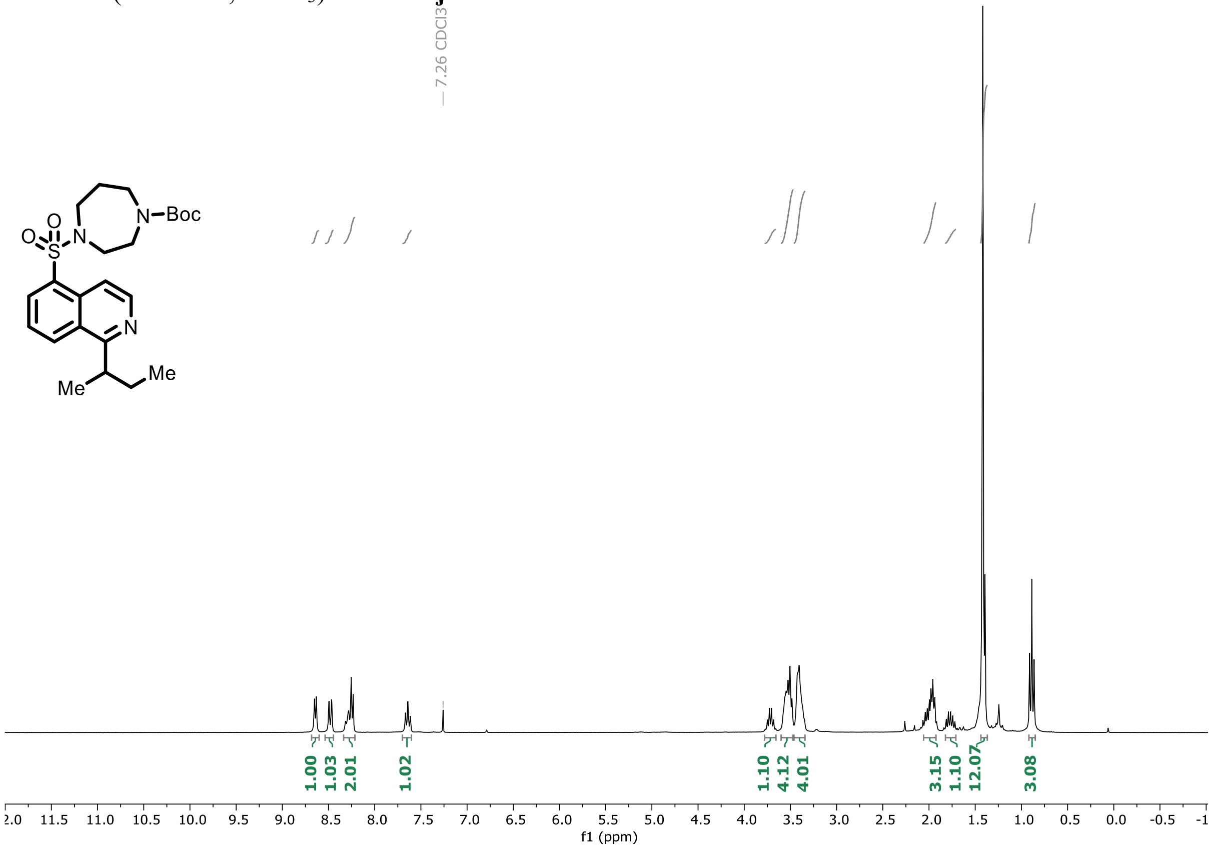

^{13}C NMR (101 MHz, $CDCl_3$) of **29 major**

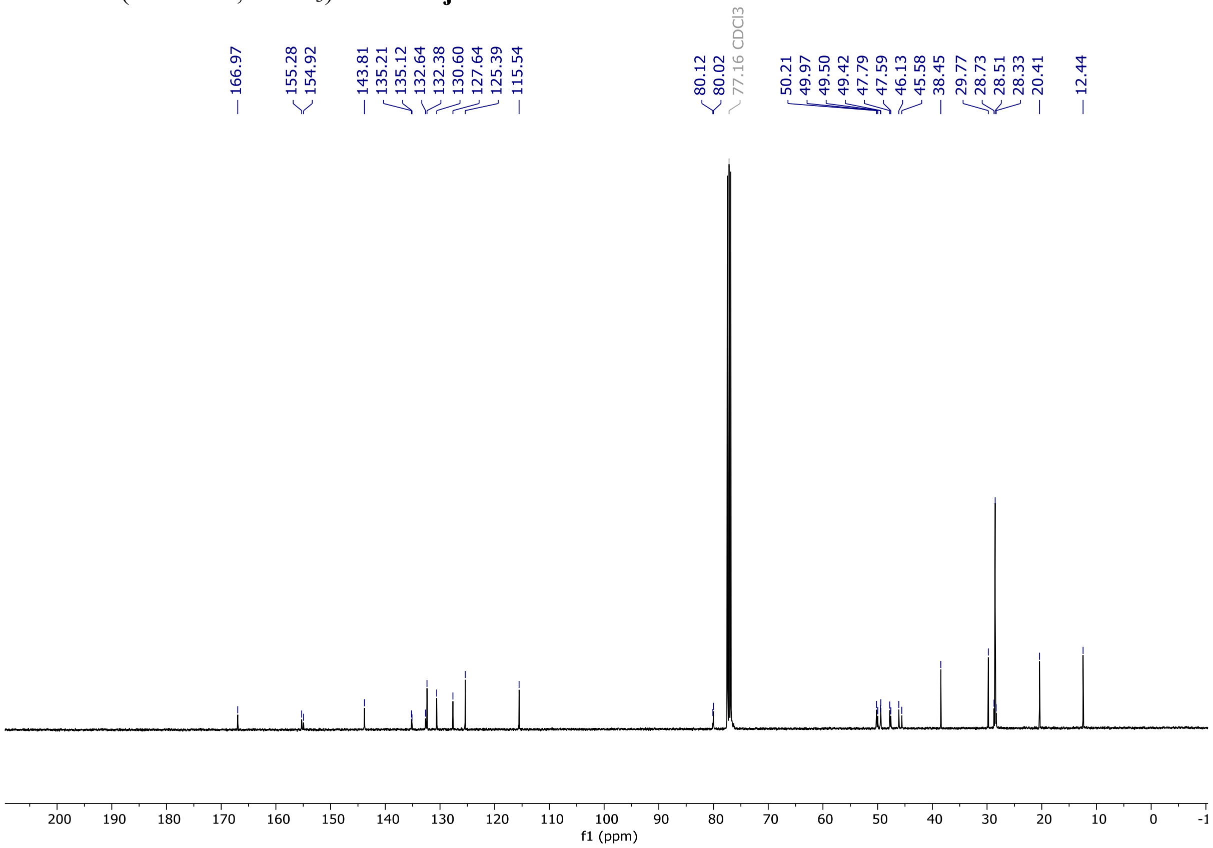

^{1}H NMR (400 MHz, $CDCl_3$) of **29 minor**

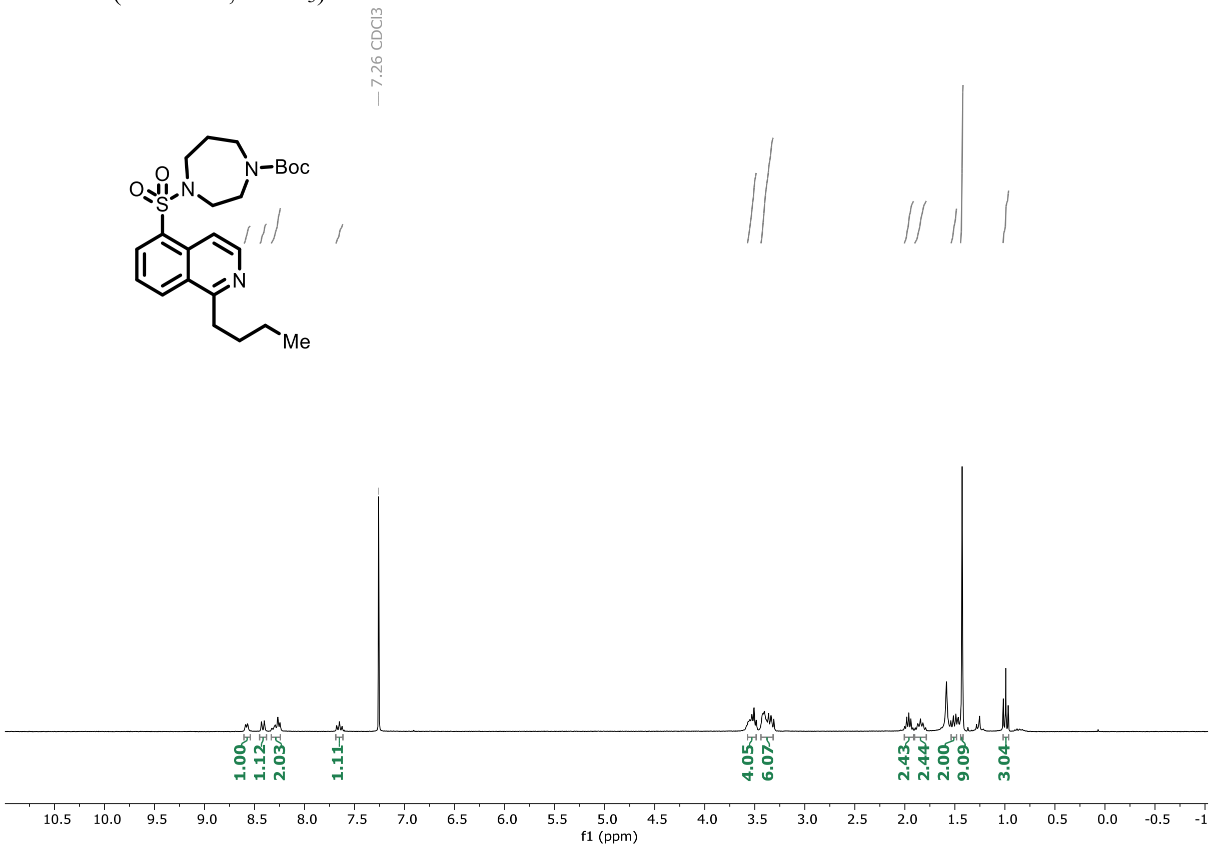

^{13}C NMR (101 MHz, $CDCl_3$) of **29 minor**

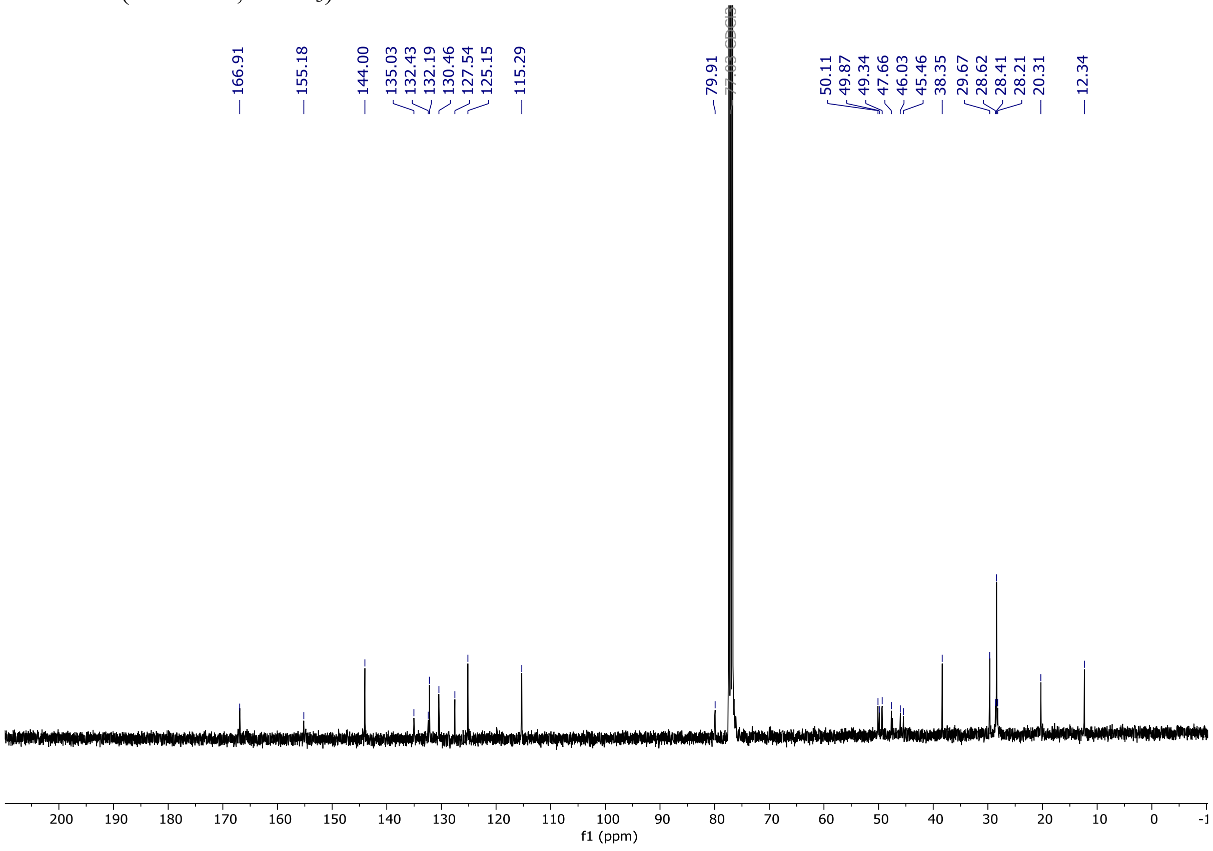

^{1}H NMR (400 MHz, $CDCl_3$) of **30**

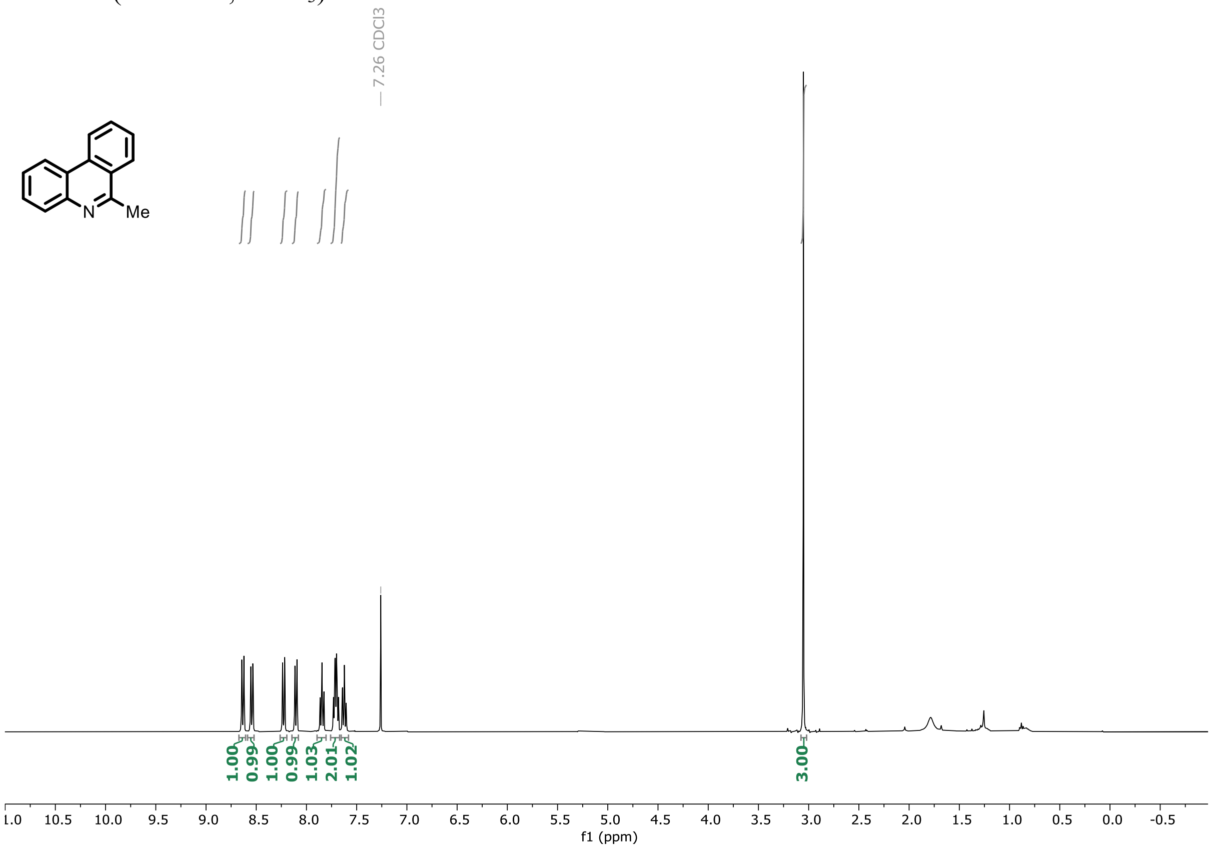

^{13}C NMR (101 MHz, $CDCl_3$) of **30**

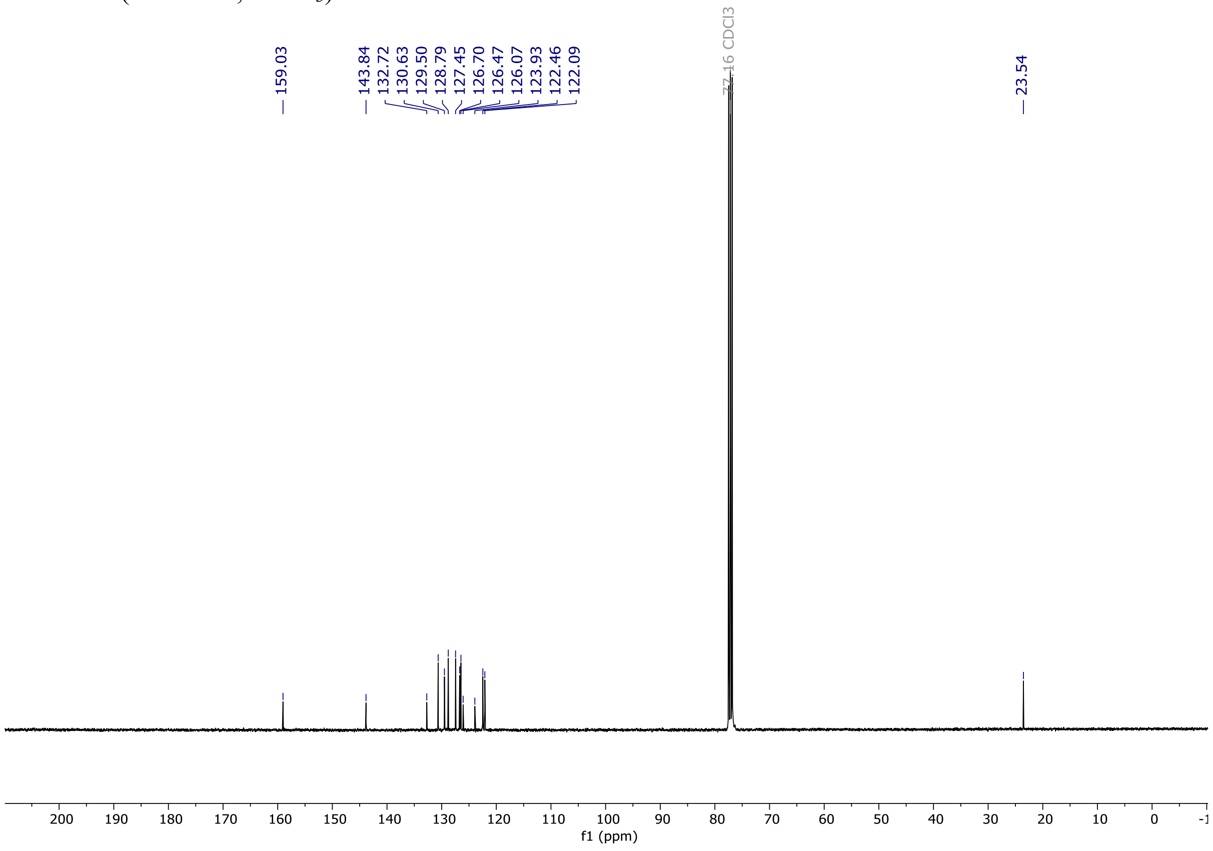

^{1}H NMR (400 MHz, $CDCl_3$) of **31**

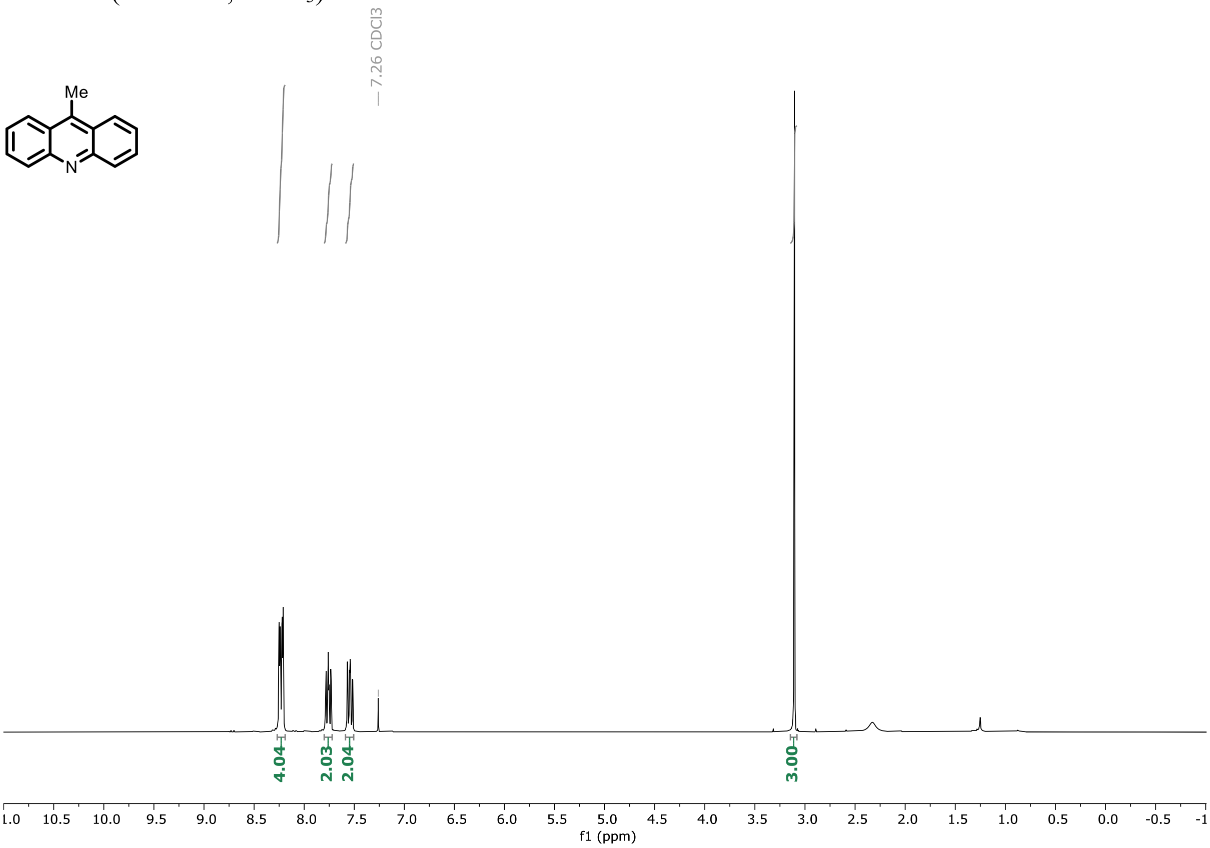

^{13}C NMR (101 MHz, $CDCl_3$) of **31**

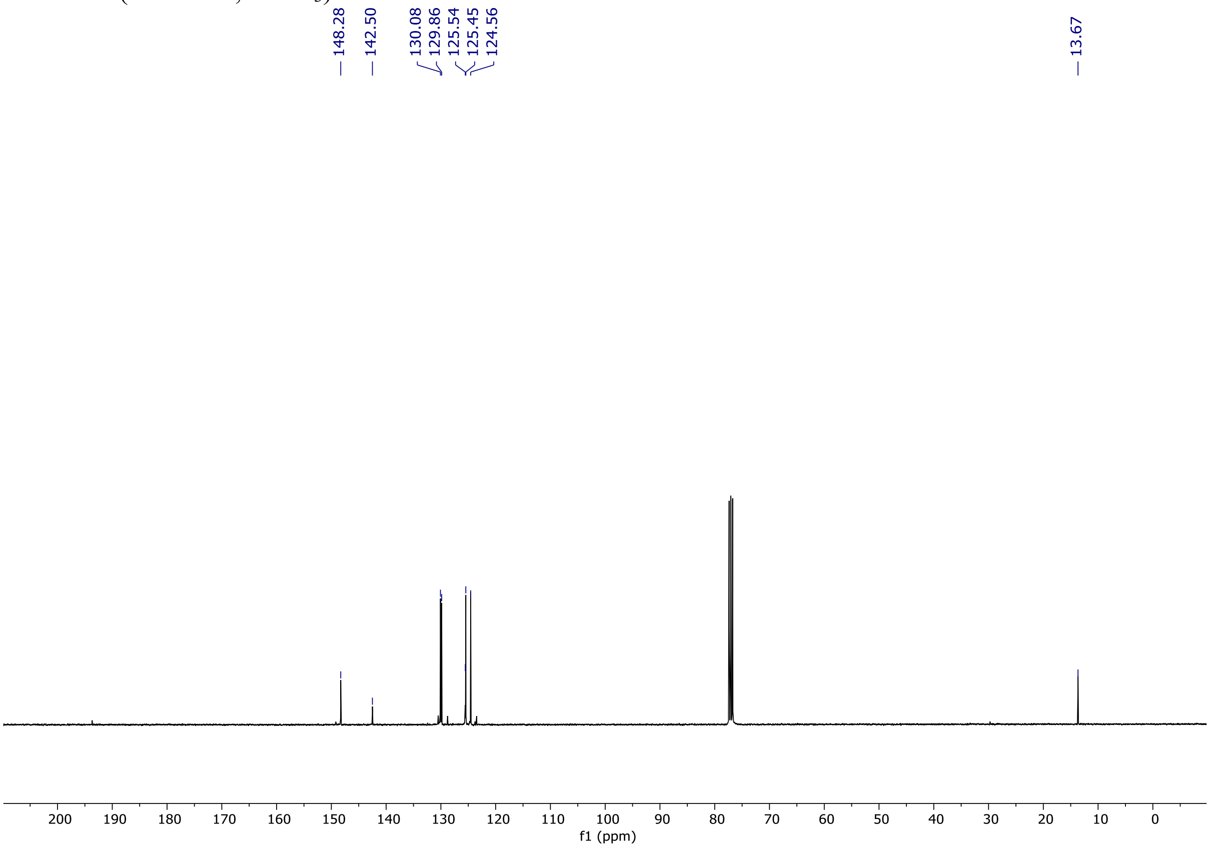

^{1}H NMR (400 MHz, $CDCl_3$) of **32**

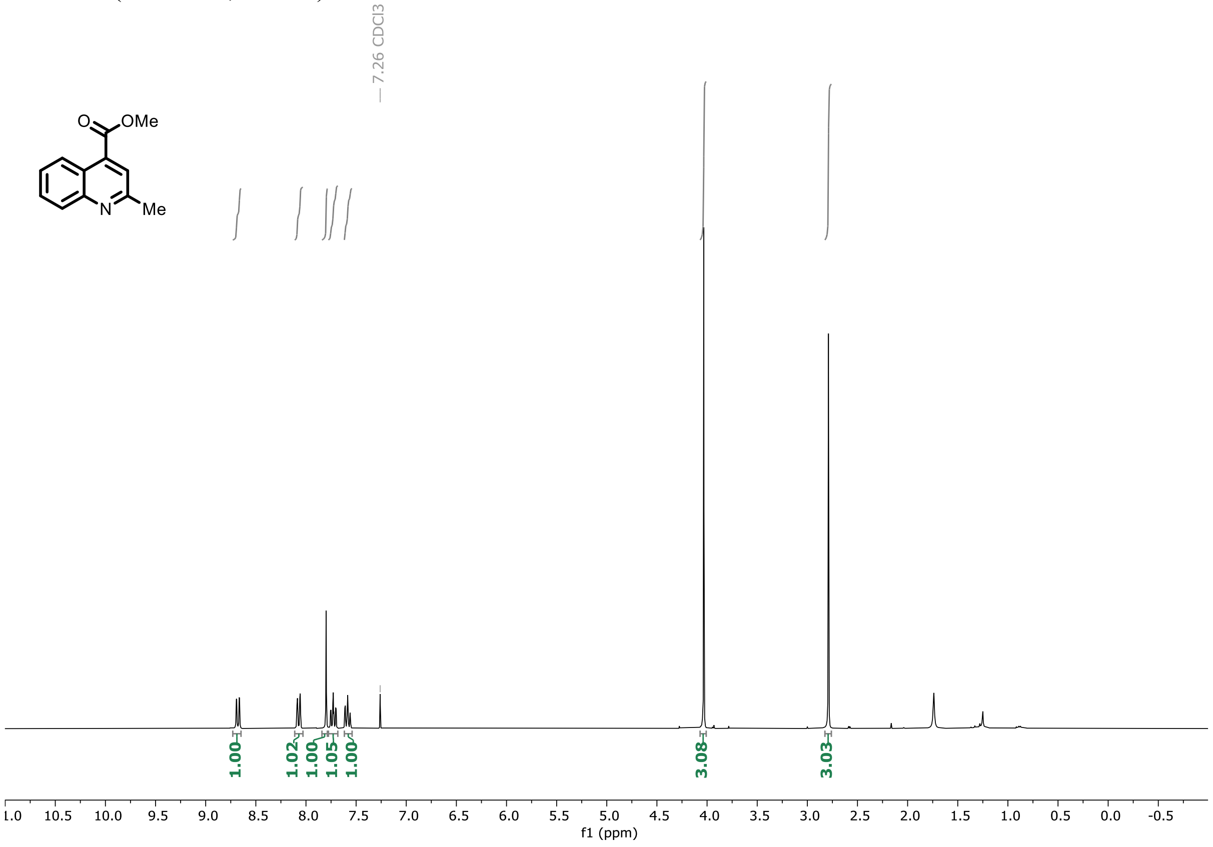

^{13}C NMR (101 MHz, $CDCl_3$) of **32**

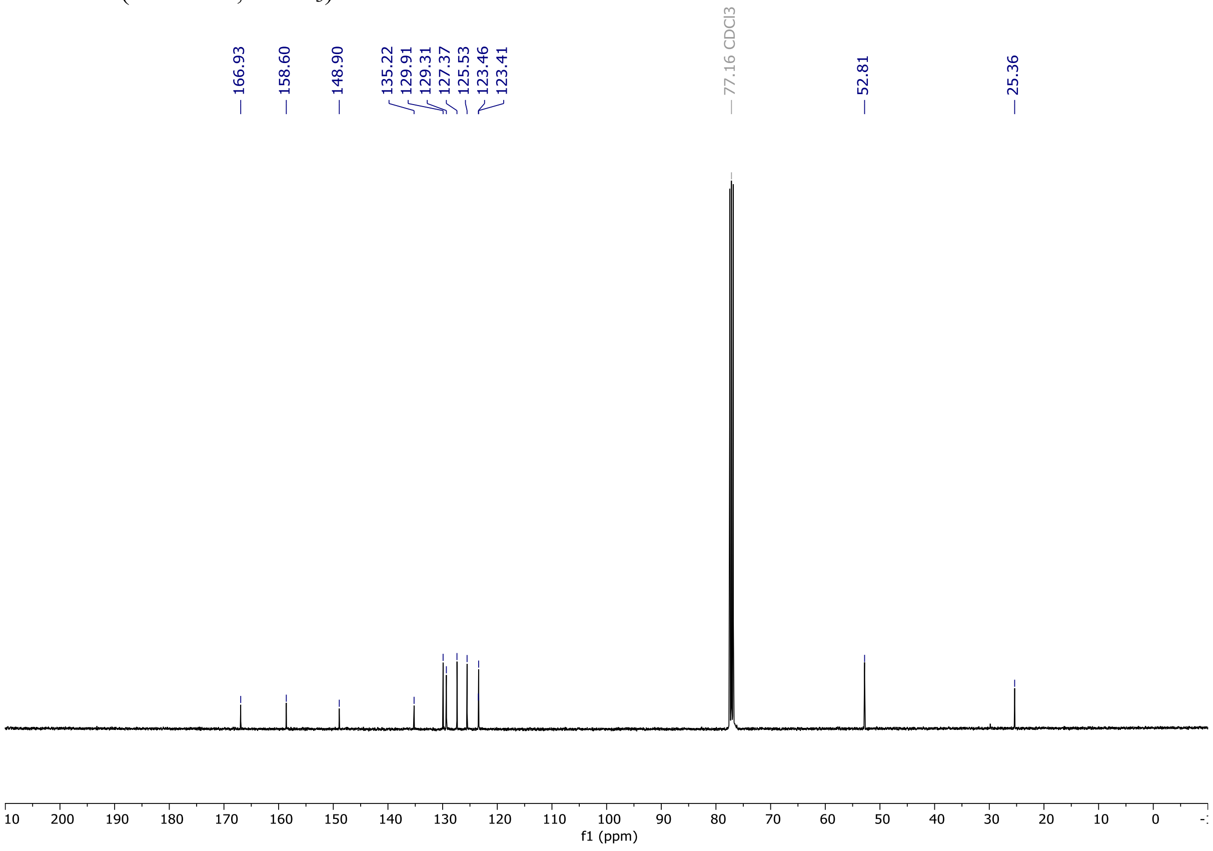

^{1}H NMR (400 MHz, $CDCl_3$) of **33**

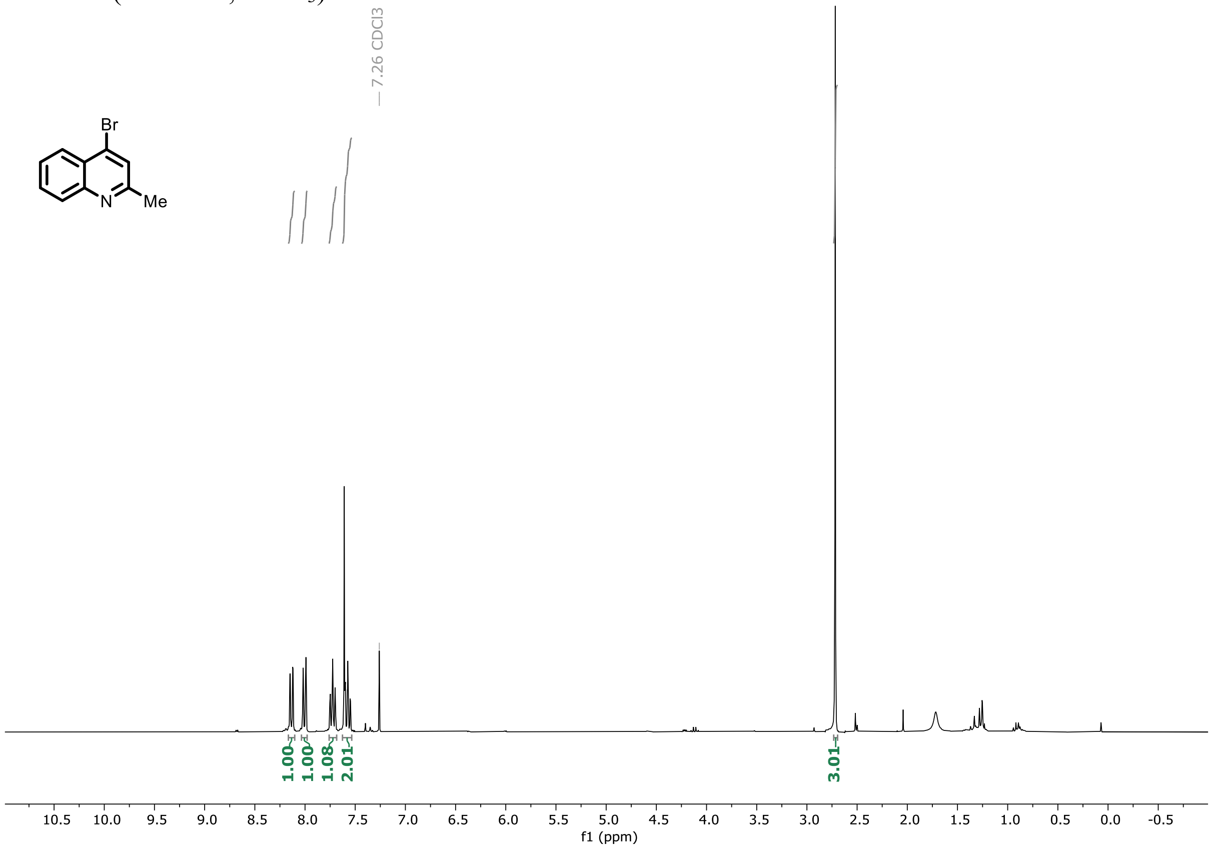

^{13}C NMR (101 MHz, $CDCl_3$) of **33**

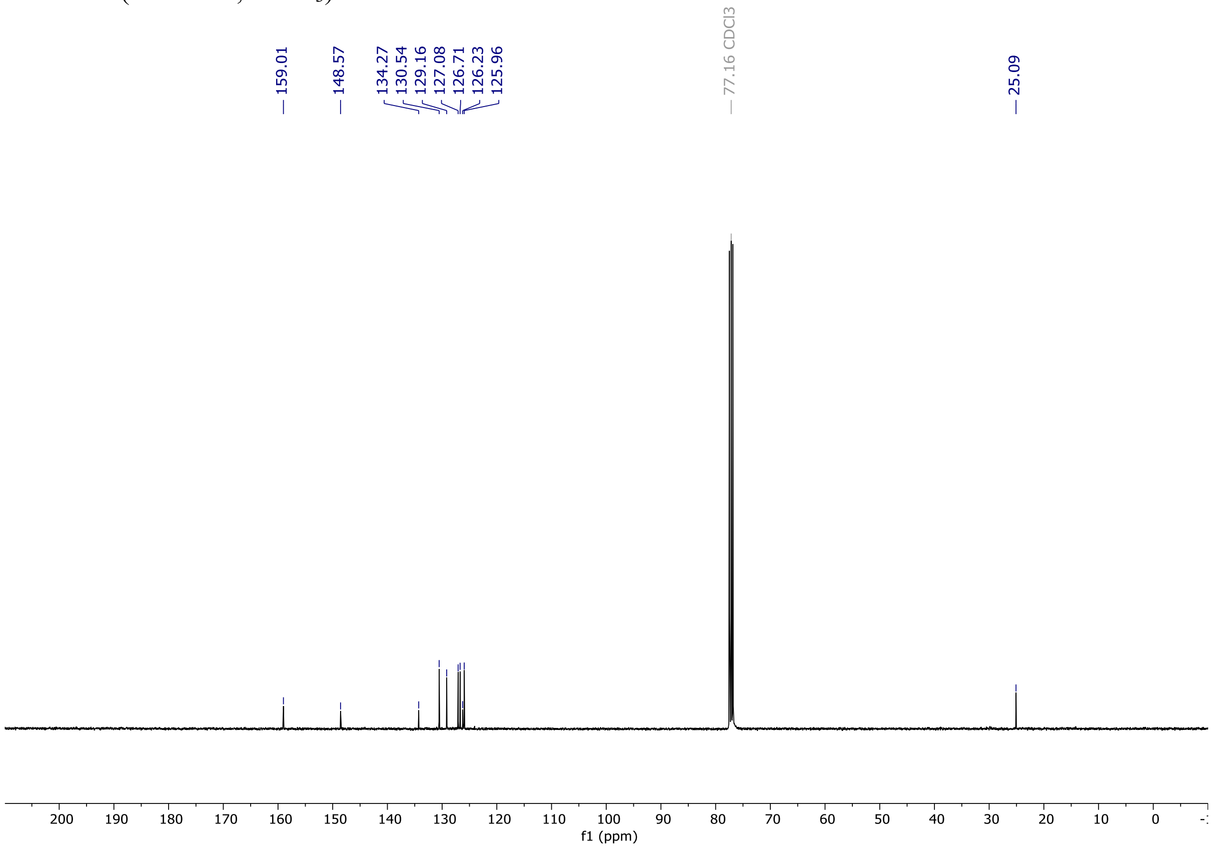

^{1}H NMR (400 MHz, $CDCl_3$) of **34**

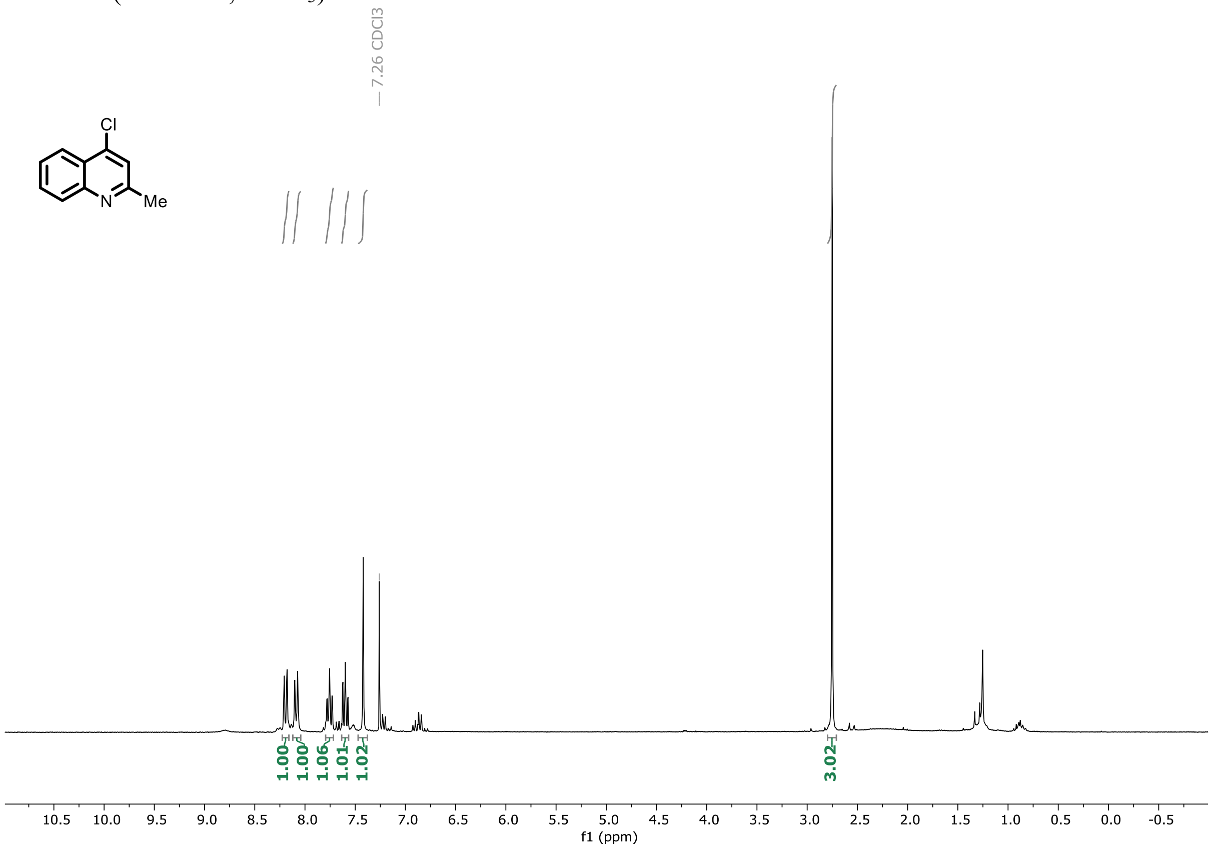

^{13}C NMR (101 MHz, $CDCl_3$) of **34**

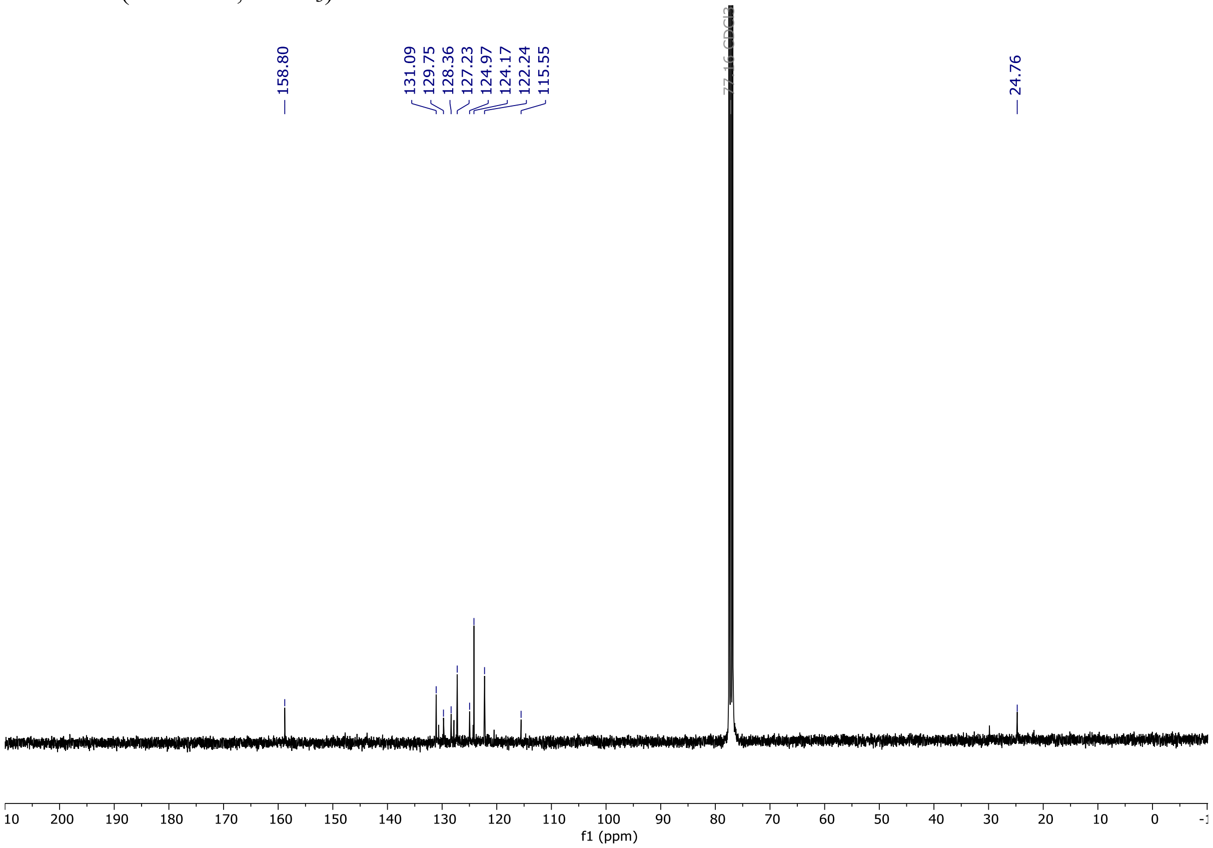

^{1}H NMR (400 MHz, $CDCl_3$) of **35**

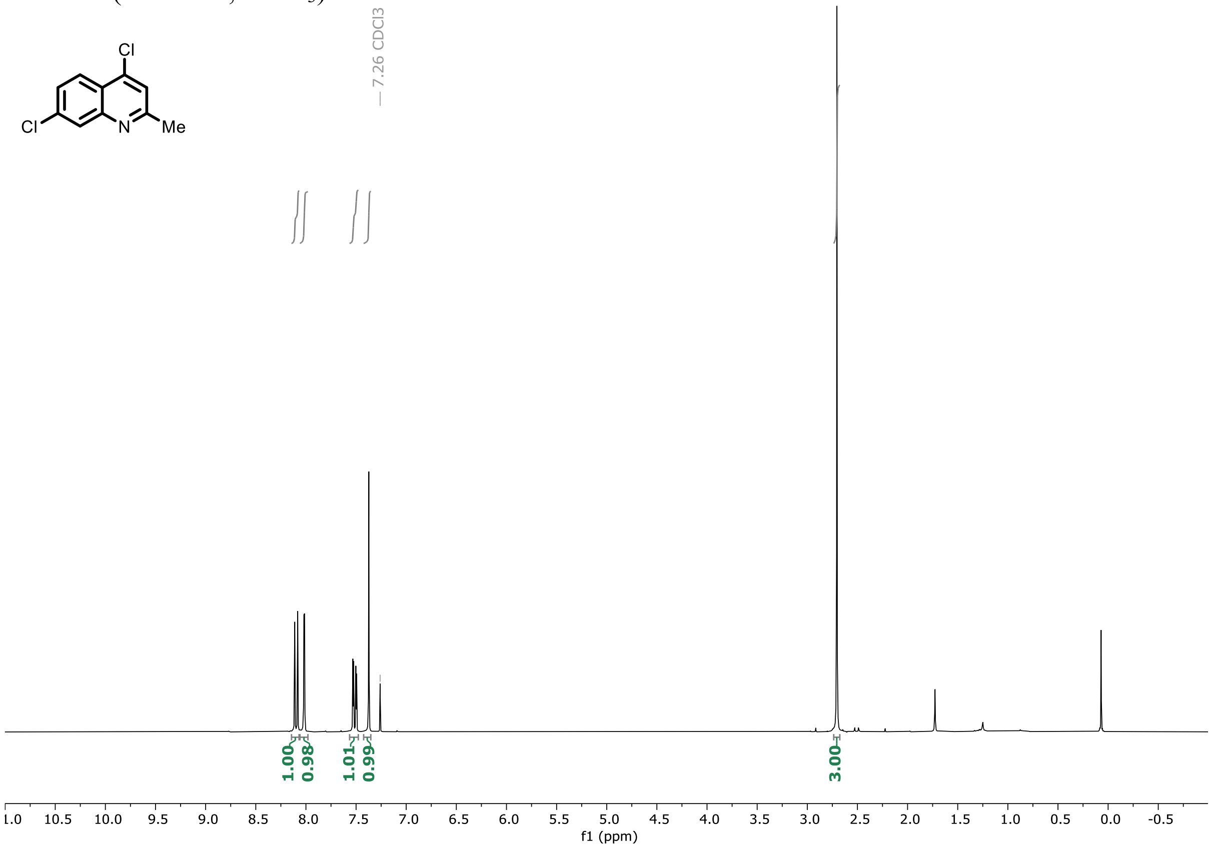

^{13}C NMR (101 MHz, $CDCl_3$) of **35**

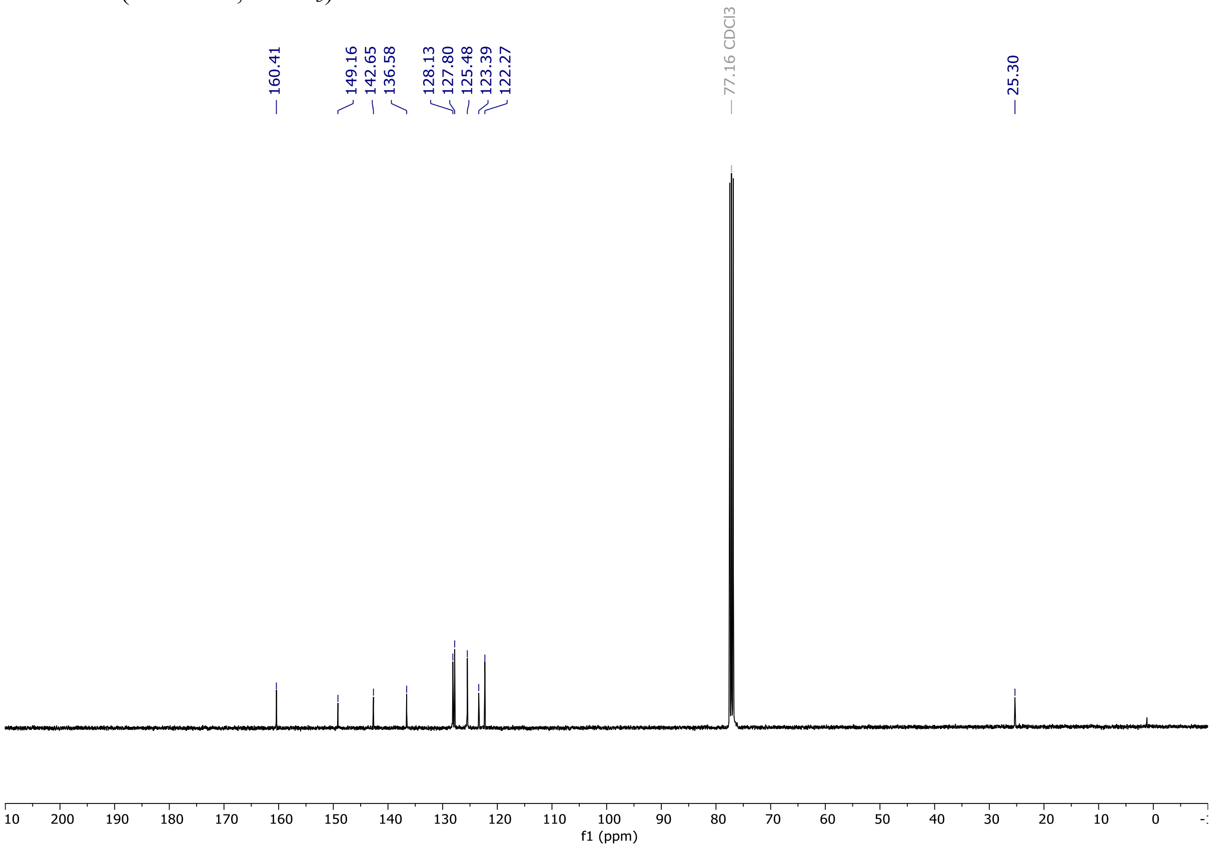

^{1}H NMR (400 MHz, $CDCl_3$) of **36**

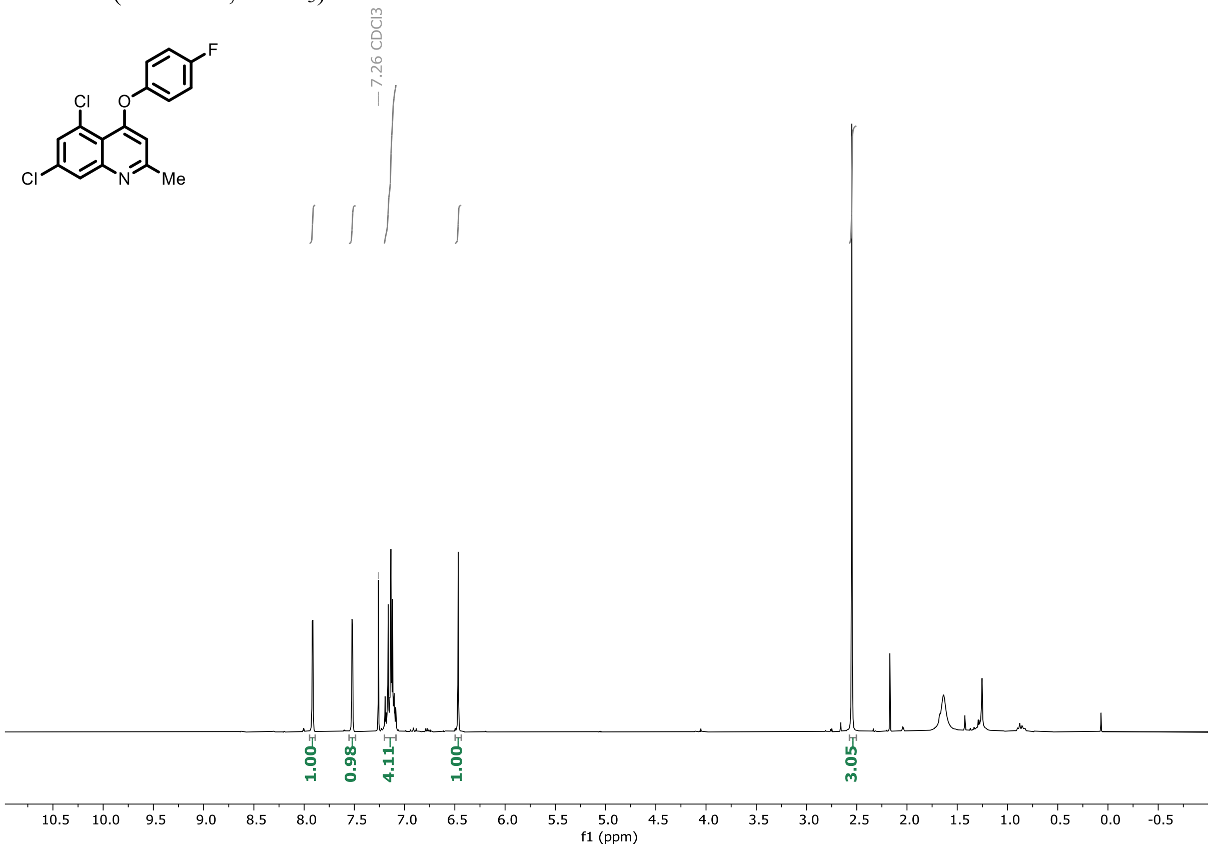

^{13}C NMR (101 MHz, $CDCl_3$) of **36**

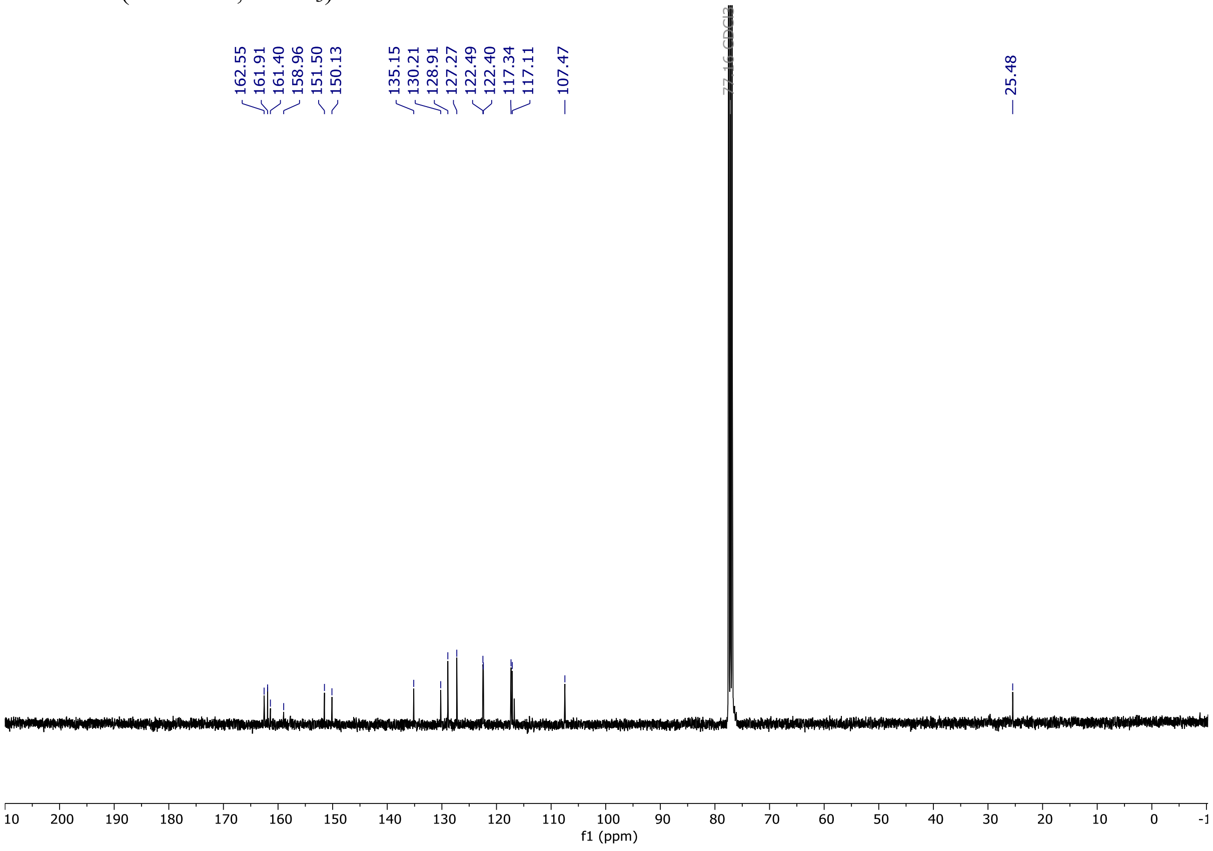

^{19}F NMR (282 MHz, $CDCl_3$) of **36**

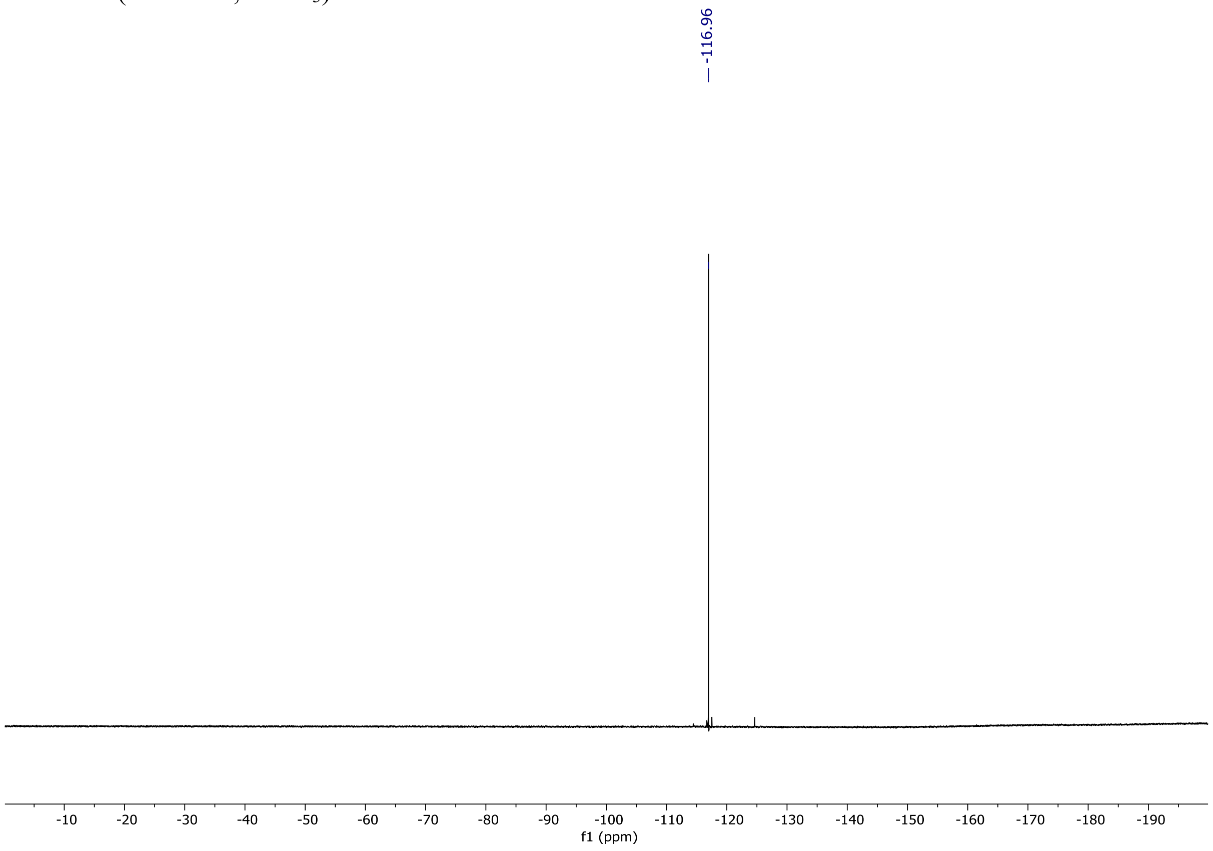

^{1}H NMR (400 MHz, $CDCl_3$) of **37**

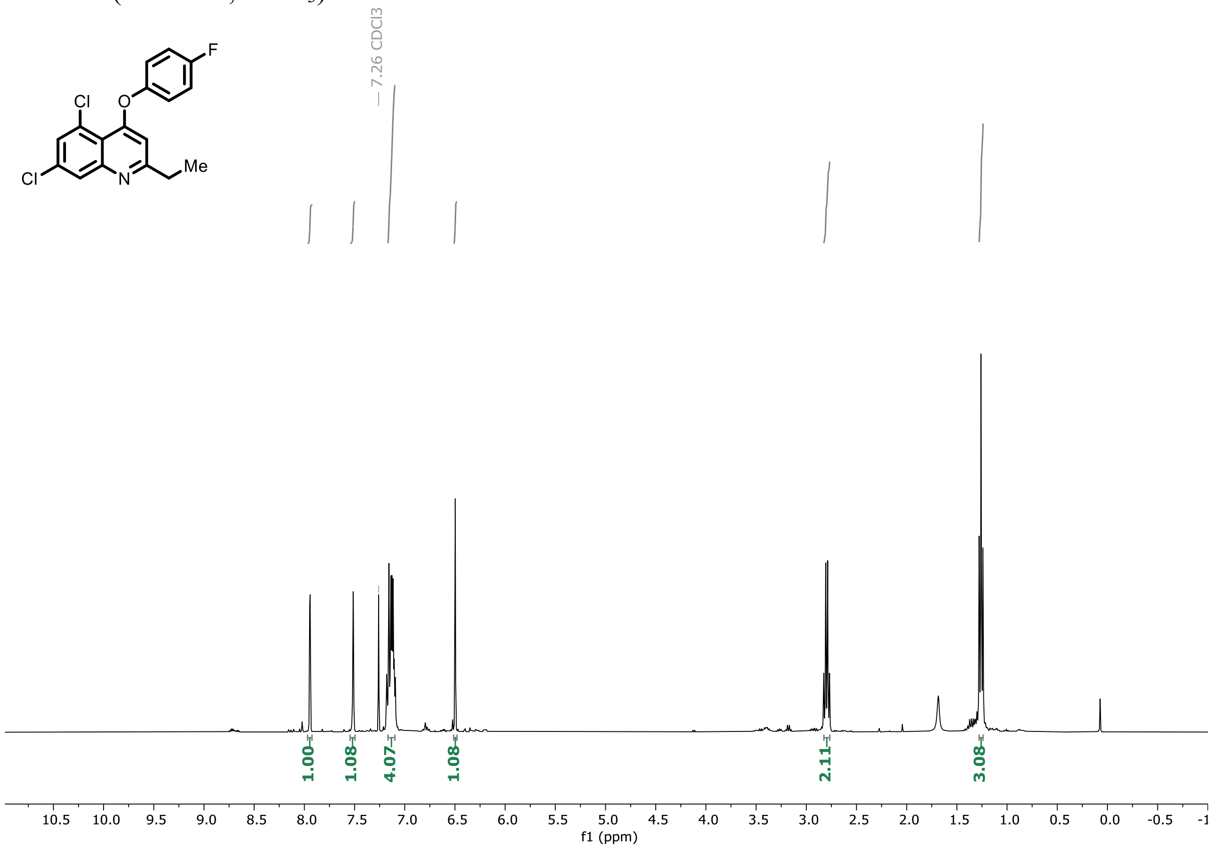

^{13}C NMR (101 MHz, $CDCl_3$) of **37**

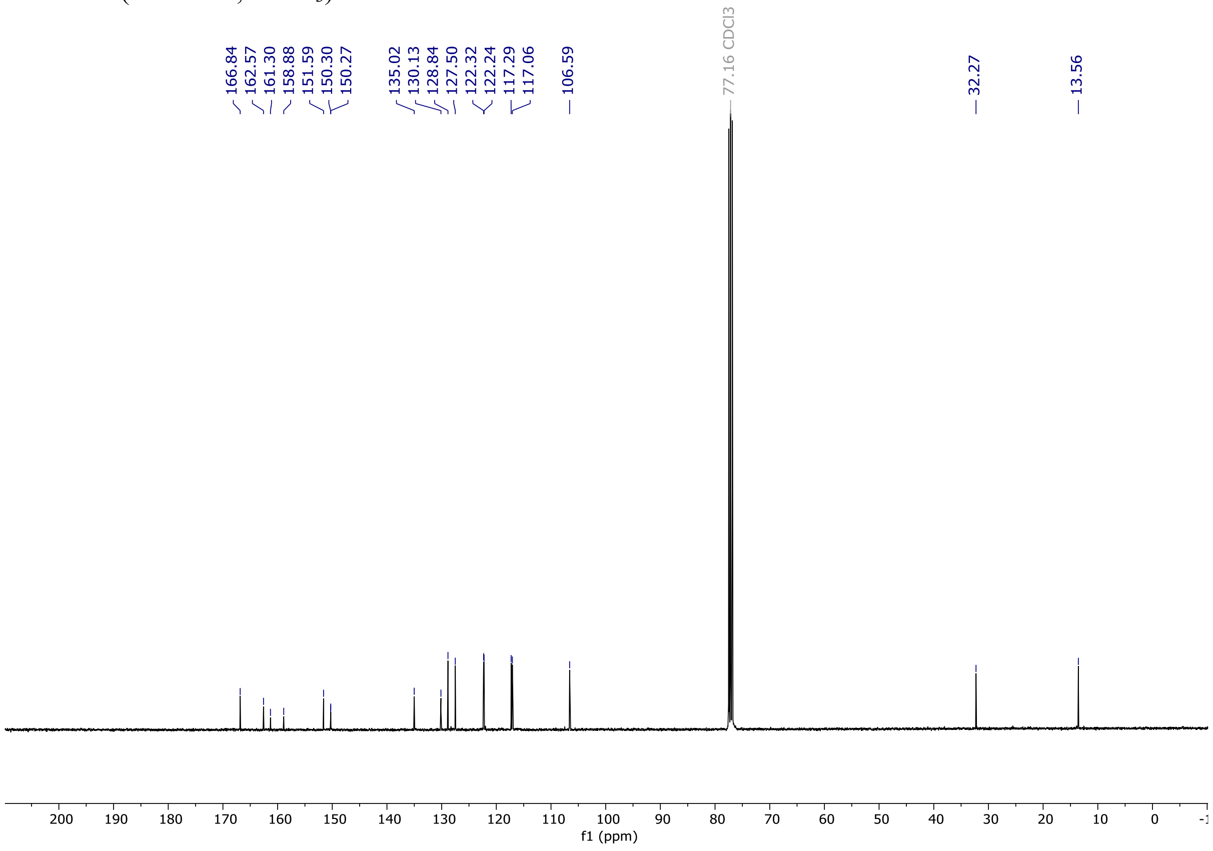

^{19}F NMR (282 MHz, $CDCl_3$) of **37**

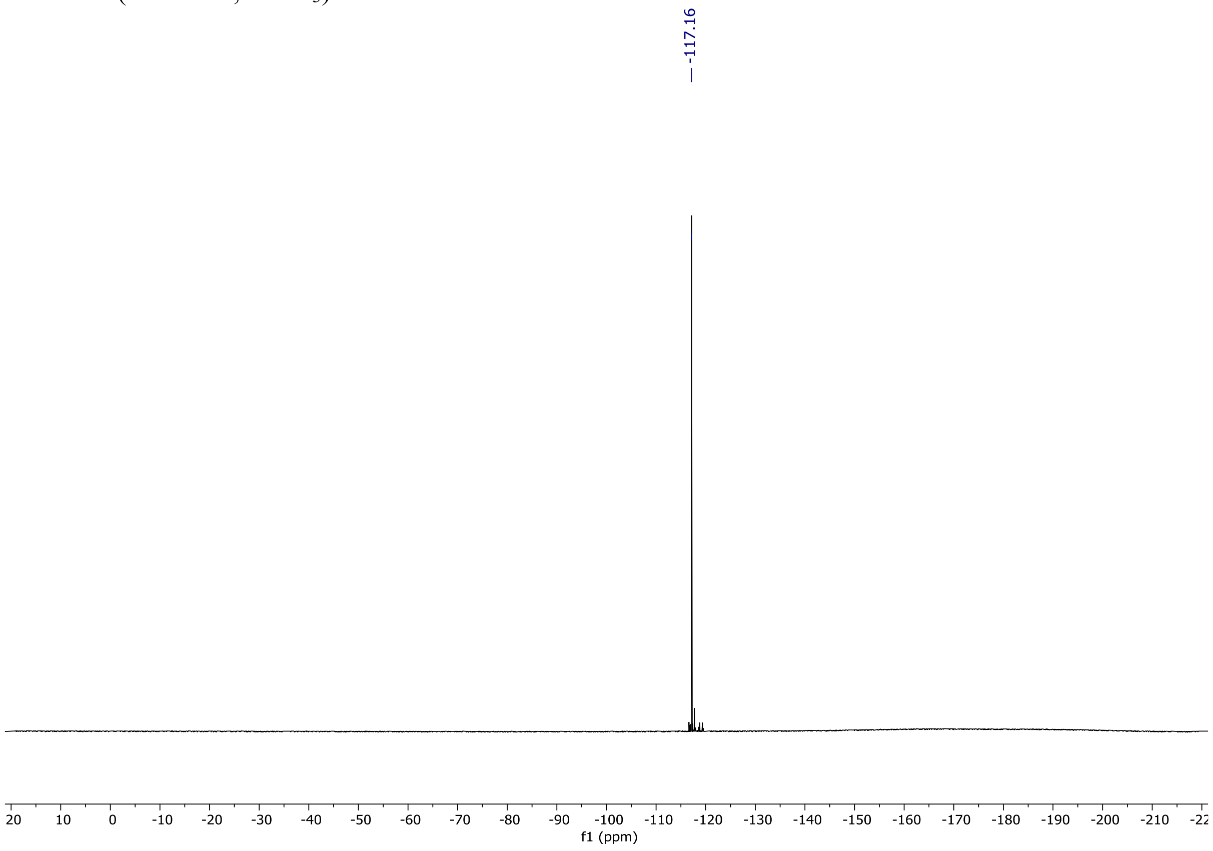

^{1}H NMR (400 MHz, $CDCl_3$) of **38**

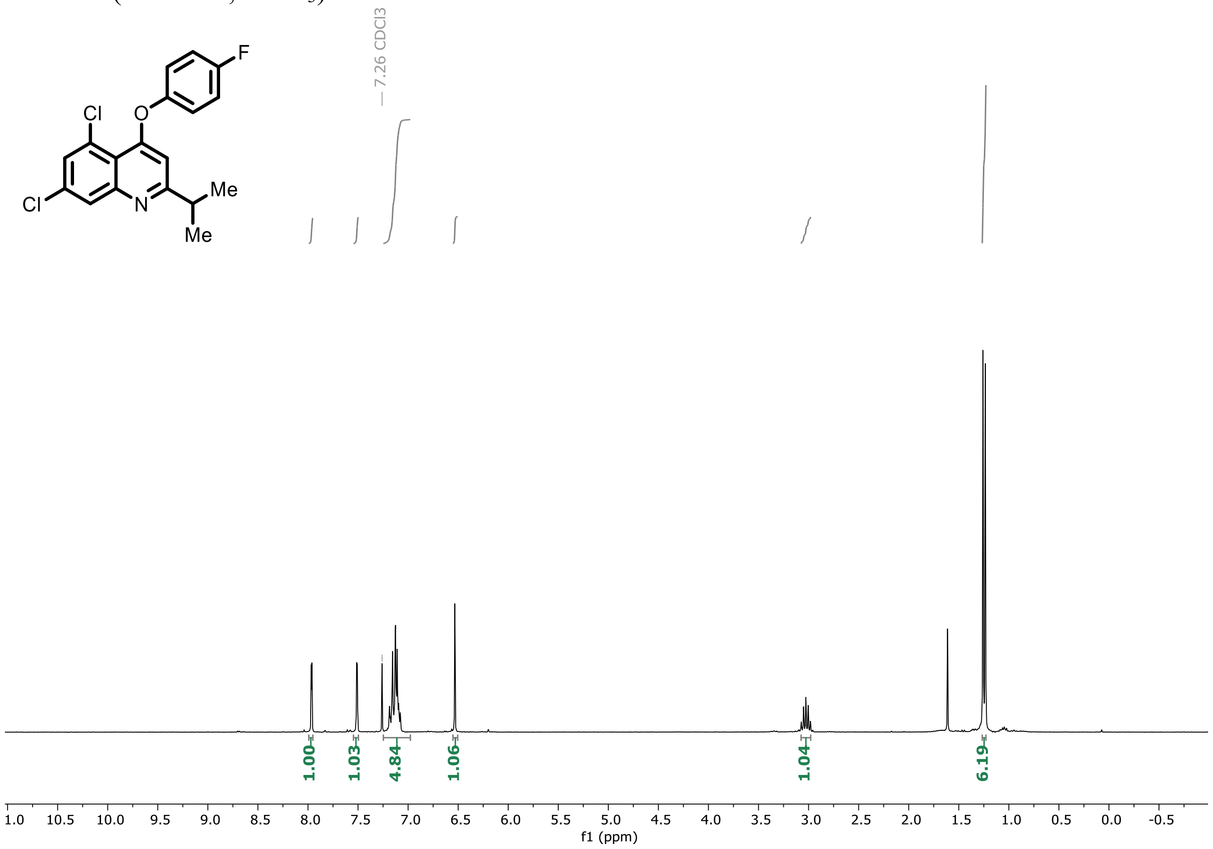

^{13}C NMR (101 MHz, $CDCl_3$) of **38**

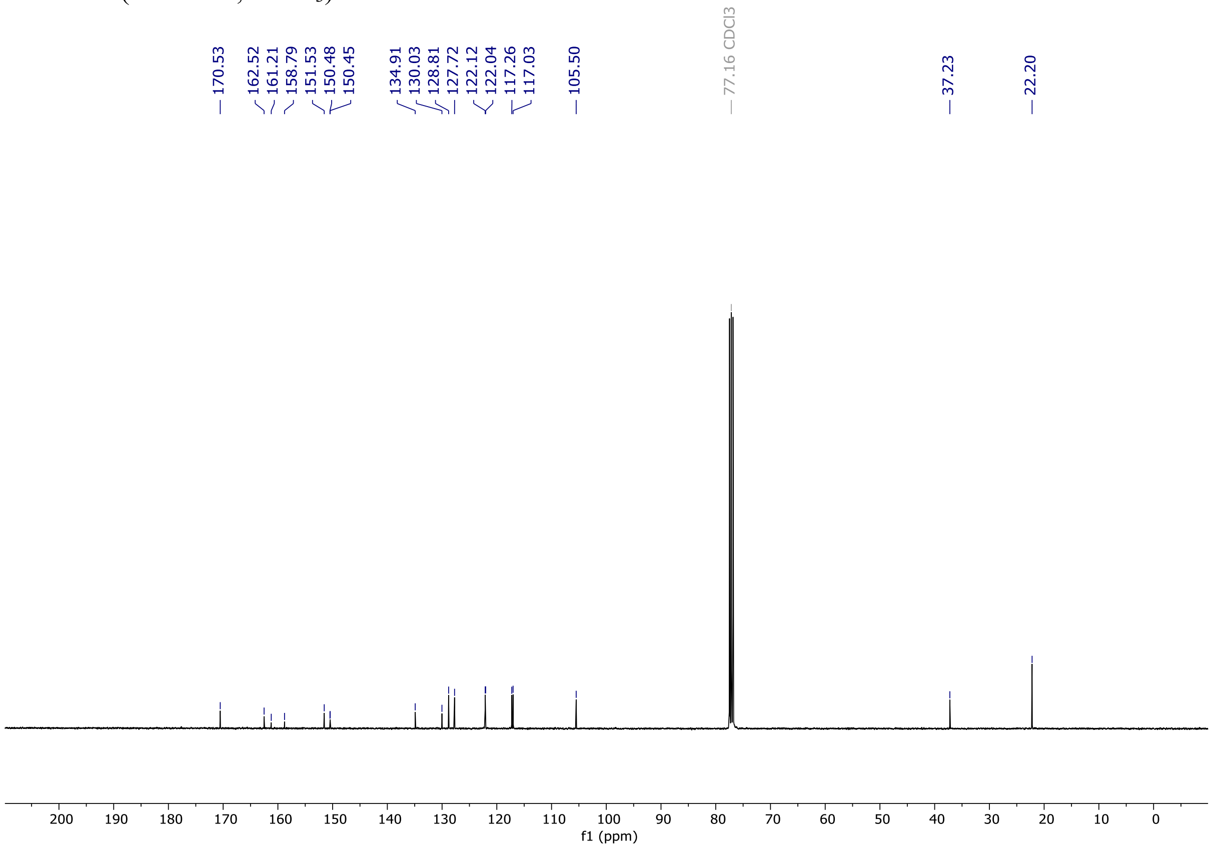

^{19}F NMR (282 MHz, $CDCl_3$) of **38**

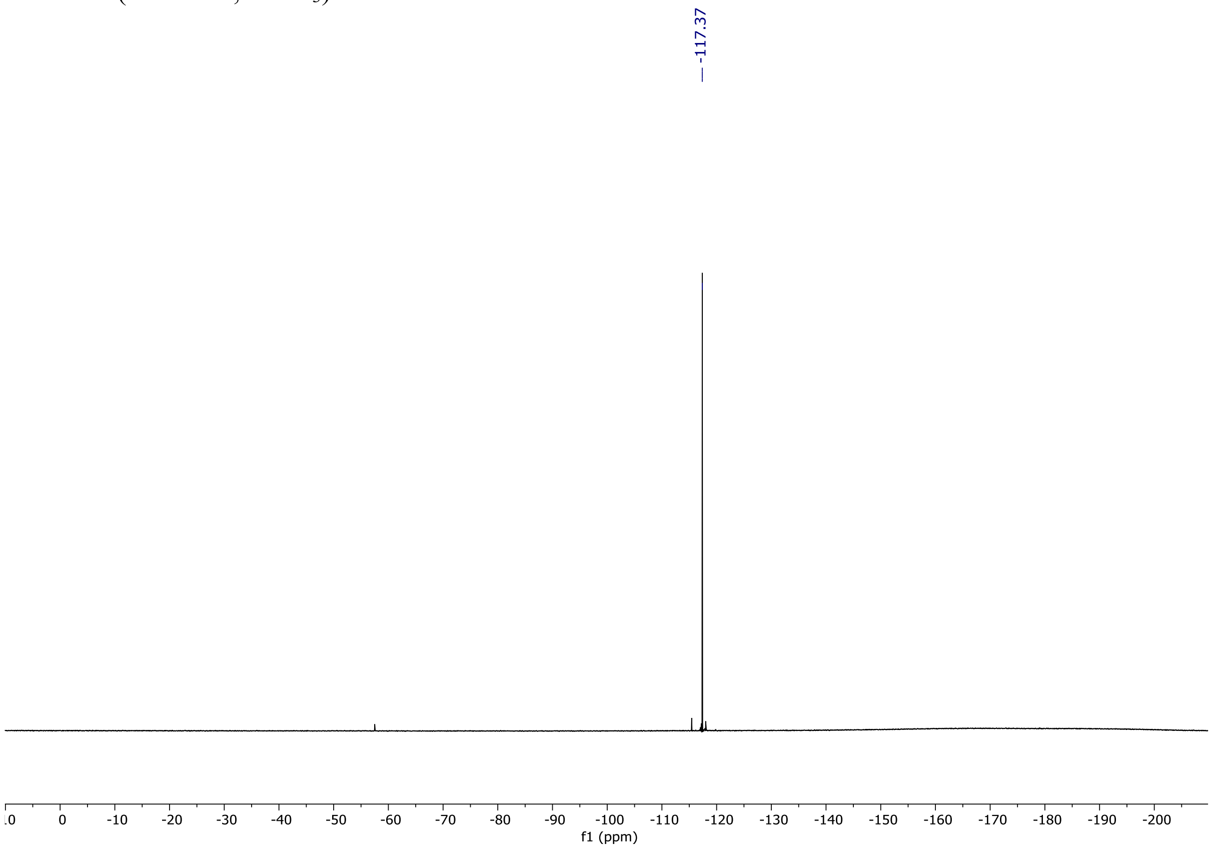

^{1}H NMR (400 MHz, $CDCl_3$) of **39**

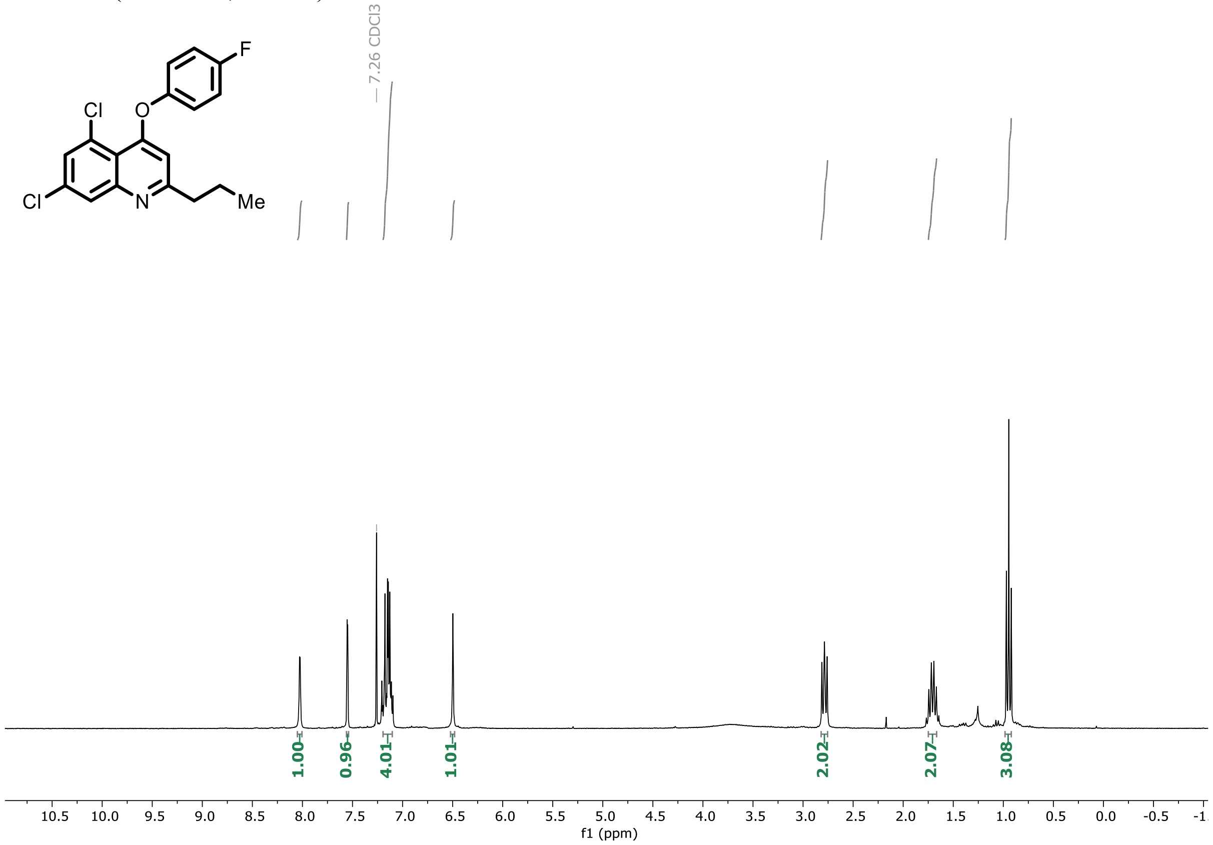

^{13}C NMR (101 MHz, $CDCl_3$) of **39**

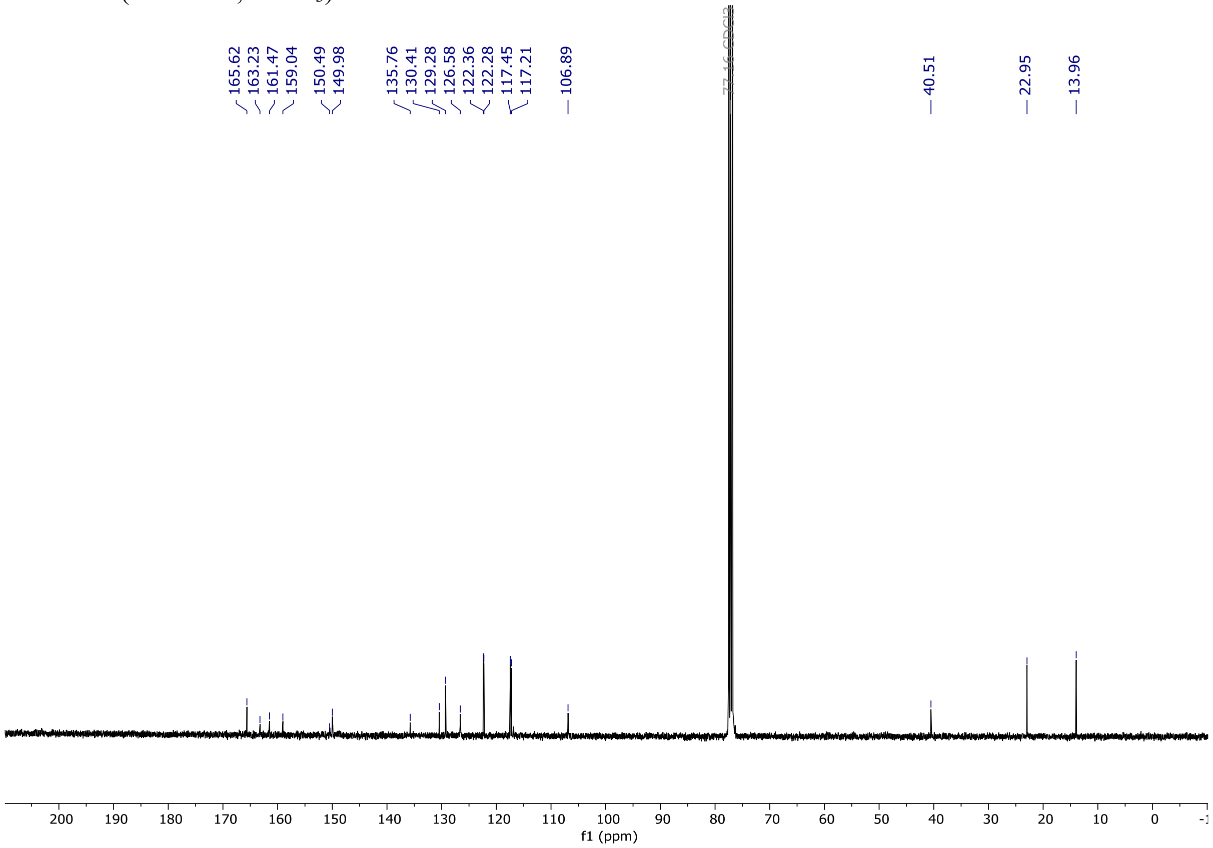

^{19}F NMR (282 MHz, $CDCl_3$) of **39**

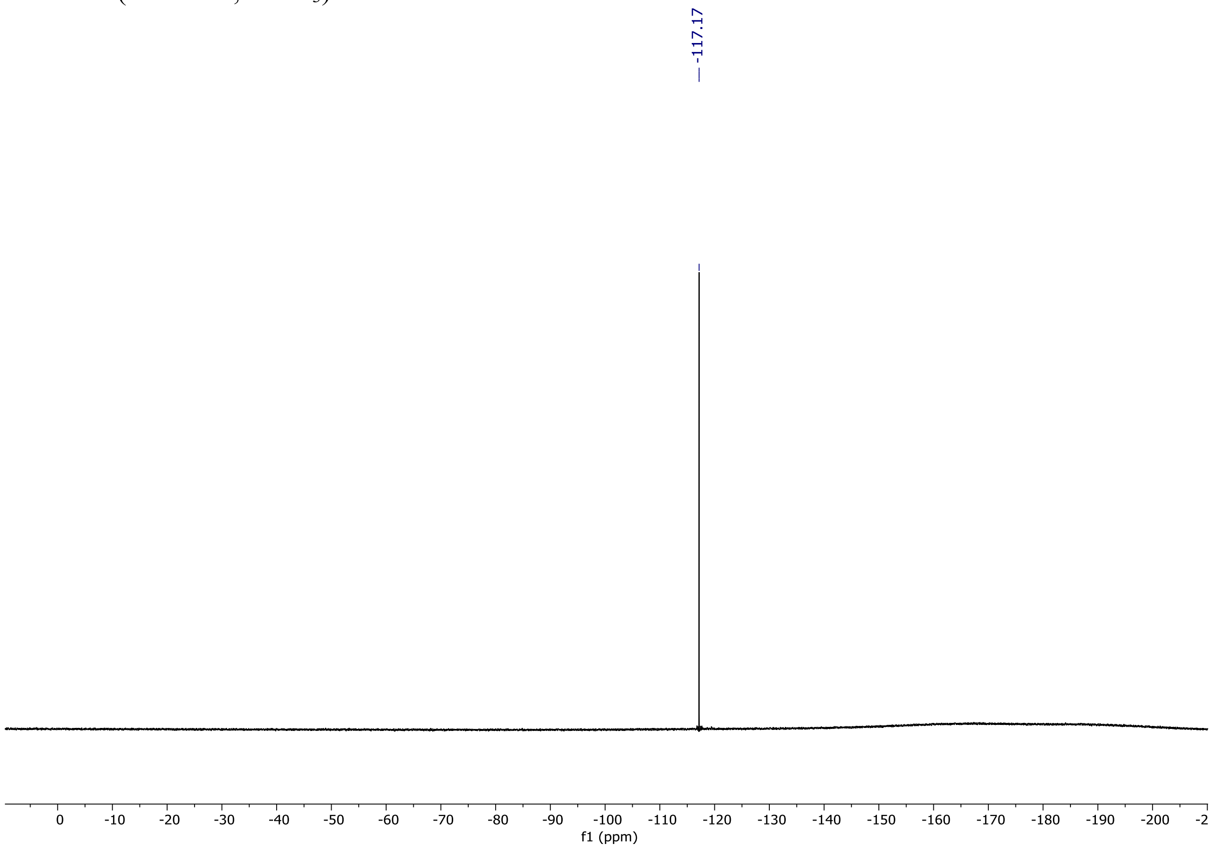

^{1}H NMR (400 MHz, $CDCl_3$) of **40**

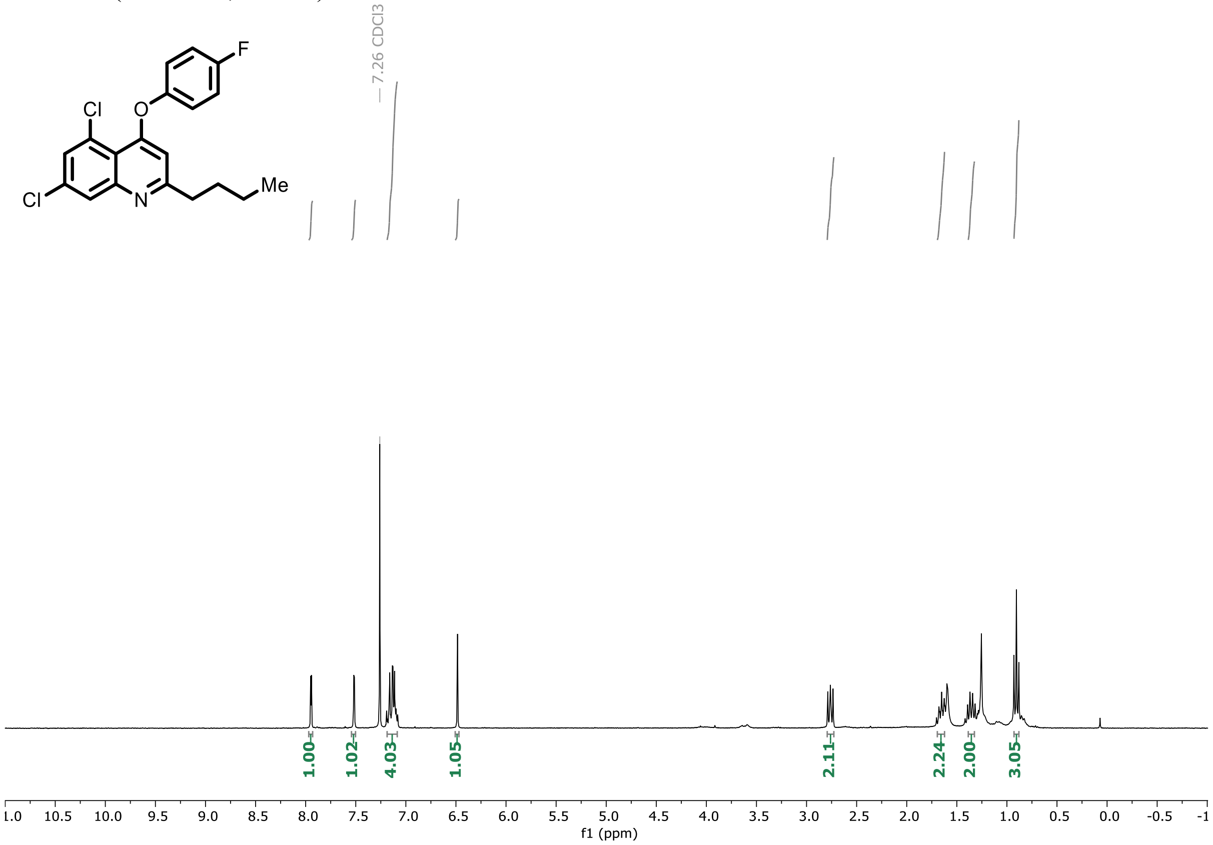

^{13}C NMR (101 MHz, $CDCl_3$) of **40**

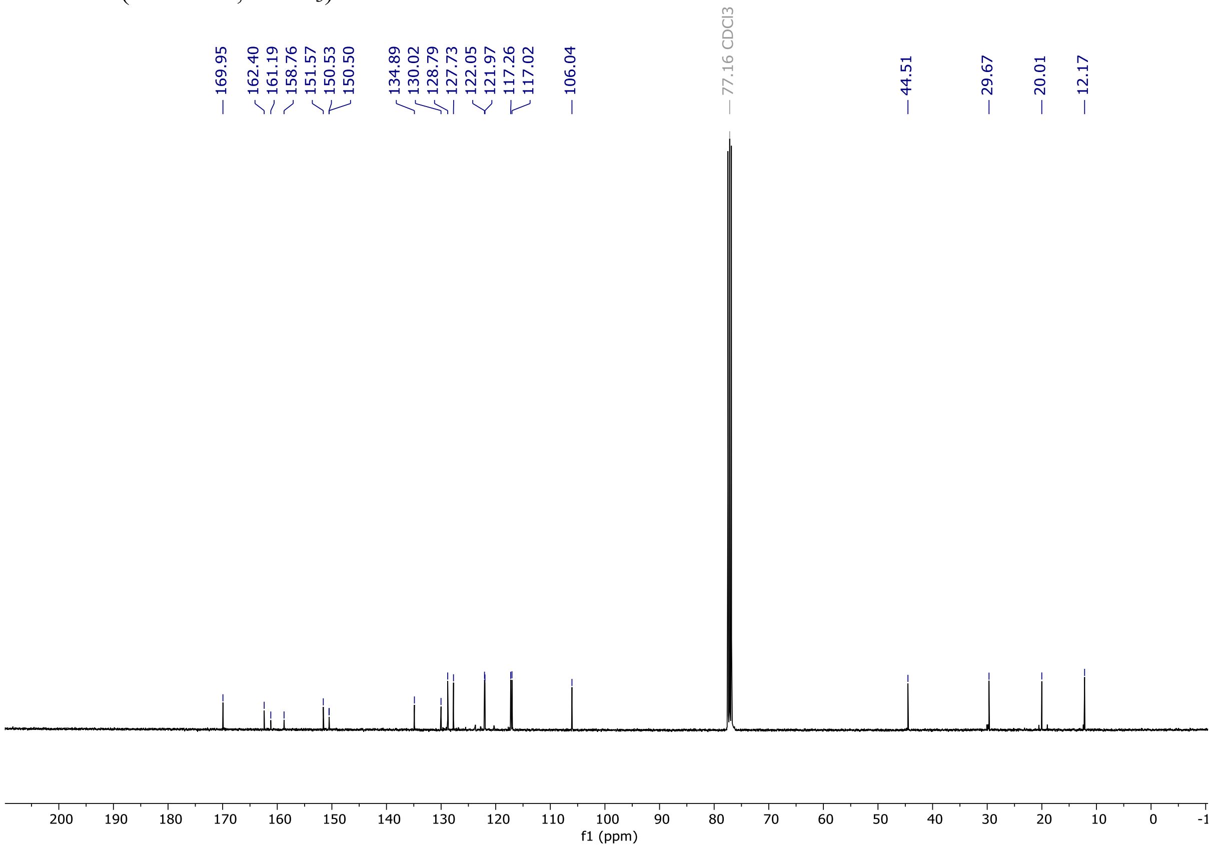

^{19}F NMR (282 MHz, $CDCl_3$) of **40**

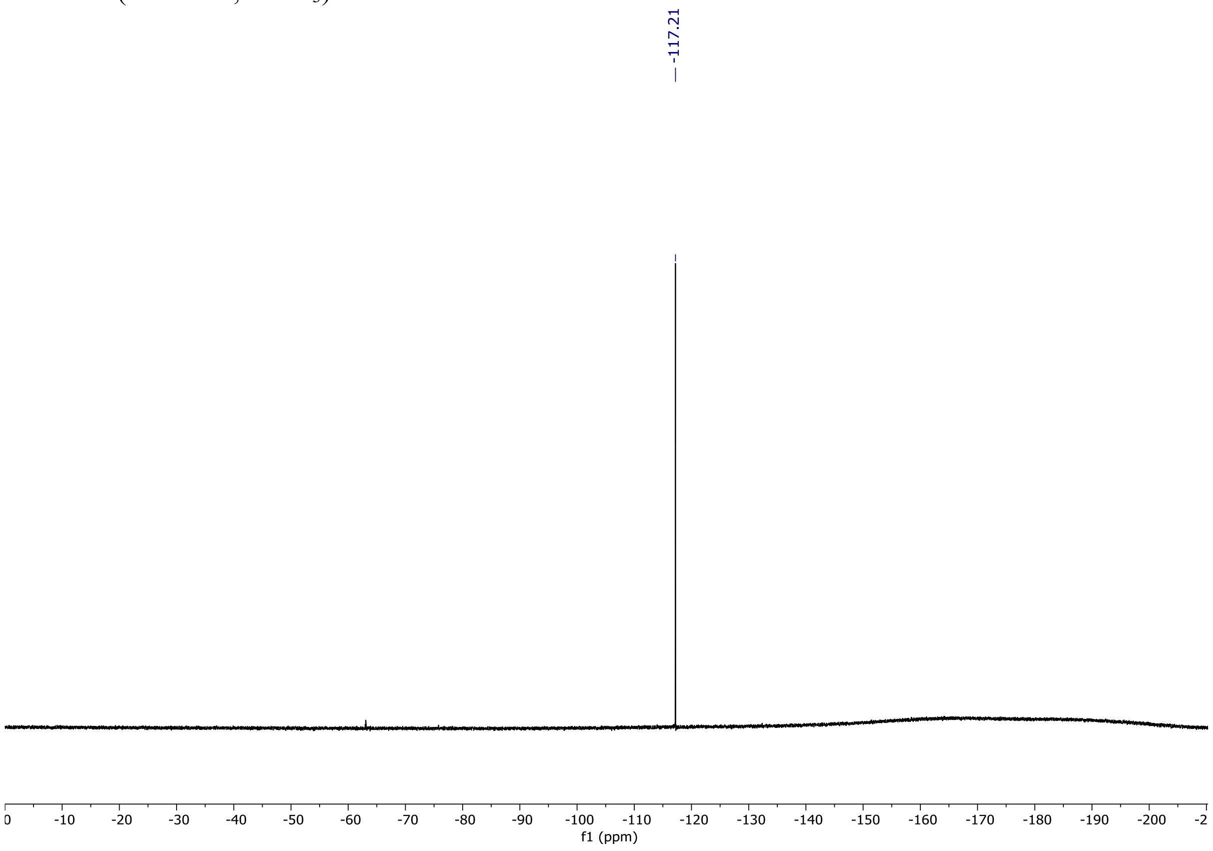

^{1}H NMR (400 MHz, $CDCl_3$) of **41**

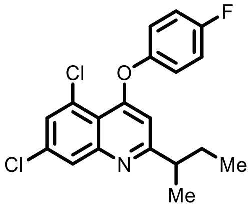

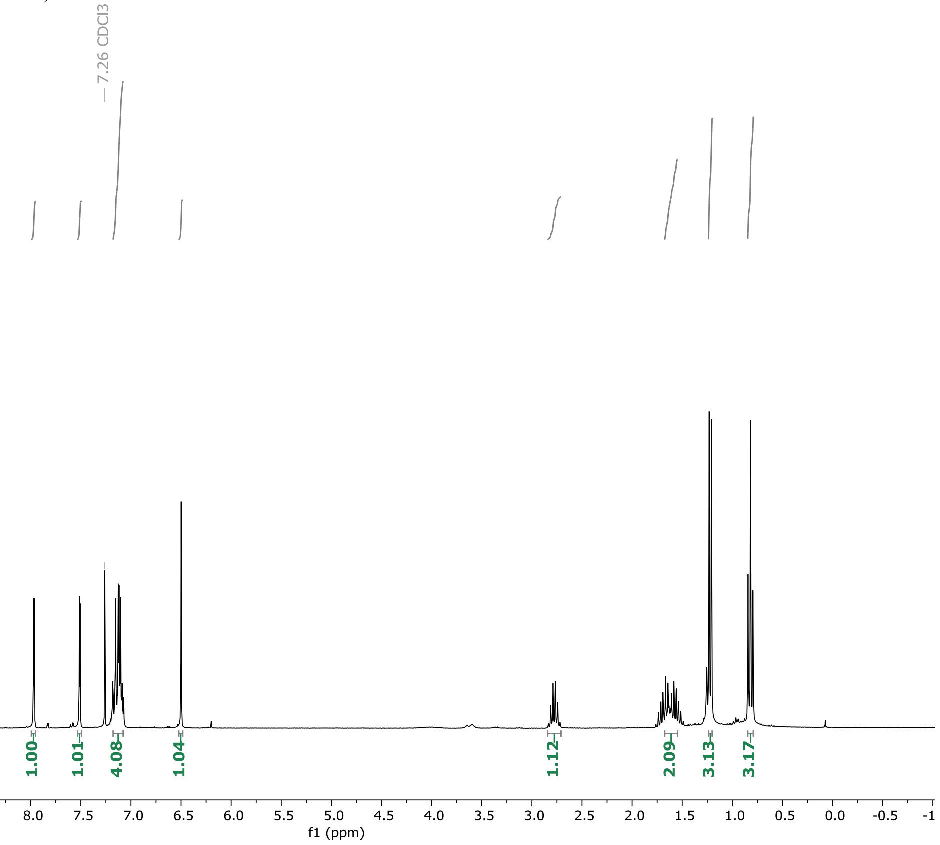

^{13}C NMR (101 MHz, $CDCl_3$) of **41**

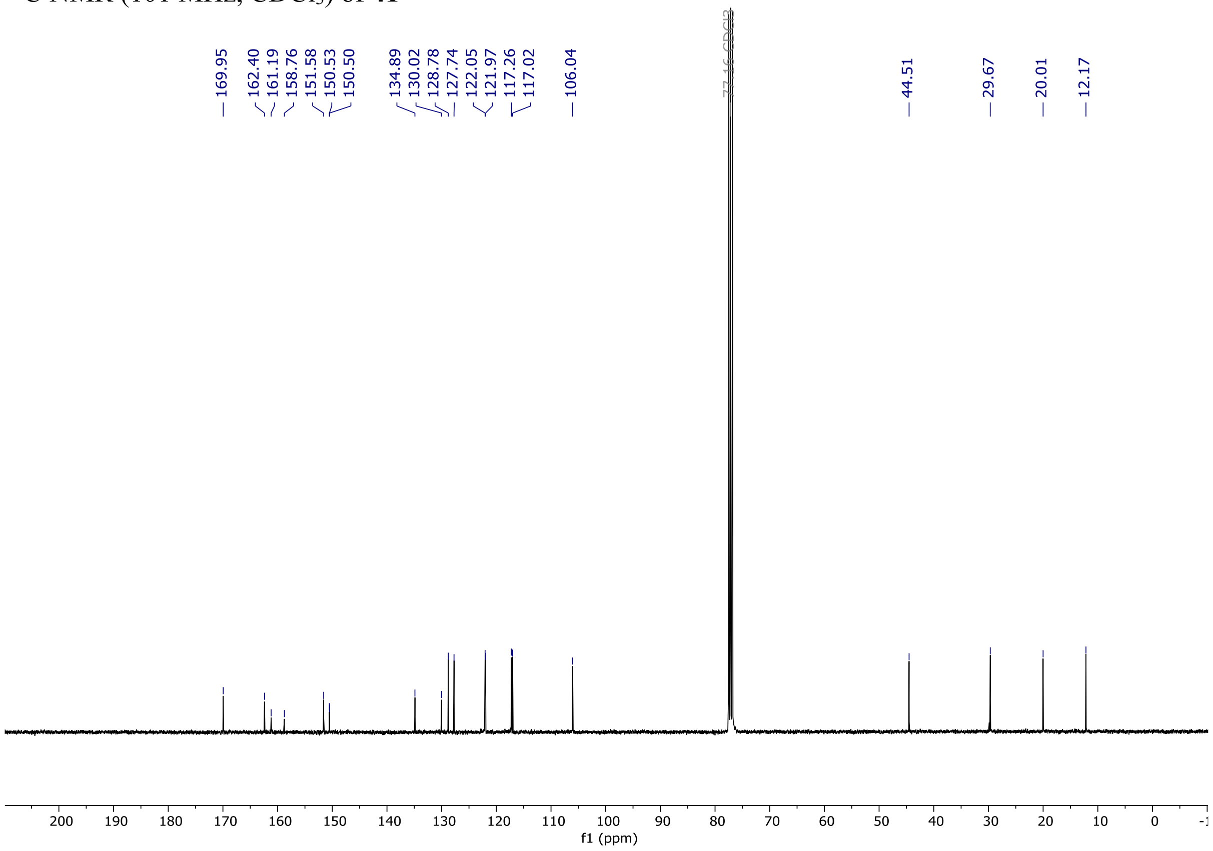

^{19}F NMR (282 MHz, $CDCl_3$) of **41**

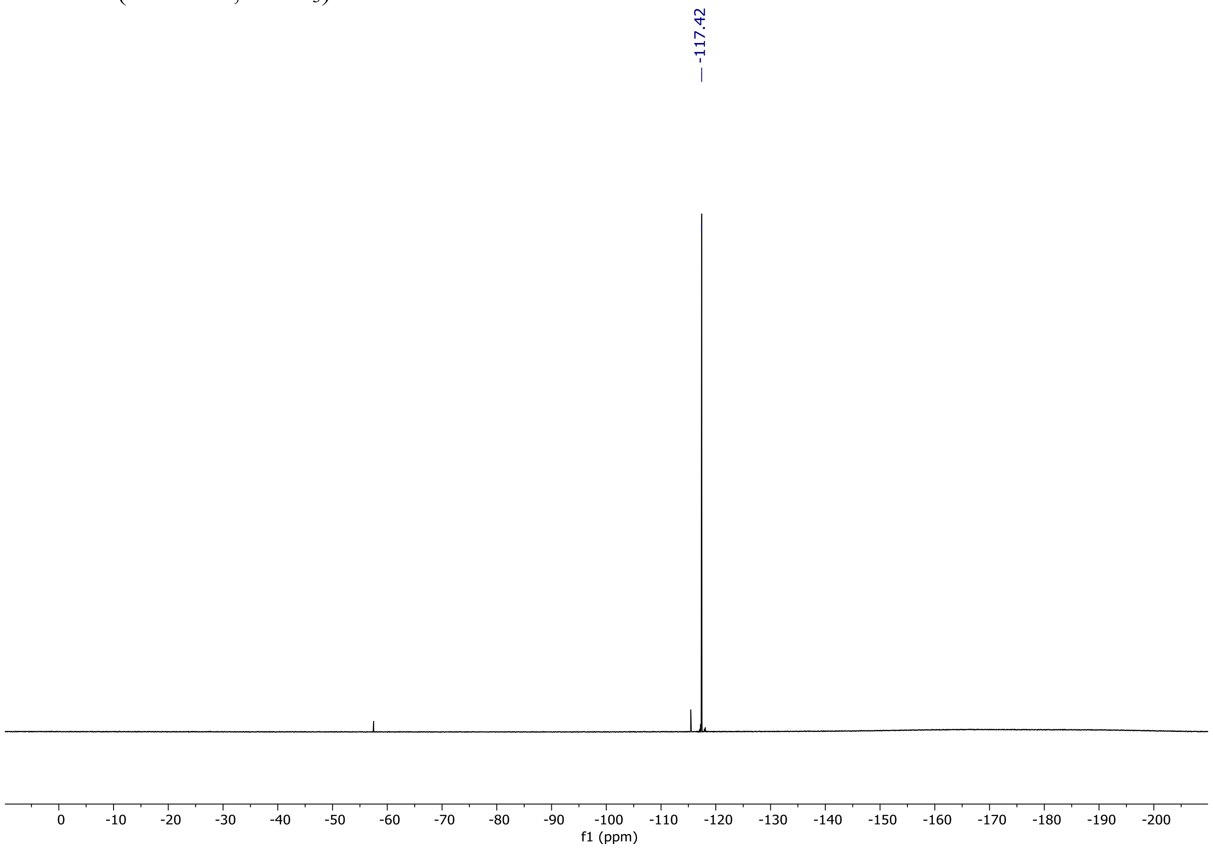

12. GC-MS Data

All the GC-FID data recorded by analysis of the crude reaction mixtures.

GC spectrum of **22**

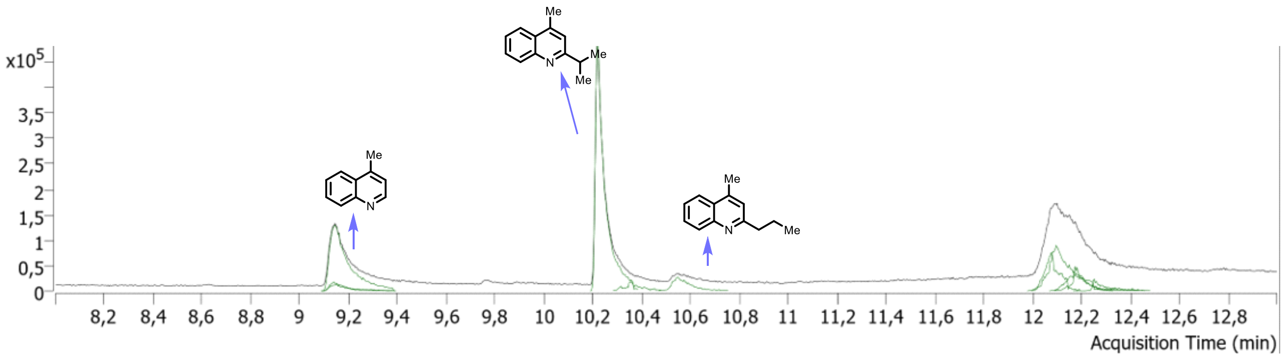

Peak	RT	Peak Height	Peak Area	Area %
1	10.2	268018,9	1166279,9	92 %
2	10.5	11444,6	100897,8	8 %

GC spectrum of **23**

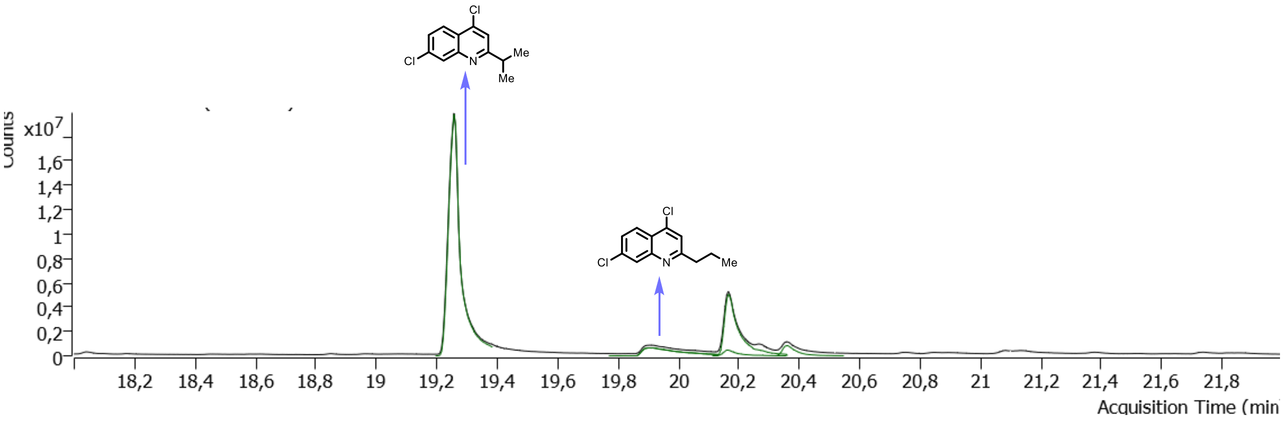

Peak	RT	Peak Height	Peak Area	Area %
1	19.2	10141135,7	57865451,2	91.4 %
2	19.9	419874,5	5504149,0	8.6 %

GC spectrum of **26**

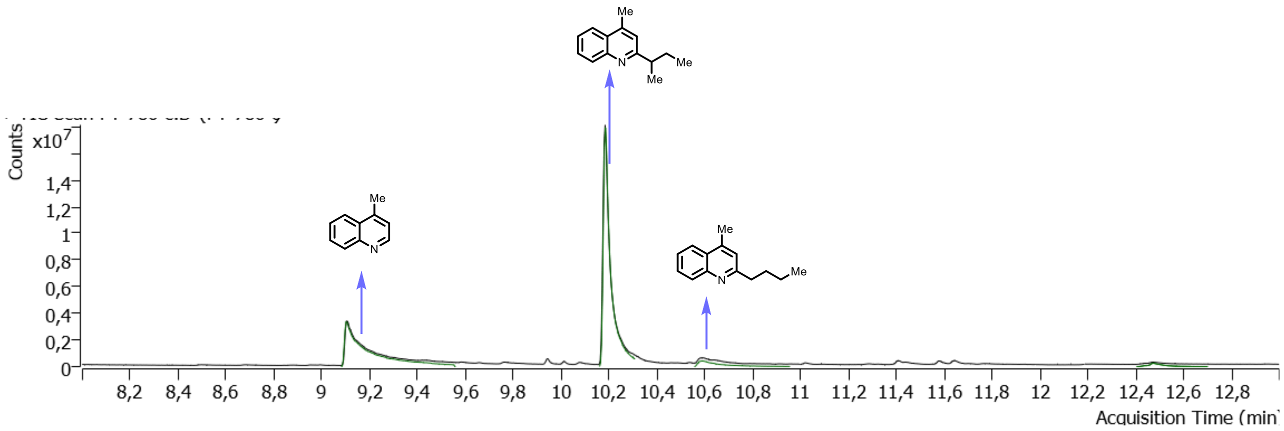

Peak	RT	Peak Height	Peak Area	Area %
1	10.1	9946954,6	41069467,1	96 %
2	10.6	232308,1	1748534,6	4 %

GC spectrum of **27**

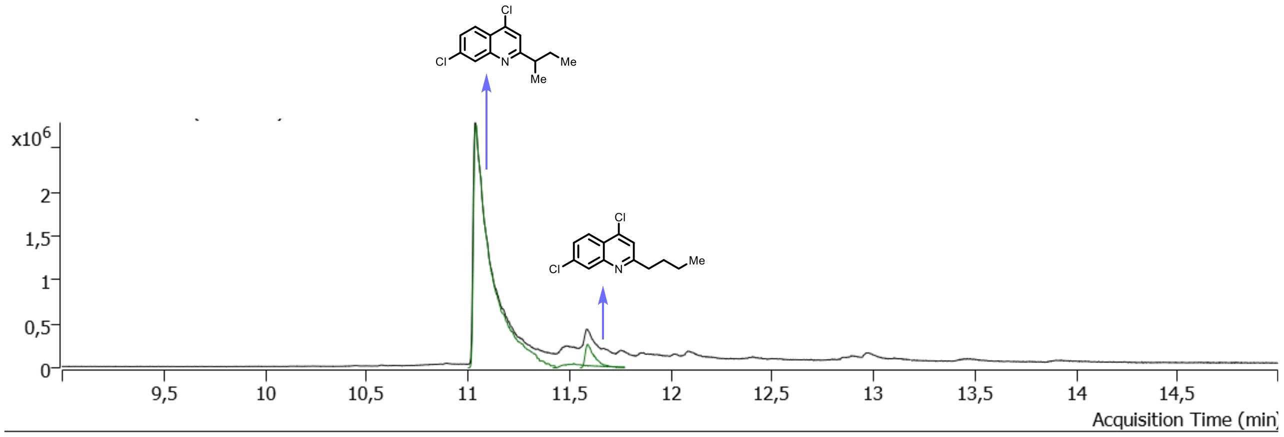

Peak	RT	Peak Height	Peak Area	Area %
1	11.0	1487333,6	16791132,9	97 %
2	11.5	32643,4	502602,8	3 %
